# Glycoconjugation of Betulin Derivatives Using Copper-Catalyzed 1,3-Dipolar Azido-Alkyne Cycloaddition Reaction and a Preliminary Assay of Cytotoxicity of the Obtained Compounds

**DOI:** 10.3390/molecules25246019

**Published:** 2020-12-18

**Authors:** Mirosława Grymel, Gabriela Pastuch-Gawołek, Anna Lalik, Mateusz Zawojak, Seweryn Boczek, Monika Krawczyk, Karol Erfurt

**Affiliations:** 1 Department of Organic Chemistry, Bioorganic Chemistry and Biotechnology, Silesian University of Technology, B. Krzywoustego 4, 44-100 Gliwice, Poland; gabriela.pastuch@polsl.pl (G.P.-G.); mateuszzawojak@wp.pl (M.Z.); seweryn.boczek16@gmail.com (S.B.); monika.krawczyk@polsl.pl (M.K.); 2 Biotechnology Center, Silesian University of Technology, B. Krzywoustego 8, 44-100 Gliwice, Poland; anna.lalik@polsl.pl; 3 Department of Systems Biology and Engineering, Silesian University of Technology, Akademicka 16, 44-100 Gliwice, Poland; 4 Department of Chemical Organic Technology and Petrochemistry, Silesian University of Technology, B. Krzywoustego 4, 44-100 Gliwice, Poland; karol.erfurt@polsl.pl

**Keywords:** betulin glycoconjugates, click chemistry, 1,3-dipolar cycloaddition, anticancer activity

## Abstract

Pentacyclic lupane-type triterpenoids, such as betulin and its synthetic derivatives, display a broad spectrum of biological activity. However, one of the major drawbacks of these compounds as potential therapeutic agents is their high hydrophobicity and low bioavailability. On the other hand, the presence of easily transformable functional groups in the parent structure makes betulin have a high synthetic potential and the ability to form different derivatives. In this context, research on the synthesis of new betulin derivatives as conjugates of naturally occurring triterpenoid with a monosaccharide via a linker containing a heteroaromatic 1,2,3-triazole ring was presented. It has been shown that copper-catalyzed 1,3-dipolar azide-alkyne cycloaddition reaction (CuAAC) provides an easy and effective way to synthesize new molecular hybrids based on natural products. The chemical structures of the obtained betulin glycoconjugates were confirmed by spectroscopic analysis. Cytotoxicity of the obtained compounds was evaluated on a human breast adenocarcinoma cell line (MCF-7) and colorectal carcinoma cell line (HCT 116). The obtained results show that despite the fact that the obtained betulin glycoconjugates do not show interesting antitumor activity, the idea of adding a sugar unit to the betulin backbone may, after some modifications, turn out to be correct and allow for the targeted transport of betulin glycoconjugates into the tumor cells.

## 1. Introduction

For several decades, natural products (*NPs*) have been widely researched in terms of searching for potential therapeutic agents. One important class of natural plant products is pentacyclic lupane-type triterpenoids, among them betulin (*BN*). In recent years, due to a strong biological activity and low toxicity, the great interest of scientists has been focused on its naturally occurring bioactive skeleton, as evidenced by numerous new semi-synthetic derivatives reported every year. Betulin (*BN*, 3-lup-20(29)-ene-3,28-diol) is cheap, easily accessible from natural resources, and can be readily extracted from the bark of several species of trees, especially from the white birch (*Betula pubescens*) [1,2]. *BN* possesses a broad spectrum of biological activity confirmed by its anticancer [3,4,5,6,7,8,9,10,11,12,13], antibacterial [14,15], anti-HIV [14,16], anti-inflammatory [17,18,19], antiviral [20,21], antimalarial activity [17,19], and hepatoprotective properties [9,22,23].

**Figure 1:**
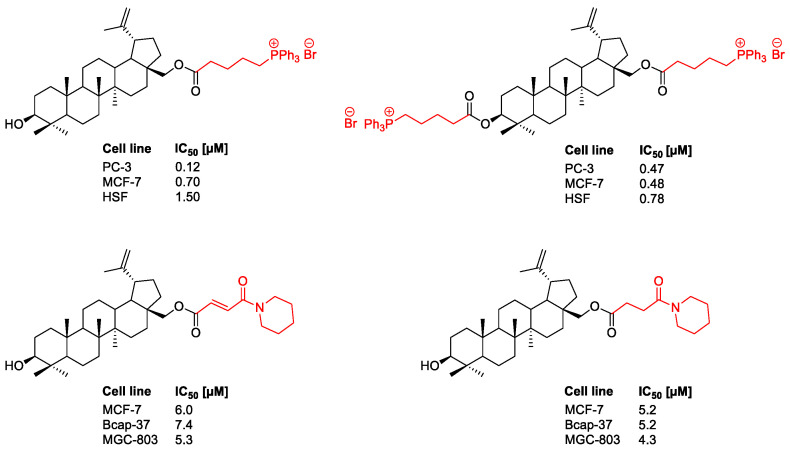
Antitumor activities of betulin derivatives against human breast carcinoma (MCF-7 and Bcap-37), human gastric carcinoma (MGC-803), human prostate carcinoma (PC-3), and human skin fibroblast (HSF) cell lines [12,27].

One of the major drawbacks of betulin and analogs as potential therapeutic agents is its poor hydrophilicity, poor solubility in aqueous media like blood serum and polar solvents used for bioassays, and low bioavailability. However, due to the presence of easily transformable functional groups (including C3OH, C28OH) in the parent structure, betulin has a high synthetic potential and the ability to form numerous semisynthetic derivatives. Thus, the parent structure of betulin, being a biologically attractive scaffold with a high safety profile, makes it possible to carry out a variety of structural modifications to improve its pharmacokinetic properties. In some cases [5,6,9,12,24,25,26], the transformations gave compounds with a higher bioavailability and higher biological activity than the parent compound (Figure 1).

**Scheme 1:**
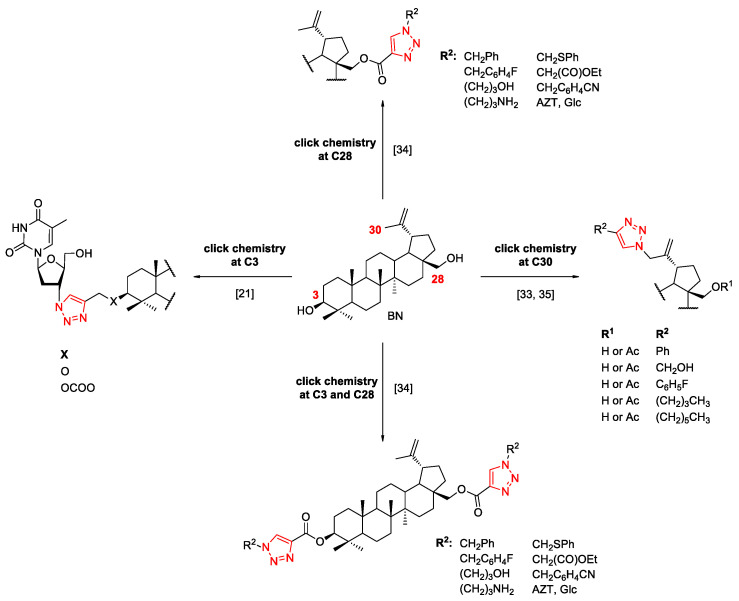
Functionalization of betulin at the C3, C28, and C30 position via CuAAC [33,34,35].

One of the strategies to improve the pharmacokinetic properties of betulin is its link to the 1,2,3-triazole ring, which is an important five-membered heterocyclic scaffold due to the extensive biological activities. This framework can be obtained through the copper-catalyzed azide-alkyne cycloaddition (CuAAC). In addition, the 1,2,3-triazole ring can be successfully used for conjugation in mild conditions and with high yields, with two or more molecules of interest. Due to the stability of triazoles in typical physiological conditions, they act as ideal linkers and could improve the hydrophilic property of the parent skeleton of NPs via the ability to create hydrogen bonding. The 1,2,3-triazole moiety mimics the naturally occurring amide bonds, and at the same time, it is more stable [28,29,30,31]. This methodology allows the functionalization of *BN* at position C3, C28, and C30, with different substrate (Scheme 1). Although the triazole derivatives of betulin have been widely researched, no structures have been found so far that would demonstrate cytotoxicity at a level that would allow them to be used as drugs [32].

**Figure 2:**
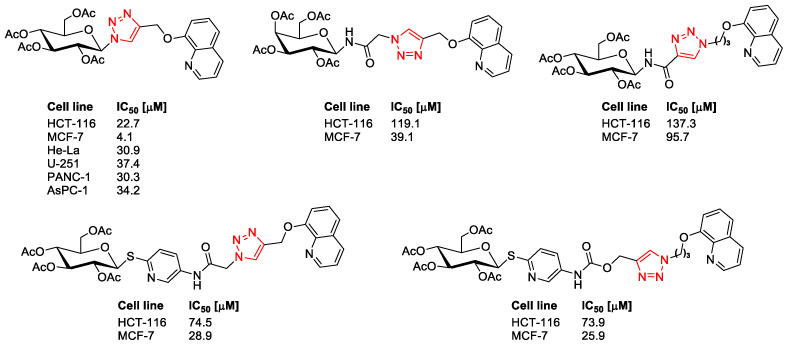
Selected quinoline glycoconjugates with anticancer activity.

The motivation for our research was the successes in the synthesis of molecular hybrids presented in Figure 2, obtained with the use of a natural biologically active compound, where the introduction of a triazole ring and a sugar unit into the parent structure of quinoline resulted in the improvement of pharmacological properties of the obtained glycoconjugates [36,37,38,39].

The presence of a sugar unit in the hybrid molecules improves their pharmacokinetic properties, including solubility and intermembrane transport, as well as the selectivity in targeting drugs for a specific purpose. Sugar moieties are known to influence the pharmacokinetic properties of the respective compounds, such as absorption, distribution, and metabolism [40]. Since it is generally accepted that glycosides are more water soluble than the respective aglycones, glycosidation of triterpenes should increase hydrosolubility and improve the pharmacological properties. Furthermore, it is known that cancerous cells need a more significant sugar contribution than normal cells [41]. It is related to the specific metabolism of glucose by cancer cells as well as with their often-increased proliferation compared to healthy cells. This phenomenon is known as the Warburg effect and arises from mitochondrial metabolic changes. It consists in the fact that cancer cells produce their energy through glycolysis followed by lactic acid fermentation, characteristic of hypoxic conditions, and its level is much higher (more than 100 times) than in healthy cells, for which the main source of energy is mitochondrial oxidative phosphorylation [42]. An increased glycolysis process in cancer cells is associated with overexpression of GLUT transporters (special transmembrane proteins that facilitate concentration-dependent glucose uptake inside the cell) [43]. Consequently, this difference could be exploited to support the absorption of the therapeutic agent by the tumoral site, which allows for controlled drug delivery.

**Scheme 2:**
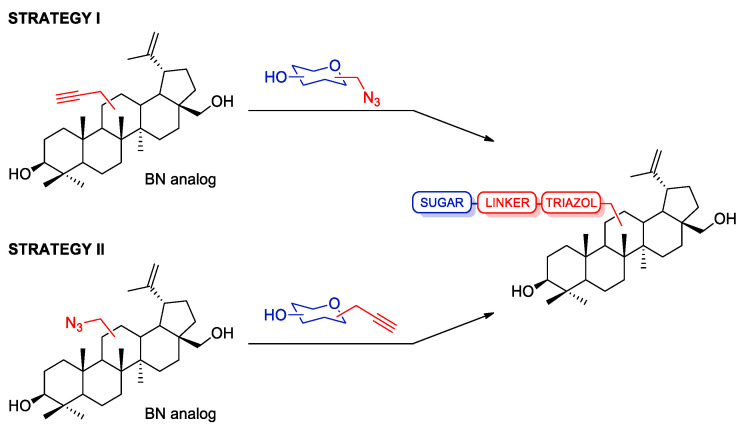
Strategies for the synthesis of betulin glycoconjugates.

Our research group decided to focus on the synthesis of the new betulin glycoconjugates via click chemistry reaction. We designed semi-synthetic betulin derivatives as a combination of naturally occurring triterpenoid (BN) with proven broad biological activity, with a monosaccharide through a linker containing a heteroaromatic 1,2,3-triazole ring. The choice of the sugar unit for glycoconjugation was determined by the willingness to increase the selectivity of potential drugs by using the overexpression of GLUT transporters in some types of cancers. These transporters also recognize other sugars, such as galactose, mannose, and glucosamine [44]. It was decided to attach either D-glucose or D-galactose units due to the common occurrence of these sugars in nature. These sugars were supposed to perform the function of a drug carrier across the cell membrane by adapting to the structure of the GLUT transporter [43]. The configuration at the anomeric center of the sugar also seems important from the point of view of matching with the transporter. The results of research on the structural requirements for binding variously substituted sugars to the sugar transporters indicate that the β-configuration is preferred for binding to the GLUT transporters [45,46]. In order to obtain a library of compounds intended for the initial assessment of biological cytotoxicity, we tested two strategies for conjugating the sugar unit with the parent skeleton of betulin, both based on the 1,3-dipolar azido-alkyne cycloaddition reaction (Scheme 2).

**Figure 3:**
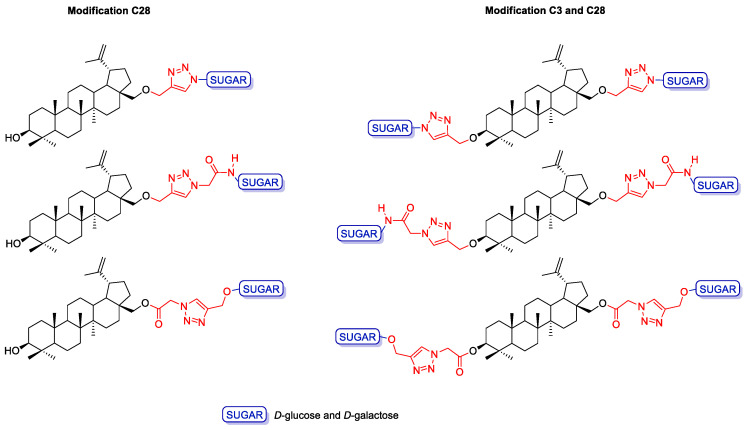
General structure of the betulin glycoconjugates.

*Strategy I* involves the introduction of a propargyl moiety into the betulin structure at position C28 (monosubstituted betulin analogs) or C3 and C28 (disubstituted betulin analogs), followed by the synthesis of glycoconjugates containing the 1,2,3-triazole ring, resulting from combining propargyl betulin derivatives and appropriate sugar derivatives containing an azide moiety. *Strategy II*, on the other hand, consists of adding an appropriate linker (O(CO)CH_2_N_3_) to the betulin backbone one at position C28 or at two positions, C3 and C28, and the synthesis of glycoconjugates via click chemistry with the use of betulin azides and propargyl sugar derivatives. The linker that connects the betulin scaffold to the sugar unit is an important element that may influence the potential biological activity of the discussed betulin glycoconjugates. In this study, we report the synthesis of several glycoconjugates, having a linker containing a 1,2,3-triazole moiety and an additional ether (*O*-glycosidic bond), ester, or amide moiety (Figure 3). The triazole ring seems to be an ideal linker component as it improves water solubility, thus allowing in vivo administration; it is analogous to an amide function for its electronic properties but simultaneously is resistant to hydrolysis, it is sufficiently stable in biological systems, and finally, it is rigid enough, which allows avoidance of internal interaction between the two linked moieties. On the other hand, the presence of a glycosidic, amide, or ester linkage gives a chance for the hydrolysis of the glycoconjugate into its biologically active components after the compound enters the cell under the action of hydrolytic enzymes. The cytotoxicity of the obtained compounds was evaluated on a human breast adenocarcinoma cell line (MCF-7) and colorectal carcinoma cell line (HCT 116).

## 2. Results and Discussion

### 2.1. Synthesis of Betulin Analogs

**Scheme 3:**
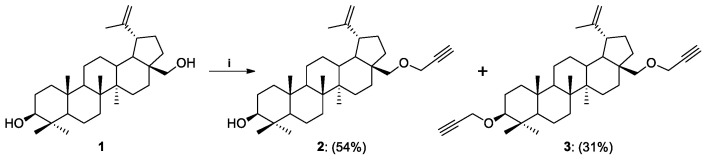
Synthesis of propargyl derivatives of betulin **2** and **3**. Reagents and Conditions: (**i**) propargyl bromide, NaH, THF, r.t., 24 h.

The introduction of a propargyl moiety into the betulin molecule was based on the procedure described by Khan et al. for betulinic acid [47]. It was assumed that both mono- and dipropargyl derivatives of betulin could be formed due to the presence in the substrate of the primary OH group at the C28 position and the secondary OH group at the C3 position. The propargylated betulin was prepared as shown in Scheme 3. Crystalline betulin was reacted with propargyl bromide in alkaline medium (NaH) in tetrahydrofuran. In the search for the optimal procedure, a number of modifications were made, by selecting the proportions of reagents, reaction time, and temperature. It was found that it is advantageous to use the reagents in a molar ratio (BN/NaH/HC≡CCH_2_Br, 1:4:3.2) at ambient temperature for 24 h.

**Scheme 4:**
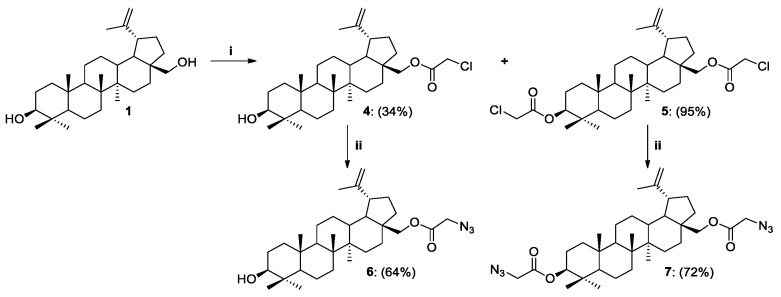
Synthesis of betulin derivatives **4**–**7**. Reagents and Conditions: (**i**) Cl(CO)CH_2_Cl, DIPEA, DMAP, THF, r.t., 24 h; (**ii**) NaN_3_, DMF, 90 °C, 3 h.

The synthesis of 28-*O*-chloroacetylbetulin **4** and 3,28-*O,O’*-di(chloroacetyl)betulin **5** was performed by modifying the procedure described by Komissarova et al. in 2017 [48]. Chloroacetic chloride was added dropwise to the betulin solution in THF, while triethylamine (Et_3_N) was replaced with *N,N*-diisopropylethylamine (DIPEA) and 4-(dimethylamino)pyridine (DMAP). We found that the application of twice the excess of chloroacetic chloride to BN results in the formation of only 3,28-*O,O’*-di(chloroacetyl)betulin **5** with a high yield (95%, Scheme 4). On the other hand, when the reagents were used in a molar ratio (Cl(CO)CH_2_Cl/DIPEA/DMAP, 1.2:1.5:0.1), a modification of the betulin skeleton was observed at both the C28 and C3 positions. The maximum yield with which a monosubstituted product **4** can be obtained is 34%. Replacement of the chlorine anion in betulin analogs **4** and **5** by azide moiety was performed at an elevated temperature (90 °C) using NaN_3_ and DMF as a solvent. 28-*O*-Azidoacetylbetulin **6** and 3,28-*O,O’*-di(azidoacetyl)betulin **7** were purified by column chromatography to give pure compounds with 64% and 72% yields, respectively (Scheme 4, Table 1).

### 2.2. Synthesis of Sugar Derivatives

**Scheme 5:**
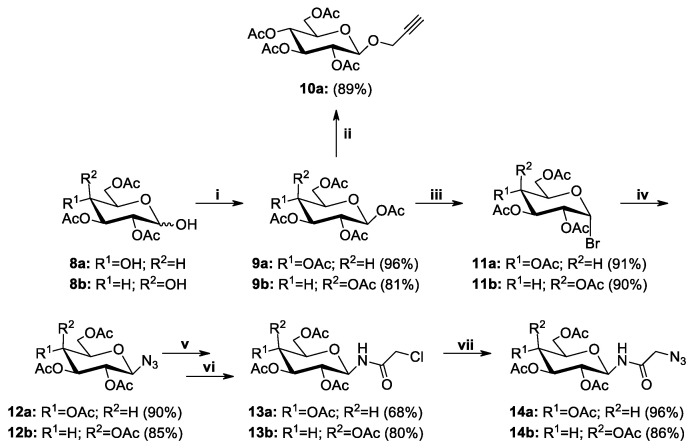
Synthesis of sugar derivatives **9**–**14**. Reagents and Conditions: (**i**) CH_3_COONa, Ac_2_O, b.p., 1 h; (**ii**) propargyl alcohol, BF_3_·Et_2_O, DCM, 0 °C→r.t, 2 h; (**iii**) CH_3_COOH, 33% HBr/AcOH, r.t., 1 h; (**iv**) NaN_3_, TBASH, CHCl_3_/NaHCO_3_, r.t., 2 h; (**v**) 20% Pd(OH)_2_/C, THF:EtOH (2:1, *v*/*v*), H_2_, 1.5 bar, 2 h; (**vi**) chloroacetyl chloride, TEA, DCM, r.t., 1 h; (**vii**) NaN_3_, DMF, r.t., 24 h.

Sugar derivatives substituted at the anomeric position were the second necessary structural element for the synthesis of betulin glycoconjugates. The synthetic route to the corresponding derivatives of D-glucose and D-galactose **9**–**14** is shown in Scheme 5. As mentioned before, the choice of such sugar moieties is dictated by the frequency of their natural occurrence as well as their importance for cell metabolism. All sugar derivatives were prepared according to known procedures involving the acetylation of free sugars **8a** or **8b** and conversion of per-*O*-acetylated derivatives **9a** or **9b** into the corresponding glycosyl bromides **11a** or **11b [49,50].** The glycosyl bromides were used for further reactions leading to obtain 2,3,4,6-tetra-*O*-acetyl-β-glycosyl azides **12a** and **12b [36]**. D-Glucose derivative **10a**, in which the alkynyl moiety was introduced by the formation of an *O*-glycosidic linkage, was prepared by reacting per-*O*-acetylated D-glucose **9a** with propargyl alcohol in the presence of BF_3_·Et_2_O as a Lewis acid catalyst [51]. Sugar derivatives **13a** and **13b** containing an amide moiety at the sugar anomeric position were obtained in a two-step procedure. First, glycosyl azides **12a** and **12b** were converted to the corresponding 1-aminosugars through a hydrogenation reaction in a Parr apparatus using palladium hydroxide deposited on activated charcoal and then such obtained intermediates were reacted with chloroacetyl chloride in the presence of TEA, which neutralized the formed hydrogen chloride. Finally, the terminal chlorine atom in compounds **13a** and **13b** was exchanged with an azide group, which made it possible to obtain sugar derivatives **14a** and **14b** as a result of the reaction with sodium azide in anhydrous DMF [37].

### 2.3. Modifications of the Natural Betulin Skeleton via Click Chemistry

**Scheme 6:**
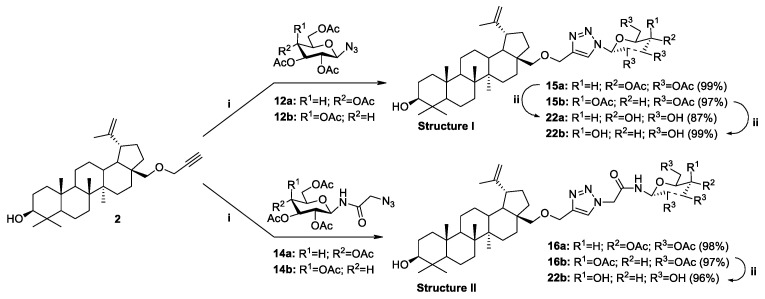
Synthesis of glycoconjugates type I and II according to Strategy I. Reagents and Conditions: (**i**) CuSO_4_·5H_2_O, NaAsc, *i*-PrOH/H_2_O (1:1, *v*/*v*), r.t., 24 h; (**ii**) 1. NaOMe, MeOH, r.t., 0.5 h; 2. *Amberlyst*-15.

**Scheme 7:**
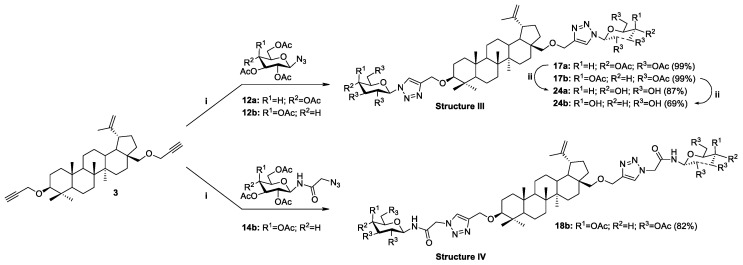
Synthesis of glycoconjugates type **III** and **IV** according to Strategy I. Reagents and Conditions: (**i**) CuSO_4_·5H_2_O, NaAsc, i-PrOH/H_2_O (1:1, *v*/*v*), r.t., 24 h; (**ii**) 1. NaOMe, MeOH, r.t., 0.5 h: 2. *Amberlyst-15*.

**Scheme 8:**
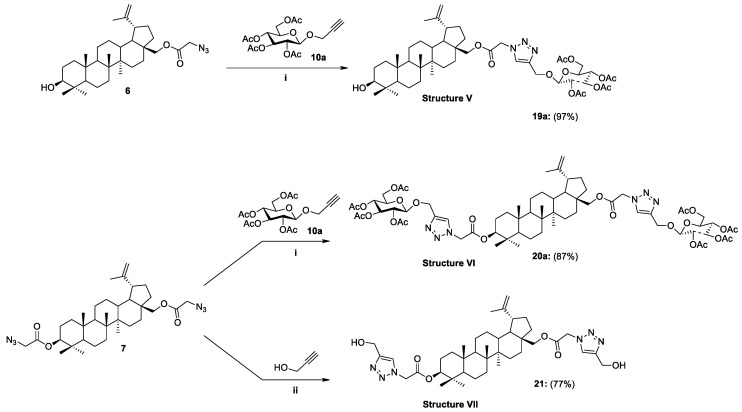
Synthesis of glycoconjugates type **V–VII** according to Strategy II. Reagents and Conditions: (**i**) CuSO_4_·5H_2_O, NaAsc, *i*-PrOH/THF/H_2_O, r.t., 24 h, mol. ratio **6**/**10a**/CuSO_4_·5H_2_O/NaAsc, 1:1:0.5:1.1; **7**/**10a**/CuSO_4_·5H_2_O/NaAsc, 1:2:1.1:0.6; (**ii**) propargyl alcohol, CuSO_4_·5H_2_O, NaAsc, *i*-PrOH/THF/H_2_O (1.6:1:1.1, *v*/*v*/*v*), r.t., 24 h, mol ratio **7**/C_3_H_4_O /CuSO_4_·5H_2_O/NaAsc, 1:3:1.2:2.2.

The copper-catalyzed azide-alkyne cycloaddition reaction (CuAAC) is widely used to modify natural bioactive compounds, and its main advantage is the ability to synthesize new molecular hybrids with high yields under mild conditions. In the course of our research, we developed a methodology for the synthesis of new previously unknown betulin derivatives, consisting of joining the triterpenoid skeleton with a monosaccharide through an appropriate linker containing a heteroaromatic 1,2,3-triazole ring. Glycoconjugation of betulin derivatives was based on the click chemistry reaction (CuAAC) using propargylbetulin derivatives **2**, **3** and protected D-glucose **12a**, **14a** or D-galactose **12b**, **14b** derivatives containing an azide group (Strategy I, Scheme 6 and Scheme 7) or propargyl *O*-glycoside derivative of *per*-*O*-acetylated-D-glucose **10a** with appropriate betulin azides **6**, **7** (Strategy II, Scheme 8). Building blocks designed in this way make it possible to obtain a whole range of betulin glycoconjugates, containing in the linker structure different combinations of an *O*-glycosidic linkage, an ester bond, an *O*-methylene 1,2,3-triazole linker, and an amide moiety.

The general procedure of glycoconjugation of betulin derivatives involves mixing of 28-*O*-propargylbetulin **2** or 3,28-*O,O’*-di(propargyl)betulin **3** with the appropriate monosugar derivative (D-glucose or D-galactose) in the water-alcohol solvent system (*i*-PrOH/H_2_O). In the case of 28-*O*-azidoacetylbetulin **6** and 3,28-*O,O’*-di(azidoacetyl)betulin **7**, due to the too low solubility of the substrates in the *i*-PrOH/H_2_O solvent system, it was necessary to use an additional solvent (THF). In each case, the reaction was carried out under an argon atmosphere at room temperature for 24 h, using CuSO_4_·5H_2_O as a catalyst and sodium ascorbate (NaAsc) as an agent reducing Cu(II) to Cu(I).

As a result of the described reactions, five new monosubstituted betulin derivatives (**15a**, **15b**, **16a**, **16b**, and **19a**) modified at position C28, as well as four new disubstituted betulin derivatives (**17a**, **17b**, **18b**, and **20a**), modified at positions C3 and C28 were isolated by column chromatography in very good yields (97–99% and 82–99%, respectively, Table 2).

It has been considered that the use of an acyl protected sugar moiety may not be sufficient to achieve the desired hydrophilicity of the novel BN analogs. In order to compare the properties of glycoconjugates and assess the effect of acyl group presence, for the five per-*O*-acetylated glycoconjugates BN (**15a, 15b**, **16a, 16b, 17b**), the deprotection of the sugar unit was performed by applying a standard Zemplén procedure [52], under mild conditions using 1 M methanolic solution of sodium methoxide in methanol. The reaction was carried out at room temperature for 120 min. The final step was to neutralize the reaction mixture with the use of *Amberlyst-15* ion exchange resin, after which the mixture was filtered to give betulin glycoconjugates (**22a, 22b**, **23b, 24a**) in high yield (87-99%), except compound **24b** (69%), as shown in Table 2.

Our proposed concept of adding a linker to the scaffold of betulin is based on introducing a chloroacetyl moiety into its structure and then converting the obtained analogs into azidoacetyl derivatives. In the case of the analog **21**, in the final stage, the azide moiety was coupled with propargyl alcohol using the click chemistry reaction. As a result, a 3,28-disubstituted betulin **21** modified with a linker containing a 1,2,3-triazole ring substituted in the C4 position with a hydroxymethyl group was obtained in a yield of 77% (Scheme 8). The motivation to obtain a 3,28-*O,O’*-di(2-(4-(hydroxymethyl-*1H*-1,2,3-triazol-1-yl)acetyl)betulin **21** was the desire to check whether the addition of a sugar unit is necessary to improve the biological properties of BN or whether the introduction of the linker containing the 1,2,3-triazole ring alone would be sufficient. The structures of all synthesized compounds were confirmed by nuclear magnetic resonance (^1^H-, ^13^C-NMR, gHSQC), infrared spectroscopy (FT-IR), and high-resolution mass spectrometry (HRMS).

### 2.4. Cytotoxicity Studies

The obtained betulin glycoconjugates were screened to determine their cytotoxicity. The research was conducted on two cell lines: HCT 116 (colorectal carcinoma cell line) and MCF-7 (human breast adenocarcinoma cell line). In these lines, overexpression of the glucose and galactose transporters was observed [53,54,55]. The aim of the research was to initially estimate the influence of the modifications of the parent betulin backbone by adding a sugar unit/units, as well as to examine the influence of the structure of linker connecting betulin with sugar on the cytotoxicity of the tested compounds.

**Figure 4:**
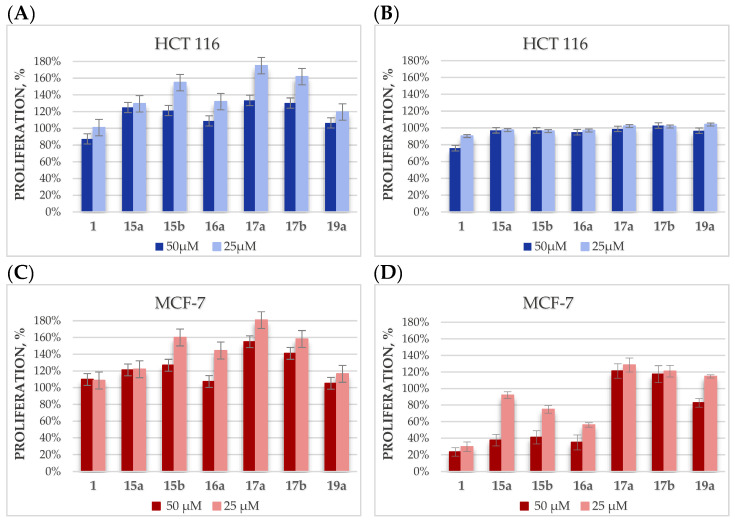
The dependence of HCT 116 and MCF-7 cell proliferation on the concentration of the betulin glycoconjugates, after 24 (**A,C**) or 48 h (**B,D**) of incubation.

In the first stage, betulin glycoconjugates in which the hydroxyl groups of the sugar unit were protected with acetyl groups were selected for research. The choice of the type of hydroxyl group protection was dictated by the sensitivity of acetyl groups to the action of hydrolytic enzymes and the fact that these groups provide glycoconjugates with increased hydrophobicity, which should facilitate passive transport into the cell. The proliferation of tumor cells (HCT 116, MCF-7) treated with tested compounds (**15a**, **15b**, **16a**, **17a**, **17b**, **19a**) at 50- and 25-µM concentrations were determined after 24 and 48 h of incubation with the Cell Counting Kit-8 (CCK-8) based on the water-soluble tetrazole salt of WST-8. The CCK-8 kit, selected for the determination of viability and cytotoxicity, is less toxic and has higher sensitivity than other tetrazole salt-based tests, such as MTT or MTS [56]. The effect of these compounds was compared with the effect of betulin **1** doses, as shown in Figure 4.

Unfortunately, in the case of the HCT 116 cell line, it was found that the designed betulin glycoconjugates, both at 24 and 48 h, did not inhibit the proliferation of tumor cells. Additionally, it was observed that in some cases, the tested compounds even stimulate tumor cells to grow faster. It is especially visible after 24 h for analogs **15b**, **17a**, and **17b** (Figure 4A). Our hypothesis explaining this fact is based on the assumption that these derivatives are able to enter cancer cells, where they are degraded by hydrolytic enzymes with the release of sugar molecules, which is an additional source of energy allowing the proliferation growth. In the case of the MCF-7 cell line, after 24 h of incubation with the tested glycoconjugates, a similar effect was observed as for the HCT 116 cell line. However, after 48 h, a concentration-dependent cytotoxic effect of the glycoconjugates **15a**, **15b**, and **16a** was observed (Figure 4D). These glycoconjugates, given to cells at the 50-µM concentration, showed cytotoxicity comparable to that of betulin. In glycoconjugates **15a**, **15b**, and **16a**, the betulin skeleton was modified at position C28 by adding a sugar unit via an *O*-methylene-1,2,3-triazole linker or the same linker extended with an *N*-methyleneamide group. Presumably, the protection of the sugar hydroxyl groups improved the passive transport of the glycoconjugates inside the cell, where they released betulin under the action of hydrolytic enzymes. In the case of the disubstituted analogs BN **17a** and **17b**, modified at both the C3 and C28 positions, such a beneficial effect was not observed, which may be related to the prolonged effect of the release of as many as two glucose molecules (per each unit of betulin), which are the source of energy for increased proliferation. Quite unexpectedly, for the monosubstituted glycoconjugate **19a**, no significant cytotoxic activity was observed on tumor cells. Perhaps, the ester bond connecting the betulin backbone with the sugar unit is too labile and some part of the compound undergoes hydrolysis even before it penetrates the cell.

In order to check whether the introduction of the sugar unit increases the affinity of the obtained molecules for GLUT transporters, the acetyl protections in the sugar fragment of the obtained glycoconjugates were removed, and then cytotoxicity tests of the obtained compounds were carried out on the same cell lines (HCT 116, MCF-7). The cytotoxicity of the disubstituted BN derivative **21**, in which a linker contains only the 1,2,3-triazole system, without a sugar unit, was also tested. This study was designed to check the influence of the presence of the 1,2,3-triazole ring on the activity of the betulin derivative.

**Figure 5:**
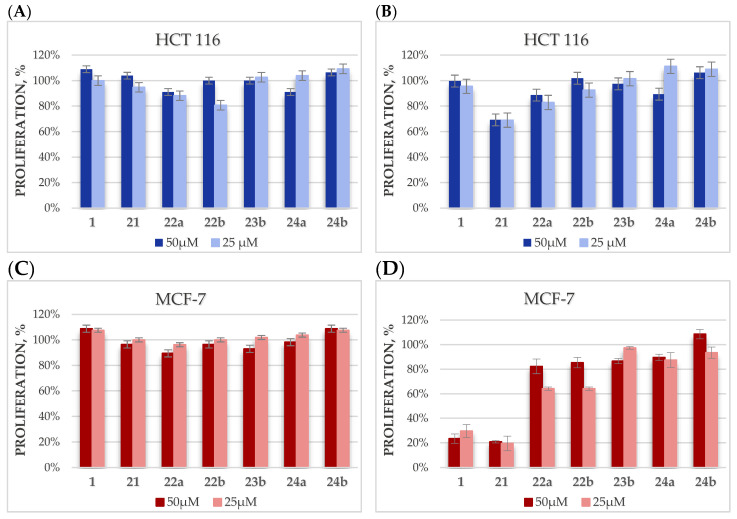
The dependence of HCT 116 and MCF-7 cell proliferation on the concentration of the betulin glycoconjugates, after 24 (**A**,**C**) or 48 h (**B**,**D**) of incubation.

When the research was carried out on the HCT 116 cell line, both after 24 h and after 48 h, no significant effect of inhibition of tumor cell proliferation by the tested glycoconjugates was observed (**22a**, **22b**, **23b**, **24a**, **24b**). Interestingly, derivative **21** after 48 h shows slightly higher cytotoxicity compared to the activity of the parent BN backbone (Figure 5B). In the case of the MCF-7 cell line, after 24 h of incubation, the effect caused by the glycoconjugates (**22a**, **22b**, **23b**, **24a**) is only slightly better than that observed with BN. However, already after 48 h of incubation, the significantly higher cytotoxic activity of BN was observed compared to glycoconjugates. Surprisingly, as was in the case with the HCT 116 cell line, BN derivative **21** without a sugar unit, but only with an attached linker with the 1,2,3-triazole system, proved to be the most active. After 48 h of incubation, only 20% cell proliferation for this compound was observed as shown in Figure 5D. 

In the course of further studies, for betulin **1** and its derivatives **15a**, **15b**, **16a,** and **21**, the ability to inhibit the proliferation of MCF-7 cells in a wider spectrum of concentrations was carried out in order to determine their IC_50_ value. The same compounds were also tested against NHDF cell line (normal human dermal fibroblast-neonatal cells), to assess the safety of their uses. Assays were performed in the concentration range of 50–6.25 µM for both tested cell lines. Unfortunately, it turned out that glycoconjugates **15a**, **15b**, and **16a** are equally cytotoxic in relation to healthy cells and to the tested tumor lines (Table 3). Additionally, the most active of betulin derivative **21**, even at the lowest tested dose, is extremely cytotoxic and allows the proliferation of both cell lines to be kept at only 20%. The cytotoxicity of compound **21** significantly exceeds that of betulin, which may suggest that further modification of this compound is necessary to improve its selectivity. 

### 2.5. Cytotoxic Activity Predicted Lipophilicity 

In medicinal chemistry, the aqueous solubility and lipophilicity of a drug are important molecular parameters determining the absorption and the bioavailability. The lipophilicity is indicated by the logarithm of a partition coefficient (logP), which reflects the concentration ratio of the drug at equilibrium partitioning between octanol and water phases [57,58].

Betulin **1**, which is the subject of our research, is characterized by a highly limited solubility in polar solvents. This property may result from the complex structure, the presence of five fused aliphatic rings, and the isopropenyl moiety. The only polar fragments of the molecule are the two hydroxyl groups at C3 and C28. In this study, in order to determine the effect of various substituents on the value of the logP coefficient for several betulin derivatives, we used the prediction base *MolInspirations* to evaluate in silico the parameter (Table 4). 

**Figure 6:**
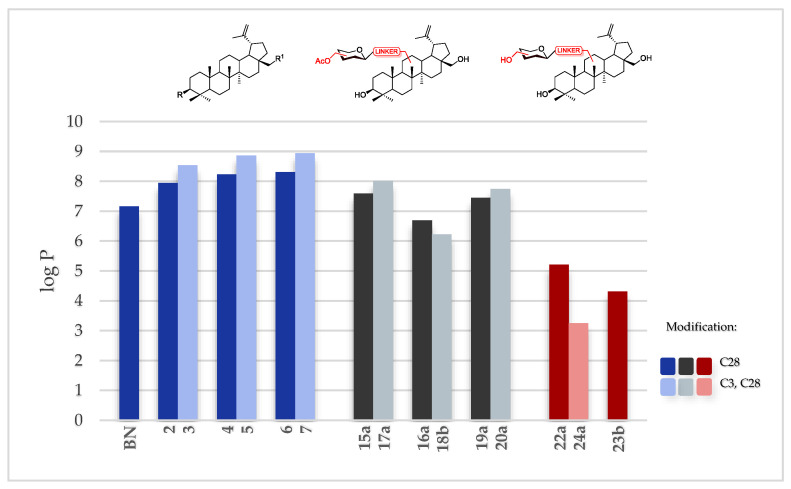
LogP of BN and betulin analogs **2**–**24**.

As we expected, adding a sugar moiety decreases the lipophilicity (<logP) of betulin analogs. Moreover, in the case of triazole derivatives of betulin modified with a sugar unit, the difference was revealed when the structures containing a per-*O*-acetylated sugar unit **15**–**20** and an unprotected sugar unit **22**–**24** were compared. The linker type is also crucial. For compounds bearing a substituent of the OCH_2_TriazGlc/Gal type at C3 and C28 position, the logP values are higher than for compounds bearing substituents with an ester moiety in the same position. On the other hand, the lowest values of the coefficient have derivatives in which an amide group is present in the linker structure. Only betulin glycoconjugates **23** and **24** have logP values smaller than 5 (Figure 6).

## 3. Materials and Methods 

### 3.1. General Information

NMR spectra were recorded on a Varian spectrometer at operating frequencies of 600 or 400 and 150 or 100 MHz, respectively, using TMS as the resonance shift standard and NMR solvents (CDCl_3_, CD_3_OD or DMSO-*d*_6_), which was purchased from ACROS Organics (Geel, Belgium). All chemical shifts (δ) are reported in ppm and coupling constants (*J*) in Hz. The following abbreviations were used to explain the observed multiplicities: s: singlet, d: doublet, dd: doublet of doublets, ddd: doublet of doublet of doublets, t: triplet, dd~t: doublet of doublets resembling a triplet (with similar values of coupling constants), m: multiplet, br: broad. IR-spectra were measured on an FTIR spectrophotometer (ATR method). High-resolution mass spectrometry (HRMS) analyses were performed on a Waters LCT Premier XE system using the electrospray-ionization (ESI) technique. Optical rotations were measured with a JASCO P-2000 polarimeter using a sodium lamp (589.3 nm) at room temperature. Melting points were determined using a Boethius M HMK hot-stage apparatus. Reactions were monitored by TLC on precoated plates of silica gel 60 F254 (Merck Millipore). TLC plates were inspected under UV light (λ = 254 nm) or charring after spraying with 10% solution of sulfuric acid in ethanol. Crude products were purified using column chromatography performed on Silica Gel 60 (70–230 mesh, Fluka).

Cell viability was measured using a Cell Counting Kit-8 (Bimake) according to the manufacturer’s protocol. The absorbance on the CKK-8 assay was measured spectrophotometrically at the 450-nm wavelength using a plate reader (Epoch, BioTek, USA).

28-*O*-Chloroacetylbetulin **4** and 3,28-*O,O’*-di(2-chloroacetyl)betulin **5** [48], 1,2,3,4,6-penta-*O*-acetyl-β-D-glucopyranose **9a**, 1,2,3,4,6-penta-*O*-acetyl-β-D-galactopyranose **9b** [53], propargyl 2,3,4,6-tetra-*O*-acetyl-β-D-glucopyranoside **10a** [55], 2,3,4,6-tetra-*O*-acetyl-α-D-glucopyranosyl bromide **11a**, 2,3,4,6-tetra-*O*-acetyl-α-D-galactopyranosyl bromide **11b** [54], 2,3,4,6-tetra-*O*-acetyl-β-D-glucopyranosyl azide **12a**, 2,3,4,6-tetra-*O*-acetyl-β-D-galactopyranosyl azide **12b** [36], 2,3,4,6-tetra-*O*-acetyl-*N*-(β-D-glucopyranosyl)chloroacetamide **13a**, 2,3,4,6-tetra-*O*-acetyl-*N*-(β-D-galacto-pyranosyl)chloroacetamide **13b**, 2,3,4,6-tetra-*O*-acetyl-*N*-(β-D-glucopyranosyl)azidoacetamide **14a**, and 2,3,4,6-tetra-*O*-acetyl-*N*-(β-D-galactopyranosyl)azidoacetamide **14b** [37] were prepared according to the respective published procedures. All used chemicals were purchased from Sigma-Aldrich, Fluka, Avantor, and ACROS Organics and were used without purification.

### 3.2. Chemistry

#### 3.2.1. Procedure for the Synthesis of Betulin Analogs

##### Procedure for the Synthesis of 28-*O*-propargylbetulin **2** and 3,28-*O,O’*-di(propargyl)betulin **3**

Betulin **1** (0.226 mmol, 100.0 mg) and sodium hydride (NaH, 0.904 mmol, 21.7 mg) were dissolved in dry THF (1.3 mL) followed by addition of propargyl bromide (0.723 mmol, 0.062 mL). The heterogenous reaction mixture was left on the magnetic stirrer for 24 h at room temperature. The reaction progress was monitored on TLC in an eluents system DCM:AcOEt (10:1). Then, NH_4_Cl (0.411 mmol, 22.0 mg, 5 mL H_2_O) was added, extracted with Et_2_O (5 × 4 mL), brine (5 mL) added, and dried over MgSO_4_. After, this was followed by filtration and evaporation in vacuo. The crude products were purified using column chromatography (DCM:AcOEt, gradient: 50:1 to 10:1).

*28-O-Propargylbetulin **2*** was obtained as a white solid (58.8 mg, 54% yield); m.p.: 81—85 °C; [α]^25^_D_ = +11.3 (c 0.5, CHCl_3_); HRMS (ESI^+^): calcd for C_33_H_53_O_2_ ([M + H]^+^): *m/z* 481.4046; found: *m/z* 481.4050; ^1^H NMR (400 MHz, CDCl_3_): δ_H_ 4.68 (s, br, 1H, H-29a), 4.58 (s, br, 1H, H-29b), 4.18 (dd, 1H, *J_1_* 2.4 Hz, *J_2_* 16.0 Hz, OCH_a_C≡), 4.11 (dd, 1H, *J_1_* 2.4 Hz, *J_2_* 16.0 Hz, OCH_b_C≡), 3.70 (d, 1H, *J* 9.2 Hz, H-28a), 3.21-3.15 (m, 2H, H-28b, H-3), 2.45-2.38 (m, 1H, H-19), 2.42 (dd~t, 1H, *J* 2.4 Hz, ≡CH), 2.1-0.60 (m, 25H, CH, CH_2_), 1.68 (s, 3H, H-30), 1.05, 0.97 br, 0.83, 0.76 (all s, 3H each, H-23—H-27) ppm; ^13^C NMR (150 MHz, CDCl_3_) δ_C_ 150.64, 109.56, 80.51, 78.97, 74.01, 68.27, 58.61, 55.28, 50.42, 48.91, 47.99, 42.66, 40.98, 38.86, 38.69, 37.17, 34.69, 34.22, 29.90, 29.79, 27.99, 27.39, 27.14, 25.22, 20.87, 19.09, 18.29, 16.11, 15.98, 15.37, 14.83 ppm (Supplementary Materials, Table S1); IR (ATR) ν: 3350-3200, 2940, 2871, 1640, 1457, 1092, 882 cm^−1^.

*3,28-O,O’-Di(propargyl)betulin **3*** was obtained as a yellow resin (36.5 mg, 31% yield); m.p.: resin; [α]^25^_D_ = +31.9 (c 0.5, CHCl_3_); HRMS (ESI^+^): calcd for C_36_H_55_O_2_ ([M + H]^+^): *m/z* 519.4202; found: *m/z* 519.4202; ^1^H NMR (600 MHz, CDCl_3_): δ_H_ 4.68 (d, 1H, *J* 2.4 Hz, H-29a), 4.58 (dd, 1H, *J_1_* 1.8 Hz, *J_2_* 2.4 Hz, H-29b), 4.21-4.12 (m, 4H, 2x CH_2_C≡), 3.70 (dd, 1H, *J_1_* 1.2 Hz, *J_2_* 8.4 Hz, H-28a), 3.16 (d, 1H, *J* 9.0 Hz, H-28b), 3.00 (dd, 1H, *J_1_* 4.2 Hz, *J_2_* 11.4 Hz, H-3), 2.44-2.39 (m, 1H, H-19), 2.41 i 2.36 (all dd~t, 1H each, *J* 2.4 Hz, ≡CH), 2.10-0.60 (m, 24H, CH, CH_2_), 1.68 (s, 3H, H-30), 1.04, 0.98, 0.97, 0.83, 0.76 (all s, 3H each, H-23—H-27) ppm; ^13^C NMR (150 MHz, CDCl_3_) δ_C_ 150.64, 109.56, 85.86, 80.96, 80.50, 74.01, 73.41, 68.25, 58.62, 56.42, 55.87, 50.41, 48.92, 48.00, 47.02, 42.65, 41.03, 38.55, 37.12, 34.69, 34.24, 29.91, 29.71, 27.99, 27.12, 25.24, 20.91, 18.25, 19.11, 16.10, 16.00, 15.99, 14.79 ppm (Supplementary Materials, Table S1); IR (ATR) ν: 3314, 3261, 2952, 1610, 1453, 1377, 1085, 1068, 881 cm^−1^.

##### Procedure for the Synthesis of 28-*O*-(2-azidoacetyl)betulin **6**

28-*O-*(2-Chloroacetyl)betulin **4** (0.193 mmol, 100.0 mg) was dissolved in DMF (1.93 mL) and sodium azide (0.963 mmol, 62.6 mg) was added. The heterogenous reaction mixture was stirred at 90 °C for 3 h. Then, H_2_O (1 mL) and saturated NaHCO_3_ solution (4 mL) was added. It was extracted with DCM (5 × 4 mL) and dried over MgSO_4_. After that, this was followed by filtration and evaporation in vacuo. The crude product was purified using column chromatography (DCM:MeOH, gradient: 100:1 to 10:1).

*28-O-(2-Azidoacetyl)betulin **6*** was obtained as a white solid (65.0 mg, 64% yield); ^1^H NMR (600 MHz, CDCl_3_): δ_H_ 4.70 (d, 1H, *J* 1.8 Hz, H-29a), 4.60 (d, 1H, *J* 1.2 Hz, H-29b), 4.41 (dd, 1H, *J_1_* 1.2 Hz, *J_2_* 10.8 Hz, H-28a), 3.99 (d, 1H, *J* 11.4 Hz, H-28b), 3.89 (s, 2H, CH_2_N_3_), 3.18 (dd, 1H, *J_1_* 4.8 Hz, *J_2_* 11.4 Hz, H-3), 2.44 (td, 1H, *J_1_* 5.8 Hz, *J_2_* 10.9 Hz, H-19), 2.10–0.60 (m, 25H, CH, CH_2_), 1.68 (s, 3H, H-30), 1.04, 0.98, 0.97, 0.83, 0.76 (all s, 3H each, H-23–H-27) ppm; ^13^C NMR (150 MHz, CDCl_3_) δ_C_ 168.65, 149.76, 109.92, 78.84, 64.31, 55.21, 50.41, 50.26, 48.72, 47.57, 46.33, 42.62, 40.79, 38.77, 38.62, 37.06, 34.39, 34.09, 29.59, 27.89, 27.30, 26.92, 25.09, 20.67, 19.04, 18.19, 16.00, 15.94, 15.27, 14.69 ppm (Supplementary Materials, Table S1).

##### Procedure for the Synthesis of 3,28-*O,O’*-di(2-azidoacetyl)betulin **7**

3,28-*O,O’*-Di(2-chloroacetyl)betulin **5** (0.168 mmol, 100.0 mg) was dissolved in 0.71 mL DMF and sodium azide (0.897 mmol, 58.3 mg) was added. The heterogenous reaction mixture was stirred at 90 °C for 3 h. Then, H_2_O and saturated NaHCO_3_ solution (4 mL) was added. It was extracted with DCM (4 × 4 mL) and dried over MgSO_4_. After that, this was followed by filtration and evaporation in vacuo. The crude product was purified using column chromatography (DCM:MeOH, gradient: 100:1 to 10:1).

*3,28-O,O’-Di(2-azidoacetyl)betulin **7*** was obtained as a white solid (73.6 mg, 72% yield); ^1^H NMR (600 MHz, CDCl_3_): δ_H_ 4.70 (d, 1H, *J* 1.2 Hz, H-29a), 4.61–4.58 (m, 2H, H-29b, H-3), 4.42 (d, 1H, *J* 11.4 Hz, H-28a), 3.98 (d, 1H, *J* 10.8 Hz, H-28b), 3.89 and 3.85 (all s, 2H each, 2x CH_2_N_3_), 2.44 (td, 1H, *J_1_* 5.7 Hz, *J_2_* 10.8 Hz, H-19), 2.10–0.60 (m, 24H, CH, CH_2_), 1.69 (s, 3H, H-30), 1.04, 0.98, 0.87, 0.866, 0.862 (all s, 3H each, H-23–H-27) ppm; ^13^C NMR (150 MHz, CDCl_3_) δ_C_ 168.75, 168.15, 149.84, 110.07, 83.21, 64.40, 55.36, 50.67, 50.52, 50.25, 48.80, 47.69, 46.43, 42.67, 40.91, 38.34, 37.89, 37.06, 34.49, 34.08, 29.67, 27.94, 27.00, 25.06, 23.68, 20.74, 19.05, 18.15, 16.54, 16.15, 16.04, 14.75 ppm (Supplementary Materials, Table S1); IR (ATR) ν: 2942, 2867, 1728, 1243, 1181, 1017, 979, 754 cm^−1^.

#### 3.2.2. General Procedure for the Synthesis of Betulin Glycoconjugates **15**–**16** according to Strategy I (Modification C28)

To a well-stirred 28-*O*-propargylbetulin **2** (0.208 mmol, 100.0 mg) and 2,3,4,6-tetra-*O*-acetyl-β-D-galactopyranosyl or 2,3,4,6-tetra-*O*-acetyl-β-D-glucopyranosyl azide **12a**–**b** (0.208 mmol, 77.7 mg) or **14a–b** (0.208 mmol, 89.5 mg) in *i*-PrOH (2 mL) were added CuSO_4_·5H_2_O (0.125 mmol, 31.2 mg) dissolved in H_2_O (1 mL) and sodium ascorbate (0.229 mmol, 45.3 mg) dissolved in H_2_O (1 mL) succesively. The reaction mixture was stirred under argon atmosphere for 24 h at room temperature. After completion of the reaction (was monitored by TLC), the reaction mixture was extracted with DCM (**15a–b**: 5 × 5 mL) or AcOEt **(16a**–**b**: 5 × 5 mL), then the mixture was dried over MgSO_4_ and was concentrated in vacuo. The crude residue was separated/purified using column chromatography (**15a–b**: DCM:AcOEt, gradient: 20:1 to 1:1; **16a**–**b**: DCM:MeOH, gradient: 50:1 to 10:1).

*Product **15a** (28-OCH_2_TriazGlcBN**)*** was obtained as a white solid (175.1 mg, 99% yield); m.p.: 108–109 °C; [α]^25^_D_ = −21.8 (c 0.5, CHCl_3_); HRMS (ESI^+^): calcd for C_47_H_72_N_3_O_11_ ([M + H]^+^): *m/z* 854.5167; found: *m/z* 854.5165; ^1^H NMR (600 MHz, CDCl_3_): δ_H_ 7.75 (s, br, 1H, H-5_triaz._), 5.89 (d, 1H, *J* 9.0 Hz, H-1_Glc_), 5.46 (dd~t, 1H, *J_1_* 9.1 Hz, *J_2_* 9.5 Hz, H-3_Glc_), 5.42 (dd~t, 1H, *J_1_* 9.0 Hz, *J_2_* 9.5 Hz, H-2_Glc_), 5.25 (dd~t, 1H, *J_1_* 9.1 Hz, *J_2_* 10.2 Hz, H-4_Glc_), 4.67 (d, 1H, *J* 13.2 Hz, OCH_a_), 4.66 (s, br, 1H, H-29a), 4.60 (d, 1H, *J* 12.6 Hz, OCH_b_), 4.55 (dd, 1H, *J_1_* 1.2 Hz, *J_2_* 2.4 Hz, H-29b), 4.30 (dd, 1H, *J_1_* 5.0 Hz, *J_2_* 12.6 Hz, H-6a_Glc_), 4.14 (dd, 1H, *J_1_* 2.1 Hz, *J_2_* 12.6 Hz, H-6b_Glc_), 4.00 (ddd, 1H, *J_1_* 2.1 Hz, *J_2_* 5.0 Hz, *J_3_* 10.2 Hz, H-5_Glc_), 3.63 (d, 1H, *J* 8.4 Hz, H-28a), 3.19–3.17 (m, 2H, H-28a, H-3), 2.38 (td, 1H, *J_1_* 5.8 Hz, *J_2_* 11.1 Hz, H-19), 2.10–0.60 (m, 25H, CH, CH_2_), 2.08, 2.07, 2.03, 1.88 (all s, 3H each, 4x CH_3_CO), 1.66 (s, 3H, H-30), 1.02, 0.97, 0.96, 0.83, 0.76 (all s, 3H each, H-23–H-27) ppm; ^13^C NMR (150 MHz, CDCl_3_) δ_C_ 170.45, 169.90, 169.32, 168.87, 150.57, 146.62, 120.58, 109.60, 85.73, 78.95, 75.12, 72.73, 70.32, 68.94, 67.72, 64.95, 61.58, 55.31, 50.42, 48.85, 47.88, 47.26, 42.68, 40.92, 38.88, 38.73, 37.51, 37.17, 34.77, 34.26, 29.91, 29.85, 28.00, 27.42, 25.22, 20.85, 20.66, 20.53, 20.51, 20.20, 19.06, 18.32, 16.11, 16.03, 15.37, 14.79 ppm (Supplementary Materials, Table S2); IR (ATR) ν: 3400–3500, 2941, 1751, 1650, 1455, 1367, 1216, 1038, 731 cm^−1^.

*Product **15b** (28-OCH_2_TriazGalBN****)*** was obtained as a white solid (201.7 mg, 97% yield); m.p.: 117–118 °C; [α]^25^_D_ = −9.4 (c 0.5, CHCl_3_); HRMS (ESI^+^): calcd for C_47_H_71_N_3_O_11_Na ([M + Na]^+^): *m/z* 876.4986; found: *m/z* 876.4989; ^1^H NMR (600 MHz, CDCl_3_): δ_H_ 7.80 (s, br, 1H, H-5_triaz._), 5.84 (d, 1H, *J* 9.3 Hz, H-1_Gal_), 5.58 (dd, 1H, *J_1_* 9.3 Hz, *J_2_* 10.2 Hz, H-2_Gal_), 5.55 (dd, 1H, *J_1_* 0.8 Hz, *J_2_* 3.4 Hz, H-4_Gal_), 5.25 (dd, 1H, *J_1_* 3.4 Hz, *J_2_* 10.2 Hz, H-3_Gal_), 4.69–4.57 (m, 4H, OCH_2_, H-29a, H-29b), 4.24–4.18 (m, 2H, H-6a_Ga__l_, H-5_Gal_), 4.13 (d, 1H, *J_1_* 6.3 Hz, *J_2_* 10.9 Hz, H-6b_Gal_), 3.63 (d, 1H, *J* 8.2 Hz, H-28a), 3.21–3.17 (m, 2H, H-28a, H-3), 2.40 (td, 1H, *J_1_* 6.0 Hz, *J_2_* 11.1 Hz, H-19), 2.30–0.60 (m, 25H, CH, CH_2_), 2.22, 2.04, 2.01, 1.90 (all s, 3H each, 4x CH_3_CO), 1.66 (s, 3H, H-30), 1.02, 0.97, 0.96, 0.83, 0.76 (all s, 3H each, H-23–H-27). ppm; ^13^C NMR (150 MHz, CDCl_3_) δ_C_ 170.28, 169.95, 169.80, 169.04, 150.61, 146.46, 120.76, 109.60, 86.27, 78.96, 74.02, 70.89, 68.94, 67.85, 66.87, 64.91, 61.15, 55.30, 50.41, 48.84, 47.87, 47.26, 42.69, 40.93, 38.88, 38.72, 37.50, 37.17, 34.79, 34.25, 29.95, 29.86, 28.00, 27.42, 27.23, 25.22, 20.84, 20.66, 20.62, 20.49, 20.30, 19.06, 18.33, 16.11, 16.00, 15.37, 14.80 ppm (Supplementary Materials, Table S2); IR (ATR) ν: 3600–3200, 2930, 2868, 1749, 1650, 1456, 1370, 1214, 1042, 731 cm^−1^.

*Product **16a** (28-OCH_2_TriazCH_2_(CO)NHGlcBN)* was obtained as a white solid (190.0 mg, 99% yield); m.p.: 127-129 °C; [α]^25^_D_ = +11.4 (c 0.5, CHCl_3_); HRMS (ESI^+^): calcd for C_49_H_74_N_4_O_12_Na ([M + Na]^+^): *m/z* 933.5201; found: *m/z* 933.5202; ^1^H NMR (600 MHz, CDCl_3_): δ_H_ 7.66 (s, br, 1H, H-5_triaz._), 6.99 (d, 1H, *J* 8.8 Hz, NH), 5.29 (dd~t, 1H, *J* 9.5 Hz, H-1_Glc_), 5.21 (dd, 1H, *J_1_* 8.9 Hz, *J_2_* 9.4 Hz, H-3_Glc_**^a^**), 5.12 (d, 1H, *J* 16.6 Hz, OCH_a_**^b^**), 5.05 (dd, 1H, *J_1_* 9.4 Hz, *J_2_* 10.1 Hz, H-4_Glc_**^a^**), 5.04 (d, 1H, *J* 16.5 Hz, OCH_b_**^b^**), 4.90 (dd~t, 1H, *J* 9.6 Hz, H-2_Glc_**^a^**), 4.67 (s, br, 3H, H-29a, CH_2_CO**^b^**), 4.57 (dd, 1H, *J_1_* 1.4 Hz, *J_2_* 2.1 Hz, H-29b), 4.28 (dd, 1H, *J_1_* 4.4 Hz, *J_2_* 12.5 Hz, H-6a_G__lc_), 4.09 (dd, 1H, *J_1_* 2.2 Hz, *J_2_* 12.5 Hz, H-6b_Glc_), 3.81 (ddd, 1H, *J_1_* 2.2 Hz, *J_2_* 4.4 Hz, *J_3_* 10.1 Hz, H-5_Glc_), 3.63 (d, 1H, *J* 9.1 Hz, H-28a), 3.22-2.18 (m, 2H, H-28b, H-3), 2.39 (td, 1H, *J_1_* 5.8 Hz, *J_2_* 10.8 Hz, H-19), 2.15–0.60 (m, 25H, CH, CH_2_), 2.08, 2.03, 2.02, 2.01 (all s, 3H each, 4x CH_3_CO), 1.67 (s, 3H, H-30), 0.97, 0.96, 0.956, 0.82, 0.77 (all s, 3H each, H-23–H-27) ppm; ^13^C NMR (150 MHz, CDCl_3_) δ_C_ 170.86, 170.57, 169.84, 169.49, 165.68, 150.53, 146.71, 123.76, 109.64, 78.99, 78.41, 73.80, 72.51, 70.32, 68.95, 68.01, 65.14, 61.56, 55.28, 52.59, 50.38, 48.83, 47.88, 47.23, 42.64, 40.88, 38.86 38.70, 37.49, 37.15, 34.73, 34.23, 29.86, 29.69, 28.01, 27.40, 27.16, 25.18, 20.83, 20.73, 20.56, 20.54, 19.08, 18.33, 16.12, 15.93, 15.41, 14.79 ppm (Supplementary Materials, Table S2); IR (ATR) ν: 3200–3400, 2937, 1749, 1650, 1450, 1367, 1223, 1037, 993, 907, 882, 753 cm^−1^. **^a,b^** Reverse signal assignment is possible.

*Product **16b** (28-OCH_2_TriazCH_2_(CO)NHGalBN)* was obtained as a white solid (184.0 mg, 97% yield); m.p.: 117-120 °C; [α]^25^_D_ = +14.3 (c 0.5, CHCl_3_); HRMS (ESI^+^): calcd for C_49_H_74_N_4_O_12_Na ([M + Na]^+^): *m/z* 933.5201; found: *m/z* 933.5202; ^1^H NMR (600 MHz, CDCl_3_): δ_H_ 7.66 (s, br, 1H, H-5_triaz._), 6.93 (d, 1H, *J* 8.9 Hz, NH), 5.43 (dd, 1H, *J_1_* 0.9 Hz, *J_2_* 3.3 Hz, H-4_Gal_), 5.19 (dd, 1H, *J_1_* 8.9 Hz, *J_2_* 9.2 Hz, H-1_Gal_), 5.12 and 4.99 (qAB, 2H, *J* 3.3 Hz, CH_2_CO), 5.11 (dd, 1H, *J_1_* 3.3 Hz, *J_2_* 10.3 Hz, H-3_Gal_), 5.06 (dd, 1H, *J_1_* 9.2 Hz, *J_2_* 10.3 Hz, H-2_Gal_), 4.68 (s, 2H, OCH_2_), 4.67 and 4.57 (s, br, 1H each, H-29a, H-29b), 4.11 (dd, 1H, *J_1_* 6.9 Hz, *J_2_* 11.3 Hz, H-6a_Gal_), 4.07 (dd, 1H, *J_1_* 6.2 Hz, *J_2_* 11.23 Hz, H-6b_Gal_), 4.02 (m, 1H, H-5_Gal_), 3.63 (d, 1H, *J* 8.9 Hz, H-28a), 3.22–3.18 (m, 2H, H-28b, H-3), 2.39 (td, 1H, *J_1_* 5.9 Hz, *J_2_* 10.8 Hz, H-19), 2.30–0.60 (m, 25H, CH, CH_2_), 2.15, 2.04, 2.03, 1.98 (all s, 3H each, 4x CH_3_CO), 1.67 (s, 3H, H-30), 0.97, 0.96, 0.957, 0.82, 0.77 (all s, 3H each, H-23–H-27) ppm; ^13^C NMR (150 MHz, CDCl_3_) δ_C_ 171.00, 170.37, 169.98, 169.73, 165.67, 150.53, 146.60, 123.86, 109.63, 78.98, 78.61, 72.56, 70.71, 68.98, 68.06, 67.08, 65.13, 61.08, 55.29, 52.55, 50.39, 48.84, 47.89, 47.24, 42.65, 40.89, 38.87, 38.71, 37.50, 37.16, 34.74, 34.24, 29.88, 29.86, 28.01, 27.39, 27.17, 25.19, 20.84, 20.67, 20.64, 20.60, 20.50, 19.08, 18.33, 16.12, 15.94, 15.40, 14.79 ppm (Supplementary Materials, Table S2); IR (ATR) ν: 3100–2800, 1749, 1650, 1368, 1222, 1084, 1046, 752 cm^−1^. 

#### 3.2.3. General Procedure for the Synthesis of Betulin Glycoconjugates **17**–**18** according to Strategy I (Modification C3 and C28)

To a well-stirred 3,28-*O,O’*-di(2-propargyl)betulin **3** (0.193 mmol, 100.0 mg) and per-*O*-acetylated glucopyranosyl or galactopyranosyl azides **12a**–**b** (0.385 mmol, 143.9 mg) or **14a**–**b** (0.385 mmol, 165.9 mg) in *i*-PrOH (6.6 mL), CuSO_4_·5H_2_O (0.231 mmol, 57.8 mg) dissolved in H_2_O (3.3 mL) and sodium ascorbate (0.424 mmol, 84.0 mg) dissolved in H_2_O (3.3 mL) were added successively. The reaction mixture was stirred under argon atmosphere for 24 h at room temperature. After completion of the reaction (was monitored by TLC), the reaction mixture was extracted with (**17a**–**b**: DCM 5 × 5 mL; **18b**: AcOEt 5 × 5 mL), then the mixture was dried over MgSO_4_ and was concentrated in vacuo. The crude residue was separated/purified using column chromatography (**17a**–**b**: DCM:AcOEt, gradient: 15:1 to 1:1; **18b**: DCM:MeOH, gradient: 40:1 to 10:1).

*Product **17a**(3,28-di(OCH_2_TriazGlc)BN)* was obtained as a white solid (242.0 mg, 99% yield); m.p.: 126–127 °C; [α]^25^_D_ = −6.1 (c 0.5, CHCl_3_); HRMS (ESI^+^): calcd for C_64_H_93_N_6_O_20_ ([M + H]^+^): *m/z* 1265.6445; found: *m/z* 1265.6426; ^1^H NMR (600 MHz, CDCl_3_): δ_H_ 7.75 and 7.74 (all s, 1H each, 2x H-5_triaz._), 5.90 and 5.89 (all d, 1H each, *J_1_* 9.0 Hz and *J_2_* 9.4 Hz, 2x H-1_Glc_), 5.50-5.40 (m, 4H, 2x H-2_Glc_, 2x H-3_Glc_)), 5.24 and 5.25 19 (all dd, 1H each, *J_1_* 9.4 Hz, *J_2_* 10.1 Hz and *J_1_* 9.2 Hz, *J_2_* 10.1 Hz, 2x H-4_Glc_), 4.78–4.54 (m, 3H, OCH_2_, H-29b), 4.32–4.28 (m, 2H, 2x H-6a_Glc_), 4.36-4.13 (m, 2H, 2x H-6b_Glc_), 4.03-4.00 (m, 2H, 2x H-5_Glc_), 3.62 (d, 1H, *J* 8.4 Hz, H-28a), 3.18 (d, 1H, *J* 9.0 Hz, H-28b), 2.95 (dd, 1H, *J_1_* 4.2 Hz, *J_2_* 12.0 Hz, H-3), 2.38 (td, 1H, *J_1_* 5.7 Hz, *J_2_* 11.0 Hz, H-19), 2.20-0.60 (m, 24H, CH, CH_2_), 2.08, 2.07, 2.069, 2.031, 2.029, 1.88, 1.87 (all s, 3H each, 8x CH_3_CO), 1.66 (s, 3H, H-30), 1.01, 0.95, 0.89, 0.84, 0.78 (all s, 3H each, H-23–H-27) ppm; ^13^C NMR (150 MHz, CDCl_3_) δ_C_ 170.46, 170.44, 169.91, 169.89, 169.32, 168.86, 168.79, 150.57, 147.26, 146.59, 120.58, 120.50, 109.61, 86.65, 85.69, 85.62, 75.08 75.04, 72.81 72.71, 70.31 70.18, 68.92, 67.71 67.70, 64.93, 62.88, 61.57, 55.68, 50.36, 48.82, 47.84, 47.25, 42.65, 40.94, 38.81, 38.52, 37.48, 37.11, 34.76, 34.24, 29.89, 29.83, 27.97, 27.18, 25.20, 22.83, 20.85, 20.66, 20.53, 20.51, 20.17, 19.05, 18.24, 16.25, 16.12, 16.02, 14.73 ppm (Supplementary Materials, Table S2); IR (ATR) ν: 2942, 1750, 1457, 1368, 1216, 1091, 1055, 1036, 913, 732 cm^−1^.

*Product **17b**(3,28-di(OCH_2_TriazGal)BN)* was obtained as a white solid (242.0 mg, 99% yield); m.p.: 122-123 °C; [α]^25^_D_ = +4.9 (c 0.5, CHCl_3_); HRMS (ESI ^+^ ): calcd for C_64_H_92_N_6_O_20_Na ([M + Na]^+^): *m/z* 1287.6264; found: *m/z* 1287.6206; ^1^H NMR (600 MHz, CDCl_3_): δ_H_ 7.80 and 7.79 (all s, br, 1H each, 2x H-5_triaz._), 5.86 and 5.856 (all d, 1H each, *J* 9.4 Hz, 2x H-1_Gal_), 5.61 and 5.58 (all dd, 1H each, *J_1_* 9.4 Hz, *J_2_* 10.2 Hz, 2x H-2_Gal_), 5.56–5.54 (m, 2H, 2x H-4_Gal_), 5.25 and 5.26 (all dd, 1H each, *J_1_* 5.4 Hz, *J_2_* 10.2 Hz, 2x H-3_Gal_), 4.78–4.56 (m, 6H, 2x OCH_2_, H-29a, H-29b), 4.26–4.23 (m, 2H, 2x H-5_Gal_), 4.22–4.10 (m, 4H, 2x H-6a_Gal_, 2x H-6b_Gal_), 3.62 (d, 1H, *J* 8.8 Hz, H-28a), 3.20 (d, 1H, *J* 9.1 Hz, H-28b), 2.97 (dd, 1H, *J_1_* 4.3 Hz, *J_2_* 11.7 Hz, H-3), 2.40 (td, 1H, *J_1_* 5.7 Hz, *J_2_* 11.1 Hz, H-19), 2.23, 2.22, 2.044, 2.04, 2.013, 2.01, 1.90, 1.88 (all s, 3H each, 8x CH_3_CO), 2.30–0.60 (m, 24H, CH, CH_2_), 1.67 (s, 3H, H-30), 1.01, 0.95, 0.90, 0.84, 0.78 (all s, 3H each, H-23–H-27) ppm; ^13^C NMR (150 MHz, CDCl_3_) δ_C_ 170.31, 170.28, 169.95, 169.79, 169.03, 168.93, 150.59, 147.06, 146.42, 120.77, 120.66, 109.62, 86.63, 86.22, 86.17, 73.98, 70.94, 70.86, 68.91, 67.86, 67.74, 66.91, 64.88, 61.18, 55.68, 52.88, 50.36, 48.83, 47.86, 47.25, 42.66, 40.95, 38.82, 38.53, 37.48, 37.12, 34.78, 34.24, 29.94, 29.84, 27.96, 27.20, 22.87, 25.20, 20.86, 20.66, 20.63, 20.49, 20.29, 20.27, 19.05, 18.26, 16.26, 16.13, 16.00, 14.74 ppm (Supplementary Materials, Table S2); IR (ATR) ν: 2942, 1748, 1457, 1368, 1213, 1089, 1043, 921, 730 cm^−1^.

*Product **18b**(3,28-di(OCH_2_TriazCH_2_(CO)NHGal)BN)* was obtained as a white solid (216.9 mg, 82% yield); m.p.: 142–145 °C; [α]^25^_D_ = +4.9 (c 0.5, CHCl_3_); HRMS (ESI^+^): calcd for C_68_H_99_N_8_O_22_ ([M + H]^+^): *m/z* 1379.6900; found: *m/z* 1379.6533; ^1^H NMR (600 MHz, CDCl_3_): δ_H_ 7.66 and 7.64 (all s, 1H each, 2x H-5_triaz._), 6.86 and 6.85 (all d, 1H each, *J* 8.8 Hz, 2x NH), 5.45-5.40 (m, 2H, 2x H-4_Gal_), 5.22-5.18 (m, 2H, 2x H-1_Gal_), 5.14–4.98 (m, 8H, 2x H-2_Gal_, 2x H-3_Gal_, 2x CH_2_), 4.84–4.57 (m, 6H, H-29a, H-29b, 2x CH_2_), 4.14-4.07 (m, 4H, 2x H-6a_Ga__l_, 2x H-6b_Ga__l_), 4.06–4.01 (m, 2H, 2x H-5_Gal_), 3.63 (d, 1H, *J* 8.9 Hz, H-28a), 3.22 (d, 1H, *J* 9.0 Hz, H-28b), 2.97 (dd, 1H, *J_1_* 4.2 Hz, *J_2_* 11.7 Hz, H-3), 2.39 (td, 1H, *J_1_* 5.9 Hz, *J_2_* 10.6 Hz, H-19), 2.30–0.60 (m, 24H, CH, CH_2_), 2.15, 2.14, 2.04, 2.03, 2.02, 1.98, 1.977, 1.96 (all s, 3H each, 8x CH_3_CO), 1.67 (s, 3H, H-30), 0.96, 0.95, 0.92, 0.83, 0.77 (all s, 3H each, H-23–H-27) ppm; ^13^C NMR (150 MHz, CDCl_3_) δ_C_ 171.03, 170.92, 170.34, 169.98, 169.71, 165.68, 165.55, 150.54, 147.49, 146.77, 123.75, 123.61, 109.65, 86.89, 78.75, 78.72, 72.59, 72.57, 70.67, 70.64, 69.01, 68.06, 68.00, 67.06, 65.21, 63.20, 61.11, 61.00, 55.69, 52.71, 52.65, 50.36, 48.85, 47.90, 47.25, 42.65, 40.93, 38.85, 38.52, 37.50, 37.14, 34.75, 34.24, 29.88, 29.69, 28.10, 27.17, 25.20, 22.86, 20.87, 20.67, 20.62, 20.60, 20.59, 20.50, 19.09, 18.26, 16.30, 16.15, 15.96, 14.76, ppm (Supplementary Materials, Table S2); IR (ATR) ν: 2950, 1748, 1620, 1510, 1450, 1368, 1218, 1084, 1045, 970, 890, 720 cm^−1^.

#### 3.2.4. Procedure for the Synthesis of Betulin Glycoconjugates **19a** according to Strategy II (Modification C28)

To a well-stirred 28-*O*-(2-azidoacetyl)betulin **6** (0.190 mmol, 100.0 mg) and *per*-*O*-acetylated glucopyranosyl propargyl **10a** (0.190 mmol, 73.5 mg) in *i*-PrOH (2 mL) and THF (1 mL), CuSO_4_·5H_2_O (0.114 mmol, 22.6 mg) dissolved in H_2_O (1 mL) and sodium ascorbate (0.209 mmol, 52.2 mg) dissolved in H_2_O (1 mL) were added successively. The reaction mixture was stirred under argon atmosphere for 24 h at room temperature. After completion of the reaction (was monitored by TLC), the reaction mixture was extracted with DCM (5 × 5 mL), then the mixture was dried over MgSO_4_ and was concentrated in vacuo. The crude residue was separated/purified using column chromatography (DCM:MeOH, gradient: 50:1 to 10:1).

*Product **19a** (28-O(CO)CH_2_TriazCH_2_OGlcBN)* was obtained as a white solid (168.3 mg, 97% yield); m.p.: 99–101 °C; [α]^25^_D_ = −15.6 (c 0.5, CHCl_3_); HRMS (ESI+): calcd for C_49_H_73_N_3_O_13_Na ([M + Na]^+^): m/z 934.5041; found: m/z 934.5045; ^1^H NMR (600 MHz, CDCl_3_): δ_H_ 7.69 (s, br, 1H, H-5_triaz_.), 5.22-5.17 (m, 2H, H-1_Glc_, H-3_Glc_), 5.10 (dd~t, 1H, *J_1_* 9.6 Hz, *J_2_* 9.8 Hz, H-2_Glc_), 5.02 (ddt, 1H, *J_1_* 8.0 Hz, *J_2_* 9.5 Hz, H-4Glc), 4.97 and 4.86 (qAB, 2H, CH_2_), 4.68 (s, br, 2H, CH_2_), 4.68 and 4.60 (all s, br, 1H each, H-29a, H-29b), 4.43 (d, 1H, *J* 10.9 Hz, H-28a), 4.26 (dd, 1H, *J_1_* 4.7 Hz, *J_2_* 12.3 Hz,H-6a_Glc_), 4.17 (dd, 1H, *J_1_* 2.1 Hz, *J_2_* 12.3 Hz, H-6b_Glc_), 3.97 (d, 1H, *J* 11.0 Hz, H-28b), 3.73 (ddd, 1H, *J_1_* 2.1 Hz, *J_2_* 4.7 Hz, *J_3_* 9.5 Hz, H-5_Glc_), 3.18 (dd, 1H, *J_1_* 4.7 Hz, *J_2_* 11.5 Hz, H-3), 2.41 (td, 1H, *J_1_* 5.7 Hz, *J_2_* 10.7 Hz, H-19), 2.20-0.60 (m, 25H, CH, CH_2_), 2.10, 2.03, 2.004, 2.00 (all s, 3H each, 4x CH_3_CO), 1.68 (s, 3H, H-30), 1.01, 0.98, 0.97, 0.82, 0.76 (all s, 3H each, H-23–H-27) ppm; ^13^C NMR (150 MHz, CDCl_3_) δ_C_ 170.65, 170.18, 169.45, 169.42, 166.55, 149.73, 124.28, 110.10, 78.94, 72.79, 71.94, 71.21, 68.36, 61.80, 50.86, 64.96, 62.93, 55.29, 50.85, 50.33, 48.80, 47.64, 46.45, 42.71, 40.88, 38.87, 38.71, 37.68, 37.15, 34.39, 34.18, 29.60, 29.44, 27.99, 27.01, 27.39, 25.16, 20.78, 20.74, 20.67, 20.60, 19.11, 18.28, 16.10, 16.01, 15.36, 14.78 ppm (Supplementary Materials, Table S2); IR (ATR) ν: 3400–3200, 2942, 1747, 1640, 1456, 1366, 1218, 1037, 982, 883, 769 cm^−1^.

#### 3.2.5. Procedure for the Synthesis of Betulin Glycoconjugates **20a** according to Strategy II (Modification C3 and C28)

To a well-stirred 3,28-*O,O*’-di(2-azidoacetyl)betulin **7** (0.164 mmol, 100.0 mg) and per-*O*-acetylated glucopyranosyl propargyl **10a** (0.329 mmol, 126.9 mg) in *i*-PrOH (4.2 mL) and THF (1.8 mL), CuSO_4_·5H_2_O (0.181 mmol, 45.1 mg) dissolved in H_2_O (2.1 mL) and sodium ascorbate (0.099 mmol, 19.5 mg) dissolved in H_2_O (2.1 mL) were added successively. The reaction mixture was stirred under argon atmosphere for 24 h at room temperature. After completion of the reaction (was monitored by TLC), the reaction mixture was extracted with DCM (5 × 5 mL), then the mixture was dried over MgSO4 and was concentrated in vacuo. The crude residue was separated/purified using column chromatography (DCM:MeOH, gradient: 30:1 to 10:1).

*Product **20a** (3,28-di(O(CO)CH_2_TriazCH_2_OGlc)BN)* was obtained as a white solid (198.0 mg, 87% yield); m.p.: 109–112 °C; [α]^25^_D_ = −19.0 (c 0.5, CHCl_3_); HRMS (ESI+): calcd for C_68_H_97_N_6_O_24_ ([M + H]^+^): m/z 1381.6554; found: m/z 1381.6218; ^1^H NMR (600 MHz, CDCl_3_): δ_H_ 7.68 and 7.66 (all s, 1H each, 2x H-5_triaz_.), 5.23-5.13 (m, 4H, 2x (H-1_Glc_ H-3_Glc_)), 5.12–5.06 (m, 2H, 2x H-2_Glc_), 5.05-4.99 (m, 2H, 2x H-4_Glc_), 4.99-4.83 (m, 4H, 2x CH_2_), 4.69–4.66 (m, 5H, H-29a, 2x CH_2_), 4.60 (s, br, 1H, H-29b), 4.43 (d, 1H, J 10.6 Hz, H-28a), 4.26 and 4.27 (all dd, 1H each, *J_1_* 1.0 Hz, *J_2_* 12.4 Hz, 2x H-6a_Glc_), 4.19–4.14 (m, 2H, 2x H-6b_Glc_), 3.96 (d, 1H, *J* 11.0 Hz, H-28b), 3.75–3.70 (m, 2H, 2x H-5_Glc_), 4.56 (dd, 1H, *J_1_* 5.2 Hz, *J_2_* 10.1 Hz, H-3), 2.41 (td, 1H, *J_1_* 5.7 Hz, *J_2_* 10.7 Hz, H-19), 2.20–0.60 (m, 24H, CH, CH_2_), 2.093, 2.09, 2.028, 2.025, 2.03, 2.00, 1.998, 1.994 (all s, 3H each, 8x CH_3_CO), 1.68 (s, 3H, H-30), 1.01, 0.98, 0.97, 0.84, 0.77 (all s, 3H each, H-23–H-27) ppm; ^13^C NMR (150 MHz, CDCl_3_) δ_C_ 170.64, 170.16, 169.43, 166.56, 165.90, 160.42, 149.68, 144.65, 144.55, 124.25, 124.24, 110.16, 83.86, 68.37, 72.80, 71.94, 71.22, 68.37, 62.94, 61.81, 61.80, 55.30, 51.05, 50.83, 50.21, 48.77, 47.65, 46.45, 42.73, 40.89, 38.27, 37.90, 37.65, 37.03, 34.38, 34.05, 29.57, 29.43, 28.01, 27.00, 25.08, 23.61, 21.96, 20.77, 20.66, 20.65, 20.60, 19.10, 18.11, 16.39, 16.12, 16.00, 14.75 ppm (Supplementary Materials, Table S2); IR (ATR) ν: 2930, 1744, 1620, 1367, 1216, 1037, 979, 880, 752 cm^−1^. 

#### 3.2.6. Procedure for the Synthesis of 3,28-*O,O’*-di(2-(4-(hydroxymethyl-1H-1,2,3-triazol-1-yl)acetyl)betulin **21**

To a well-stirred 3,28-*O,O’*-di(2-azidoacetyl)betulin **7** (0.663 mmol, 403.6 mg) in *i*-PrOH (7.6 mL) and THF (4.8 mL), propargyl alcohol (1.989 mmol, 111.5 mg, 0.116 mL) and CuSO_4_·5H_2_O (0.796 mmol, 198.6 mg) dissolved in H_2_O (2.7 mL) and sodium ascorbate (1.458 mmol, 288.9 mg) dissolved in H_2_O (2.7 mL) were added successively. The reaction mixture was stirred under argon atmosphere for 24 h at room temperature. After completion of the reaction (was monitored by TLC), the reaction mixture was extracted with DCM (9 × 30 mL), then mixture was dried over MgSO_4_ and was concentrated in vacuo. The crude residue was separated/purified using column chromatography (DCM:MeOH, gradient: 20:1 to 5:1).

*Product **21** (3,28-di(O(CO)CH_2_TriazCH_2_OH)BN)* was obtained as a white solid (367.2 mg, 77% yield); m.p.: 105–108 °C; [α]^25^_D_ = +4.7 (c 0.5, CHCl_3_); HRMS (ESI^+^): calcd for C_40_H_61_N_6_O_6_ ([M + H]^+^): *m/z* 721.4653; found: *m/z* 721.4650; ^1^H NMR (600 MHz, CDCl_3_): δ_H_ 7.68 and 7.66 (all s, 1H each, 2x H-5_triaz._), 5.19 (d, 2H, *J* 1.0 Hz, O(CO)CH_2_), 5.15 (d, 2H, *J* 0.6 Hz, O(CO)CH_2_), 4.82 (br s, 4H, 2x CH_2_OH), 4.69 (d, 1H, *J* 1.6 Hz, H-29a), 4.60 (s, 1H, H-29b), 4.57–4.54 (m, 1H, H-3),4.42 (d, 1H, *J* 10.9 Hz, H-28a), 3.96 (d, 1H, *J* 10.9 Hz, H-28b), 2.41 (m, 3H, H-19, 2x CH_2_OH), 2.10–0.60 (m, 24H, CH, CH_2_), 1.67 (s, 3H, H-30), 1.00, 0.96, 0.83, 0.828, 0.75 (all s, 3H each, H-23–H-27) ppm; ^13^C NMR (150 MHz, CDCl_3_) δ_C_ 165.97, 165.61, 149.96, 149.66, 148.09, 123.08 110.12, 83.85, 64.92, 56.50; 55.28, 55.23, 51.08; 50.86, 50.20, 48.75, 47.65, 46.43, 42.71, 40.88, 38.26, 37.88, 37.64, 37.02, 34.37, 34.04, 29.56, 29.43, 28.00, 26.98, 25.07, 23.58, 20.73, 19.09, 18.10, 16.36, 16.11, 16.00, 14.75 ppm (Supplementary Materials, Table S3); IR (ATR) ν: 3500–300, 2927, 2871, 1743, 1456, 1376, 1266, 1219, 1040, 1006, 977, 882, 805 cm^−1^.

#### 3.2.7. Procedure for the Deprotection of Betulin Glycoconjugates **15**–**17**

Glycoconjugates **15**–**17** (1.0 mmol) were dissolved in MeOH (**15a**–**b**: 16 mL, **16a**–**b**: 35 mL, **17a**–**b**: 30 mL). Then, 1M solution of MeONa in MeOH (**15a**–**b**: 0.2 mmol, 0.2 mL, **16a**–**b**: 0.4 mmol, 0.4 mL, **17a**–**b**: 0.15 mmol, 0.15 mL) was added. Reaction was carried out for 120 min at room temperature. The reaction progress was monitored on TLC. After the reaction was complete, the mixture was neutralized with *Amberlyst-15*, filtered, and the filtrate was evaporated in vacuo. The crude products were crystallized from methanol.

*Product****22a****(28-OCH_2_TriazGlcBN)* was obtained as a white solid (69.9 mg, 87% yield); m.p.: 174–175 °C; [α]^25^_D_ = +4.1 (c 0.5, MeOH); HRMS (ESI^+^): calcd for C_39_H_63_N_3_O_7_Na ([M + Na] ^+^ ): *m/z* 708.4564; found: *m/z* 708.4555; ^1^H NMR (400 MHz, MeOD): δ_H_ 8.19 (s, br, 1H, H-5_triaz._), 5.62 (d, 1H, *J* 9.2 Hz, H-1_Glc_), 4.68–4.55 (m, 4H, H-29a, H-29b, OCH_2_), 3.91–3.86 (m, 2H, H-6a_Glc_, H-5_Glc_), 3.73 (dd, 1H, *J_1_* 5.2 Hz, *J_2_* 12.1 Hz, H-6b_Glc_), 3.65 (d, 1H, *J* 8.9 Hz, H-28a), 3.61–3.49 (m, 3H, H-4_Glc_, H-3_Glc_, H-2_Glc_), 3.23 (d, 1H, *J* 9.0 Hz, H-28b), 3.12 (dd, 1H, *J_1_* 4.9 Hz, *J_2_* 11.2 Hz, H-3), 2.43 (td, 1H, *J_1_* 5.2 Hz, *J_2_* 10.9 Hz, H-19), 2.05-0.60 (m, 25H, CH, CH_2_), 1.67 (s, 3H, H-30), 1.00, 0.98, 0.95, 0.86, 0.76 (all s, 3H each, H-23–H-27) ppm; ^13^C NMR (150 MHz, MeOD) δ_C_ 151.86, 146.36, 124.41, 110.25, 89.70, 81.18, 79.75, 78.60, 74.10, 71.00, 69.88, 65.31, 62.47, 56.90, 51.94, 50.24, 43.82, 42.21, 40.14, 38.99, 38.35, 35.83, 35.53, 31.05, 30.75, 28.68, 28.39, 28.12, 26.71, 22.06, 19.52, 18.40, 16.76, 16.68, 16.15, 15.33 ppm (Supplementary Materials, Table S3); IR (ATR) ν: 3600-3100, 2941, 2871, 1620, 1450, 1390, 1100, 1043, 1013, 880 cm^−1^.

*Product****22b****(28-OCH_2_TriazGalBN)* was obtained as a white solid (79.5 mg, 99% yield); m.p.: 218–220 °C; [α]^25^_D_ = +9.1 (c 0.5, MeOH); HRMS (ESI ^+^ ): calcd for C_39_H_63_N_3_O_7_Na ([M + Na]^+^): *m/z* 708.4564; found: *m/z* 708.4553; ^1^H NMR (400 MHz, MeOD): δ_H_ 8.21 (s, br, 1H, H-5_triaz._), 5.57 (d, 1H, *J* 9.2 Hz, H-1_Glc_), 4.68-4.56 (m, 4H, OCH_2_, H-29a, H-29b), 4.15 (dd~t, 1H, *J* 9.3 Hz, H-2_Gal_), 4.00 (dd, 1H, *J_1_* 0.9 Hz, *J_2_* 3.3 Hz, H-4_Gal_), 3.85–3.69 (m, 3H, 2H-6_Ga__l_, H-5_Gal_), 3.70 (dd, 1H, *J_1_* 3.3 Hz, *J_2_* 9.5 Hz, H-3_Gal_), 3.64 (d, 1H, *J* 8.6 Hz, H-28a), 3.23 (d, 1H, *J* 8.8 Hz, H-28b), 3.12 (dd, 1H, *J_1_* 5.0 Hz, *J_2_* 11.1 Hz, H-3), 2.43 (td, 1H, *J_1_* 5.9 Hz, *J_2_* 10.9 Hz, H-19), 2.01-0.60 (m, 25H, CH, CH_2_), 1.67 (s, 3H, H-30), 0.99, 0.98, 0.95, 0.86, 0.76 (all s, 3H each, H-23–H-27) ppm; ^13^C NMR (150 MHz, MeOD) δ_C_ 151.87, 146.54, 123.87, 110.28, 90.28, 79.90, 79.71, 75.40, 71.50, 70.32, 69.67, 65.33, 62.30, 56.86, 51.90, 50.17, 43.79, 42.15, 39.99, 40.09, 38.95, 38.32, 35.82, 35.46, 30.99, 30.79, 28.65, 28.34, 28.09, 26.64, 22.02, 19.41, 19.50, 16.76, 16.62, 16.16, 15.28 ppm (Supplementary Materials, Table S3); IR (ATR) ν: 3600–3100, 2932, 2869, 1630, 1456, 1374, 1093, 1044, 880 cm^−1^.

*Product****23b****(28-OCH_2_TriazCH_2_(CO)NHGalBN)* was obtained as a white solid (78.3 mg, 96% yield); m.p.: 187-190 °C; [α]^25^_D_ = +6.9 (c 0.5, CHCl_3_); HRMS (ESI ^+^ ): calcd for C_41_H_67_N_4_O_8_ ([M + H]^+^): *m/z* 743.4959; found: *m/z* 743.4962; ^1^H NMR (400 MHz, MeOD): 7.96 (s, br, 1H, H-5_triaz._), 7.46 (s, 1H, NH), 5.22 (q, 2H, *J* 16.4 Hz, CH_2_N), 4.90 (d, 1H, *J* 8.8 Hz, H-1_Gal_), 4.68 and 4.57 (s, br, 1H, H-29a, H-29b), 4.65 (s, 2H, OCH_2_), 3.94 (d, 1H, *J* 2.8 Hz, H-4_Gal_), 3.83–3.54 (m, 5H, H-28a, H-2_Gal_, H-3_Gal_, H-5_Gal_, H-6a_Gal_, H-6b_Gal_), 3.39 (s, 2H, CH_2_CO), 3.24 (d, 1H, *J* 9.2 Hz, H-28b), 3.18–3.14 (m, 1H, H-3), 2.39 (td, 1H, *J_1_* 5.9 Hz, *J_2_* 10.8 Hz, H-19), 2.05–0.60 (m, 25H, CH, CH_2_), 1.68 (s, 3H, H-30), 0.99, 0.98, 0.96, 0.84, 0.76 (all s, 3H each, H-23–H-27) ppm; ^13^C NMR (150 MHz, MeOD) δ_C_ 168.57, 151.91, 146.42, 126.86, 110.24, 81.77, 79.75, 78.48, 75.82, 71.51, 70.49, 69.49, 65.15, 56.90, 51.92, 50.23, 43.80, 42.18, 40.12, 40.01, 38.98, 38.35, 35.86, 35.52, 31.04, 30.78, 28.67, 28.36, 28.12, 26.67, 22.05, 19.53, 19.44, 16.77, 16.59, 16.17, 15.30 ppm (Supplementary Materials, Table S3); IR (ATR) ν: 3600–3000, 2942, 1698, 1374, 1228, 1083, 1045, 879 cm^−1^.

*Product****24a****(3,28-di(OCH_2_TriazGlc)BN)* was obtained as a white solid (63.9 mg, 87% yield); m.p.: 176–178 °C; [α]^25^_D_ = +16.4 (c 0.5, MeOH); HRMS (ESI ^+^ ): calcd for C_48_H_76_N_6_O_12_Na ([M + Na]^+^): *m/z* 951.5419; found: *m/z* 951.5441; ^1^H NMR (600 MHz, MeOD): δ_H_ 8.18 i 8.14 (all s, br, 1H each, 2x H-5_triaz._), 5.61 and 5.59 (all d, 1H each, *J* 8.7 Hz, 2x H-1_Glc_), 4.74–4.50 (m, 6H, H-29a, H-29b, 2x OCH_2_), 3.90–3.87 (m, 4H, 2x H-6a_Glc_, 2x H-5_Glc_), 3.74–3.48 (m, 9H, H-28a, 2x H-2_Glc_, 2x H-3_Glc_, 2x H-4_Glc_, 2x H-6b_Glc_), 3.23 (d, 1H, *J* 9.0 Hz, H-28b), 2.98 (dd, 1H, *J_1_* 4.2 Hz, *J_2_* 11.4 Hz, H-3), 2.43 (td, 1H, *J_1_* 5.9 Hz, *J_2_* 11.0 Hz, H-19), 2.00–0.65 (m, 25H, CH, CH_2_), 1.68 (s, 3H, H-30), 0.99, 0.98, 0.92, 0.87, 0.75 (all s, 3H each, H-23–H-27) ppm; ^13^C NMR (150 MHz, MeOD) δ_C_ 151.89, 146.99, 146.42, 124.33, 124.18, 110.24, 88.07, 89.65, 81.20, 78.64, 74.11, 71.00, 69.82, 65.36, 63.50, 62.49, 57.23, 51.93, 50.25, 43.85, 42.25, 39.85, 39.96, 39.00, 38.37, 35.84, 35.52, 31.07, 30.76, 28.67, 28.41, 28.40, 26.71, 22.10, 19.43, 18.40, 16.82, 16.79, 16.70, 15.32 ppm (Supplementary Materials, Table S3); IR (ATR) ν: 3600–3100, 2936, 1620, 1460, 1374, 1093, 1042, 896 cm^−1^.

*Product****24b****(3,28-di(OCH_2_TriazGal)BN)* was obtained as a white solid (50.7 mg, 69% yield); m.p.: 178–179 °C; [α]^25^_D_ = +22.4 (c 0.5, MeOH); HRMS (ESI^+^): calcd for C_48_H_77_N_6_O_12_ ([M + H]^+^): *m/z* 929.5599; found: *m/z* 929.5621; ^1^H NMR (600 MHz, MeOD): δ_H_ 8.21 and 8.18 (all s, br, 1H each, 2x H-5_triaz._), 5.57 and 5.56 (all d, 1H each, *J* 9.0 Hz, 2x H-1_Gal_), 4.74-4.51 (m, 6H, 2x OCH_2_, H-29a, H-29b), 4.15 and 4.14 (all dd~t, 2H each, *J* 9.4 Hz, 2x H-2_Gal_), 3.99 (m, 2H, 2x H-4_Gal_), 3.85-3.69 (m, 8H, 2x H-3_Gal,_ 2x H-5_Gal_, 2x H-6a_Ga__l_, 2x H-6b_Ga__l_), 3.64 (d, 1H, *J* 9.4 Hz, H-28a), 3.23 (d, 1H, *J* 9.0 Hz, H-28b), 2.98 (dd, 1H, *J_1_* 4.3 Hz, *J_2_* 11.8 Hz, H-3), 2.43 (td, 1H, *J_1_* 5.9 Hz, *J_2_* 10.7 Hz, H-19), 2.00–0.70 (m, 25H, CH, CH_2_), 1.68 (s, 3H, H-30), 0.99, 0.98, 0.92, 0.87, 0.76 (all s, 3H each, H-23–H-27)ppm; ^13^C NMR (150 MHz, MeOD) δ_C_ 151.89, 147.19, 146.56, 123.89, 123.64, 110.27, 90.27, 88.04, 80.04, 79.02, 75.44, 71.56, 71.55, 69.77, 65.38; 63.56, 62.50, 62.34, 57.22, 51.91, 50.22, 43.83, 42.22, 39.83, 39.97, 39.96, 38.34, 35.84, 35.48, 31.05, 30.78, 28.66, 28.37, 28.42, 26.68, 22.08, 19.42, 18.41, 16.83, 16.80, 16.66, 15.30 ppm (Supplementary Materials, Table S3); IR (ATR) ν: 3600–3100, 2925, 2869, 1650, 1456, 1374, 1219, 1090, 1045, 879 cm^−1^.

### 3.3. Biological Assays 

#### 3.3.1. Cell Lines

HCT 116 and MCF-7 cells were purchased from the American Type Culture Collection. NHDF cells were purchased from Lonza. All cells were cultured under standard conditions at 37 °C in a humidified atmosphere at 5% CO_2_ in DMEM/F12 medium (PAA) supplemented with 10% FBS (EURx).

#### 3.3.2. CCK-8 Assay

A Cell Counting Kit-8 (CCK-8) from Bimake was used to assess cell viability. Briefly, cells were seeded in 96-well plates, with three duplicate wells in each group. Cells were treated with betulin or its derivatives and incubated for 24 or 48 h. Then, CCK-8 solution was added to each well and the plate was incubated for 2 h at 37 °C. Subsequently, the absorbance at 450 nm was measured using a microplate reader (Epoch, BioTek). Cell viability rate was calculated using *CalcuSyn software* (version 2.0, Biosoft, Cambridge, UK).

## 4. Conclusions

In conclusion, we designed and synthesized a library of novel glycoconjugates of natural pentacyclic triterpenoid (BN) by employing click chemistry. The CuAAC reactions are extensively employed in the conjugate synthesis of various organic compounds, owing to their versatility, high chemoselectivity, and mild conditions. In this study, we successfully prepared new mono- and disubstituted betulin derivatives containing a sugar unit attached via a linker inclusive of the 1,2,3-triazole ring at the С3 and C28 position of the parent skeleton of BN. We confirmed that the click chemistry approach in a simple, easy and inexpensive way leads to a wide range of products with high yield, purity, and selectivity.

The methodology developed by us extends the synthetic possibilities of modifying the betulin skeleton. Importantly, it enables the simple preparation of both mono-(modification C28) and disubstituted betulin derivatives (modification C3 and C28) with high yields. The construction of the linker connecting betulin to the sugar unit depends on the type of building blocks used. According to Strategy I, synthesis starts with the preparation of an alkyne betulin derivative, which is then clicked with a 1-azido sugar. The second option is to modify the betulin with an azide moiety and click it with propargyl *O*-glucoside.

Unfortunately, a preliminary cytotoxicity assay of the obtained betulin glycoconjugates showed that the addition of a sugar unit to the native betulin structure via a few selected linkers containing a 1,2,3-triazole ring is not a significant way for biological activity. Monosubstituted betulin glycoconjugates show comparable or slightly lower activity than that of betulin alone while betulin derivatives containing two sugar units protected with acetyl groups increase cell viability, which may result from the release of glucose from the tested glycoconjugates under physiological conditions. In turn, the observed lack of biological activity of deprotected betulin glycoconjugates may be due to the insufficient affinity of these compounds for glucose transporters.

3,28-*O,O’*-Di(2-(4-(hydroxymethyl-*1H*-1,2,3-triazol-1-yl)acetyl)betulin **21**, modified only by introducing a fragment containing the 1,2,3-triazole system without the sugar unit, turned out to be surprisingly active. Unfortunately, it also turned out to be very toxic. This indicates that modifying the betulin backbone by introducing a 1,2,3-triazole ring significantly improves its cytotoxicity. However, further work should be done on increasing the selectivity of the obtained connections.

The obtained results show that despite the fact that the obtained betulin glycoconjugates do not show interesting antitumor activity, the idea of adding a sugar unit to the betulin backbone may, after some modifications, turn out to be correct and allow for the targeted transport of biologically active compounds into the tumor cells. The position used in the sugar unit for conjugation with the betulin derivative appears to be crucial for maintaining its affinity for GLUT transporters. Based on literature reports [59,60], it can be assumed that the 6-OH group (the group least involved in binding to the GLUT transporter) is the most neutral position that can be modified in sugar, so as not to reduce its affinity to the transport protein on the surface of cancer cells. Due to the above, it seems advisable to undertake further research on the chemical modifications of betulin by attaching sugar through the functionalization of this position. Glycoconjugation of betulin with the use of sugar derivatives modified in the C-6 position will be carried out both with the use of 1,3-dipolar azide-alkyne cycloaddition, as well as by creating bonds that can be broken under the action of intracellular enzymes (ester, amide, or carbamate).

## Figures and Tables

**Table 1:** Synthesis of betulin derivatives **2**–**7**.

Entry	Substrate				Product			
	No.	Solvent	Temp., °C	Time, h	No.	R (C3)	R^1^ (C28)	Yield, %
1	1	THF	r.t.	24	2	OH	OCH2C≡CH	54
					3	OCH2C≡CH	OCH2C≡CH	31
2	1	THF	r.t.	24	4	OH	O(CO)CH2Cl	34
3	1	THF	r.t.	24	5	O(CO)CH2Cl	O(CO)CH2Cl	95
4	4	DMF	90	3	6	OH	O(CO)CH2N3	64
5	5	DMF	90	3	7	O(CO)CH2N3	O(CO)CH2N3	72

**Table 2:** Summary synthesis of betulin glycoconjugates **15–24**.

Entry	Substrate				Product		
	Analog BN	No.	Sugar	R^1^, R^2^, R^3^	No.	Linker	Yield, %
STRATEGY I							
1	2	12a	Glc	OAc	15a	28-OCH2Triaz	99
2	2	12b	Gal	OAc	15b	28-OCH2Triaz	97
3	2	14a	Glc	OAc	16a	28-OCH2TriazCH2(CO)NH	98
4	2	14b	Gal	OAc	16b	28-OCH2TriazCH2(CO)NH	97
5	3	12a	Glc	OAc	17a	3,28-di-OCH2Triaz	99
6	3	12b	Gal	OAc	17b	3,28-di-OCH2Triaz	99
7	3	14b	Gal	OAc	18b	3,28-di-OCH2TriazCH2(CO)NH	82
STRATEGY II							
8	6	10a	Glc	OAc	19a	28-O(CO)CH2TriazCH2O	97
9	7	10a	Glc	OAc	20a	3,28-di-O(CO)CH2TriazCH2O	87
10	7	−	−	−	21	3,28-di-O(CO)CH2TriazOH	77
DEPROTECTION							
11	15a	−	Glc	OH	22a	28-OCH2Triaz	87
12	15b	−	Gal	OH	22b	28-OCH2Triaz	99
13	16b	−	Gal	OH	23b	28-OCH2TriazCH2(CO)NH	96
14	17a	−	Glc	OH	24a	3,28-di-OCH2Triaz	87
15	17b	−	Gal	OH	24b	3,28-di-OCH2Triaz	69

**Table 3:** Summary of cytotoxicity of BN and the betulin glycoconjugates.

Compound	Activity IC_50_ [µM] ^a,b^	
	MCF-7	NHDF
1	12.40 ± 0.14	11.60 ± 0.22
15a	65.28 ± 0.68	60.62 ± 0. 64
15b	47.70 ± 0.72	47.61 ± 0.86
16a	29.77 ± 0.52	29.82 ± 0.45

^a^ Cytotoxicity was evaluated using the CCK-8 assay; ^b^ Incubation time 48 h.

**Table 4:** LogP of BN and betulin analogs **2**–**24**.

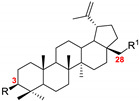			
No.	R (C3)	R^1^ (C28)	logP
1	OH	OH	7.16
2	OH	OCH2C≡CH	7.94
3	OCH2C≡CH	OCH2C≡CH	8.54
4	OH	O(CO)CH2Cl	8.23
5	O(CO)CH2Cl	O(CO)CH2Cl	8.86
6	OH	O(CO)CH2N3	8.31
7	O(CO)CH2N3	O(CO)CH2N3	8.94
15a	OH	OCH2TriazGlc(OAc)	7.59
16a	OH	OCH2TriazCH2(CO)NHGlc(OAc)	6.69
17a	OCH2TriazGlc(OAc)	OCH2TriazGlc(OAc)	8.01
18b	OCH2TriazCH2(CO)NHGal(OAc)	OCH2TriazCH2(CO)NHGal(OAc)	6.22
19a	OH	O(CO)CH2TriazCH2OGlc(OAc)	7.45
20a	O(CO)CH2TriazCH2OGlc(OAc)	O(CO)CH2TriazCH2OGlc(OAc)	7.74
21	O(CO)CH2TriazOH	O(CO)CH2TriazOH	6.38
22a	OH	OCH2TriazGlc(OH)	5.21
23b	OH	OCH2TriazCH2(CO)NHGal(OH)	4.31
24a	OCH2TriazGlc(OH)	OCH2TriazGlc(OH)	3.25

## Supplementary Materials

The following are available online. Supporting information includes experimental procedures and spectroscopic properties of synthesized compounds (**4**, **5**, **9**–**14**), ^1^H NMR, ^13^C NMR spectra of all synthesized compounds (**1**–**24**) and gHSQC and FT-IR for selected compounds.
