# Supplementary material for: Glycoconjugation of Betulin Derivatives Using Copper-Catalyzed 1,3-Dipolar Azido-Alkyne Cycloaddition Reaction and a Preliminary Assay of Cytotoxicity of the Obtained Compounds
Source: Molecules. 2020 Dec 18;25(24):6019. doi: 10.3390/molecules25246019 (PMC7766341; doi:10.3390/molecules25246019)
Supplement: Supplementary file 1 [file molecules-25-06019-s001.pdf]

Supporting Information

Glycoconjugation of betulin derivatives using copper-catalyzed 1,3-dipolar azido-alkyne cycloaddition reaction and a preliminary assay of cytotoxicity of the obtained compounds

Mirosława Grymel [1,2,*], Gabriela Pastuch-Gawołek [1,2], Anna Lalik [2,3], Mateusz Zawojak [1], Seweryn Boczek [1], Monika Krawczyk [1,2], and Karol Erfurt [4]

[1] Department of Organic Chemistry, Bioorganic Chemistry and Biotechnology, Silesian University of Technology, B. Krzywoustego 4, 44-100 Gliwice, Poland
[2] Biotechnology Center, Silesian University of Technology, B. Krzywoustego 8, 44-100 Gliwice, Poland
[3] Department of Systems Biology and Engineering, Silesian University of Technology, Akademicka 16, 44-100 Gliwice, Poland
[4] Department of Chemical Organic Technology and Petrochemistry, Silesian University of Technology, B. Krzywoustego 4, 44-100 Gliwice, Poland

* Correspondence: Miroslawa.Grymel@polsl.pl; Tel.: +48 032-237-1873 (M.G.)

1. Experimental procedures and spectroscopic properties of synthesized compounds ……..S2-S10
2. ^{1}H NMR, ^{13}C NMR of all synthesized compounds (**1-24**) and gHSQC for selected new compounds ………………………………………………..…...S11-S81
3. IR spectra for selected compounds………………….……...……………………..……...…..S82-S101

1. Procedure for the Synthesis of Betulin Analogs

1.1. *28-O-(2-chloroacetyl)betulin* **4**

A solution of betulin **1** (0.226 mmol, 100.0 mg) and catalytic amount of 4-dimethylaminopyridine (DMAP, 0.023 mmol, 2.8 mg) in dry THF (0.4 mL) was stirred, treated dropwise with a diisopropylethyl amine (DIPEA 0.339 mmola, 43.8 mg, 0.059 mL) and then chloroacetyl chloride (0.271 mmola, 30.6 mg, 0.022 mL). The resulting mixture was left on the magnetic stirrer for 30 min at 0-5 °C and then for 24 h at room temperature. The reaction progress was monitored on TLC in an eluents system DCM:MeOH (100:1). Extracted with AcOEt (4 x 4 mL), dried over $MgSO_4$ and evaporated in vacuo. The crude products were purified using column chromatography (DCM:MeOH, gradient: 100:1).

Product **4** was obtained as a white solid (39.9 mg, 34% yield); m.p.: 152-154 °C; $[\alpha]^{25}_{D}$ = +31.9 (c 0.5, $CHCl_3$); ^{1}H NMR (600 MHz, $CDCl_3$): δ_H 0.76, 0.82, 0.97, 0.98, 1.03 (all s, 3H each, H-23–H-27), 1.68 (s, 3H, H-30), 0.60-2.10 (m, 25H, CH, CH_2), 2.44 (td, 1H, J_1 5.8 Hz, J_2 11.0 Hz, H-19), 3.18 (dd, 1H, J_1 4.8 Hz, J_2 11.4 Hz, H-3), 3.97 (d, 1H, *J* 11.4 Hz, H-28b), 4.09 (s, 2H, CH_2Cl), 4.40 (d, 1H, *J* 10.8 Hz, H-28a), 4.60 (d, 1H, *J* 1.2 Hz, H-29b), 4.70 (d, 1H, *J* 1.8 Hz, H-29a) ppm; ^{13}C NMR (**Table 1**).

1.2. *3,28-O,O'-di(2-chloroacetyl)betulin* **5**

A solution of betulin **1** (0.226 mmol, 100.0 mg) and DMAP (0.023 mmol, 2.8 mg) in THF (0.83 mL) was stirred, treated dropwise with a DIPEA (0.339 mmola, 43.8 mg, 0.059 mL) and then chloroacetyl chloride (0.576 mmola, 65.1mg, 0.046 mL). The resulting mixture was left on the magnetic stirrer for 30 min at 0-5 °C and then for 24 h at room temperature. The reaction progress was monitored on TLC in an eluents system DCM:MeOH (100:1). Then, extracted with AcOEt (5 x 4 mL) and dried over $MgSO_4$. After that followed by filtration and evaporated in vacuo. The crude products were purified using column chromatography (DCM:MeOH, gradient: 1000:1 to 100:1).

Product **5** was obtained as a white solid (127.8 mg, 95% yield); m.p.: 72-73 °C; $[\alpha]^{25}_{D}$ = +16.2 (c 0.5, $CHCl_3$); ^{1}H NMR (600 MHz, $CDCl_3$): δ_H 0.86, 0.87, 0.98, 1.04 (all s, 3H each, H-23–H-27), 1.69 (s, 3H, H-30), 0.60-2.10 (m, 24H, CH, CH_2), 2.44 (td, 1H, J_1 5.8 Hz, J_2 11.0 Hz, H-19), 3.96 (d, 1H, *J* 10.8 Hz, H-28b), 4.04 (d, 2H, *J* 14.6/5,4 Hz, CH_2Cl), 4.09 (s, 2H, CH_2Cl), 4.41 (dd, 1H, J_1 1.2 Hz, J_2 10.8 Hz, H-28a), 4.60 (dd, 1H, J_1 2.1 Hz, J_2 10.1 Hz, H-3), 4.69 (s, br, 1H, H-29b), 4.70 (s, br, 1H, H-29a) ppm; ^{13}C NMR (**Table 1**); IR (ATR) ν: 2943, 2872, 1731, 1455, 1391, 1288, 1183, 978, 884, 786, 695 cm^{-1}.

Table 1. ^{13}C NMR spectrum of betulin analogs **2-7** (150 MHz, $CDCl_3$).

	At. C	BN	2	3	4	5	6	7
BETULIN SKELETON	C-1	38.72	38.69	38.55[b]	38.60	37.92	38.62	37.89
	C-2	27.41	27.39	27.99	27.27	23.46	27.30	23.68
	C-3[a]	78.98	78.97	85.86	78.79	83.24	78.84	83.21
	C-4	38.87	38.86	38.55[b]	38.74	38.22	38.77	38.34
	C-5	55.31	55.28	55.87	55.18	55.24	55.21	55.36
	C-6	18.31	18.29	18.25	18.16	18.03	18.19	18.15
	C-7	34.25	34.22	34.24	34.07	33.99	34.09	34.08
	C-8	40.94	40.98	41.03	40.88[c]	41.16	40.79	40.91
	C-9	50.42	50.42	50.41	50.23	50.15	50.26	50.25
	C-10	37.18	37.17	37.12	37.02	36.96	37.06	37.06
	C-11	20.84	20.87	20.91	20.65	20.70	20.67	20.74
	C-12	25.24	25.22	25.24	25.06	25.03	25.09	25.06
	C-13	37.33	37.58	37.58	37.54	37.55	37.58	37.66
	C-14	42.73	42.66	42.65	42.59	42.63	42.62	42.67[b]
	C-15	27.07	27.14	27.12	26.91	27.84	27.89	27.94
	C-16	29.19	29.79	29.71	29.40	29.41	29.59[b]	29.67
	C-17	47.80	47.99[b]	47.02	46.40	46.43	46.33	46.43
	C-18	47,88	48.91	48.92	48.70	48.71	48.72	48.80
	C-19[a]	48,80	47.99[b]	48.00	47.55	47.61	47.57	47.69
	C-20	150.46	150.64	150.64	149.75	149.78	149.76	149.84
	C-21	29.77	29.90	29.91	29.52	29.53	29.59[b]	29.67[b]
	C-22	33.97	34.69	34.69	34.32	34.35	34.39	34.49
	C-23	27.99	27.99	27.99	27.88	26.93	26.92	27.00
	C-24	15.35	15.37	16.00	15.26	16.06	15.27	16.04
	C-25	16.10	16.11	16.10	15.99	16.34	16.00	16.54[c]
	C-26	15.99	15.98	15.99	15.90	15.93	15.94	16.15[c]
	C-27	14.77	14.83	14.79	14.66	14.67	14.69	14.75
	C-28[a]	60.58	68.27	68.25	64.63	64.67	64.31	64.40
	C-29[a]	109.67	109.56	109.56	109.88	109.96	109.92	110.07
	C-30[a]	19.09	19.09	19.11	19.02	19.02	19.04	19.05
LINKER	$O\underline{C}H_2C\equiv CH$	–	58.61	58.62; 56.42	–	–	–	–
	$OCH_2\underline{C}\equiv CH$	–	80.51	80.96; 80.50	–	–	–	–
	$OCH_2C\equiv \underline{C}H$	–	74.01	74.01; 73.41	–	–	–	–
	$O(\underline{C}O)CH_2$	–	–	–	167.62	167.67<br>167.04	168.65	168.75<br>168.15
	$O(CO)\underline{C}H_2$	–	–	–	40.76[c]	40.92; 40.81	50.41	50.67; 50.52

[a]Signals were assigned based on experiments gHSQC; [b] Two overlapping signals; [c] Possible reverse signal assignment.

Table 2. ^{13}C NMR spectrum of betulin glycoconjugates **15-20** (150 MHz, $CDCl_3$).

	At. C	15a	15b	16a	16b	17a	17b	18b	19a	20a
BETULIN SKELETON	C-1	38.73	38.72	38.70	38.71	38.52	38.53	38.52	38.71	38.27
	C-2	27.42	27.42	27.40	27.39	22.83	22.87	22.86	27.39	23.61
	C-3[a]	78.95	78.96	78.99	78.98	86.65	86.63	86.89	78.94	83.86
	C-4	38.88	38.88	38.86	38.87	38.81	38.82	38.85	38.87	37.90[z]
	C-5	55.31	55.30	55.28	55.29	55.68	55.68	55.69	55.29	55.30
	C-6	18.32	18.33	18.33	18.33	18.24	18.26	18.26	18.28	18.11
	C-7	34.26	34.25	34.23	34.24	34.24	34.24	34.24	34.18	34.05
	C-8	40.92	40.93	40.88	40.89	40.94	40.95	40.93	40.88	40.89
	C-9	50.42	50.41	50.38	50.39	50.36	50.36	50.36	50.33	50.21
	C-10	37.17	37.17	37.15	37.16	37.11	37.12	37.14	37.15	37.03[z]
	C-11	20.85	20.84	20.83	20.84	20.85	20.86	20.87	20.78	21.96
	C-12	25.22	25.22	25.18	25.19	25.20	25.20	25.20	25.16	25.08
	C-13	37.51	37.50	37.49	37.50	37.48	37.48	37.50	37.68	37.65
	C-14	42.68	42.69	42.64	42.65	42.65	42.66	42.65	42.71	42.73
	C-15	28.00	28.00	28.01	28.01	27.97	27.96	28.10	27.99	28.01
	C-16	29.85	29.86	29.69[x]	29.88[y]	29.83	29.84	29.69	29.44	29.43
	C-17	47.26	47.26	47.23	47.24	47.25	47.25	47.25	46.45	46.45
	C-18	48.85	48.84	48.83	48.84	48.82	48.83	48.85	48.80	48.77
	C-19[a]	47.88	47.87	47.88	47.89	47.84	47.86	47.90	47.64	47.65
	C-20	150.57	150.61	150.53	150.53	150.57	150.59	150.54	149.73[m]	149.68
	C-21	29.91	29.95	29.86[x]	29.86[y]	29.89	29.94	29.88	29.60	29.57
	C-22	34.77	34.79	34.73	34.74	34.76	34.78	34.75	34.39	34.38
	C-23	27.42	27.23	27.16	27.17	27.18	27.20	27.17	27.01	27.00
	C-24	15.37	15.37	15.41	15.40	16.25	16.26	16.30	15.36	16.00
	C-25	16.11	16.11	16.12	16.12	16.12	16.13	16.15	16.10	16.12
	C-26	16.03	16.00	15.93	15.94	16.02	16.00	15.96	16.01	16.39
	C-27	14.79	14.80	14.79	14.79	14.73	14.74	14.76	14.78	14.75
	C-28[a]	68.94	68.94	68.95	68.98	68.92	68.91	69.01	64.96	68.37[o]
	C-29[a]	109.60	109.60	109.64	109.63	109.61	109.62	109.65	110.10	110.16
	C-30[a]	19.06	19.06	19.08	19.08	19.05	19.05	19.09	19.11	19.10
LINKER	CH-triazol[a]	120.58	120.76	123.76	123.86	120.58<br>120.50	120.77<br>120.66	123.75<br>123.61	124.28	124.25<br>124.24
	C-triazol	146.62	146.46	146.71	146.60	147.26<br>146.59	147.06<br>146.42	147.49<br>146.77	149.73[m]	144.65<br>144.55
	CH_2-Tri	64.95	64.91	65.14	65.13	64.93<br>62.88	64.88<br>62.88	65.21<br>63.20	62.93	62.94<br>61.80[s]
	CH_2(CO)	–	–	52.59	52.50	–	–	52.71<br>52.65	–	–
	CH_2O	–	–	–	–	–	–	–	50.85	51.05<br>50.83
	C=O	–	–	165.68	165.67	–	–	165.68<br>165.55	166.55	166.56<br>165.90

[a]Signals were assigned based on experiments gHSQC; [b-w] Two overlapping signals; [x-z] Possible reverse signal assignment.

cont. Table 2. ^{13}C NMR spectrum of betulin glycoconjugates **15-20** (150 MHz, $CDCl_3$).

SUGAR	C-1 ÷ C-6 (Glc or Gal)	85.73	86.27	78.41	78.61	85.69	86.22	78.75	72.79	72.80[p]
		75.12	74.02	73.80	72.56	85.62	86.17	78.72	71.94	71.94[s]
		72.73	70.89	72.51	70.71	75.08	73.98[g]	72.59	71.21	71.22[r]
		70.32	67.85	70.32	68.06	75.04	70.94	72.57	68.36	68.37[o]
		67.72	66.87	68.01	67.08	72.81	70.86	70.67	61.80	61.81[t]
		61.58	61.15	61.56	61.08	72.71	67.86	70.64	50.86	50.83[q]
						70.31	67.74	68.06		
						70.18	66.91[f]	68.00		
						67.71	61.18[h]	67.06[k]		
						67.70		61.11		
						61.57[c]		61.09		
	$\underline{C}H_3CO$[a]	20.20	20.30	20.54	20.50	20.17[d]	20.29;	20.50[l];	20.60[n]	20.60[u]
		20.51	20.49	20.73	20.60	20.51[d]	20.27	20.59[l]	20.67	20.65[u]
		20.53	20.62	20.56[b]	20.64	20.53[d]	20.49[i]	20.62	20.74	20.66[u]
		20.66	20.66		20.67	20.66[d]	20.63[i]	20.60		20.77[u]
							20.66[i]	20.67[l]		
	$CH_3\underline{C}O$	168.87	169.04	169.49	169.73	168.79	168.93	169.71[ł]	169.42	160.42[w]
		169.32	169.80	169.84	169.98	168.86	169.03	169.98[ł]	169.45	169.43[w]
		169.90	169.95	170.57	170.37	169.32	169.79	170.34[ł]	170.18	170.16[w]
		170.45	170.28	170.86	171.00	169.89	169.95[j]	170.92	170.65	170.64[w]
						169.91	170.00	171.03		
						170.44	170.28			
						170.46[e]	170.31			

[a]Signals were assigned based on experiments gHSQC; [b-w] Two overlapping signals; [x-z] Possible reverse signal assignment.

Table 3. ^{13}C NMR spectrum of betulin glycoconjugates **21-24** (150 MHz, MeOD).

	At. C	21^{CDCl_3}	22a	22b	23b	24a	24b
BETULIN SKELETON	C-1	38.26	40.14	39.99	40.01	39.85	39.83
	C-2	23.58	28.39	28.34	28.36	28.41	28.37
	C-3[a]	83.85	79.75	79.71	78.48	88.07	88.04
	C-4	37.88	40.14	40.09	40.12	39.96	39.97
	C-5	55.28	56.90	56.86	56.90	57.23	57.22
	C-6	18.10	18.40	19.50	19.53	18.40	18.41
	C-7	34.04	35.53	35.46	35.52	35.52	35.48
	C-8	40.88	42.21	42.15	42.18	42.25	42.22
	C-9	50.20	51.94	51.90	51.92	51.93	51.91
	C-10	37.02	38.35	38.32	38.35	38.37	38.34
	C-11	20.73	22.06	22.02	22.05	22.10	22.08
	C-12	25.07	26.71	26.64	26.67	26.71	26.68
	C-13	37.64	38.99	38.95	38.98	39.00	39.96
	C-14	42.71	43.82	43.79	43.80	43.85	43.83
	C-15	28.00	28.68	28.65	28.67	28.67	28.66
	C-16	29.43	30.75	30.99	30.78	30.76	30.78
	C-17	46.43	-[c]	-[c]	-[c]	-[c]	-[c]
	C-18	48.75	50.24	50.17	50.23	50.25	50.22
	C-19	47.65	-[c]	-[c]	-[c]	-[c]	-[c]
	C-20	149.96	151.86	151.87	151.91	151.89	151.89
	C-21	29.56	31.05	30.79	31.04	31.07	31.05
	C-22	34.37	35.83	35.82	35.86	35.84	35.84
	C-23	26.98	28.12	28.09	28.12	28.40	28.42
	C-24	16.00	16.15	16.16	16.17	16.70	16.80
	C-25	16.11	16.76	16.76	16.77	16.82	16.83
	C-26	16.36	16.68	16.62	16.59	16.79	16.66
	C-27	14.75	15.33	15.28	15.30	15.32	15.30
	C-28[a]	64.92	69.88	69.67	69.49	69.82	69.77
	C-29[a]	110.12	110.25	110.28	110.24	110.24	110.27
	C-30[a]	19.09	19.52	19.41	19.44	19.43	19.42
LINKER	CH-triazol[a]	123.08[b]	124.41	123.87	126.86	124.33<br>124.18	123.89<br>123.64
	C-triazol	149.66<br>148.09	146.36	146.54	146.42	146.99<br>146.42	147.19<br>146.56
	CH_2-Tri	51.08; 50.86	65.31	65.33	65.15	65.36; 63.50	65.38; 63.56
	CH_2(CO)	56.50; 55.23	–	–	51.92	–	–
	C=O	165.61<br>165.97	–	–	168.57	–	–
SUGAR	C-1 ÷ C-6<br>(Glc or Gal)		89.70; 81.18<br>78.60; 74.10<br>71.00; 62.47	90.28; 79.90<br>75.40; 71.50<br>70.32; 62.30	81.77; 79.75<br>75.82; 71.51<br>70.49; 69.49	89.65[b]; 81.20[b]<br>78.64[b]; 74.11[b]<br>71.00[b]; 62.49[b]	90.27; 79.02<br>75.44; 71.56<br>71.55; 80.04<br>62.50; 62.34

[a]Signals were assigned based on experiments gHSQC; [b]Two overlapping signals; [c]Signal under MeOD.

2. Synthesis of Sugar Derivatives 9 – 14

2.1. 1,2,3,4,6-Penta-O-acetyl-β-D-glucopyranose ***9a***

Dry sodium acetate (36.3 mmol, 3.0 g) and anhydrous acetic anhydride (278.0 mmol, 26 mL) were placed in a three-necked flask equipped with a reflux condenser. Anhydrous glucose (27.8 mmol, 5.0 g) was added in portions and then the mixture was stirred and heated to reflux for 1 h. The reaction progress was monitored on TLC in an eluents system toluene:AcOEt (1:1). After that the mixture was cooled and poured into ice water and stirred vigorously until until the acetic anhydride was completely hydrolyzed and the product precipitated. The product was separated by filtration in vacuo.

Product **9a** was obtained as a white solid (10.4 g, 96% yield); m.p.: 131-132 °C; $[\alpha]^{25}_{D}$ = 4.0 (c 1.0, $CHCl_3$); ^{1}H NMR (400 MHz, $CDCl_3$): δ_H 2.02, 2.03, 2.04, 2.09, 2.12 (5s, 15H, CH_3CO), 3.84 (ddd, 1H, J_1 2.3 Hz, J_2 4.5 Hz, J_3 10.2 Hz, H-5), 4.12 (dd, 1H, J_1 2.3 Hz, J_2 12.5 Hz, H-6a), 4.29 (dd, 1H, J_1 4.5 Hz, J_2 12.5 Hz, H-6b), 5.12 (dd, 1H, J_1 9.4 Hz, J_2 10.2 Hz, H-4), 5.14 (dd, 1H, J_1 8.3 Hz, J_2 9.4 Hz, H-2), 5.26 (dd, 1H, J_1 9.4 Hz, J_2 9.4 Hz, H-3), 5.72 (d, 1H, J 8.3 Hz, H-1); ^{13}C NMR (100 MHz, $CDCl_3$): δ_C 20.55, 20.56, 20.70, 20.81 ($\underline{C}H_3CO$), 61.48 (C-6), 67.79, 70.26, 72.75, 72.81 (C-2, C-3, C-4, C-5), 91.72 (C-1), 168.94, 169.23, 169.38, 170.08, 170.59 ($CH_3\underline{C}O$).

2.2. 1,2,3,4,6-Penta-O-acetyl-β-D-galactopyranose ***9b***

Dry sodium acetate (36.3 mmol, 3.0 g) and anhydrous acetic anhydride (278.0 mmol, 26 mL) were placed in a three-necked flask equipped with a reflux condenser. Anhydrous galactose (27.8 mmol, 5.0 g) was added in portions and then the mixture was stirred and heated to reflux for 1 h. The reaction progress was monitored on TLC in an eluents system toluene:AcOEt (1:1). After that the mixture was cooled and poured into ice water and stirred vigorously until until the acetic anhydride was completely hydrolyzed and the product precipitated. The product was separated by filtration in vacuo.

Product **9b** was obtained as a white solid (8.8 g, 81% yield); m.p.: 141-142 °C; $[\alpha]^{24}_{D}$ = 27.1 (c 1.0, $CHCl_3$); ^{1}H NMR (400 MHz, $CDCl_3$): δ_H 1.99, 2.04, 2.12, 2.16 (5s, 15H, CH_3CO), 4.06 (ddd, 1H, J_1 1.1 Hz, J_2 4.7, J_3 7.1 Hz, H-5), 4.12 (dd, 1H, J_1 4.7 Hz, J_2 11.4 Hz, H-6a), 4.17 (dd, 1H, J_1 7.1 Hz, J_2 11.4 Hz, H-6b), 5.08 (dd, 1H, J_1 3.4 Hz, J_2 10.4 Hz, H-3), 5.32 (dd, 1H, J_1 8.3 Hz, J_2 10.4 Hz, H-2), 5.42 (dd, 1H, J_1 1.1 Hz, J_2 3.4 Hz, H-4), 5.70 (d, 1H, J 8.3 Hz, H-1); ^{13}C NMR (100 MHz, $CDCl_3$): δ_C 20.48, 20.57, 20.60, 20.76 ($\underline{C}H_3CO$), 61.00 (C-6), 66.78, 67.83, 70.81, 71.68 (C-2,C-3, C-4, C-5), 92.14 (C-1), 168.91, 169.31, 169.89, 170.06, 170.28 ($CH_3\underline{C}O$).

2.3. Propargyl 2,3,4,6-tetra-O-acetyl-β-D-glucopyranoside ***10a***

To a solution of per-O-acetylated-D-glucose **9a** (1.281 mmol, 0.5 g) and propargyl alcohol (1.734 mmol, 100 μL) in anhydrous DCM (5 mL) at 0 °C the boron trifluoride diethylether (1.945 mmol, 240 μL) was added dropwise. The reaction mixture was stirred at room temperature for 2 h. The reaction progress was monitored on TLC in an eluents system toluene:AcOEt (2:1). After completion of the reaction, the reaction mixture was diluted with DCM and washed with ice-cold water (1 x 10 mL), saturated $NaHCO_3$ (1 x 10 mL) and saturated brine solution (1 x 10 mL). The organic layer was dried over anhydrous $MgSO_4$ and was concentrated in vacuo. The crude product was purified using column chromatography (toluene:AcOEt, gradient: 15:1 to 8:1).

Product **10a** was obtained as as a white solid (0.381 g, 77% yield); m.p.: 111-112 °C; $[\alpha]^{24}_{D}$ = 38.6 (c 1.0, $CHCl_3$); ^{1}H NMR (400 MHz, $CDCl_3$): δ_H 2.01, 2.03, 2.06, 2.09 (4s, 12H, CH_3CO), 2.48 (t, 1H, J 2.3 Hz, CH), 3.73 (ddd, 1H, J_1 2.3 Hz, J_2 4.7, J_3 10.2 Hz, H-5), 4.15 (dd, 1H, J_1 2.3 Hz, J_2 12.3 Hz, H-6a), 4.27 (dd, 1H, J_1 4.7 Hz, J_2 12.3 Hz, H-6b), 4.32-4.42 (m, 2H, CH_2), 4.78 (d, 1H, J 7.8 Hz, H-1), 5.01 (dd, 1H, J_1 7.8 Hz, J_2 9.4 Hz, H-2), 5.10 (dd, 1H, J_1 9.8 Hz, J_2 10.2 Hz, H-4), 5.24 (dd, 1H, J_1 9.4 Hz, J_2 9.8 Hz, H-3); ^{13}C NMR (100 MHz, $CDCl_3$): δ_C 20.53, 20.54, 20.62, 20.65 (CH_3CO), 55.88 ($\underline{C}H_2CCH$), 61.74 (C-6),

68.30, 70.94, 71.90, 72.74 (C-2, C-3, C-4, C-5), 75.44 (C≡$\underline{C}$H), 78.10 ($\underline{C}$≡CH), 98.10 (C-1), 169.34, 169.37, 170.19, 170.59 ($CH_3\underline{C}O$).

*2.4. 2,3,4,6-Tetra-O-acetyl-α-D-glucopyranosyl bromide **11a***

1,2,3,4,6-Penta-*O*-acetyl-β-D-glucopyranose **9a** (7.7 mmol, 3.0 g) was dissolved in glacial acetic acid (15 mL). The flask was placed into an ice bath, and then a 33 % solution of HBr in AcOH (158.0 mmol, 9 mL) was added dropwise to the mixture. Reaction was carried out on the magnetic stirrer for 1 h at room temperature. The reaction progress was monitored on TLC in an eluents system toluene:AcOEt (1:1). After the reaction was completed, the solution was diluted with CH_2Cl_2 (50 mL) and then poured into ice-water (70 mL). The two-phase system was separated, the aqueous phase was washed with CH_2Cl_2 (2 x 50 mL) and the combined organic phases were washed with saturated $NaHCO_3$ solution (1 x 50 mL) and brine (2 x 40 mL). The organic phase was dried with $MgSO_4$, filtered and the filtrate was evaporated in vacuo. The crude products were purified using column chromatography (toluene:AcOEt, gradient: 10:1 to 6:1).

Product **11a** was obtained as a white solid (2.9 g, 91% yield); m.p.: 87-88 °C; $[\alpha]^{20}_D$ = 182.0 (c 1.0, $CHCl_3$); ^{1}H NMR (400 MHz, $CDCl_3$): δ_H 2.04, 2.05, 2.10, 2.10 (4s, 12H, CH_3CO), 4.13 (dd, 1H, J_1 1.8 Hz, J_2 12.5 Hz, H-6a), 4.29 (ddd, 1H, J_1 1.8 Hz, J_2 3.9 Hz, J_3 10.2 Hz, H-5), 4.33 (dd, 1H, J_1 3.9 Hz, J_2 12.5 Hz, H-6b), 4.84 (dd, 1H, J_1 4.1 Hz, J_2 10.0 Hz, H-2), 5.16 (dd, 1H, J_1 9.8 Hz, J_2 10.2 Hz, H-4), 5.56 (dd, 1H, J_1 9.8 Hz, J_2 10.0 Hz, H-3), 6.61 (d, 1H, J_1 4.1 Hz, H-1); ^{13}C NMR (100 MHz, $CDCl_3$): δ_C 20.55, 20.61, 20.64, 20.66 ($\underline{C}H_3CO$), 60.97 (C-6), 67.21, 70.19, 70.63, 72.16 (C-2,C-3, C-4, C-5), 86.57 (C-1), 169.45, 169.77, 169.82 170.48 ($CH_3\underline{C}O$).

*2.5. 2,3,4,6-Tetra-O-acetyl-α-D-galactopyranosyl bromide **11b***

1,2,3,4,6-Penta-*O*-acetyl-β-D-galactopyranose **9b** (7.7 mmol, 3.0 g) was dissolved in glacial acetic acid (15 mL). The flask was placed in an ice bath, and then a 33 % solution of HBr in AcOH (158.0 mmol, 9 mL) was added dropwise to the mixture. Reaction was carried out on the magnetic stirrer for 1 h at room temperature. The reaction progress was monitored on TLC in an eluents system toluene:AcOEt (1:1). After the reaction was completed, the solution was diluted with CH_2Cl_2 (50 mL) and then poured into ice-water (70 mL). The two-phase system was separated, the aqueous phase was washed with CH_2Cl_2 (2 x 50 mL) and the combined organic phases were washed with saturated $NaHCO_3$ solution (1 x 50 mL) and brine (2 x 40 mL). The organic phase was dried with $MgSO_4$, filtered and the filtrate was evaporated in vacuo. The crude products were purified using column chromatography (toluene:AcOEt, gradient: 10:1 to 6:1).

Product **11b** was obtained as a white solid (2.8 g, 90% yield); m.p.: 83-85 °C; $[\alpha]^{21}_D$ = 212.0 (c 1.0, $CHCl_3$); ^{1}H NMR (400 MHz, $CDCl_3$): δ_H 2.01, 2.06, 2.11, 2.15 (4s, 12H, CH_3CO), 4.11 (dd, 1H, J_1 6.8 Hz, J_2 11.4 Hz, H-6a), 4.19 (dd, 1H, J_1 6.4 Hz, J_2 11.4 Hz, H-6b), 4.48 (ddd, J_1 1.3 Hz, J_2 6.4 Hz, J_3 6.8 Hz, H-5), 5.05 (dd, 1H, J_1 4.0, J_2 10.6 Hz, H-2), 5.41 (dd, 1H, J_1 3.3 Hz, J_2 10.6 Hz, H-3), 5.52 (dd, 1H, J_1 1.3 Hz, J_2 3.3 Hz, H-4), 6.70 (d, 1H, J 4.0 Hz, H-1); ^{13}C NMR (100 MHz, $CDCl_3$): δ_C 20.52, 20.55, 20.60, 20.71 ($\underline{C}H_3CO$), 60.80 (C-6), 66.97, 67.76, 67.98, 71.05 (C-2, C-3, C-4, C-5), 88.10 (C-1), 169.70, 169.84, 170.01, 170.26 ($CH_3\underline{C}O$).

*2.6. 2,3,4,6-Tetra-O-acetyl-β-D-glucopyranosyl azide **12a***

2,3,4,6-Tetra-*O*-acetyl-α-D-glucopyranosyl bromide **11a** (5.3 mmol, 2.2 g) was dissolved in $CHCl_3$ (20 mL), followed by addition of saturated $NaHCO_3$ solution (20 mL), NaN_3 (27.7 mmol, 1.8 g) and TBASH (5.3 mmol, 1.8 g). Reaction was carried out on the magnetic stirrer for 2 h at room temperature. The reaction progress was monitored on TLC in an eluents system toluene:AcOEt (1:1). After the reaction was completed, the two-phase system was separated. The aqueous phase was washed with $CHCl_3$ (1 x 20 mL) and the combined organic phases were washed with brine (1 x 20 mL), saturated $NaHCO_3$ solution (1 x 20 mL) and brine (1 x 20 mL). The organic phase was dried with

$MgSO_4$, filtered and the filtrate was evaporated in vacuo. The crude products were purified using column chromatography (toluene:AcOEt, gradient: 10:1 to 6:1).

Product **12a** was obtained as a white solid (1.8 g, 90% yield); m.p.: 125-128 °C; $[\alpha]^{25}_{D}$ = -30.6 (c 1.0, $CHCl_3$); ^1H NMR (400 MHz, $CDCl_3$): δ_H 2.01, 2.03, 2.08, 2.10 (4s, 12H, CH_3CO), 3.80 (ddd, 1H, J_1 2.3 Hz, J_2 4.8 Hz, J_3 10.0 Hz, H-5), 4.18 (dd, 1H, J_1 2.3 Hz, J_2 12.5 Hz, H-6a), 4.28 (dd, 1H, J_1 4.8 Hz, J_2 12.5 Hz, H-6b), 4.65 (d, 1H, J_1 8.9 Hz, H-1), 4.96 (dd, 1H, J_1 8.9 Hz, J_2 9.5 Hz, H-2), 5.11 (dd, 1H, J_1 9.5 Hz, J_2 10.0 Hz, H-4), 5.22 (dd~t, 1H, J_1 9.5 Hz, J_2 9.5 Hz, H-3); ^{13}C NMR (100 MHz, $CDCl_3$): δ_C 20.46, 20.48, 20.62 ($\underline{C}H_3CO$), 61.60 (C-6), 67.84, 70.59, 72.55, 73.98 (C-2, C-3, C-4, C-5), 87.85 (C-6), 169.10, 169.21, 170.02, 170.50 ($CH_3\underline{C}O$).

*2.7. 2,3,4,6-Tetra-O-acetyl-β-D-galactopyranosyl azide **12b***

2,3,4,6-Tetra-*O*-acetyl-*α*-D-galactopyranosyl bromide **11b** (6.7 mmol, 2.8 g) **3** was dissolved in $CHCl_3$ (25 mL), followed by addition of saturated $NaHCO_3$ solution (25 mL), NaN_3 (35.4 mmol, 2.3 g) and TBASH (6.7 mmol, 2.3 g). Reaction was carried out on the magnetic stirrer for 2 h at room temperature. The reaction progress was monitored on TLC in an eluents system toluene:AcOEt (1:1). After the reaction was completed, the two-phase system was separated. The aqueous phase was washed with $CHCl_3$ (1 x 25 mL) and the combined organic phases were washed with brine (1 x 25 mL), saturated $NaHCO_3$ solution (1 x 25 mL) and brine (1 x 25 mL). The organic phase was dried with $MgSO_4$, filtered and the filtrate was evaporated in vacuo. The crude products were purified using column chromatography (toluene:AcOEt, gradient: 10:1 to 6:1).

Product **12b** was obtained as a white solid (2.1 g, 85% yield); m.p.: 96-98 °C; $[\alpha]^{25}_{D}$ = -15.0 (c 1.0, $CHCl_3$); ^1H NMR (400 MHz, $CDCl_3$): δ_H 1.99, 2.06, 2.09, 2.17 (4s, 12H, CH_3CO), 3.95-4.05 (m, 1H, H-5), 4.12-4.22 (m, 2H, H-6a, H-6b), 4.60 (d, 1H, J 8.7 Hz, H-1), 5.04 (dd, 1H, J_1 3.4 Hz, J_2 10.3 Hz, H-3), 5.17 (dd, 1H, J_1 8.7 Hz, J_2 10.3 Hz, H-2), 5.43 (dd, 1H, J_1 1.0 Hz, J_2 3.4 H-4); ^{13}C NMR (100 MHz, $CDCl_3$): δ_C 20.49, 20.59, 20.63, 20.65 ($\underline{C}H_3CO$), 61.21 (C-6), 66.84, 68.06, 70.72, 72.87 (C-2,C-3, C-4, C-5), 88.29 (C-1), 169.32, 169.94, 170.07, 170.32 ($CH_3\underline{C}O$).

*2.8. 2,3,4,6-Tetra-O-acetyl-N-(β-D-glucopyranosyl)chloroacetamide **13a** and 2,3,4,6-tetra-O-acetyl-N-(β-D-galactopyranosyl)chloroacetamide **13b***

General procedure: To a solution of appropriate glycopyranosyl azide **12a** or **12b** (5.36 mmol, 2 g) in THF:EtOH 2:1 solvent system (40 mL) 20 % $Pd(OH)_2$ on carbon (120 mg) was added. The reaction was conducted in Paar hydrogenation apparatus at 1.5 bar hydrogen pressure for 2 h at room temperature. The reaction mixture was filtered through a plug of celite and condensed. The crude glycopyranosyl amines, without any additional purification step, were dissolved in dry CH_2Cl_2 (20 mL) and to such solutions of 2,3,4,6-tetra-O-acetyl-β-D-glucopyranosyl amine or 2,3,4,6-tetra-O-acetyl-β-D-galactopyranosyl amine triethylamine (2.2 mL) was added. The reaction mixture was cooled to 0 °C and chloroacetyl chloride was added dropwise (670 μl, 8.4 mmol) then stirring was continued at room temperature. After 1 h, the resulting mixture was diluted with dichloromethane (100 mL) and washed with brine (2 x 60 mL). The organic layer was dried over anhydrous $MgSO_4$, concentrated in vacuo and purified by column chromatography (toluene:AcOEt, gradient 8:1 to 2:1).

Product **13a** was obtained as a white solid (1.54 g, 68% yield); m.p.: 160-163 °C; $[\alpha]^{24}_{D}$ = 8.3 (c 1.0, $CHCl_3$); HRMS (ESI$^+$): calcd for $C_{16}H_{22}ClNO_{10}Na$ ($[M+Na]^+$): m/z 446.0830; found: m/z 446.0834; ^1H NMR (400 MHz, $CDCl_3$): δ_H 2.03, 2.04, 2.06, 2.09 (4s, 12H, CH_3CO), 3.84 (ddd, 1H, J_1 2.1 Hz, J_2 4.4 Hz, J_3 10.1 Hz, H-5), 4.00 and 4.07 (qAB, 2H, J 15.4 Hz, CH_2Cl), 4.10 (dd, 1H, J_1 2.1 Hz, J_2 12.5 Hz, H-6a), 4.31 (dd, 1H, J_1 4.4 Hz, J_2 12.5 Hz, H-6b), 5.01 (dd, 1H, J_1 9.4 Hz, J_2 10.0 Hz, H-1), 5.09 (dd, 1H, J_1 9.4 Hz, J_2 10.1 Hz, H-4), 5.21 (dd, 1H, J_1 9.0 Hz, J_2 9.4 Hz, H-3), 5.33 (dd, 1H, J_1 9.0 Hz, J_2 9.4 Hz, H-2), 7.29 (d, 1H, J 9.0 Hz, CONH); ^{13}C NMR (100 MHz, $CDCl_3$): δ_C 20.57, 20.61, 20.72, 20.76 ($\underline{C}H_3CO$), 42.25 ($\underline{C}H_2Cl$), 61.56 (C-6), 68.09, 70.26, 72.55, 73.83, 78.54 (C-2, C-3, C-4, C-5, C-1), 166.81, 169.49, 169.87, 170.58, 170.79 (CO).

Product **13b** was obtained as a white solid (1.81 g, 80% yield); m.p.: 143-144 °C; $[\alpha]^{25}_D$ = 21.3 (c 1.0, $CHCl_3$); HRMS (ESI$^+$): calcd for $C_{16}H_{22}ClNO_{10}Na$ ([M+Na]$^+$): m/z 446.0830; found: m/z 446.0832; ^{1}H NMR (400 MHz, $CDCl_3$): δ_H 2.01, 2.04, 2.07, 2.16 (4s, 12H, CH_3CO), 4.01 and 4.08 (qAB, 2H, *J* 15.5 Hz, CH_2Cl), 4.04-4.18 (m, 3H, H-5, H-6a, H-6b), 5.11-5.24 (m, 3H, H-1, H-2, H-3), 5.45 (dd, 1H, J_1 0.7 Hz, J_2 3.0 Hz, H-4), 7.32 (d, 1H, *J* 6.9 Hz, CONH); ^{13}C NMR (100 MHz, $CDCl_3$): δ_C 20.54, 20.61, 20.67, 20.71 ($\underline{C}H_3CO$), 42.26 (CH_2Cl), 61.12 (C-6), 67.10, 67.97, 70.72, 72.58, 78.84 (C-2, C-3, C-4, C-5, C-1), 166.71, 169.76, 170.01, 170.34, 171.06 (CO).

*2.9. 2,3,4,6-Tetra-O-acetyl-N-(β-D-glucopyranosyl)azidoacetamide **14a** and 2,3,4,6-tetra-O-acetyl-N-(β-D-galactopyranosyl)azidoacetamide **14b***

General procedure: To a solution of 2,3,4,6-tetra-O-acetyl-N-(β-D-glucopyranosyl)chloro-acetamide **13a** or 2,3,4,6-tetra-O-acetyl-N-(β-D-galactopyranosyl) chloroacetamide **13b** (1.0 g, 2.36 mmol) in dry DMF (15 mL), sodium azide (753.0 mg, 11.59 mmol) was added. The reaction mixture was stirred at room temperature for 24 h. After completion reaction, the solvent was evaporated in vacuo, the residue was diluted with ethyl acetate (50 mL) and extracted with water (30 mL), 0.25 M HCl water solution (20 mL) and brine (20 mL). The organic layer was dried over anhydrous $MgSO_4$, concentrated in vacuo and the residues were purified by column chromatography (toluene:AcOEt, gradient 10:1 to 2:1).

Product **14a** was obtained as a white solid (975.0 mg, 96% yield); m.p.: 150-153 °C; $[\alpha]^{24}_D$ = 12.4 (c 1.0, $CHCl_3$); HRMS (ESI$^+$): calcd for $C_{16}H_{22}N_4O_{10}Na$ ([M+Na]$^+$): *m/z* 453.1234; found: *m/z* 453.1236; ^{1}H NMR (400 MHz, $CDCl_3$): δ_H 2.03, 2.04, 2.06, 2.09 (4s, 12H, CH_3CO), 3.83 (ddd, 1H, J_1 2.1 Hz, J_2 4.4 Hz, J_3 10.1 Hz, H-5), 3.95 and 4.00 (qAB, 2H, *J* 16.9 Hz, CH_2N), 4.09 (dd, 1H, J_1 2.1 Hz, J_2 12.5 Hz, H-6a), 4.30 (dd, 1H, J_1 4.4 Hz, J_2 12.5 Hz, H-6b), 4.98 (dd, 1H, J_1 9.4 Hz, J_2 9.8 Hz, H-1), 5.08 (dd, 1H, J_1 9.4 Hz, J_2 10.1 Hz, H-4), 5.22 (dd, 1H, J_1 9.0 Hz, J_2 9.4 Hz, H-3), 5.32 (dd, 1H, J_1 9.0 Hz, J_2 9.8 Hz, H-2), 7.10 (d, 1H, *J* 9.1 Hz, CONH); ^{13}C NMR (100 MHz, $CDCl_3$): δ_C 20.57, 20.61, 20.72, 20.75 ($\underline{C}H_3CO$), 52.59 (CH_2N_3), 61.56 (C-6), 68.08, 70.45, 72.57, 73.78, 78.15 (C-2, C-3, C-4, C-5, C-1), 167.41, 169.50, 169.86, 170.57, 170.88 (CO).

Product **14b** was obtained as a white solid (874.0 mg, 86% yield); m.p.: 60-63 °C; $[\alpha]^{24}_D$ = 25.4 (c 1.0, $CHCl_3$); HRMS (ESI$^+$): calcd for $C_{16}H_{22}N_4O_{10}Na$ ([M+Na]$^+$): *m/z* 453.1234; found: *m/z* 453.1227; ^{1}H NMR (400 MHz, $CDCl_3$): δ_H 2.01, 2.04, 2.07, 2.16 (4s, 12H, CH_3CO), 3.96 and 4.01 (qAB, 2H, *J* 16.8 Hz, CH_2N), 4.02-4.17 (m, 3H, H-5, H-6a, H-6b), 5.11-5.25 (m, 3H, H-1, H-2, H-3), 5.45 (dd, 1H, J_1 1.1 Hz, J_2 2.9 Hz, H-4), 7.13 (d, 1H, *J* 8.5 Hz, CONH); ^{13}C NMR (100 MHz, $CDCl_3$): δ_C 20.54, 20.60, 20.67, 20.70 ($\underline{C}H_3CO$), 52.61 (CH_2N_3), 61.14 (C-6), 67.11, 68.17, 70.73, 72.52, 78.43 (C-2, C-3, C-4, C-5, C-1), 167.31, 169.76, 170.00, 170.35, 171.15 (CO).

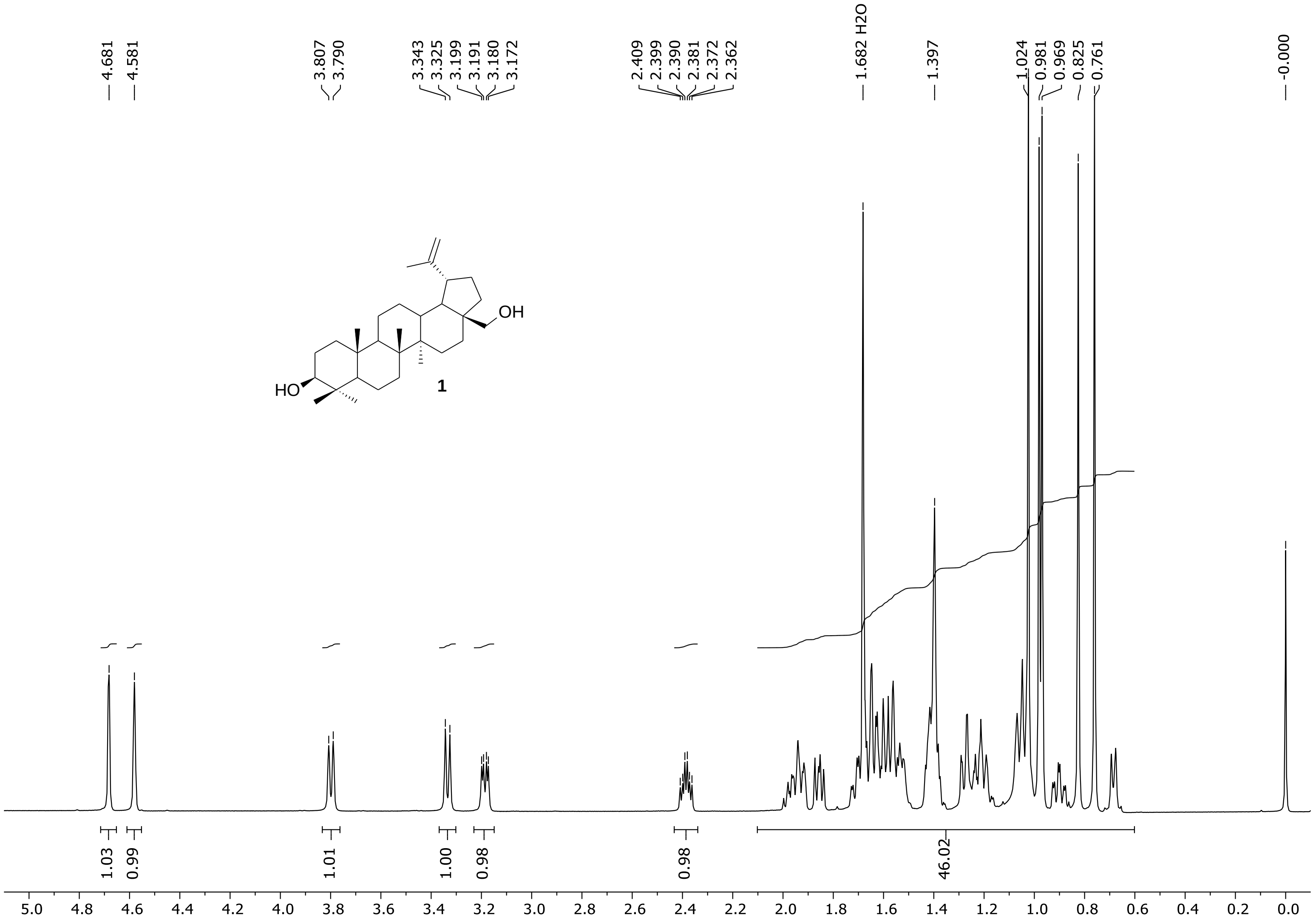

^{1}H NMR spectrum of betulin (**1**); 600 MHz/$CDCl_3$/TMS; δ (ppm).

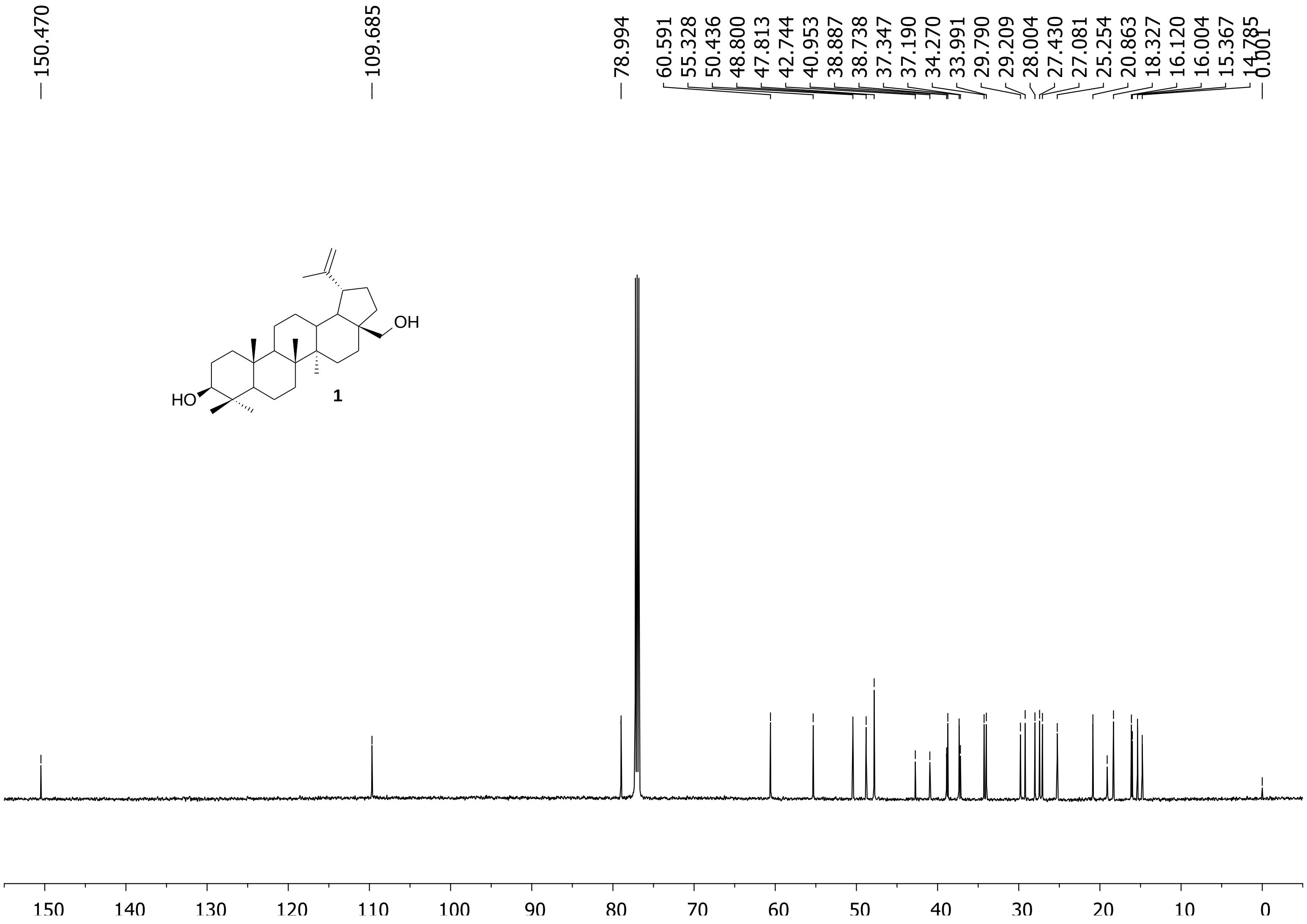

^{13}C NMR spectrum of betulin (**1**); 150 MHz/$CDCl_3$/TMS; δ (ppm).

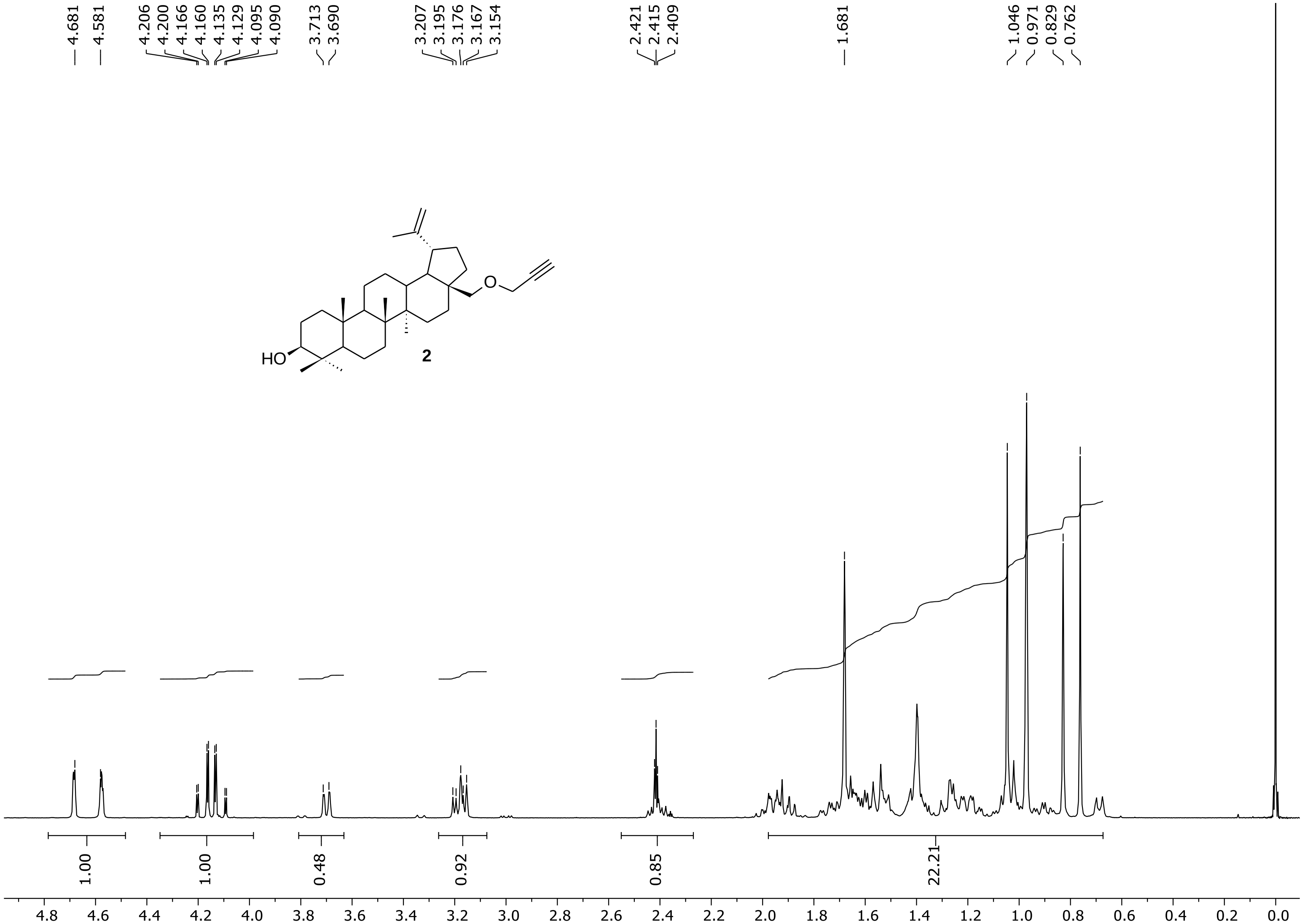

^{1}H NMR spectrum of 28-*O*-propargylbetulin (**2**); 400 MHz/$CDCl_3$/TMS; δ (ppm).

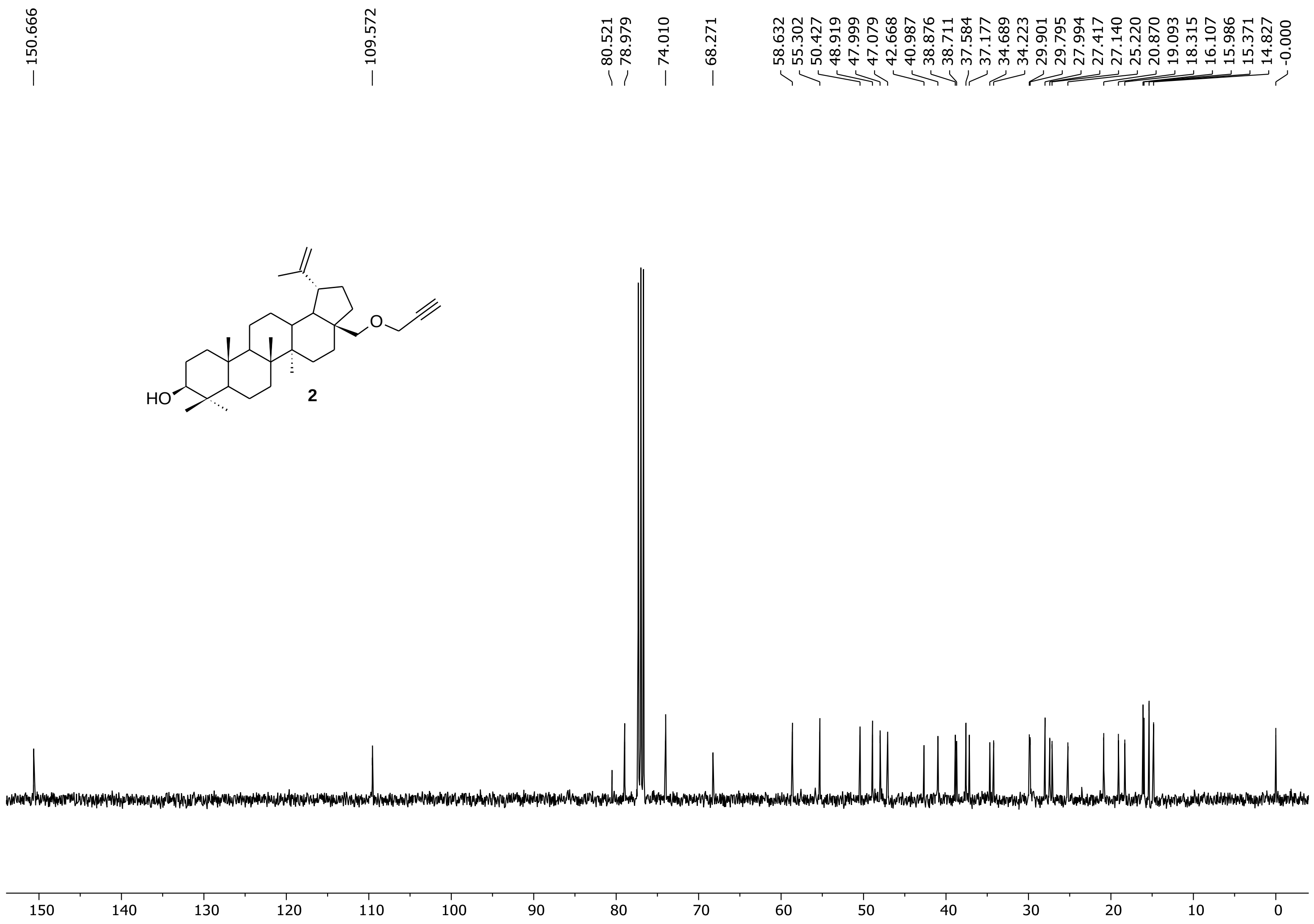

^{13}C NMR spectrum of 28-*O*-propargylbetulin (**2**); 150 MHz/$CDCl_3$/TMS; *δ* (ppm).

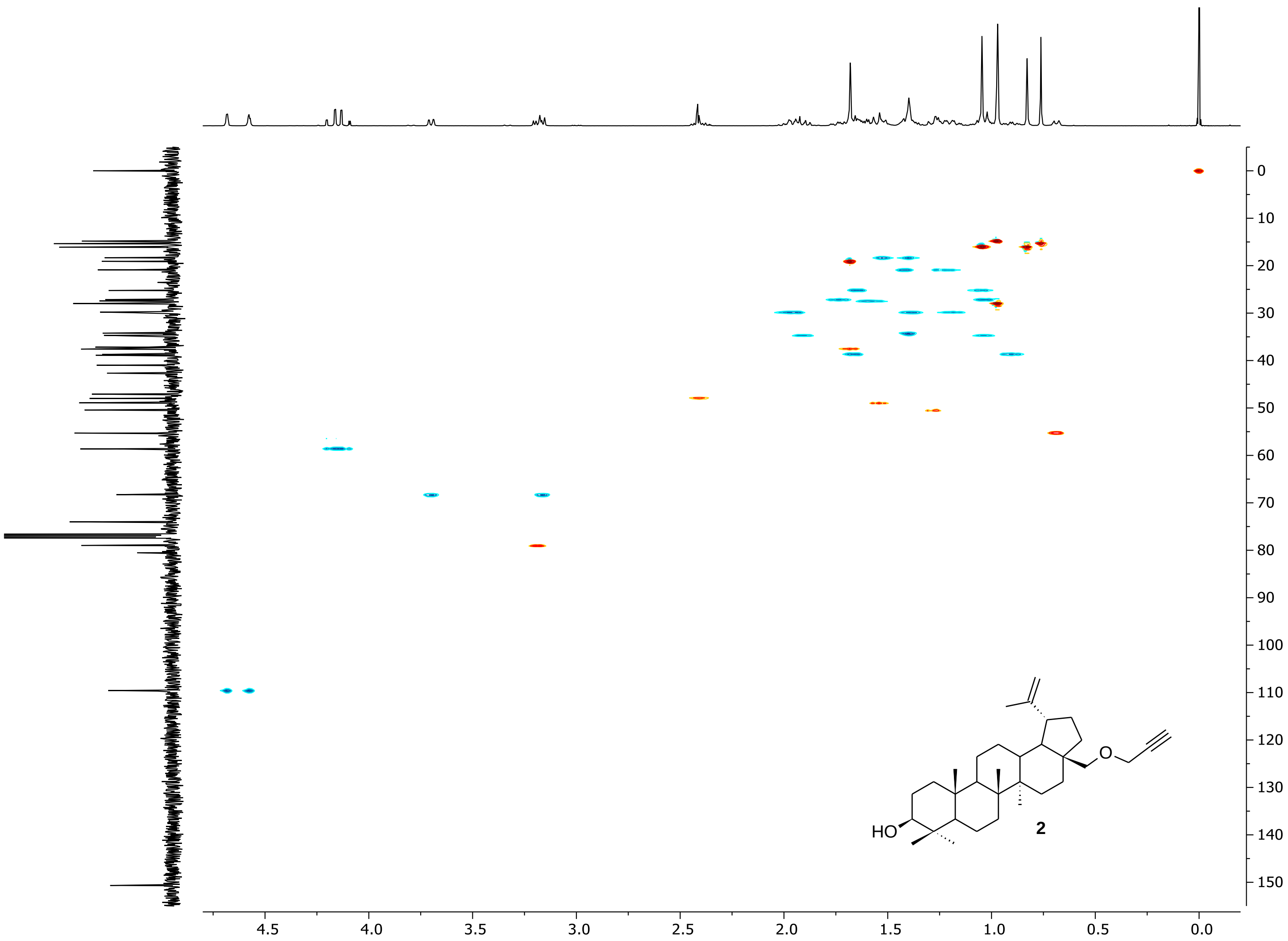

gHSQC spectrum of 28-*O*-propargylbetulin (**2**).

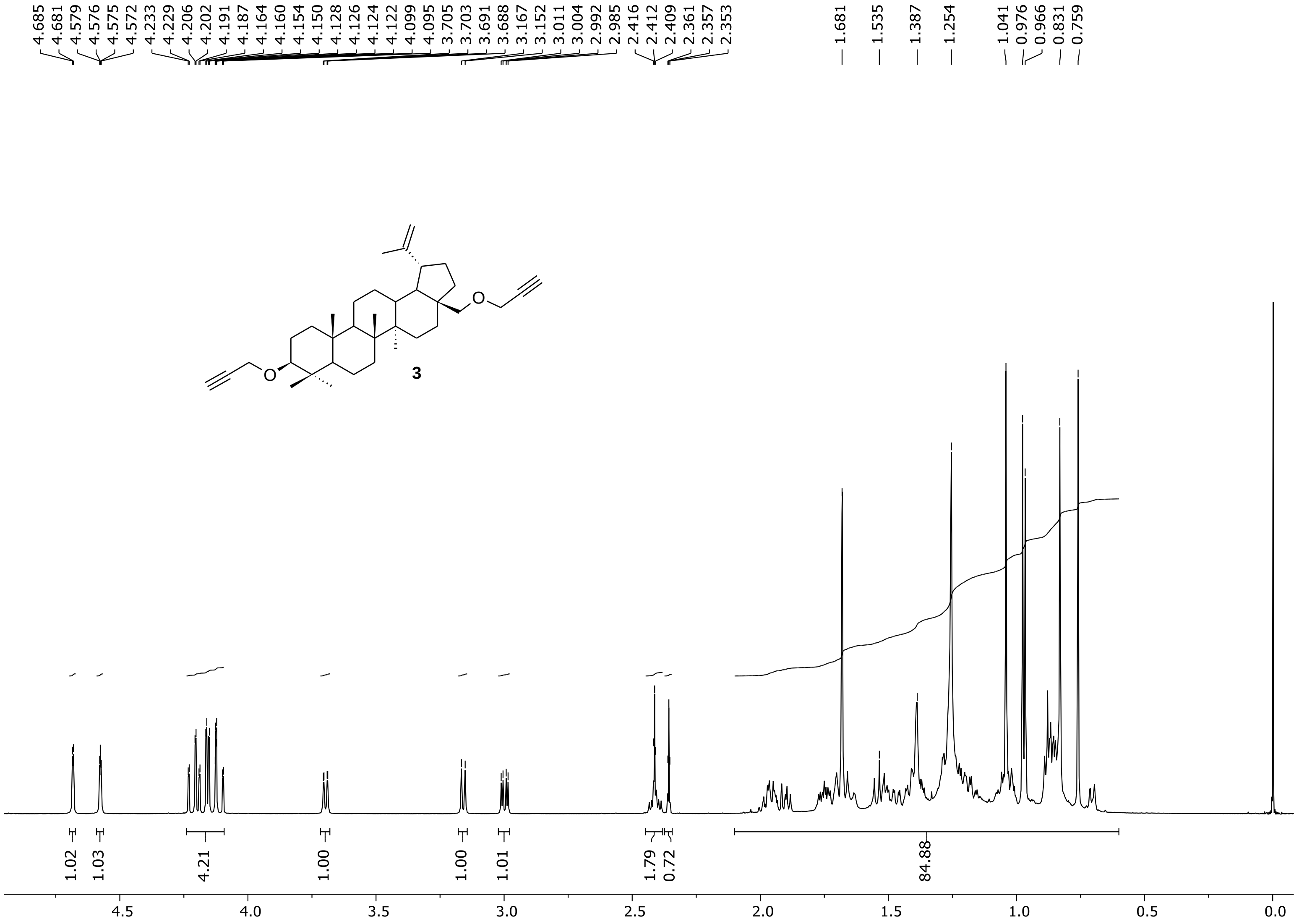

^{1}H NMR spectrum of 3,28-*O,O′*-dipropargylbetulin (**3**); 600 MHz/$CDCl_3$/TMS; *δ* (ppm).

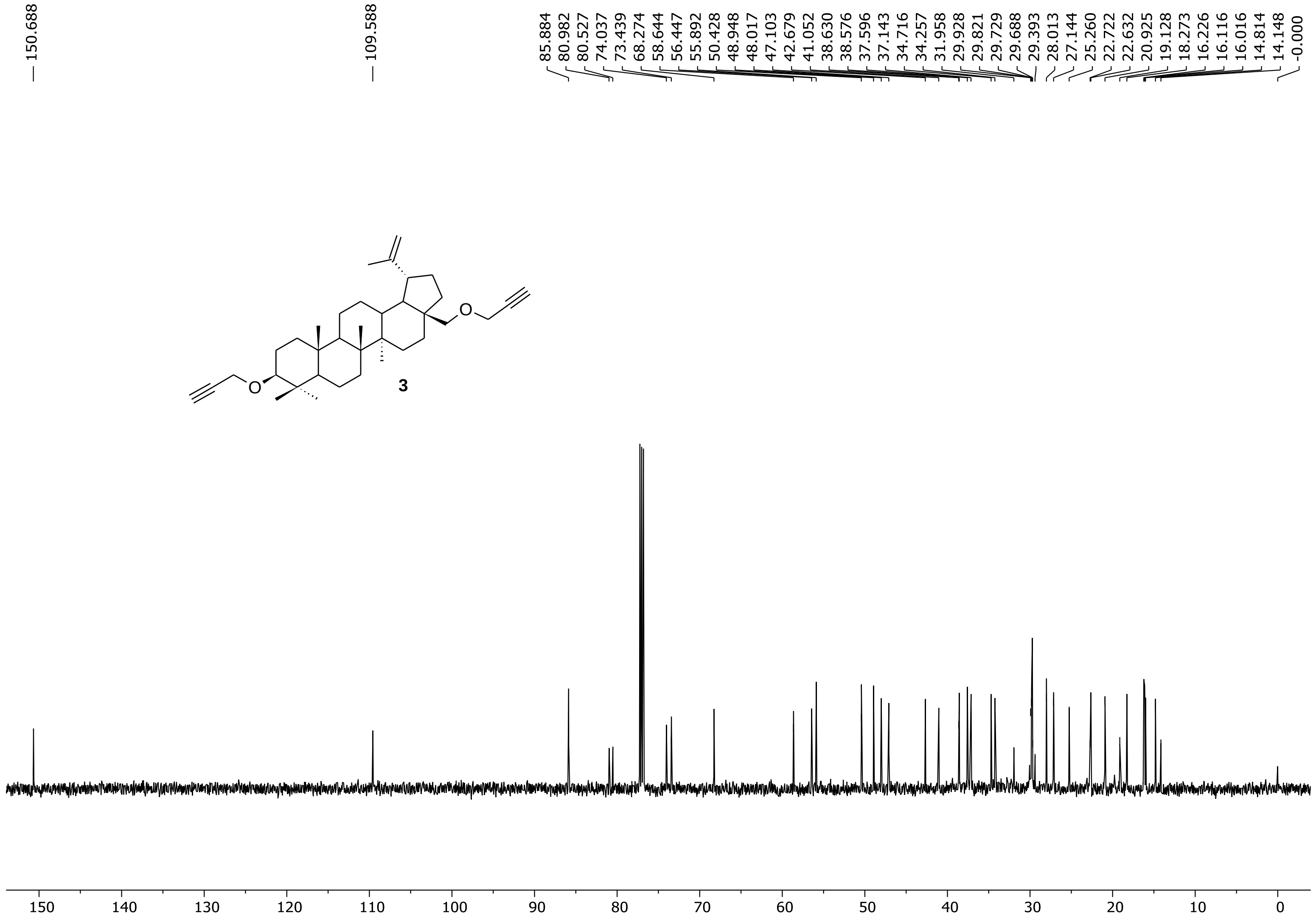

^{13}C NMR spectrum of 3,28-*O,O′*-dipropargylbetulin (**3**); 150 MHz/$CDCl_3$/TMS; *δ* (ppm).

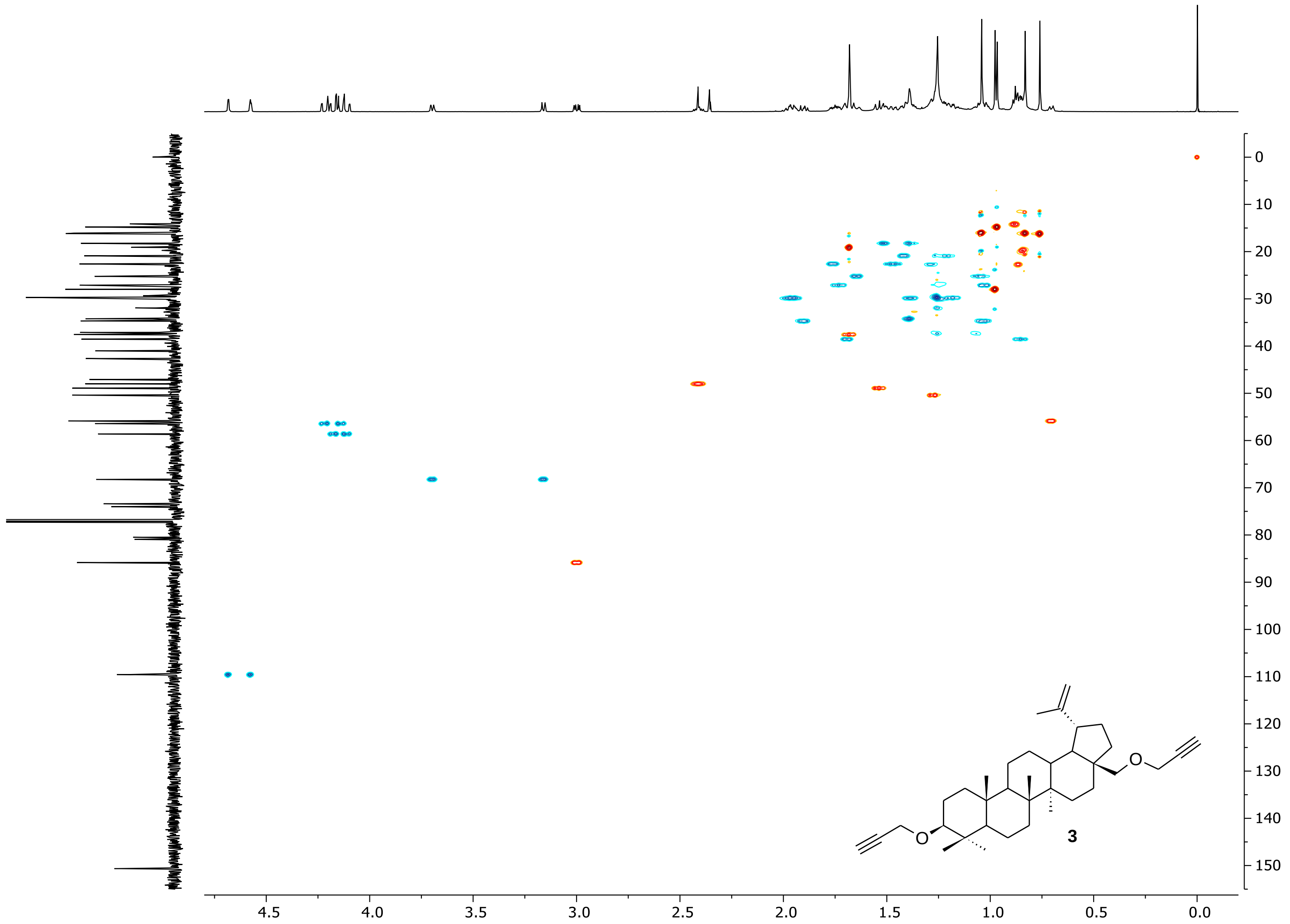

gHSQC spectrum of 3,28-*O,O′*-dipropargylbetulin (**3**).

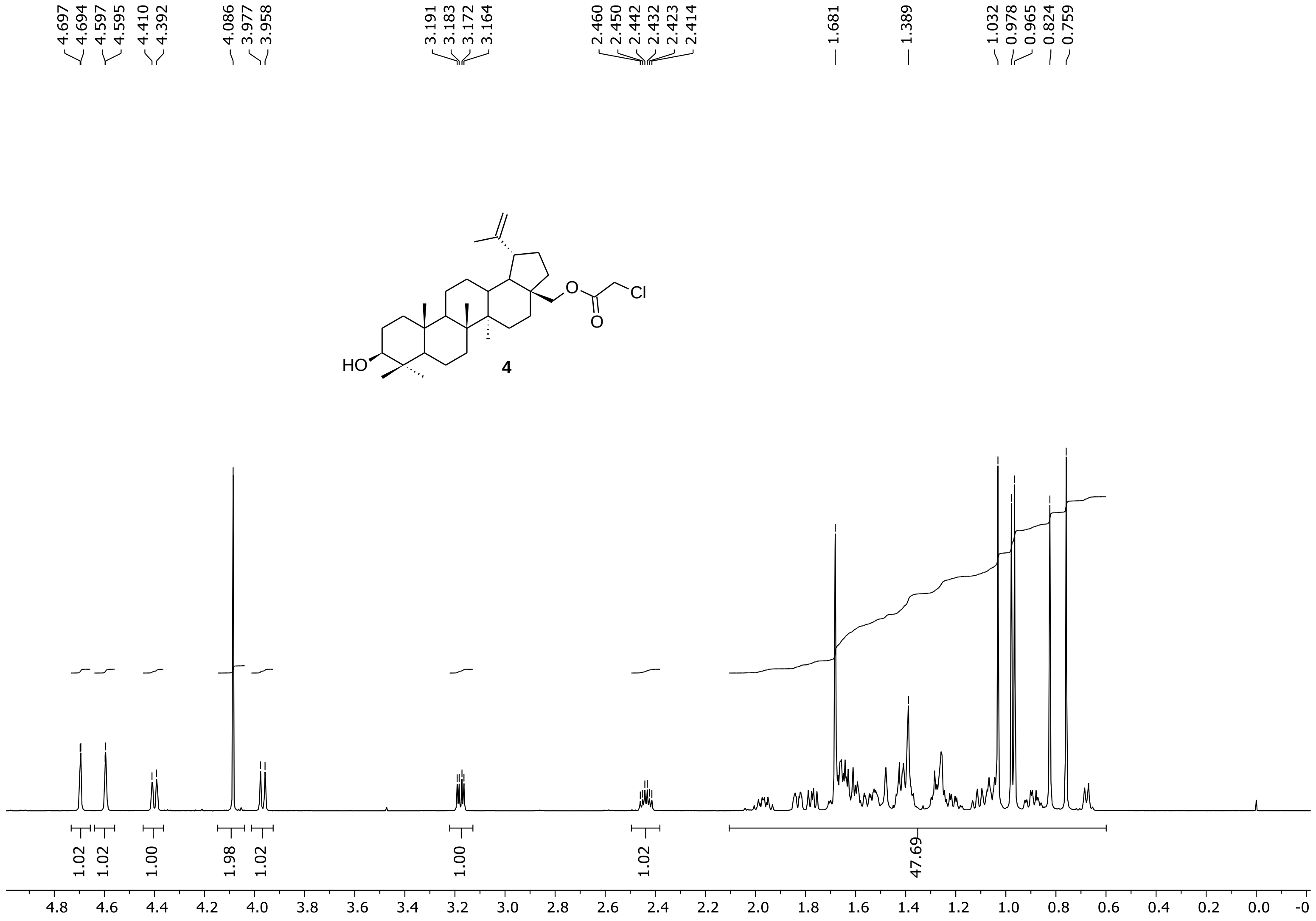

^{1}H NMR spectrum of 28-*O*-(2-chloroacetyl)betulin (**4**); 600 MHz/$CDCl_3$/TMS; δ (ppm).

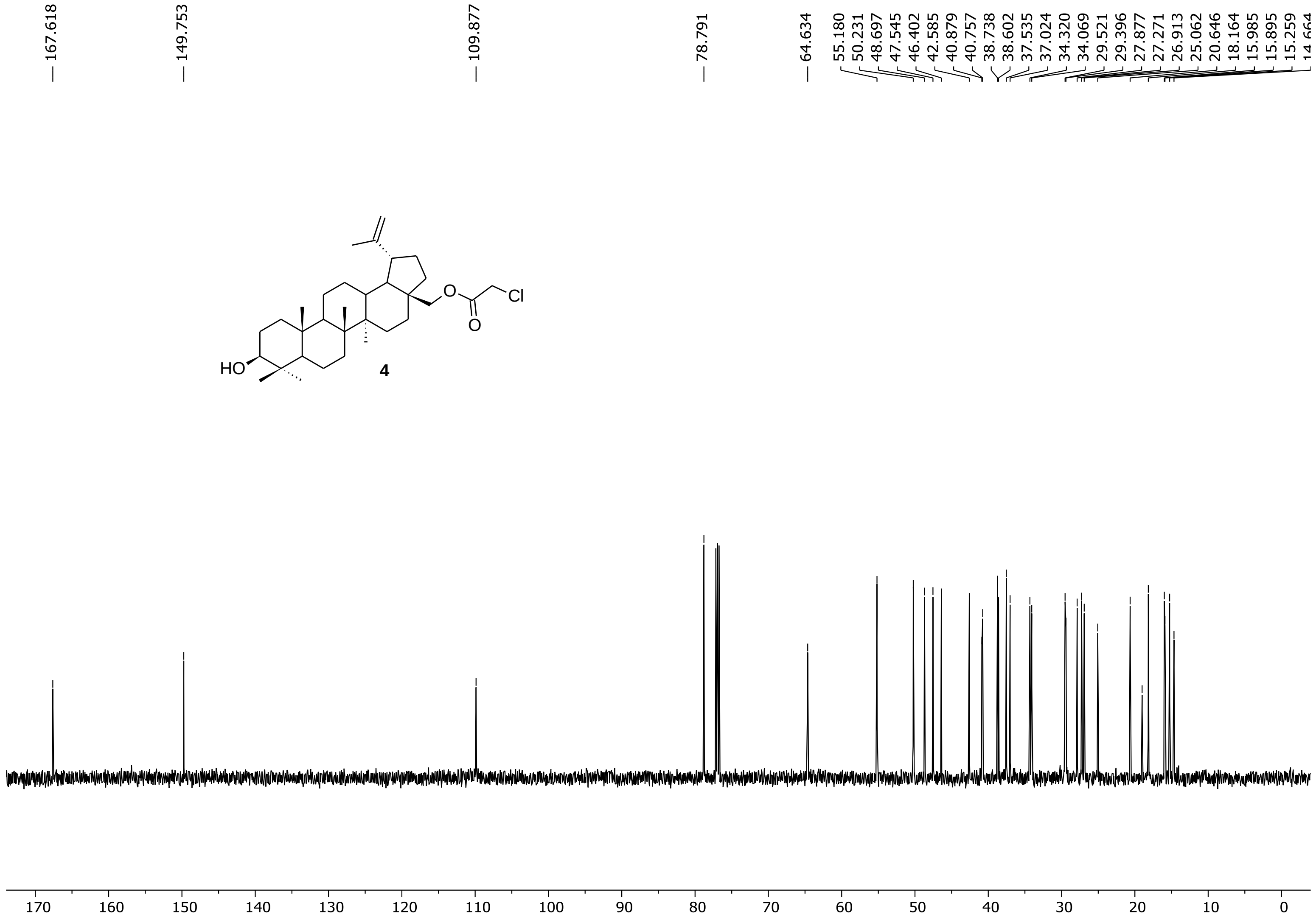

^{13}C NMR spectrum of 28-*O*-(2-chloroacetyl)betulin (**4**); 150 MHz/$CDCl_3$/TMS; δ (ppm).

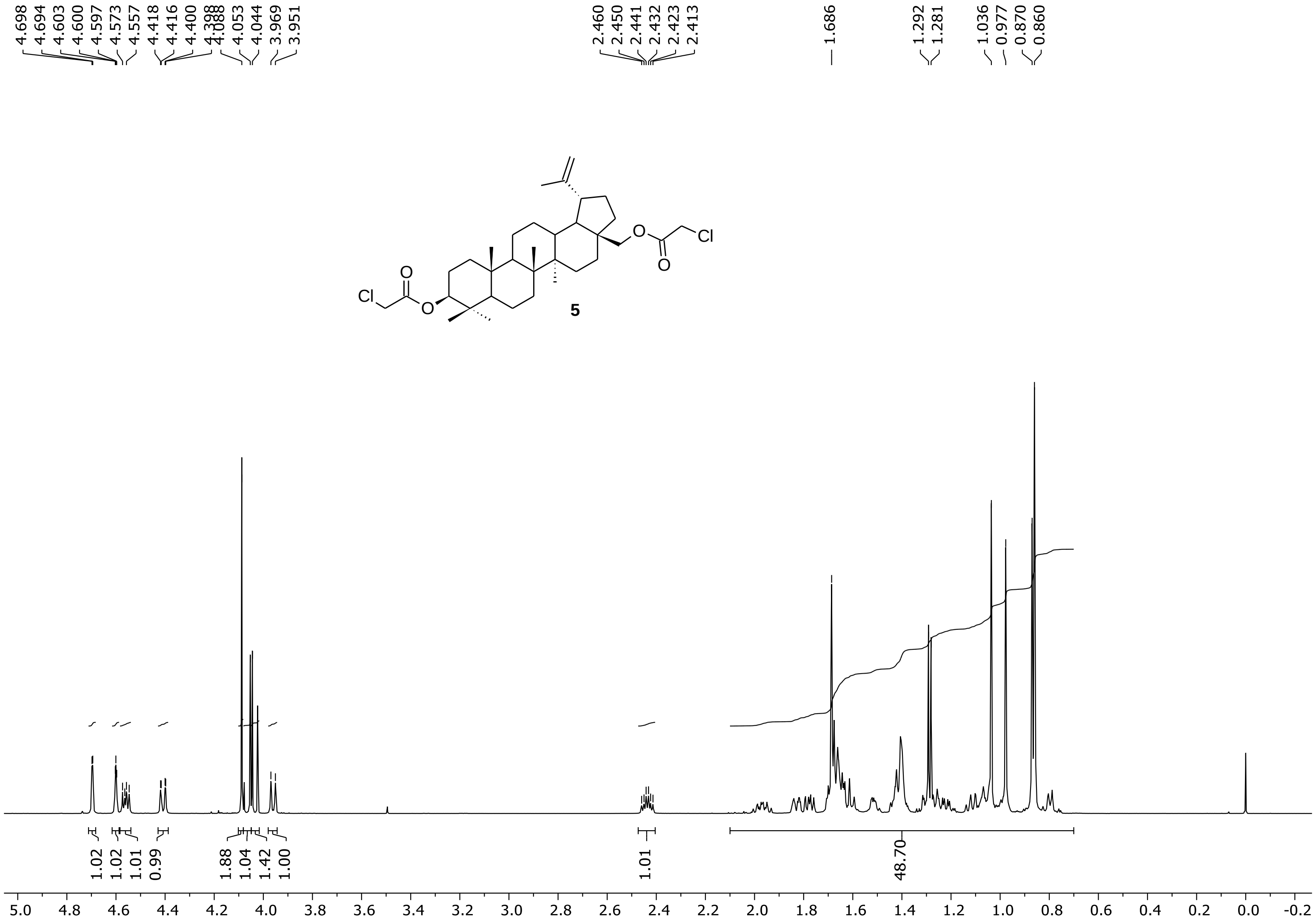

1H NMR spectrum of 3,28-*O,O′*-di(2-chloroacetyl)betulin (**5**); 600 MHz/CDCl3/TMS; δ (ppm).

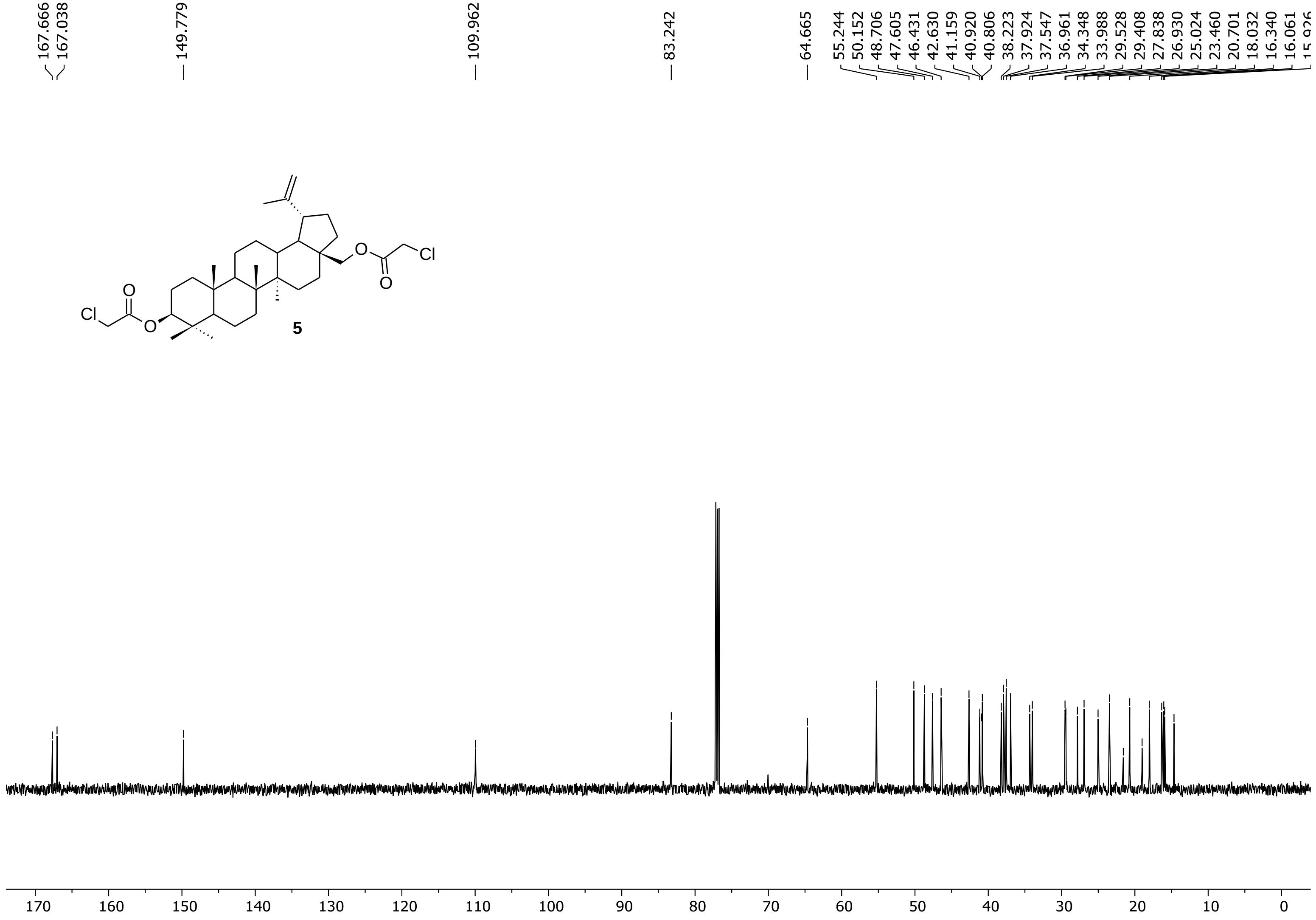

^{13}C NMR spectrum of 3,28-*O,O′*-di(2-chloroacetyl)betulin (**5**); 150 MHz/$CDCl_3$/TMS; δ (ppm).

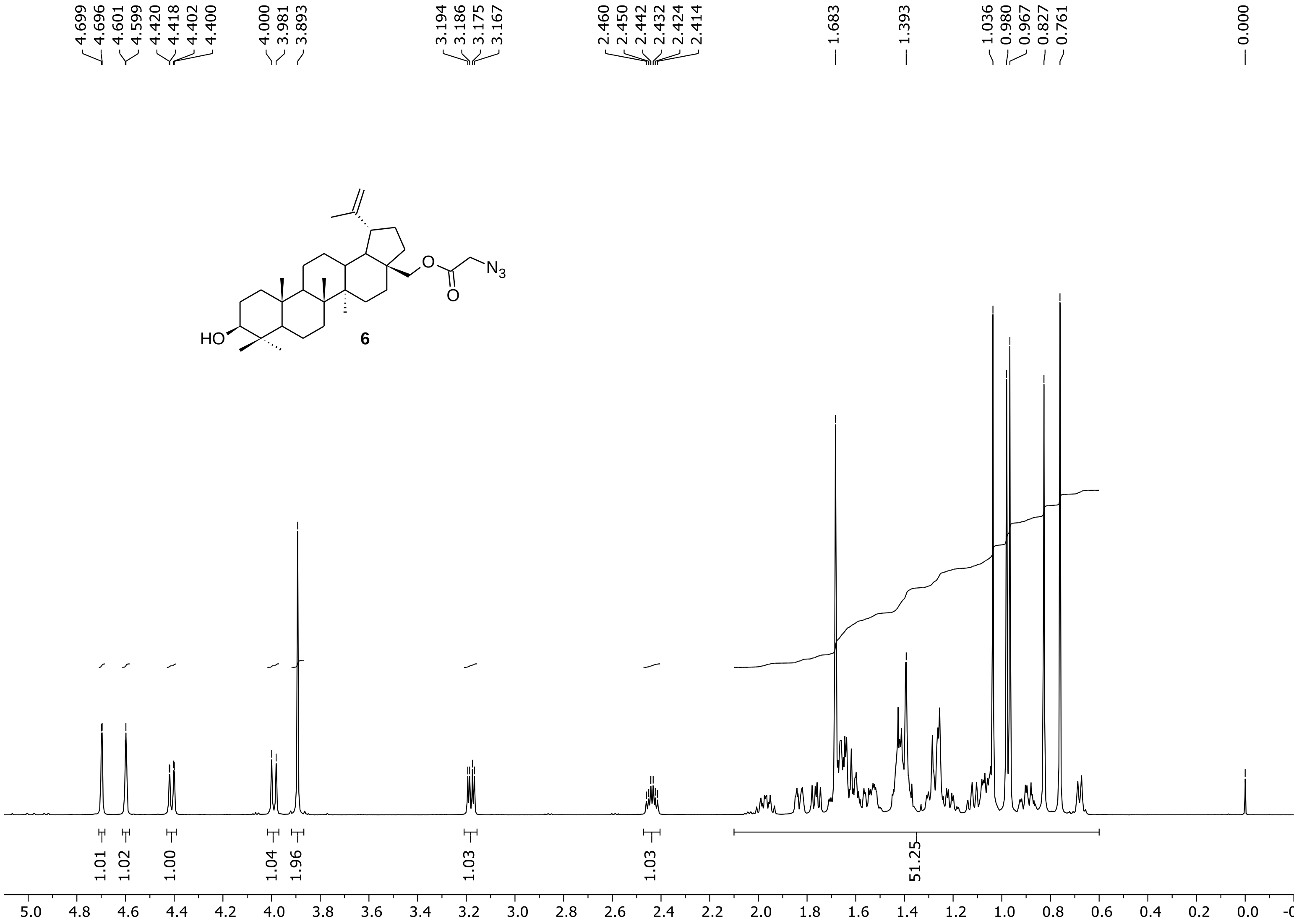

^{1}H NMR spectrum of 28-*O*-(2-azidoacetyl)betulin (**6**); 600 MHz/$CDCl_3$/TMS; δ (ppm).

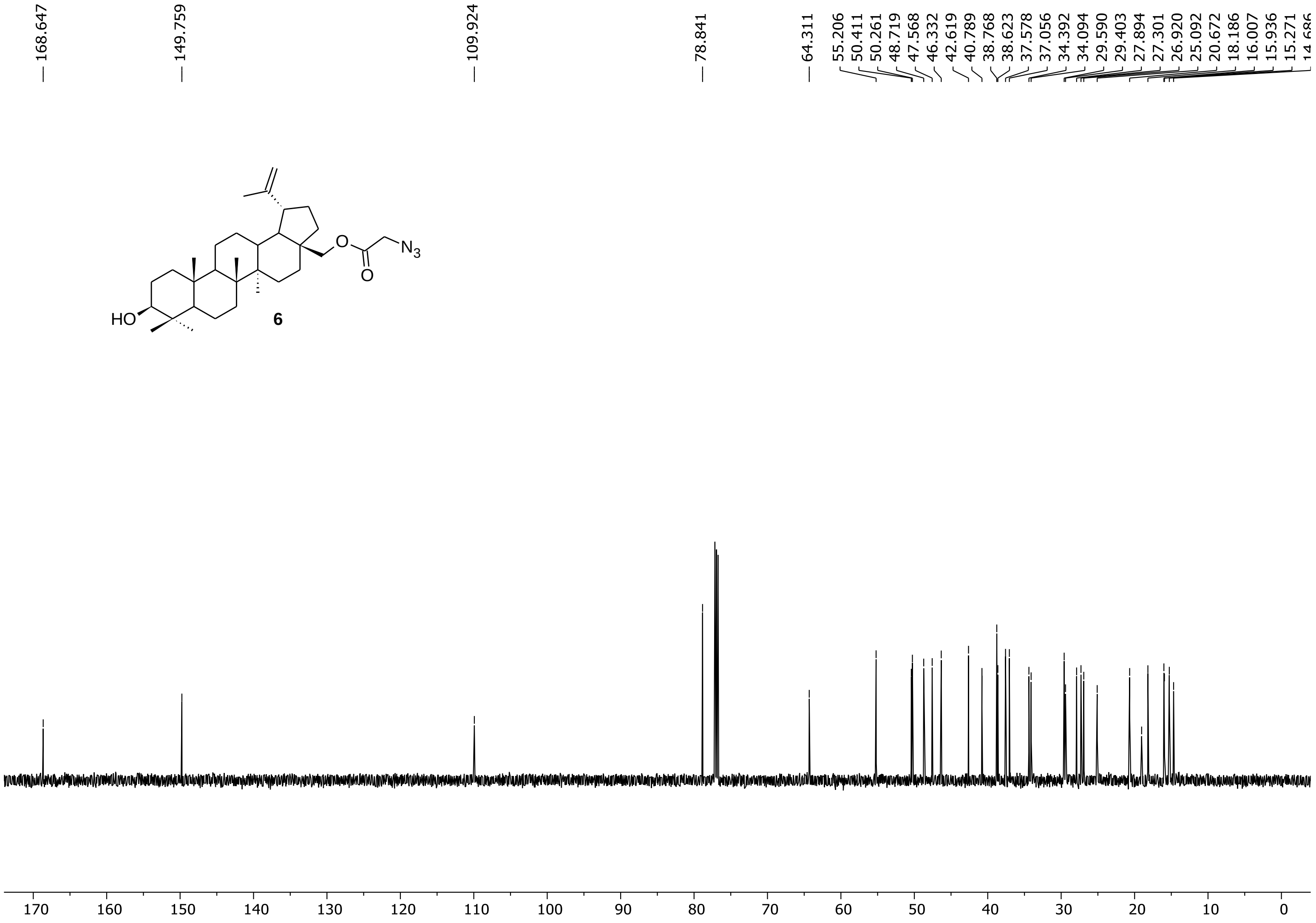

^{13}C NMR spectrum of 28-*O*-(2-azidoacetyl)betulin (**6**); 150 MHz/$CDCl_3$/TMS; δ (ppm).

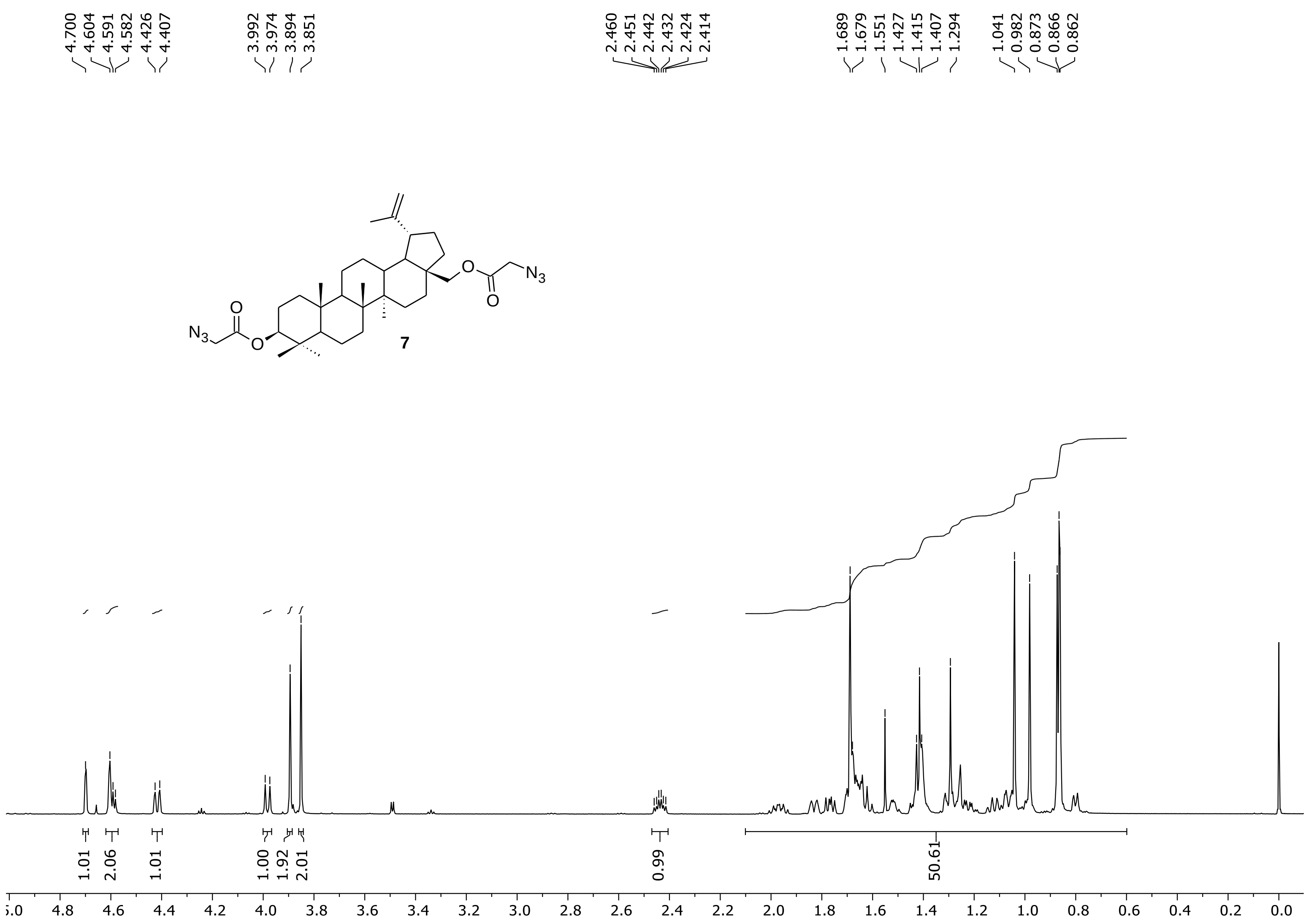

^{1}H NMR spectrum of 3,28-*O,O′*-di(2-azidoacetyl)betulin (**7**); 600 MHz/CDCl$_3$/TMS; δ (ppm).

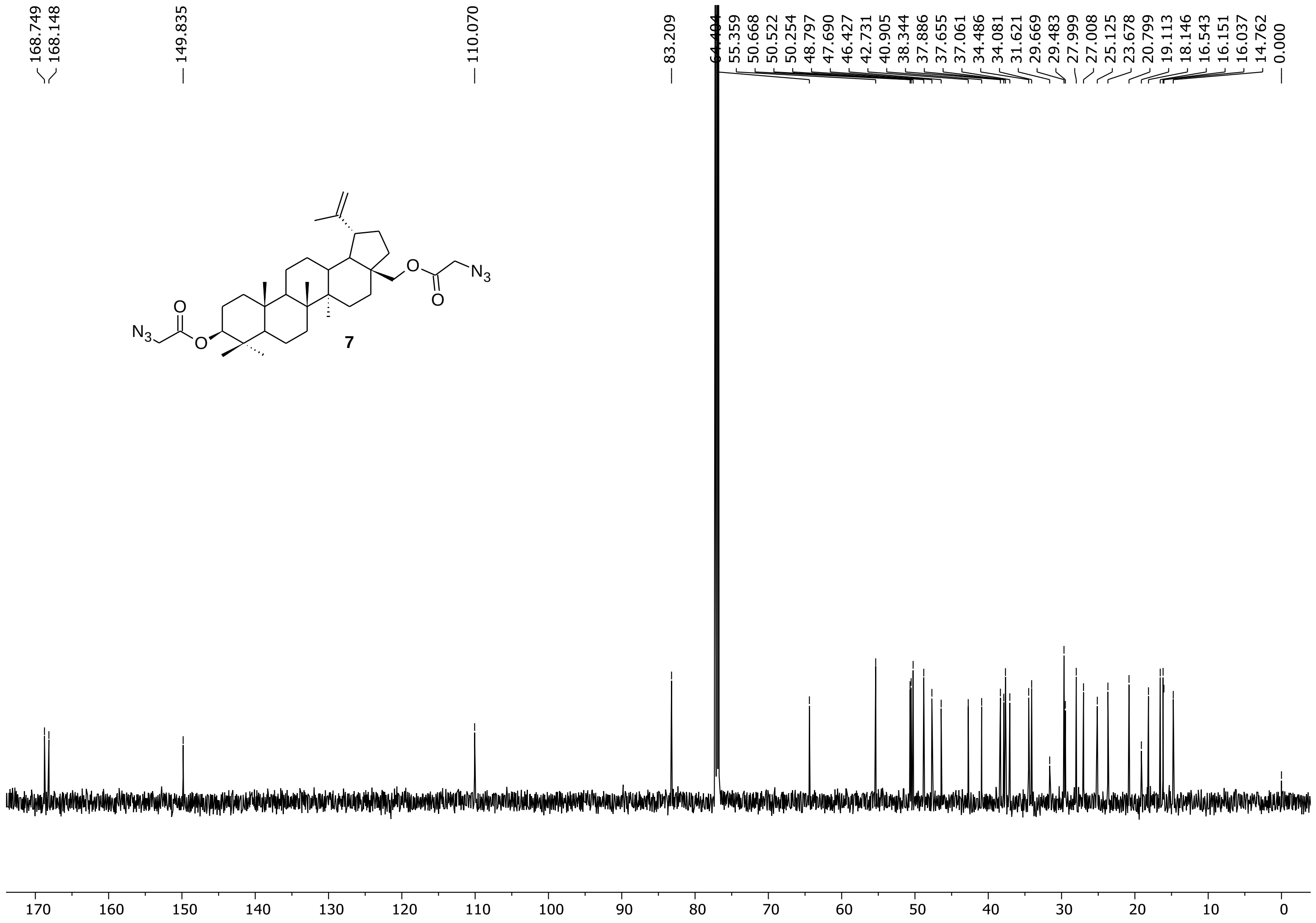

^{13}C NMR spectrum of 3,28-*O,O′*-di(2-azidoacetyl)betulin (**7**); 150 MHz/$CDCl_3$/TMS; *δ* (ppm).

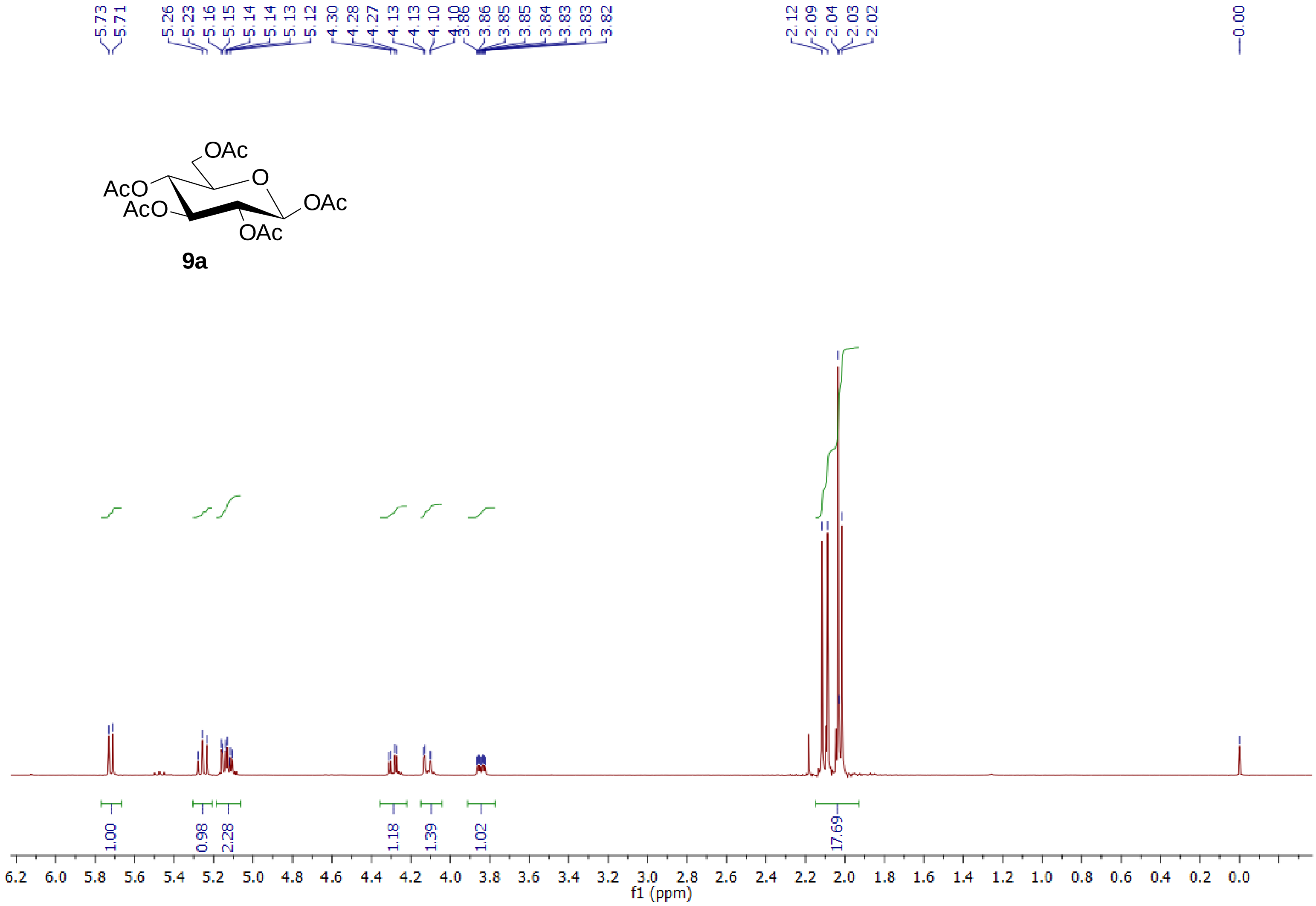

^{1}H NMR spectrum of 1,2,3,4,6-penta-*O*-acetyl-β-D-glucopyranose (**9a**); 400 MHz/$CDCl_3$/TMS; δ (ppm).

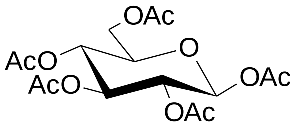

9a

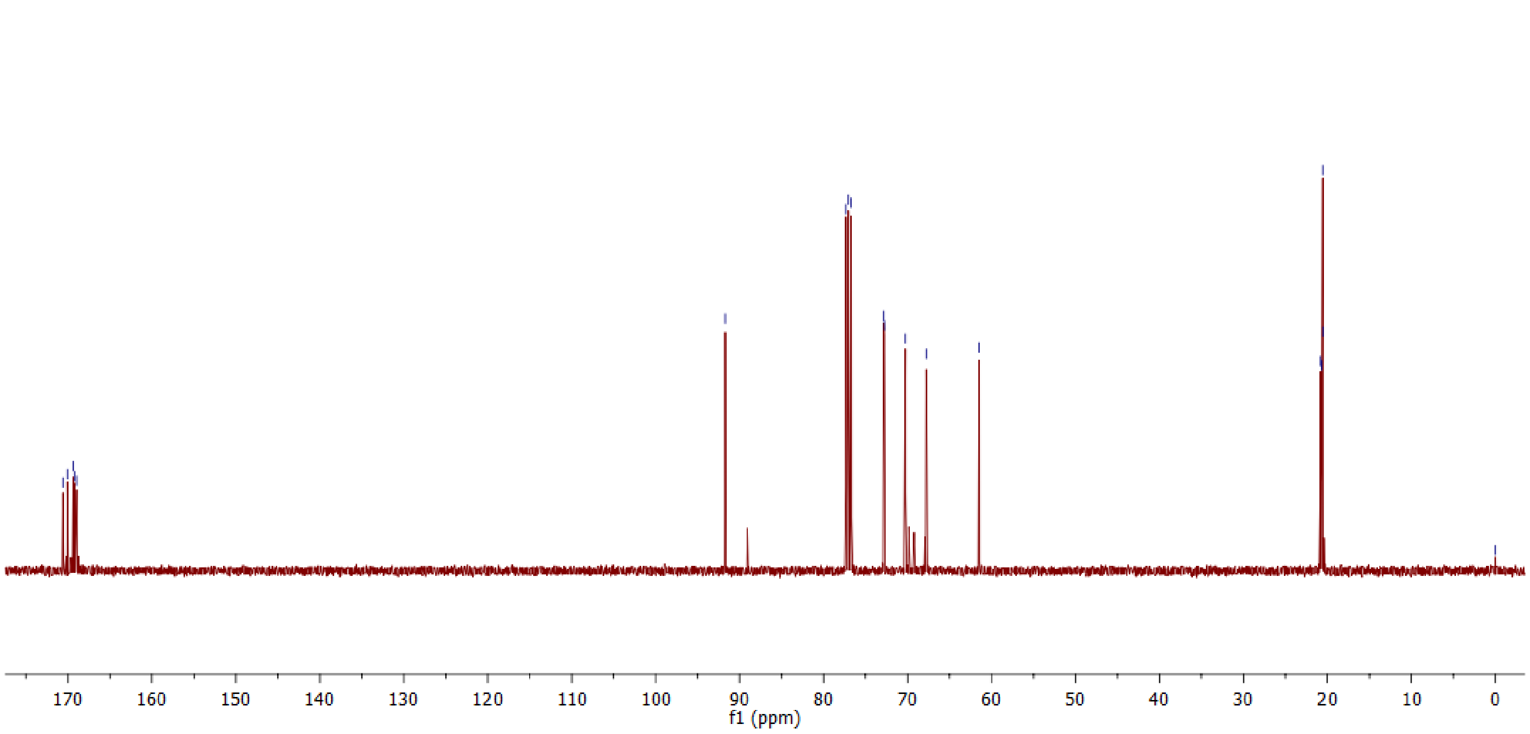

^{13}C NMR spectrum of 1,2,3,4,6-penta-*O*-acetyl-β-D-glucopyranose (**9a**); 100 MHz/$CDCl_3$/TMS; δ (ppm).

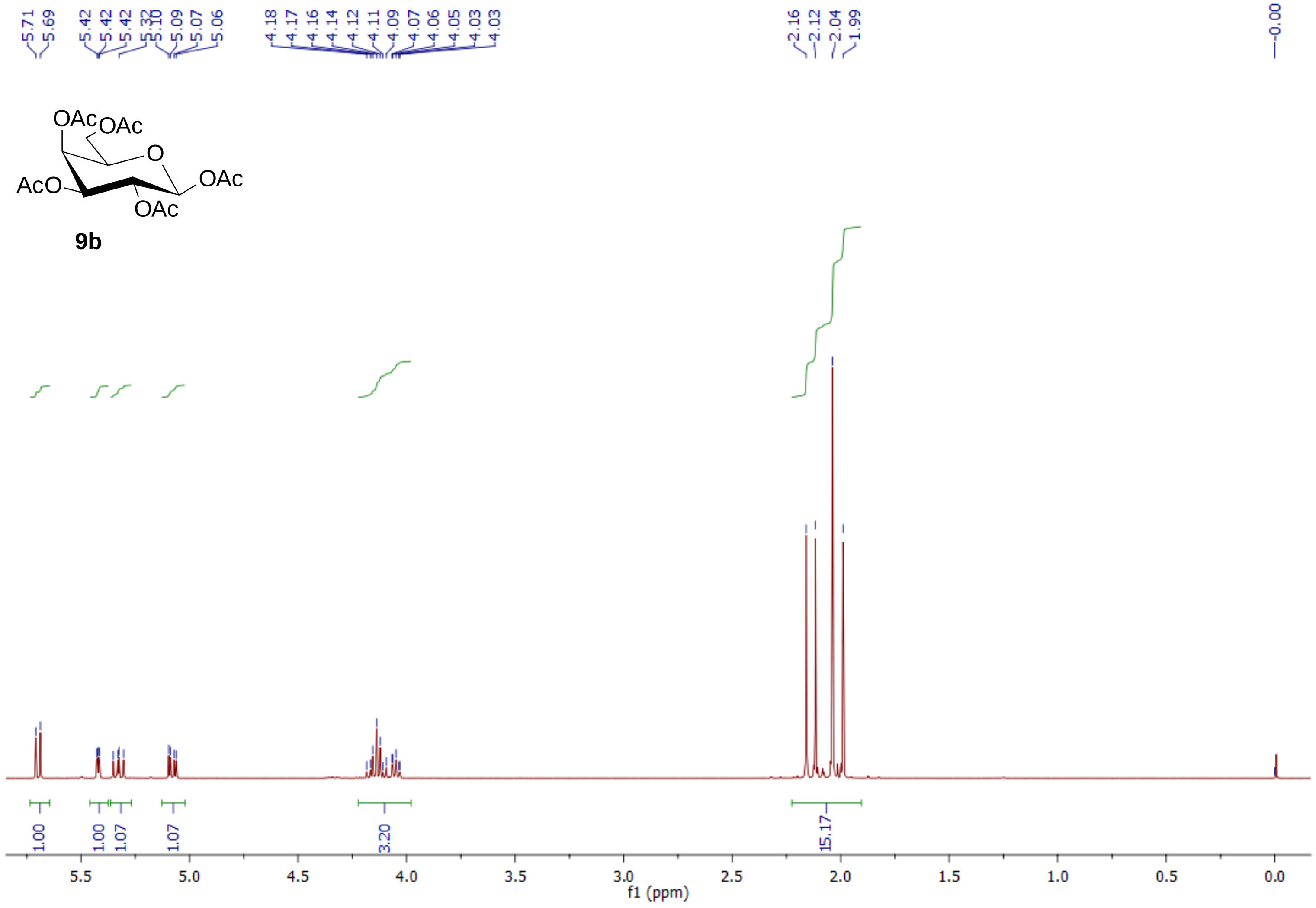

^{1}H NMR spectrum of 1,2,3,4,6-penta-*O*-acetyl-β-D-galactopyranose (**9b**); 400 MHz/$CDCl_3$/TMS; δ (ppm).

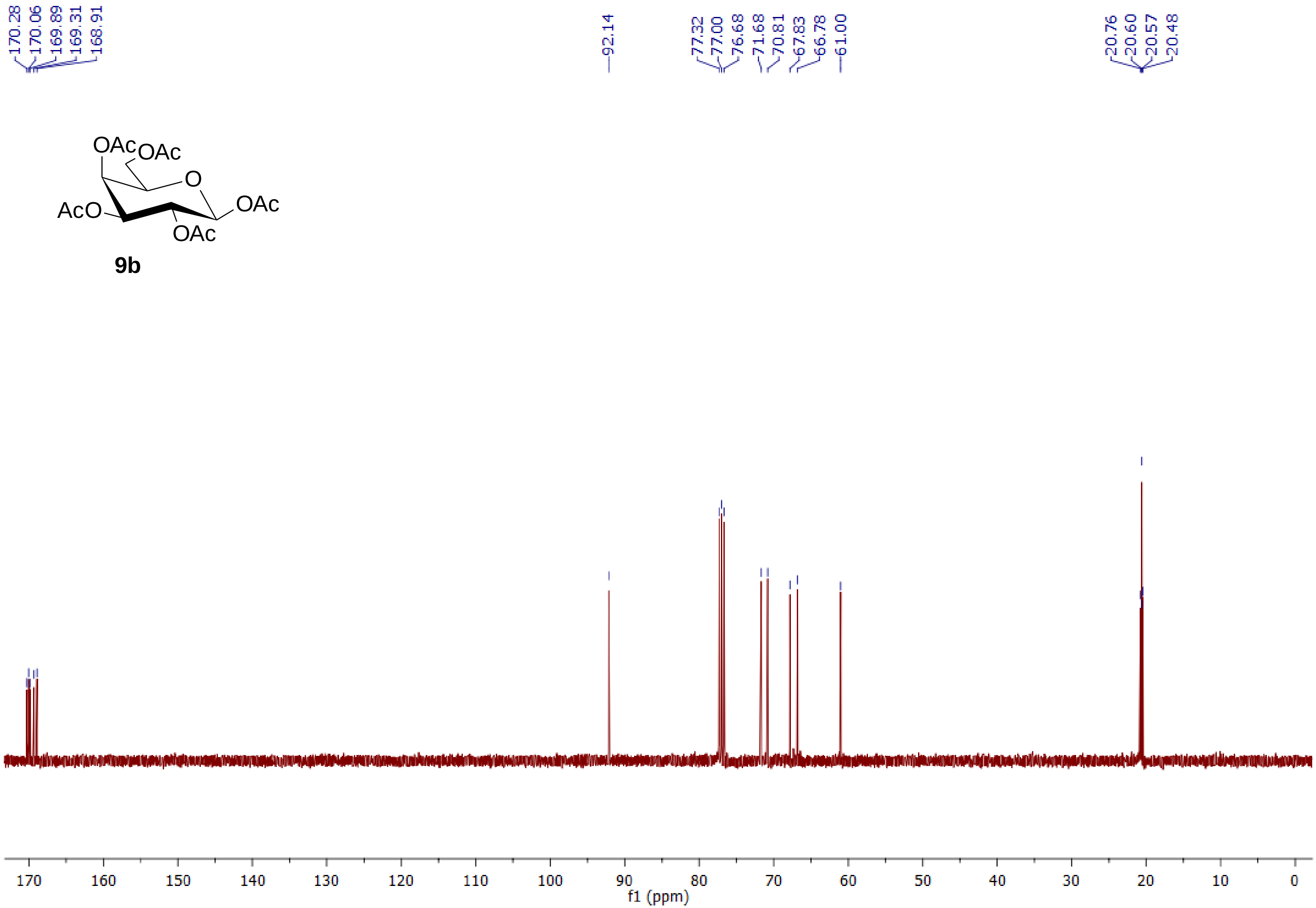

^{13}C NMR spectrum of 1,2,3,4,6-penta-*O*-acetyl-β-D-galactopyranose (**9b**); 100 MHz/$CDCl_3$/TMS; δ (ppm).

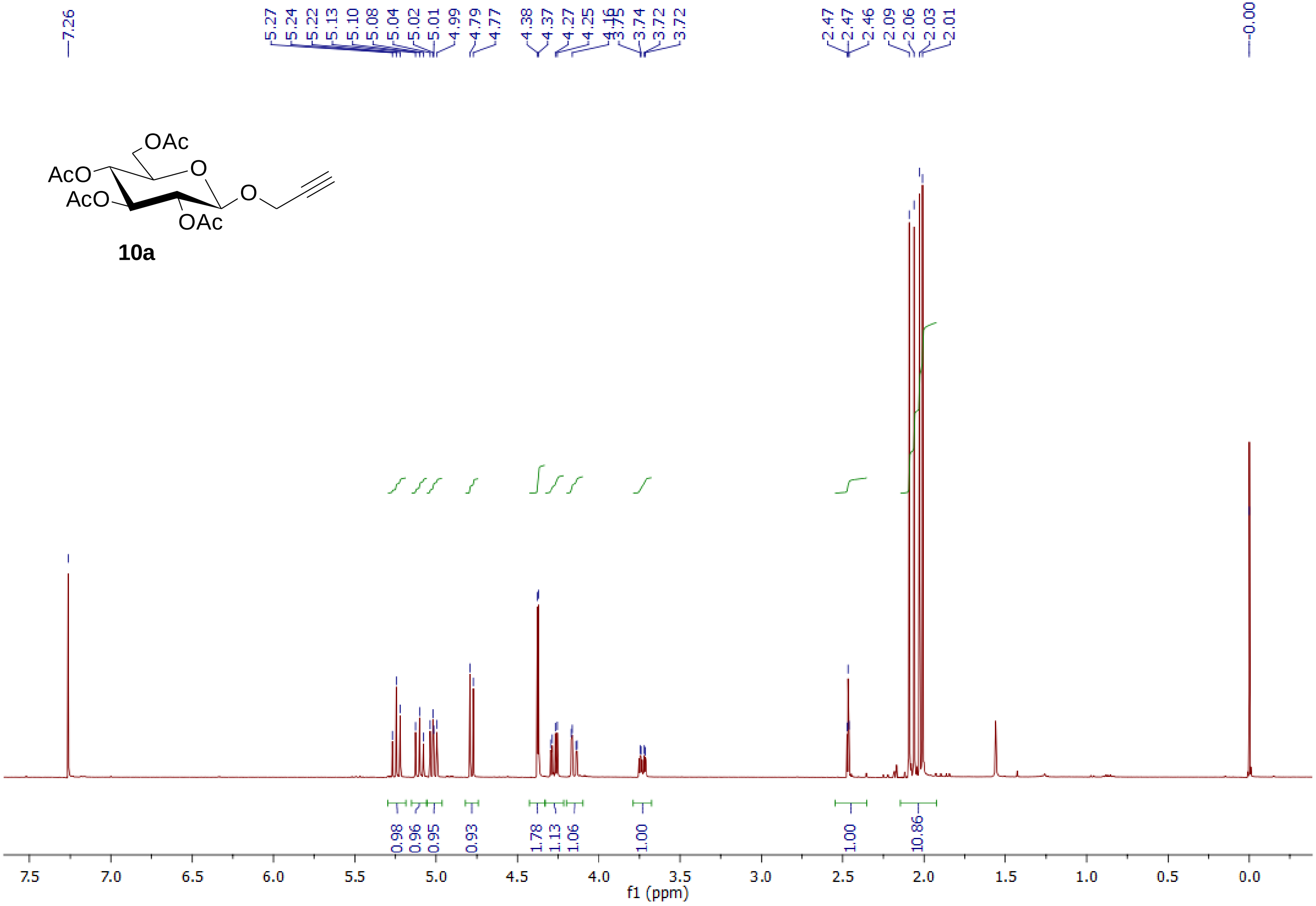

^{1}H NMR spectrum of propargyl 2,3,4,6-tetra-*O*-acetyl-β-D-glucopyranoside (**10a**); 400 MHz/$CDCl_3$/TMS; δ (ppm).

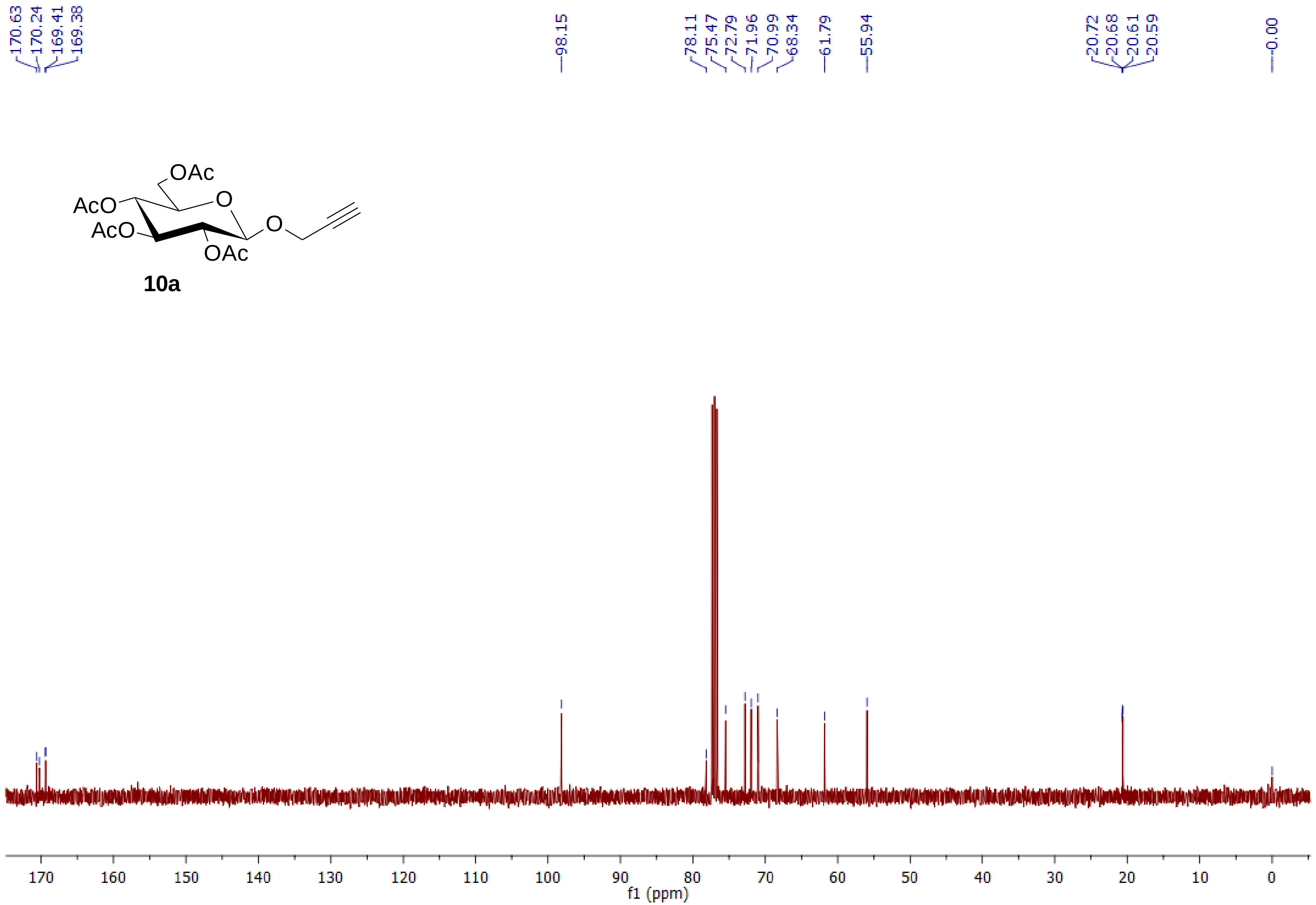

^{13}C NMR spectrum of propargyl 2,3,4,6-tetra-*O*-acetyl-β-D-glucopyranoside (**10a**); 100 MHz/$CDCl_3$/TMS; δ (ppm).

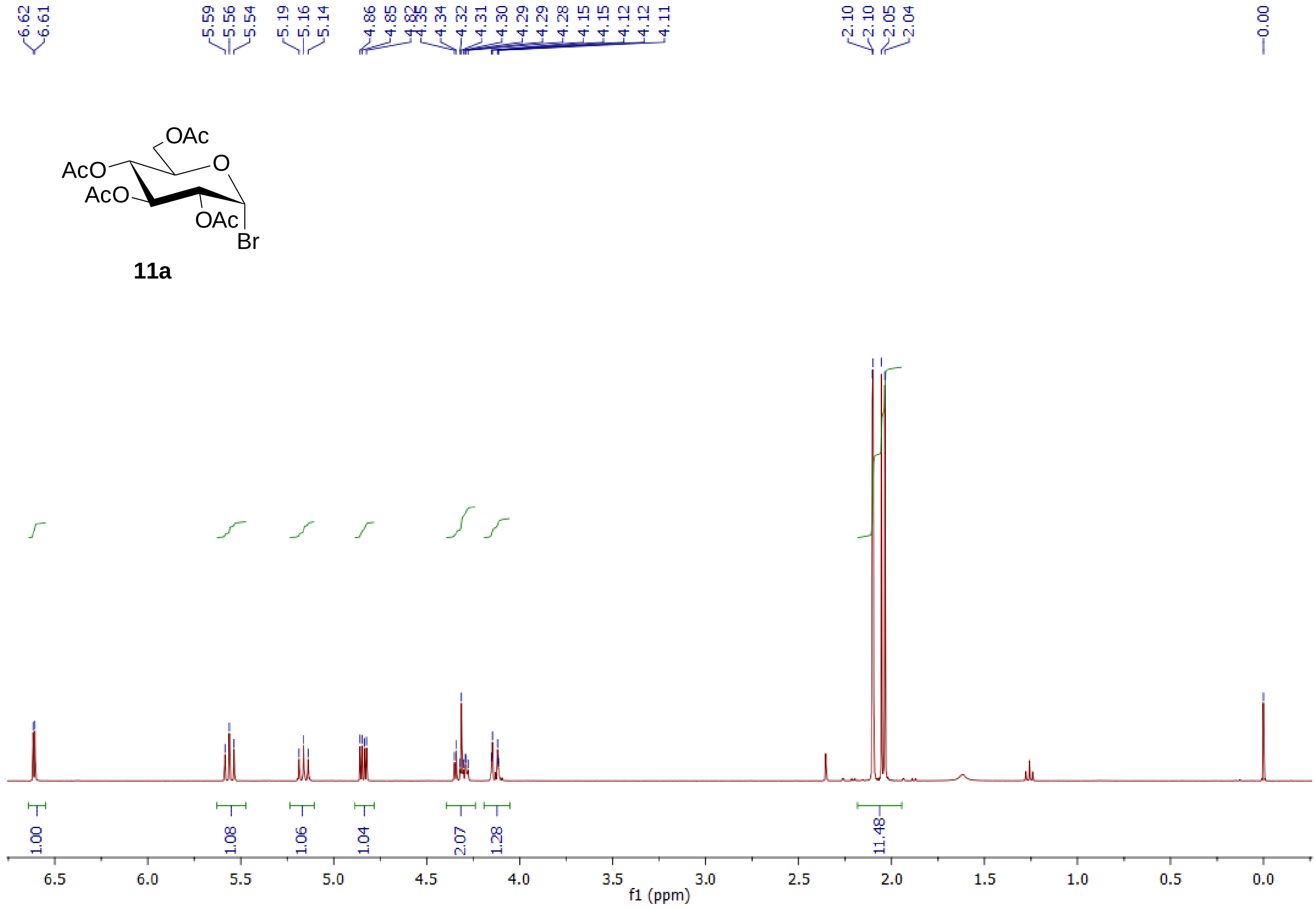

^{1}H NMR spectrum of 2,3,4,6-tetra-*O*-acetyl-α-D-glucopyranosyl bromide (**11a**); 400 MHz/$CDCl_3$/TMS; δ (ppm).

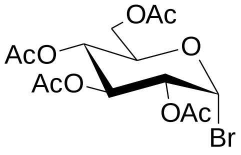

11a

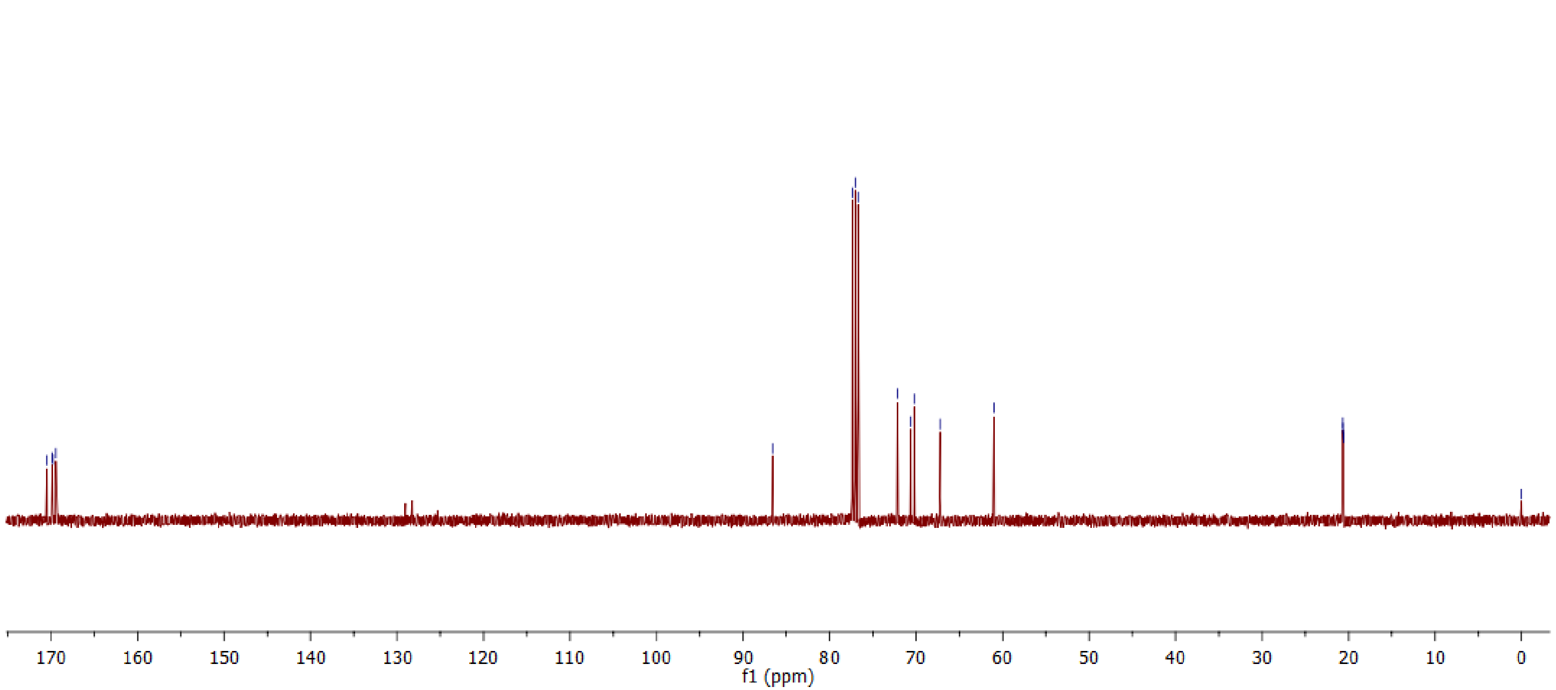

^{13}C NMR spectrum of 2,3,4,6-tetra-*O*-acetyl-α-D-glucopyranosyl bromide (**11a**); 100 MHz/$CDCl_3$/TMS; δ (ppm).

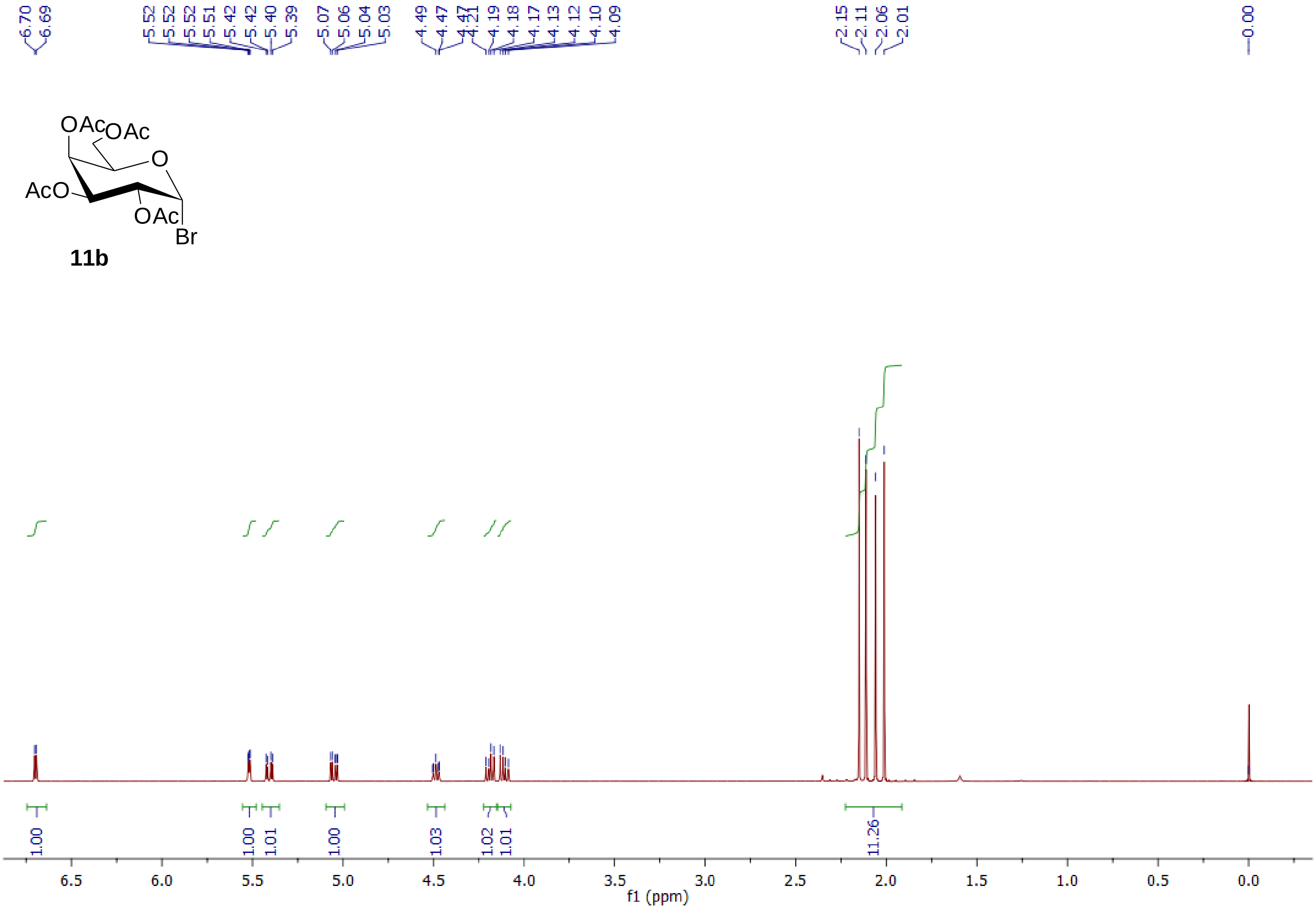

^{1}H NMR spectrum of 2,3,4,6-tetra-*O*-acetyl-α-D-galactopyranosyl bromide (**11b**); 400 MHz/CDCl3/TMS; δ (ppm).

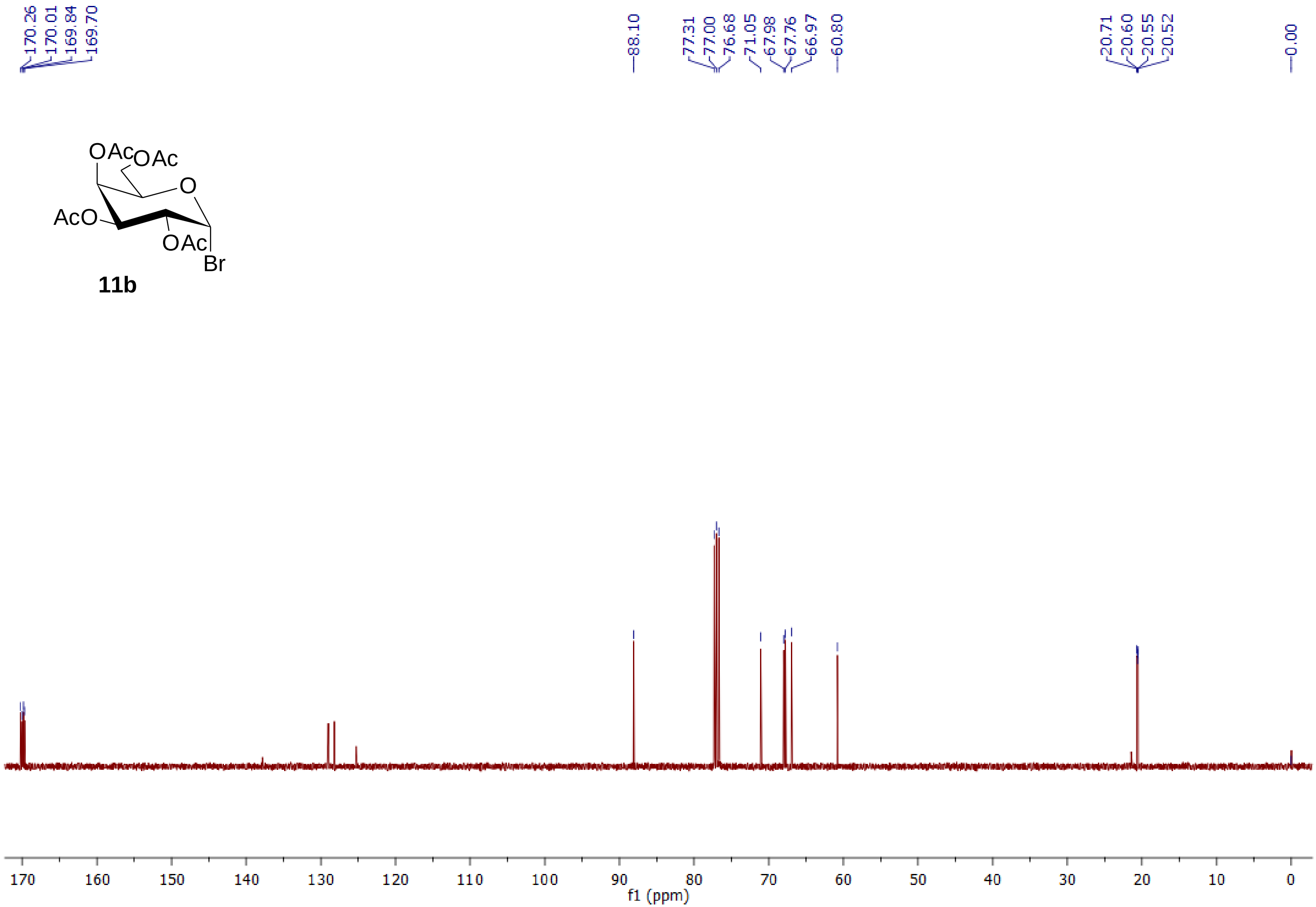

^{13}C NMR spectrum of 2,3,4,6-tetra-*O*-acetyl-α-D-galactopyranosyl bromide (**11b**); 100 MHz/$CDCl_3$/TMS; δ (ppm).

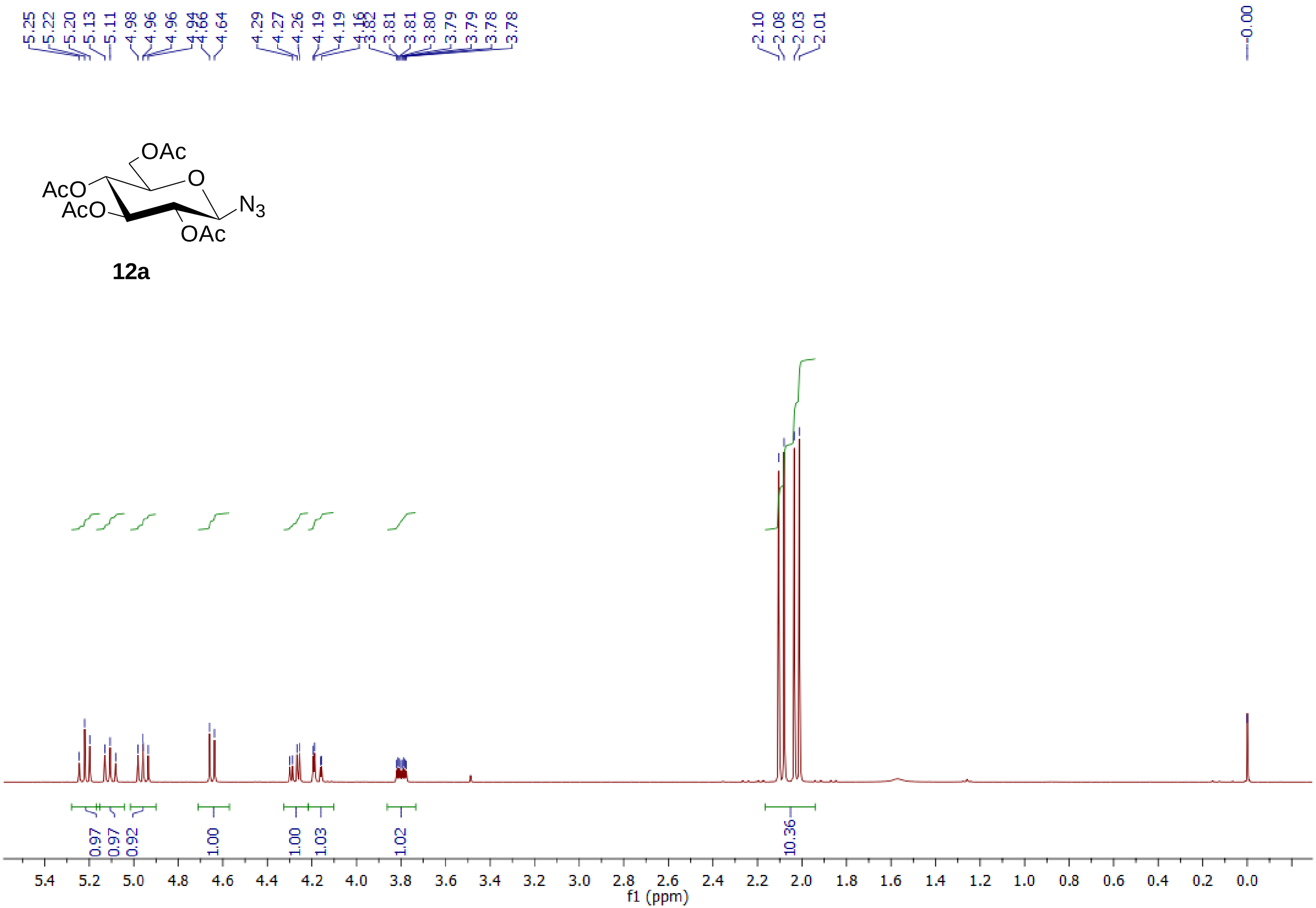

^{1}H NMR spectrum of 2,3,4,6-tetra-*O*-acetyl-β-D-glucopyranosyl azide (**12a**); 400 MHz/$CDCl_3$/TMS; δ (ppm).

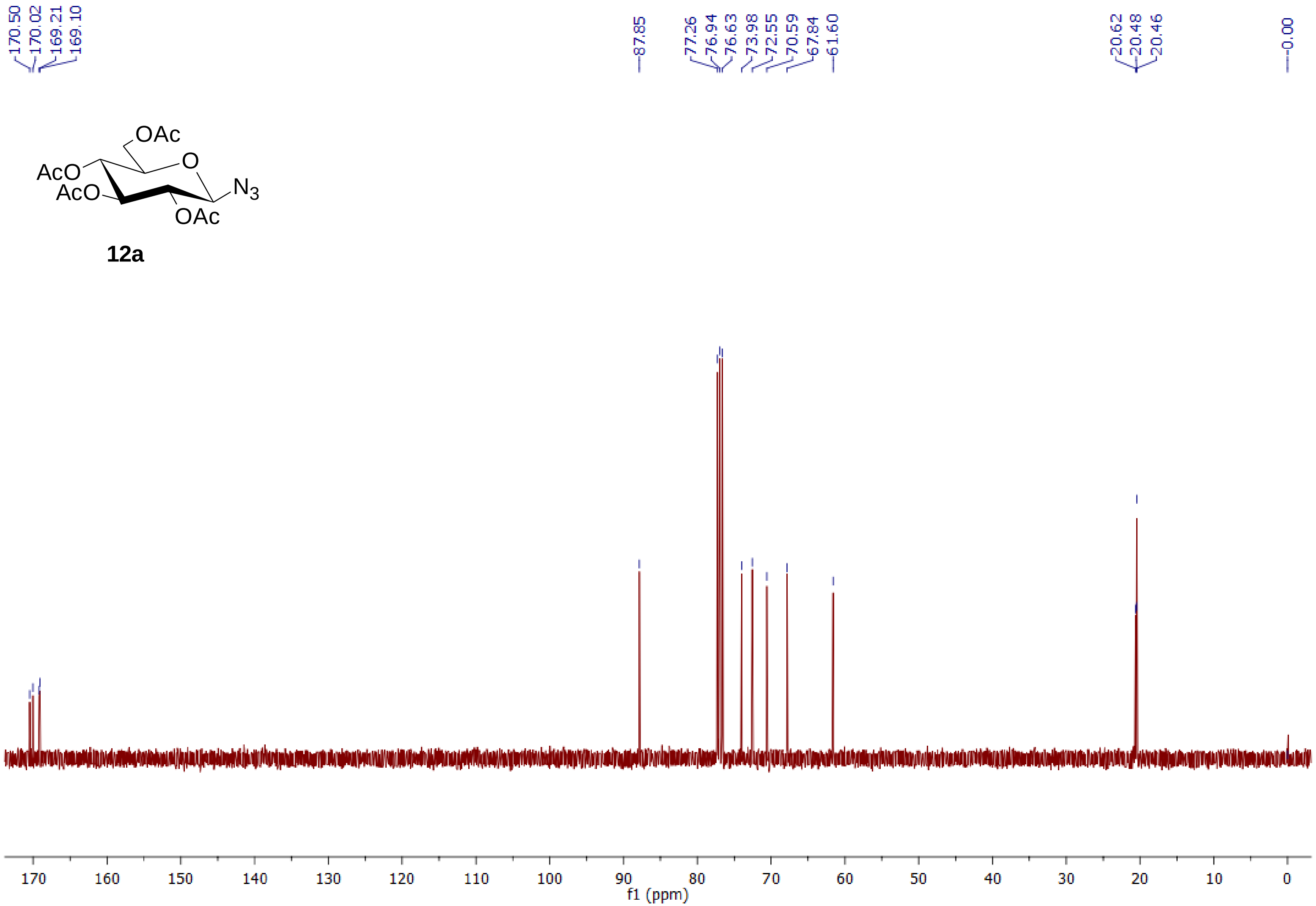

^{13}C NMR spectrum of 2,3,4,6-tetra-*O*-acetyl-β-D-glucopyranosyl azide (**12a**); 100 MHz/$CDCl_3$/TMS; δ (ppm).

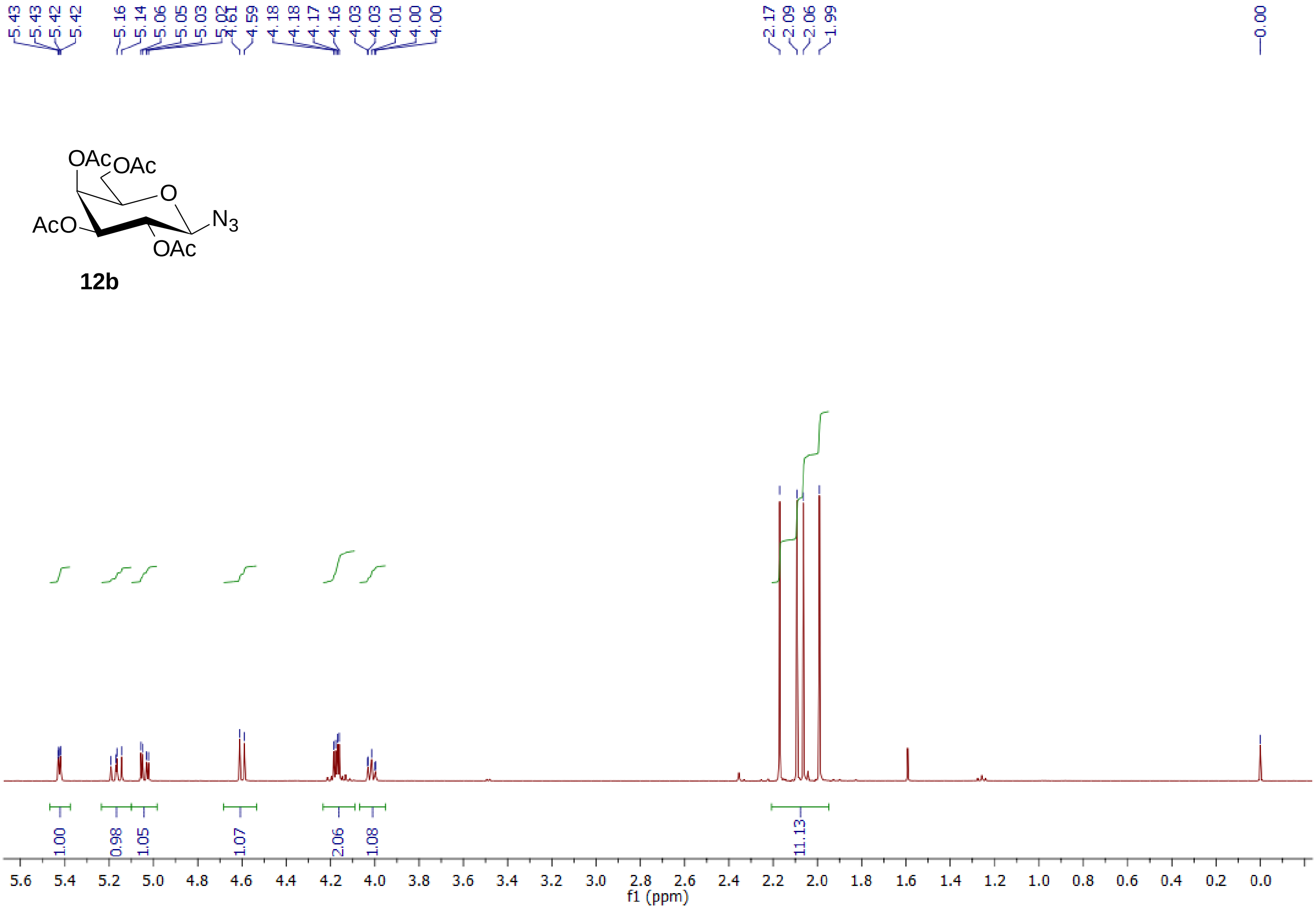

^{1}H NMR spectrum of 2,3,4,6-tetra-*O*-acetyl-β-D-galactopyranosyl azide (**12b**); 400 MHz/$CDCl_3$/TMS; δ (ppm).

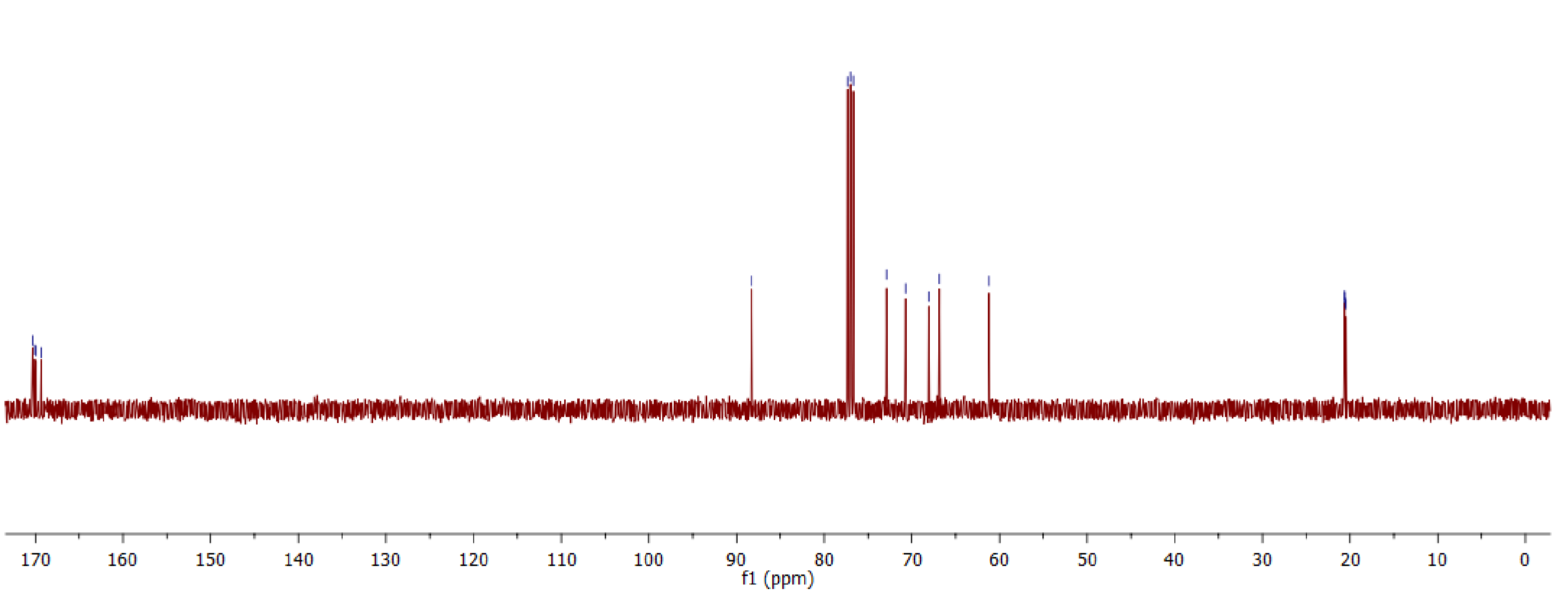

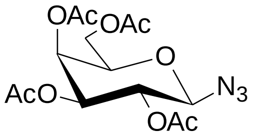

12b

^{13}C NMR spectrum of 2,3,4,6-tetra-*O*-acetyl-β-D-galactopyranosyl azide (**12b**); 100 MHz/$CDCl_3$/TMS; δ (ppm).

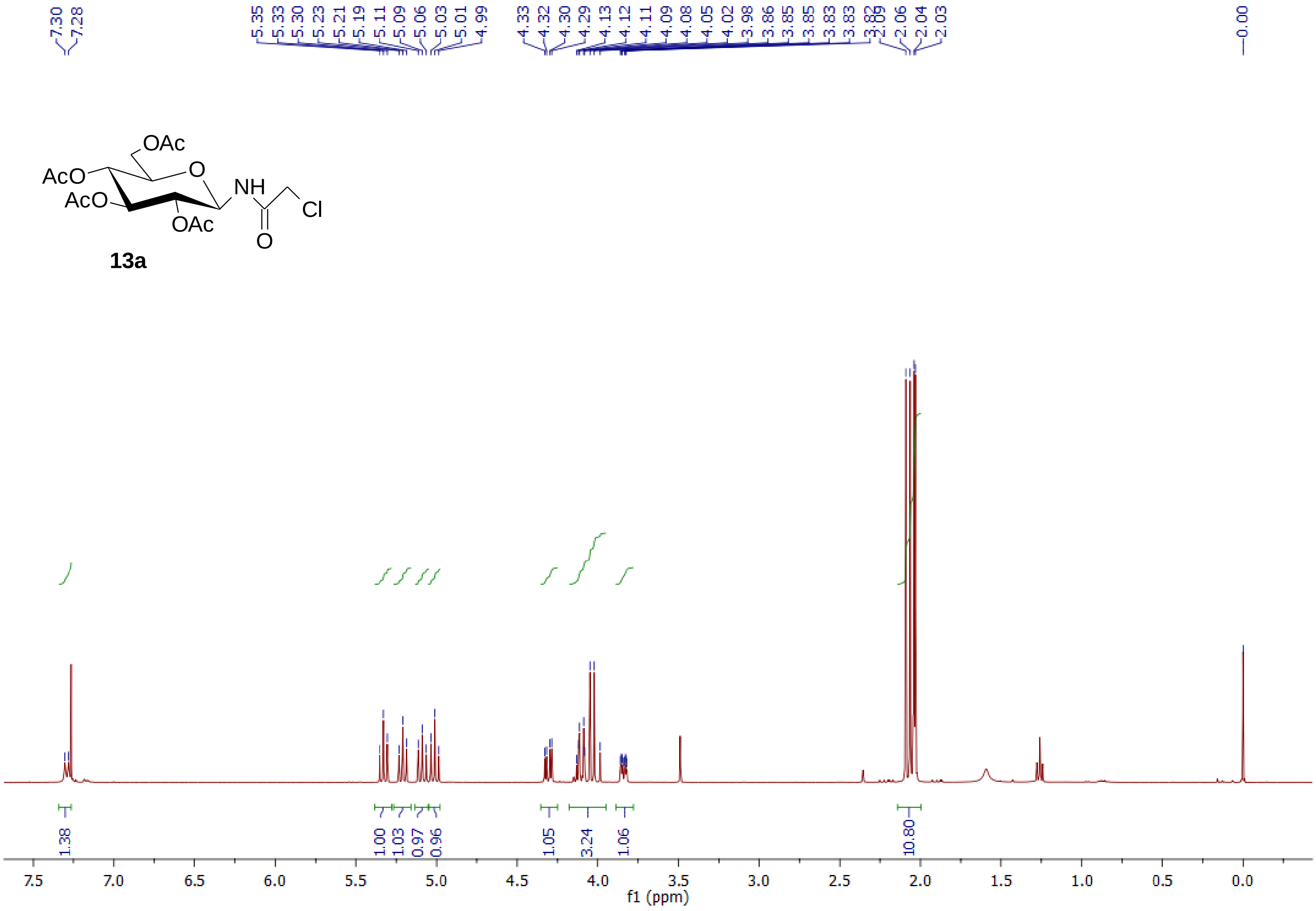

^{1}H NMR spectrum of 2,3,4,6-tetra-*O*-acetyl-*N*-(β-D-glucopyranosyl)chloroacetamide (**13a**); 400 MHz/$CDCl_3$/TMS; δ (ppm).

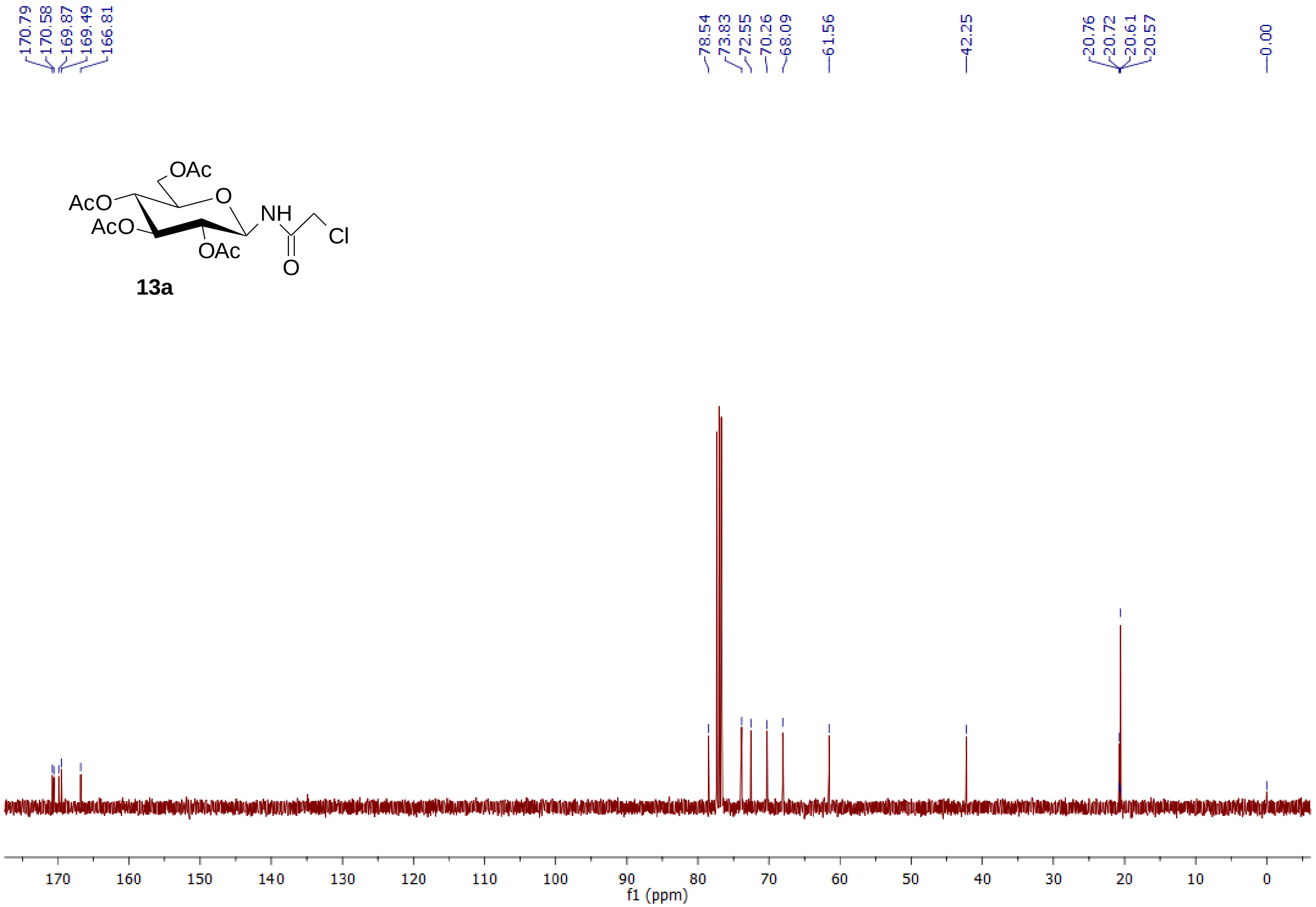

^{13}C NMR spectrum of 2,3,4,6-tetra-*O*-acetyl-*N*-(β-D-glucopyranosyl)chloroacetamide (**13a**); 100 MHz/$CDCl_3$/TMS; δ (ppm).

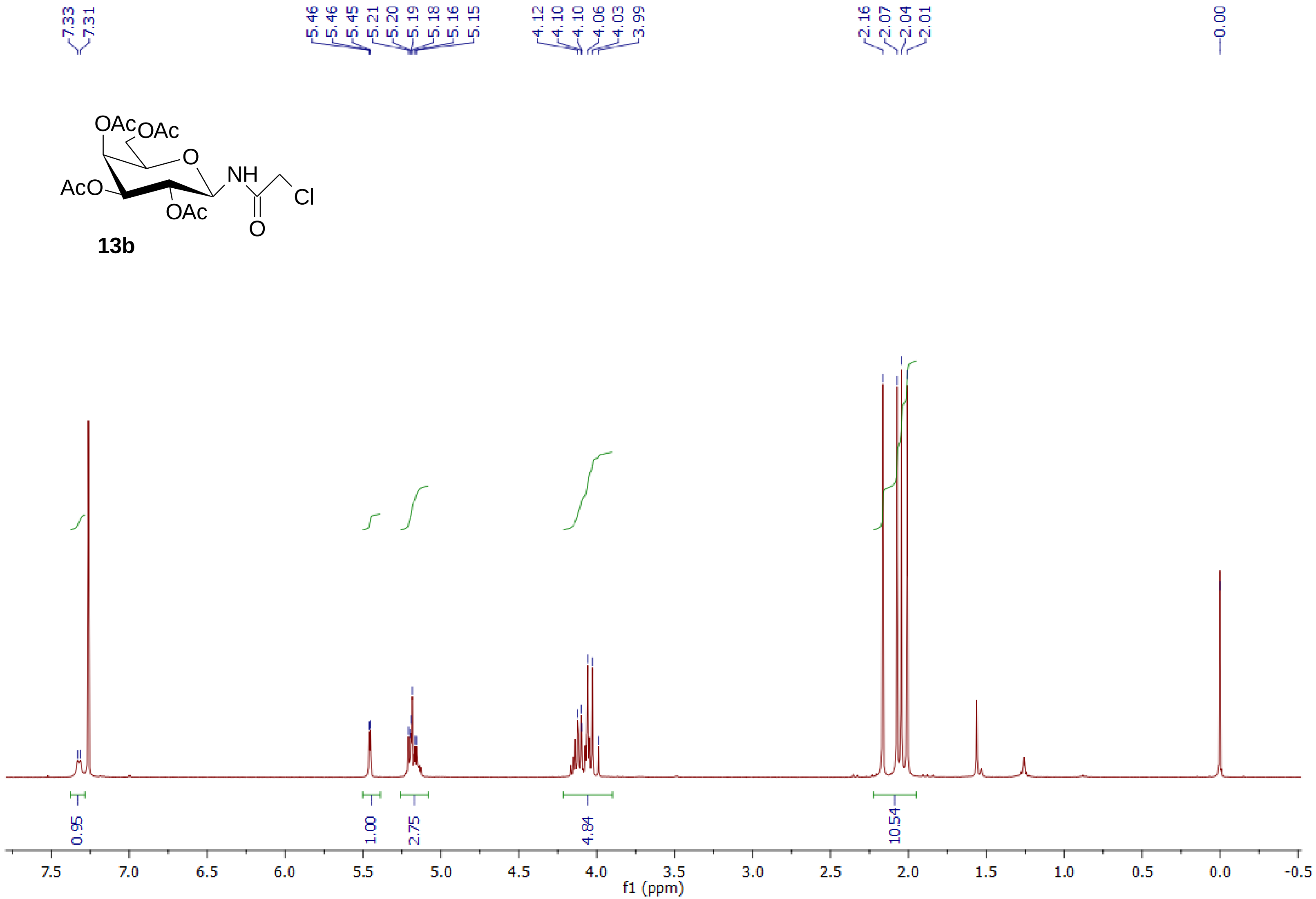

^{1}H NMR spectrum of 2,3,4,6-tetra-*O*-acetyl-*N*-(β-D-galactopyranosyl)chloroacetamide (**13b**); 400 MHz/$CDCl_3$/TMS; δ (ppm).

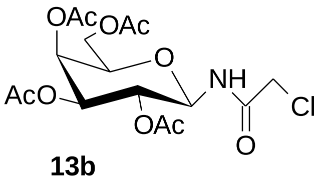

13b

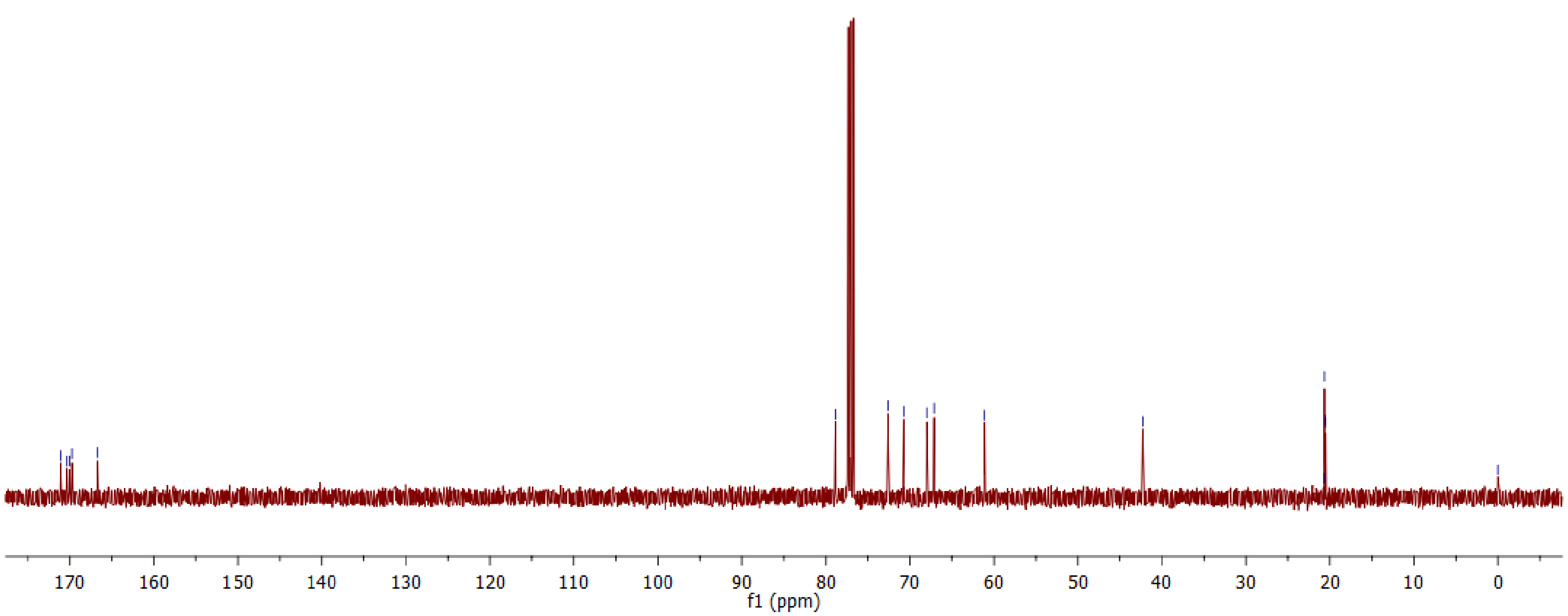

^{13}C NMR spectrum of 2,3,4,6-tetra-*O*-acetyl-*N*-(β-D-galactopyranosyl)chloroacetamide (**13b**); 100 MHz/$CDCl_3$/TMS; δ (ppm).

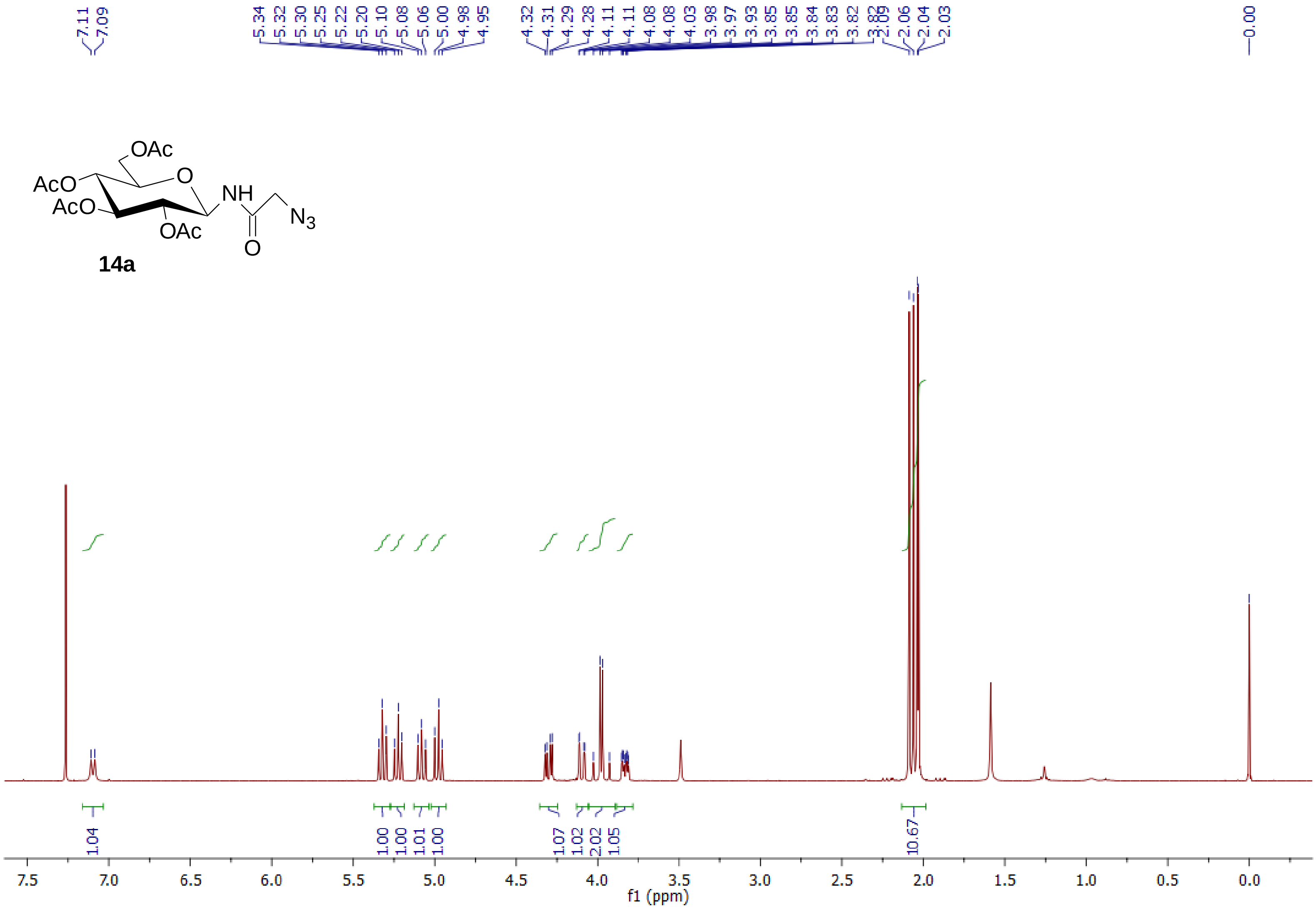

^{1}H NMR spectrum of 2,3,4,6-tetra-*O*-acetyl-*N*-(β-D-glucopyranosyl)azidoacetamide (**14a**); 400 MHz/$CDCl_3$/TMS; δ (ppm).

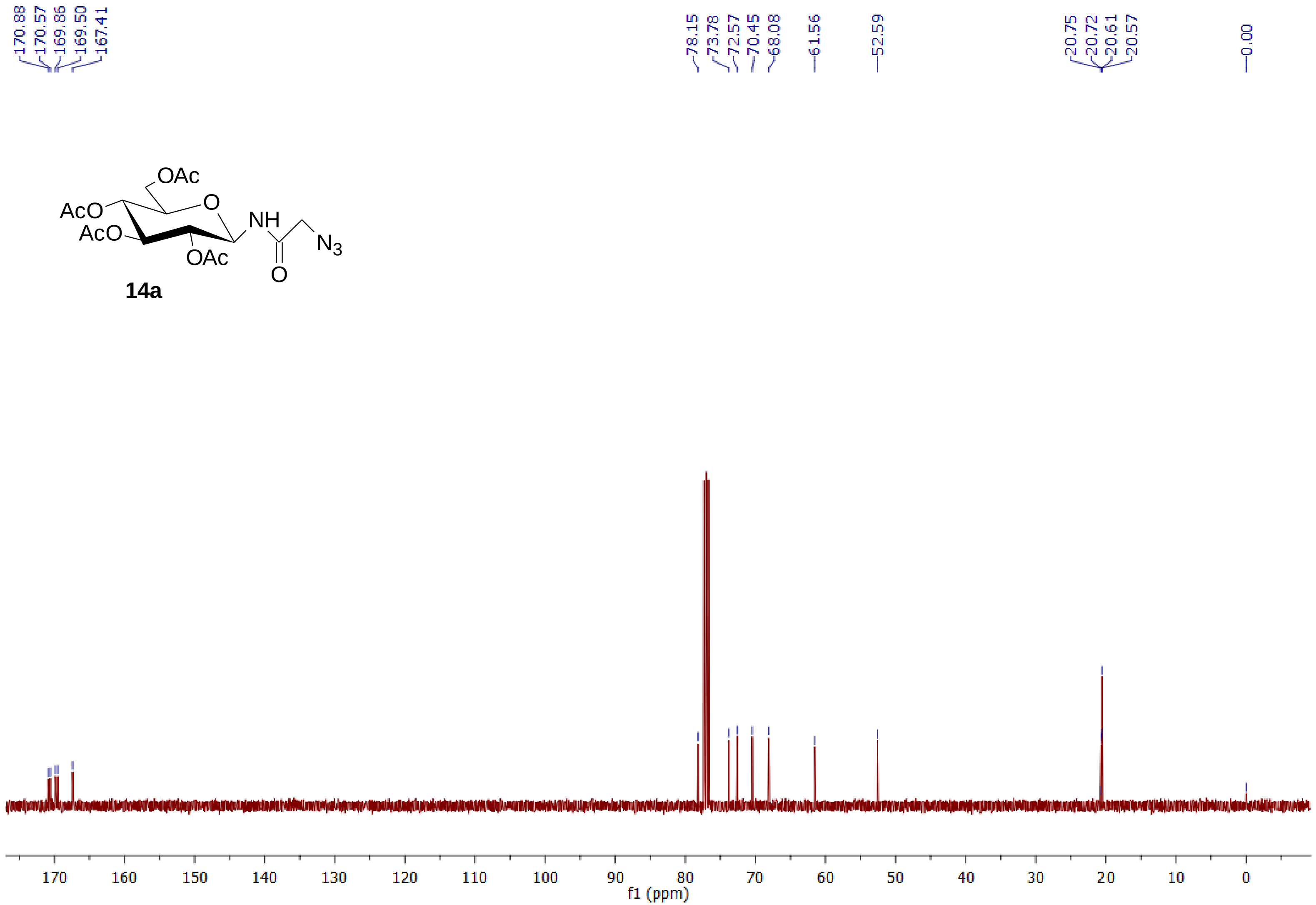

^{13}C NMR spectrum of 2,3,4,6-tetra-*O*-acetyl-*N*-(β-D-glucopyranosyl)azidoacetamide (**14a**); 100 MHz/$CDCl_3$/TMS; δ (ppm).

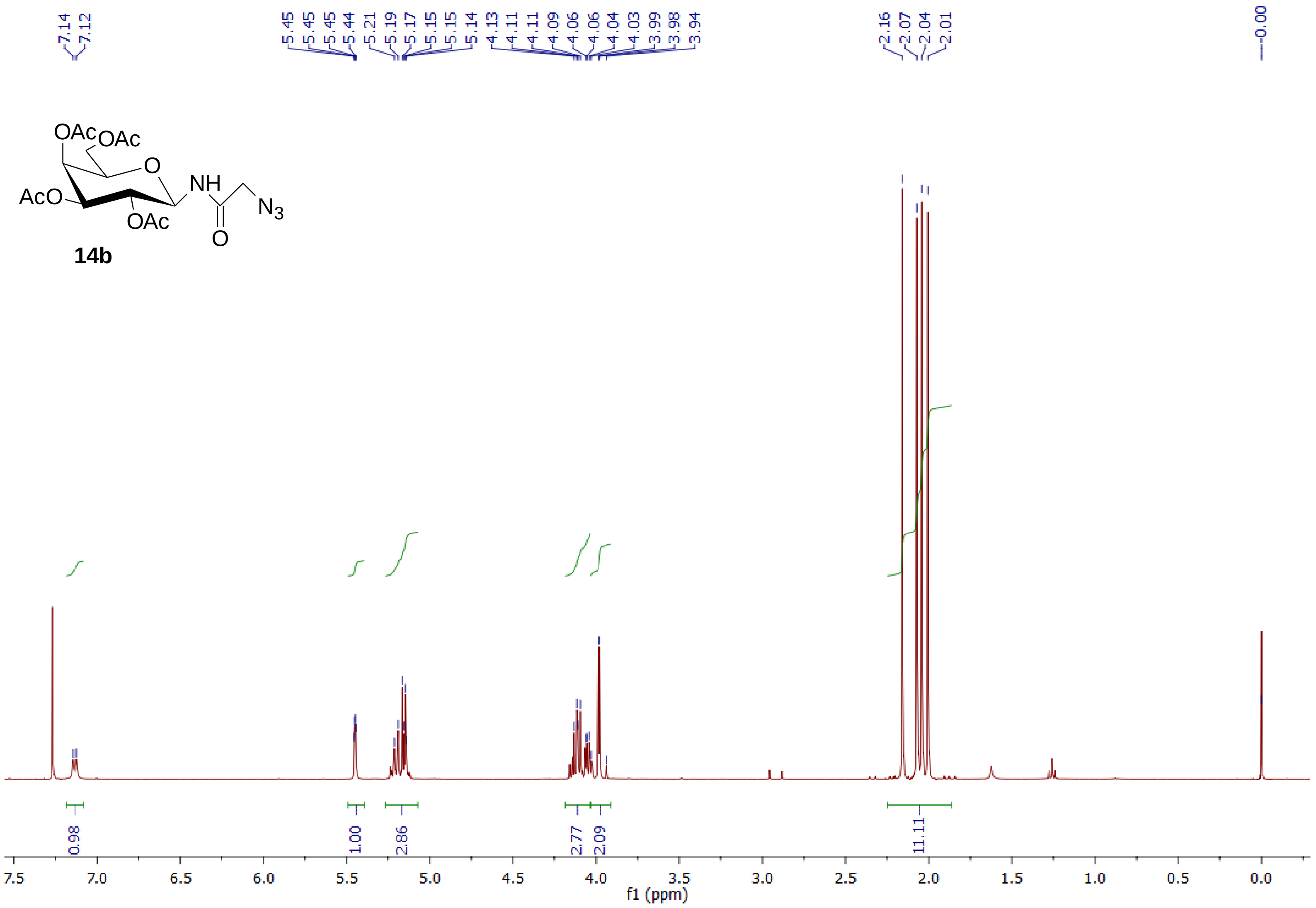

^{1}H NMR spectrum of 2,3,4,6-tetra-*O*-acetyl-*N*-(β-D-galactopyranosyl)azidoacetamide (**14b**); 400 MHz/$CDCl_3$/TMS; δ (ppm).

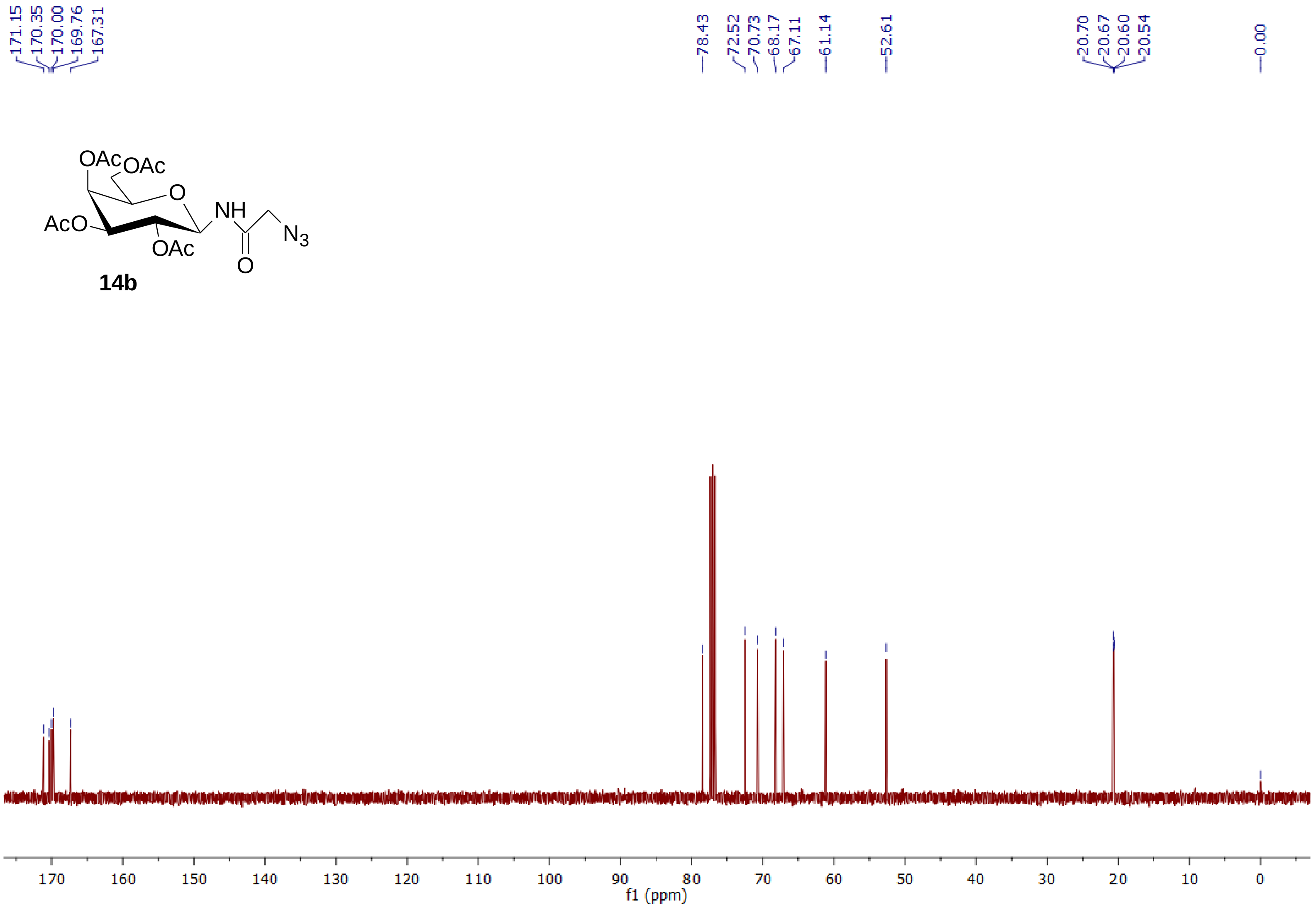

^{13}C NMR spectrum of 2,3,4,6-tetra-*O*-acetyl-*N*-(β-D-galactopyranosyl)azidoacetamide (**14b**); 100 MHz/$CDCl_3$/TMS; δ (ppm).

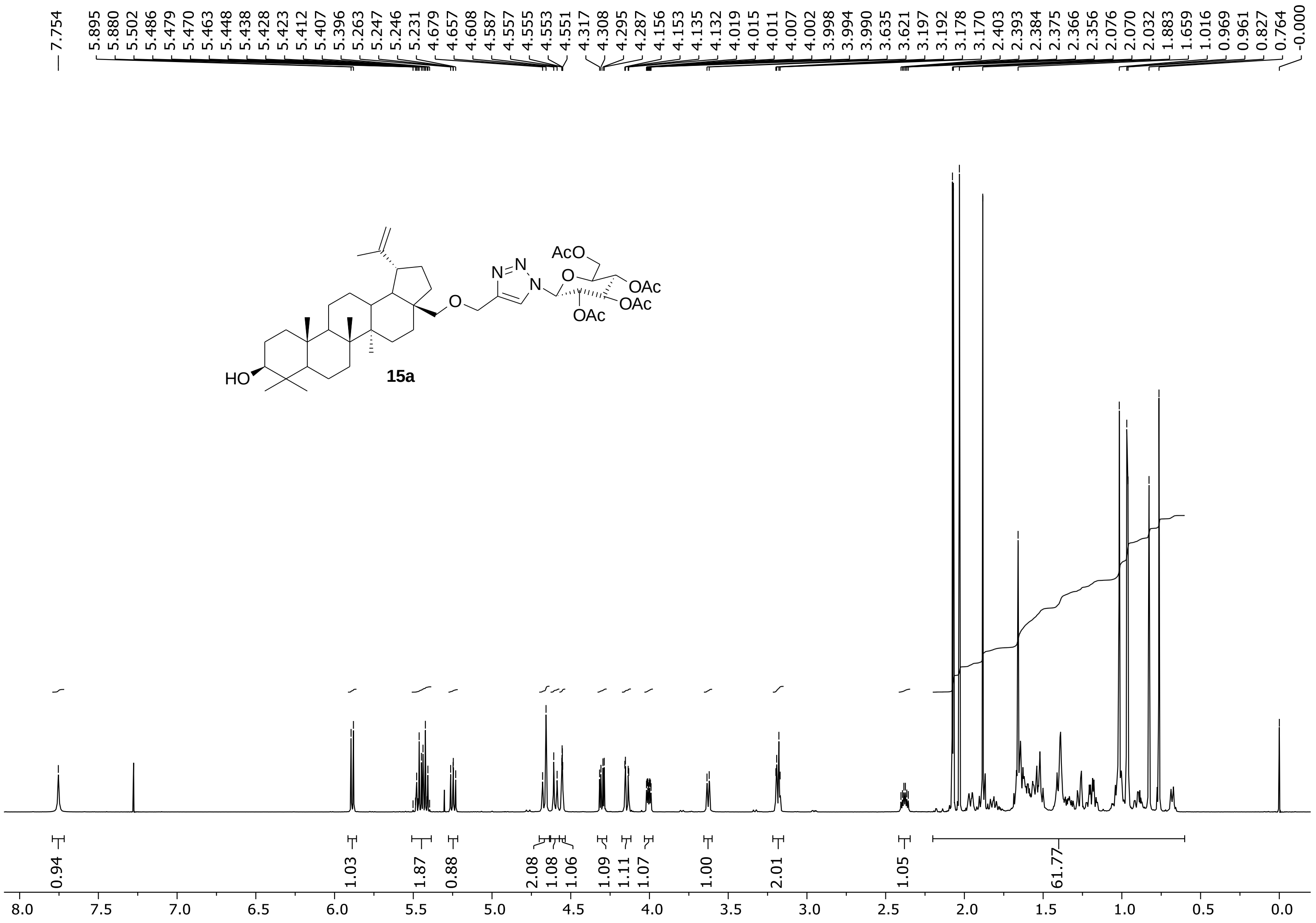

^{1}H NMR spectrum of glycoconjugates type **I** (**15a**); 600 MHz/$CDCl_3$/TMS; δ (ppm).

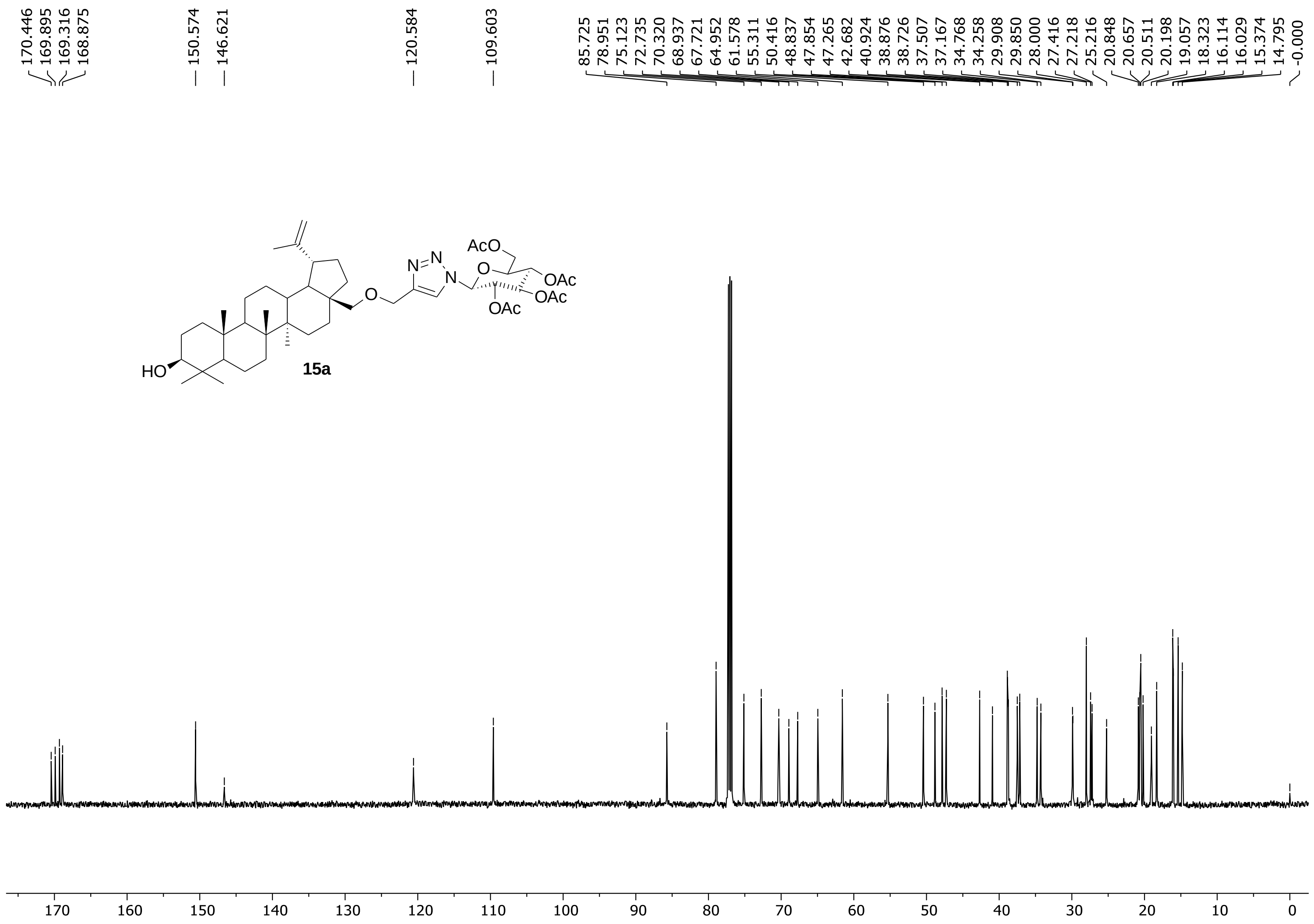

^{13}C NMR spectrum of glycoconjugates type **I** (**15a**); 150 MHz/$CDCl_3$/TMS; δ (ppm).

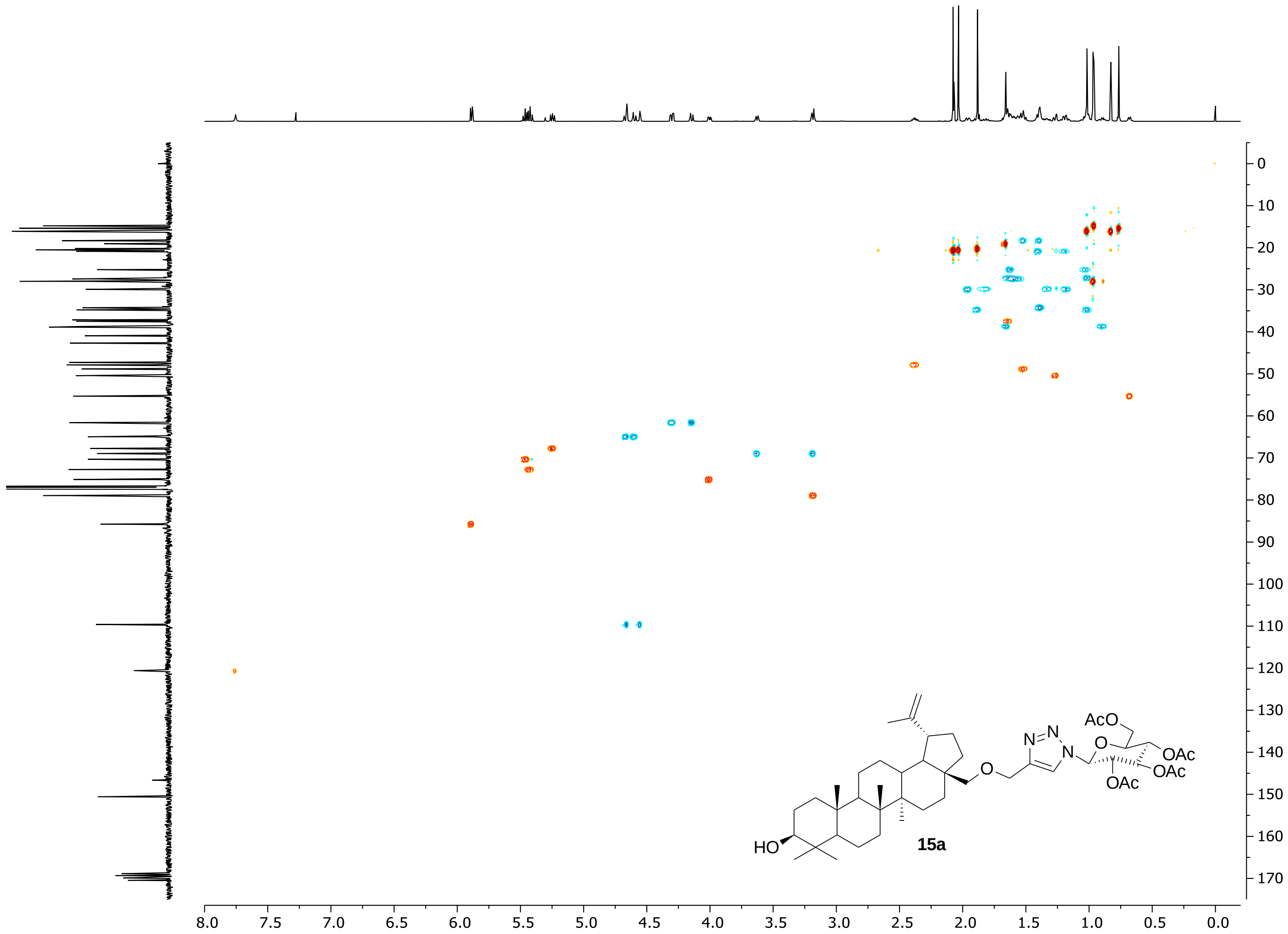

gHSQC spectrum of glycoconjugates type **I** (**15a**).

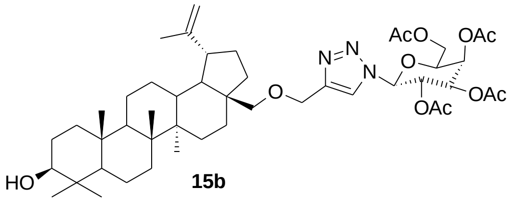

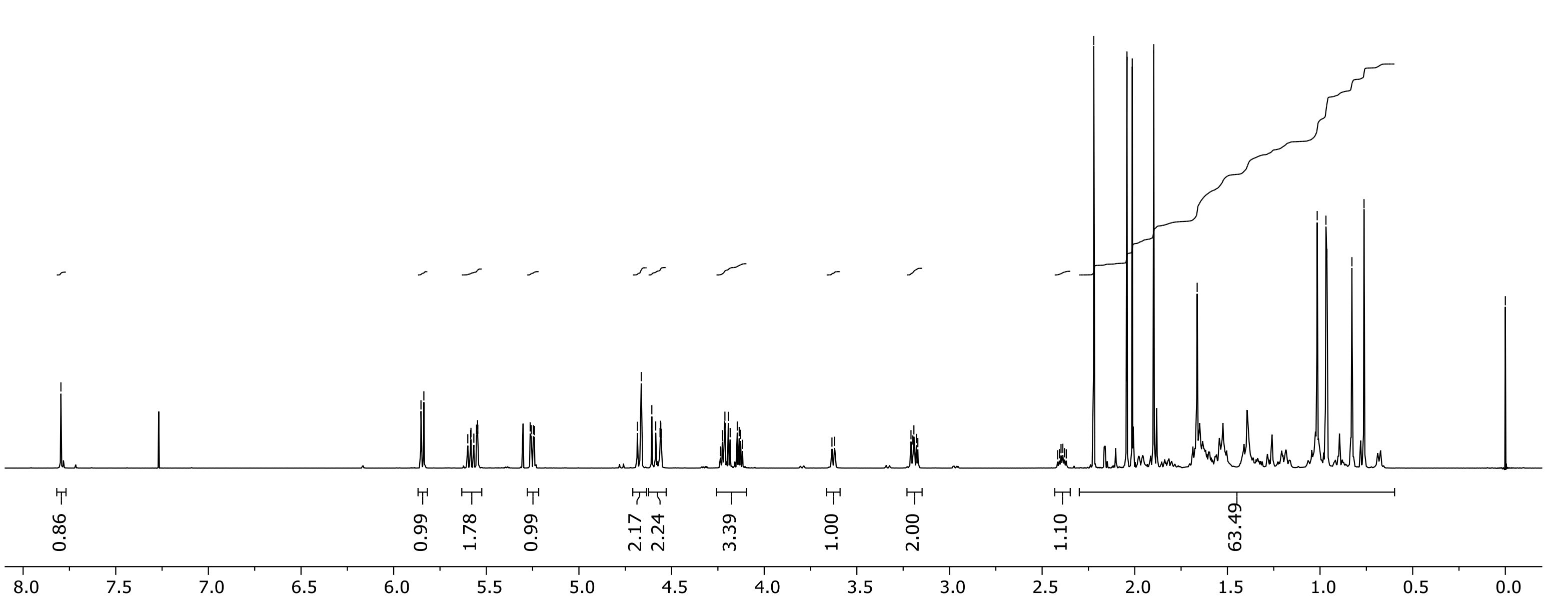

^{1}H NMR spectrum of glycoconjugates type **I** (**15b**); 600 MHz/$CDCl_3$/TMS; δ (ppm).

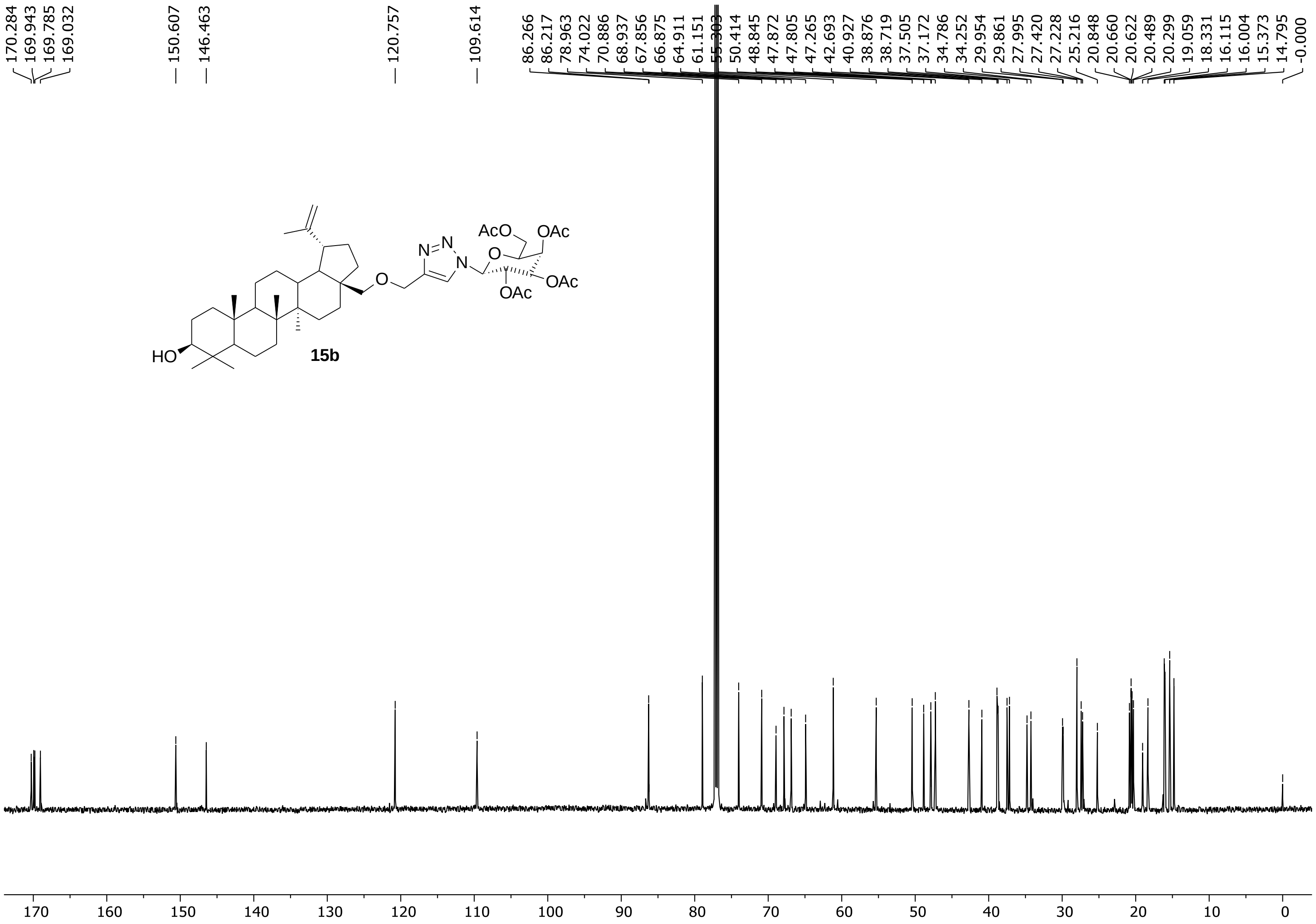

^{13}C NMR spectrum of glycoconjugates type **I** (**15b**); 150 MHz/$CDCl_3$/TMS; δ (ppm).

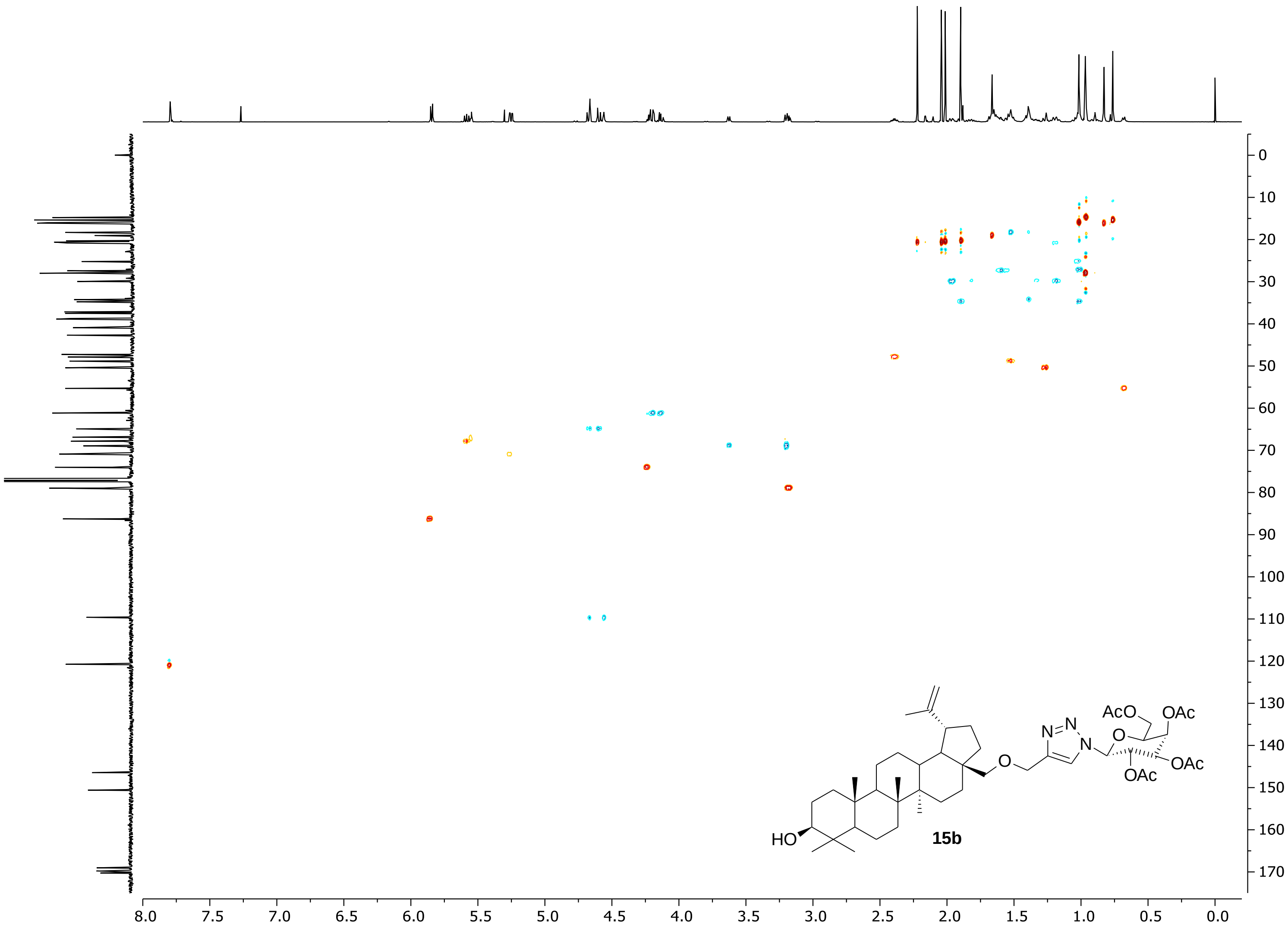

gHSQC spectrum of glycoconjugates type **I** (**15b**).

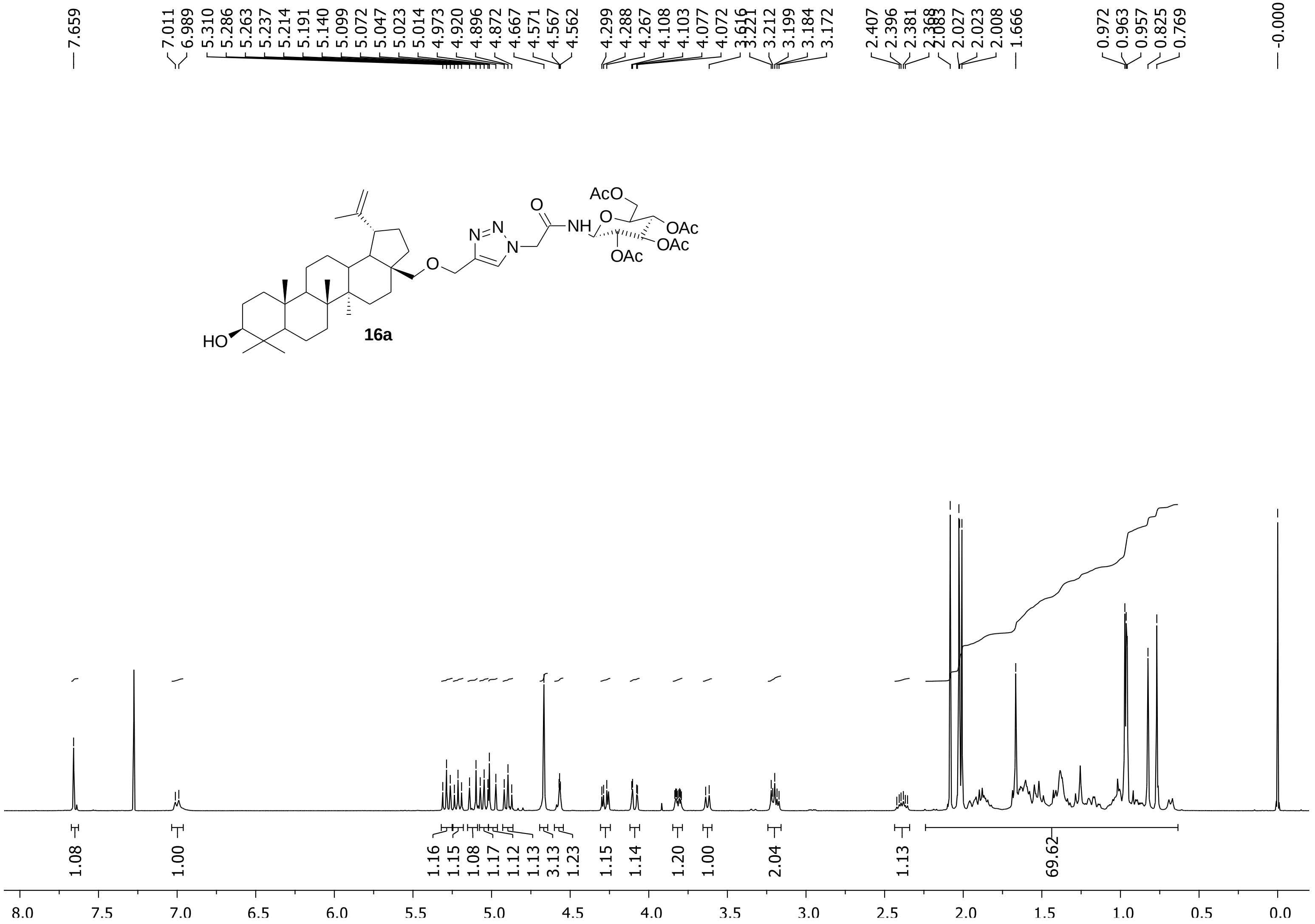

^{1}H NMR spectrum of glycoconjugates type **II** (**16a**); 600 MHz/$CDCl_3$/TMS; δ (ppm).

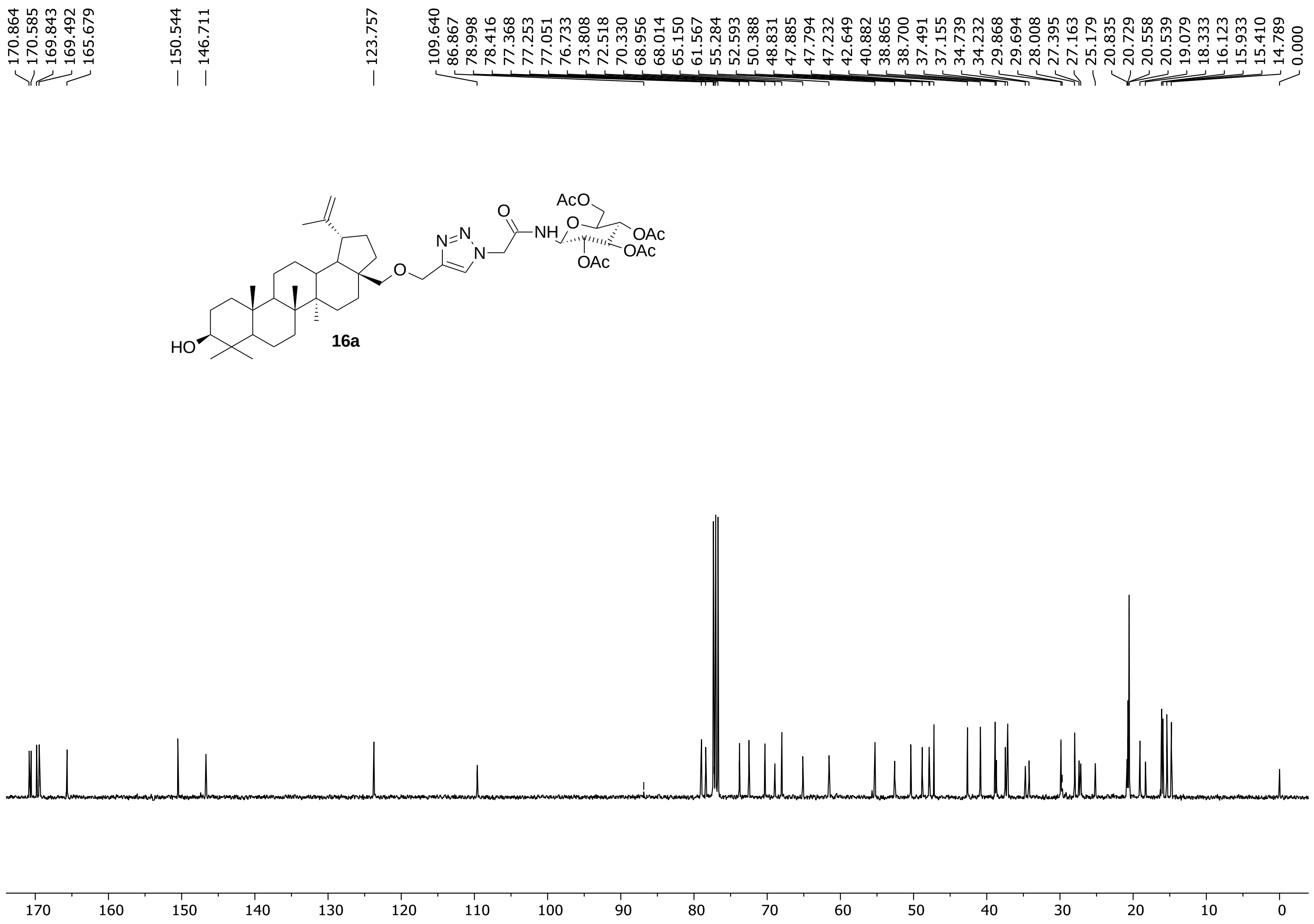

^{13}C NMR spectrum of glycoconjugates type **II** (**16a**); 150 MHz/$CDCl_3$/TMS; δ (ppm).

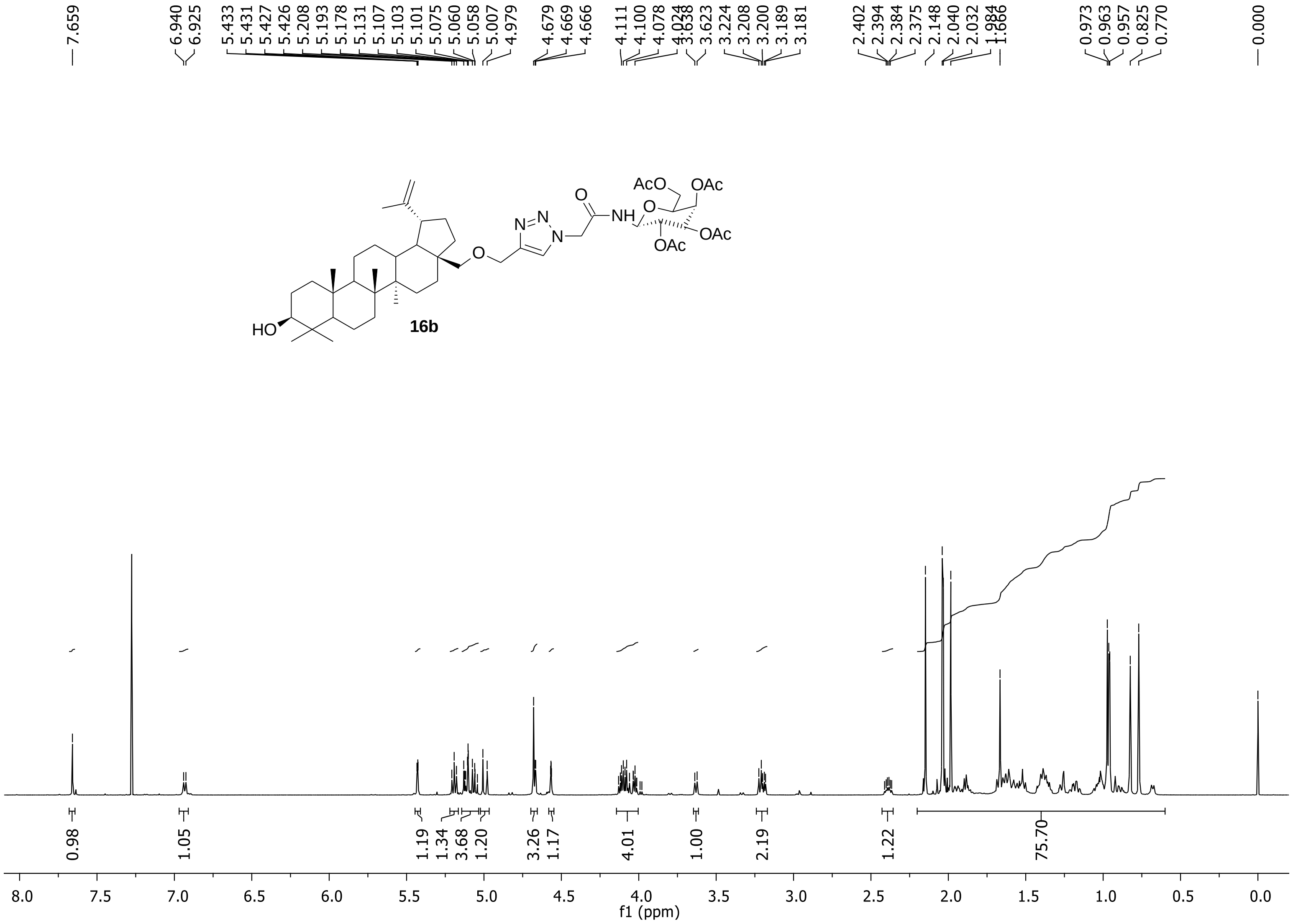

^{1}H NMR spectrum of glycoconjugates type **II** (**16b**); 600 MHz/CDCl$_3$/TMS; δ (ppm).

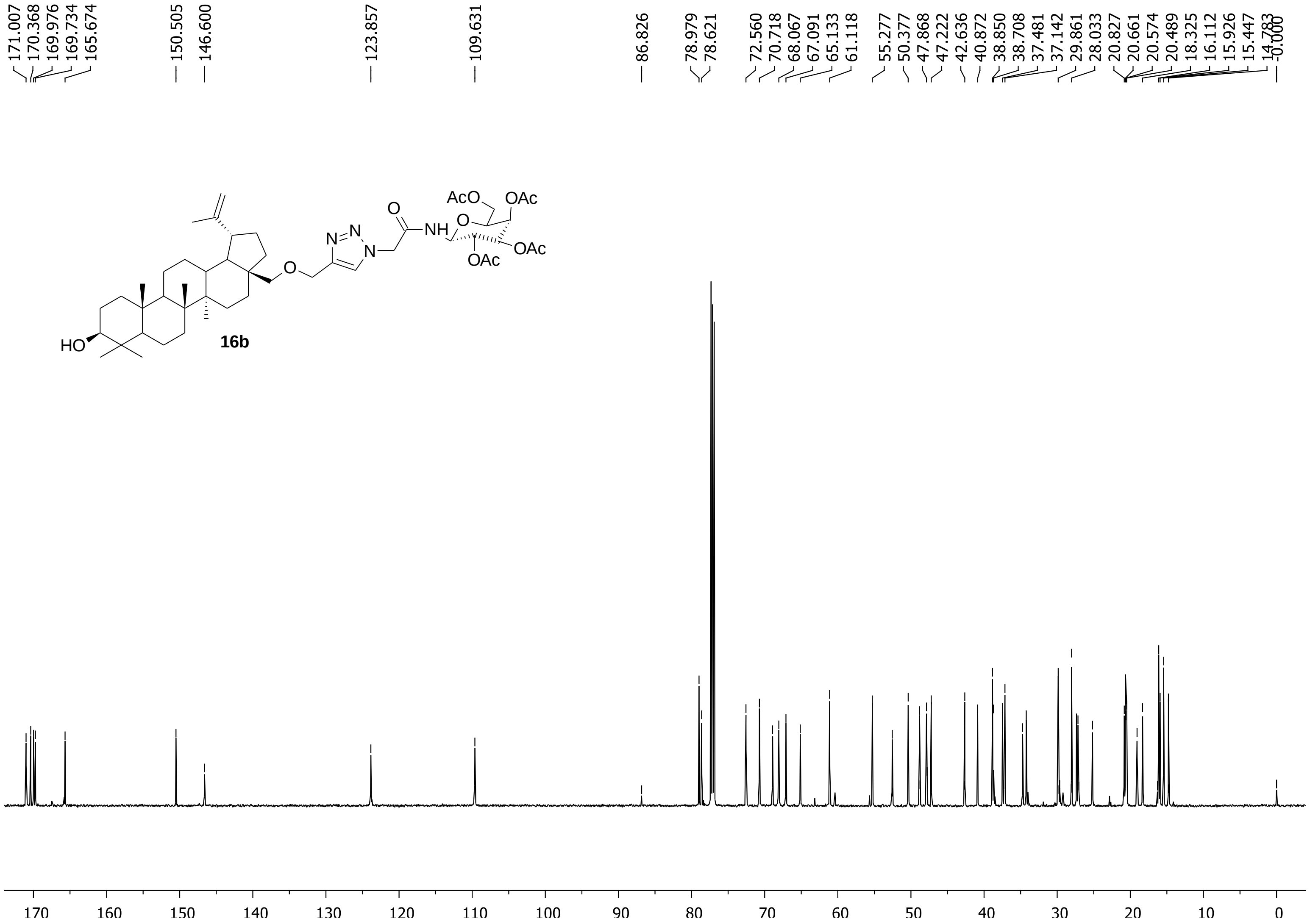

^{13}C NMR spectrum of glycoconjugates type **II** (**16b**); 150 MHz/$CDCl_3$/TMS; δ (ppm).

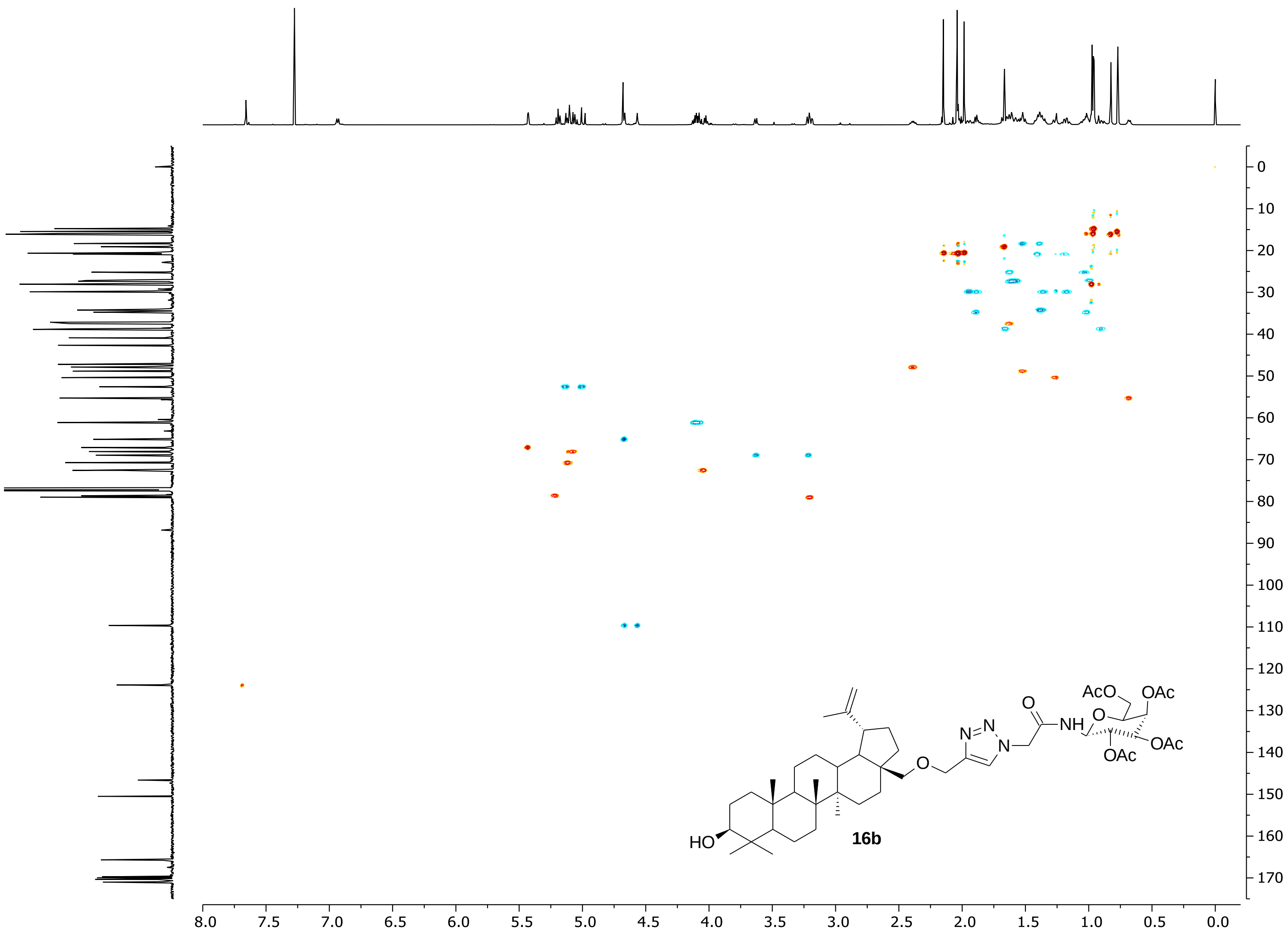

gHSQC spectrum of glycoconjugates type **II** (**16b**).

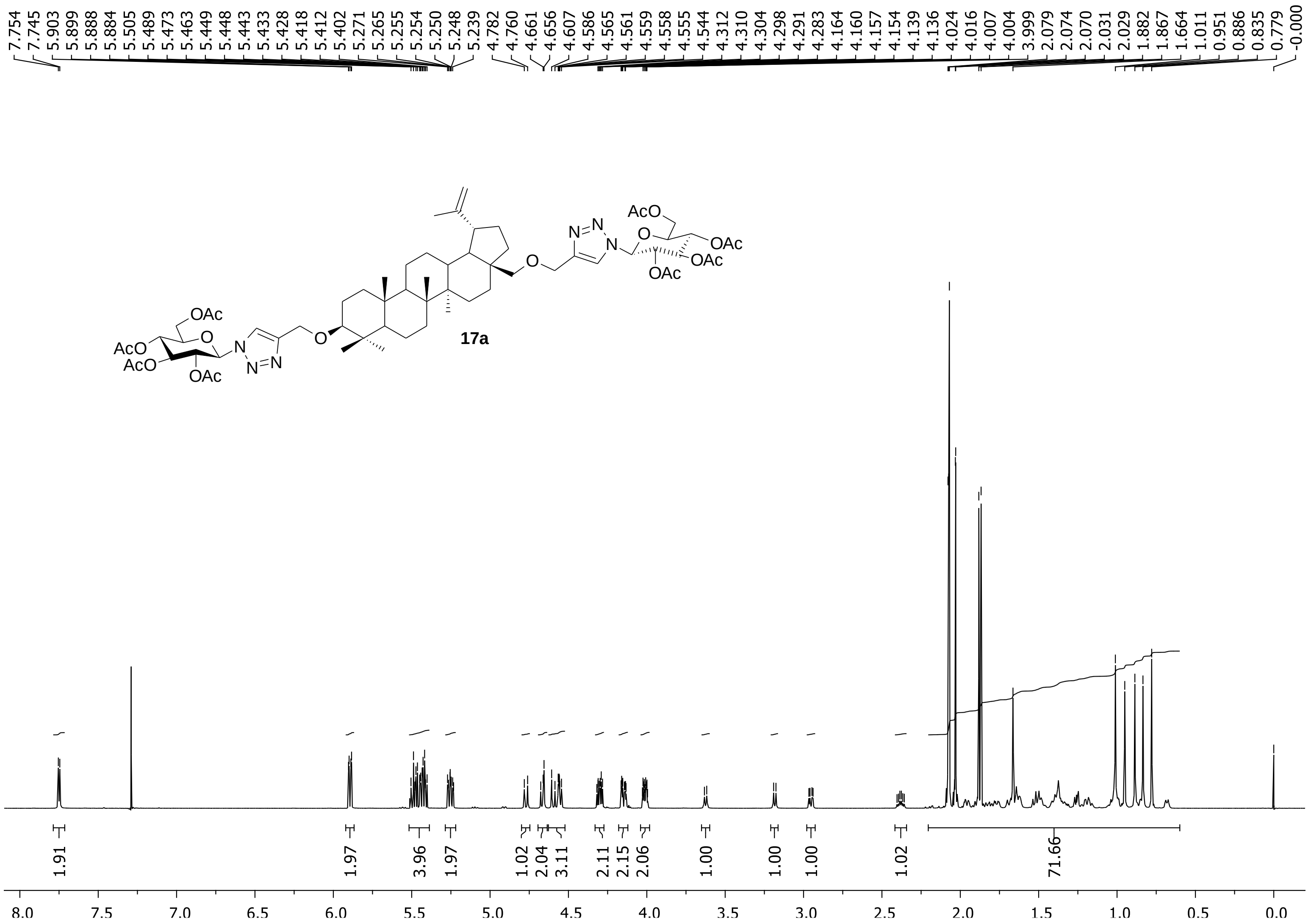

^{1}H NMR spectrum of glycoconjugates type **III** (**17a**); 600 MHz/$CDCl_3$/TMS; δ (ppm).

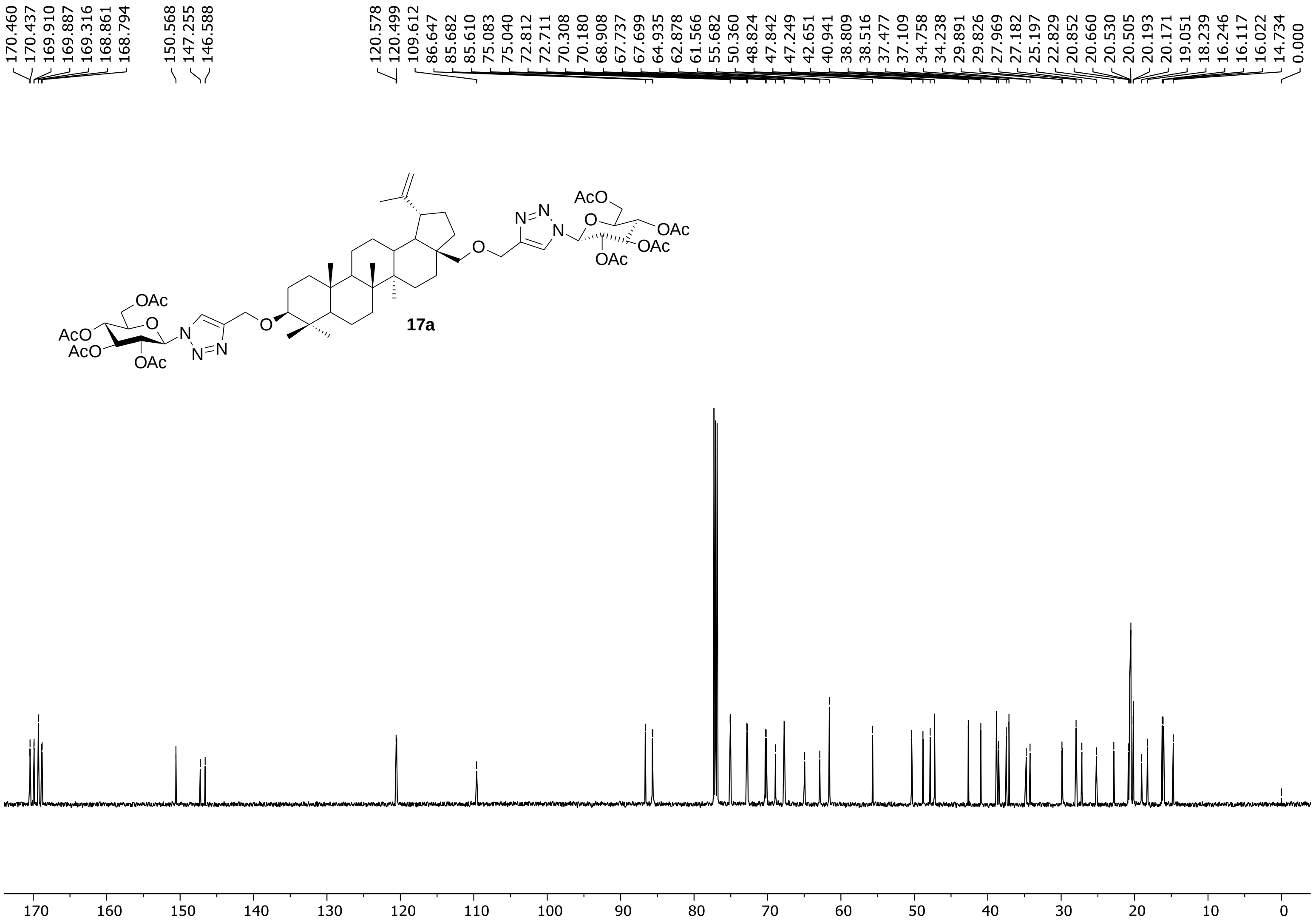

^{13}C NMR spectrum of glycoconjugates type **III** (**17a**); 150 MHz/CDCl3/TMS; δ (ppm).

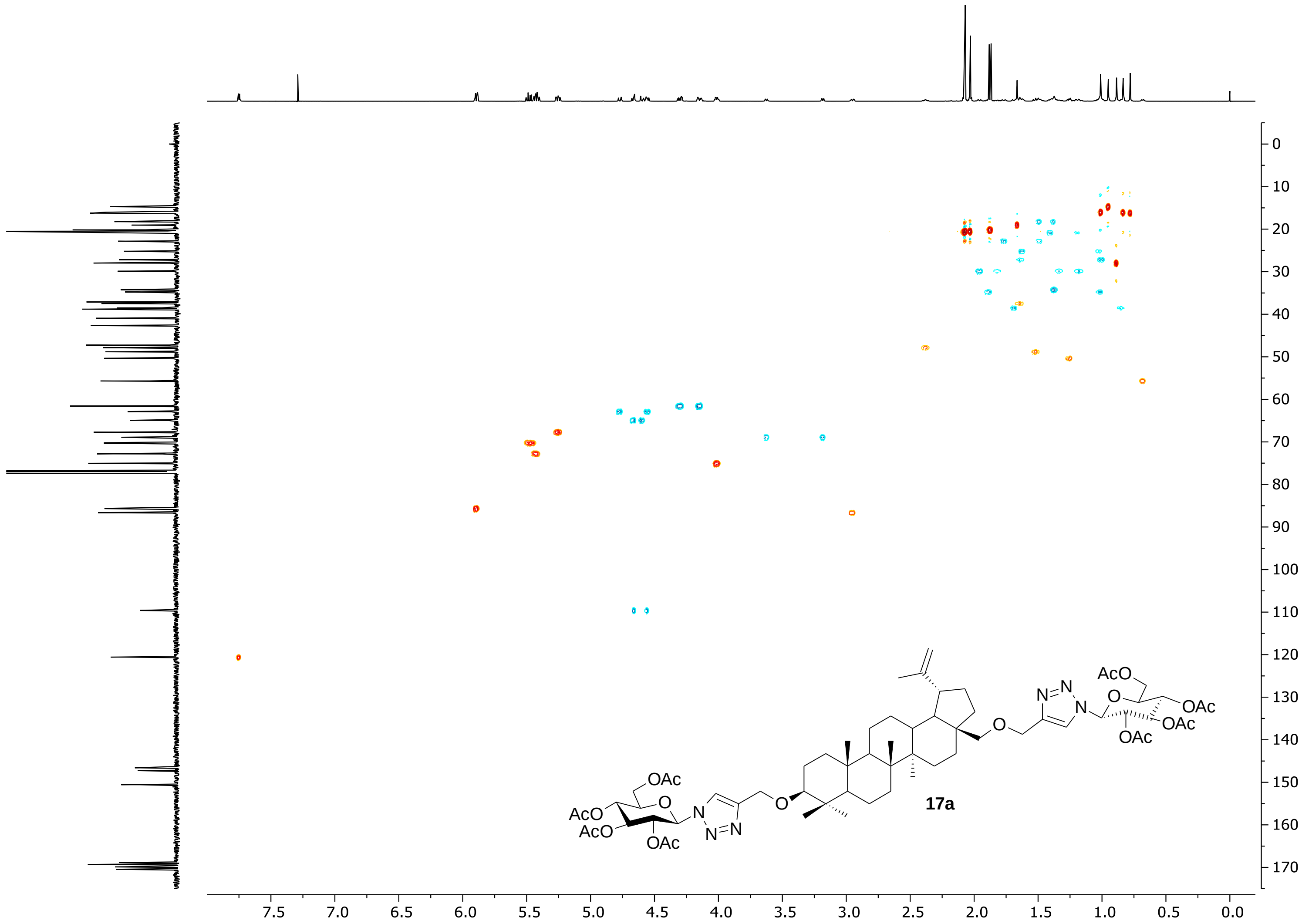

gHSQC spectrum of glycoconjugates type **III** (**17a**).

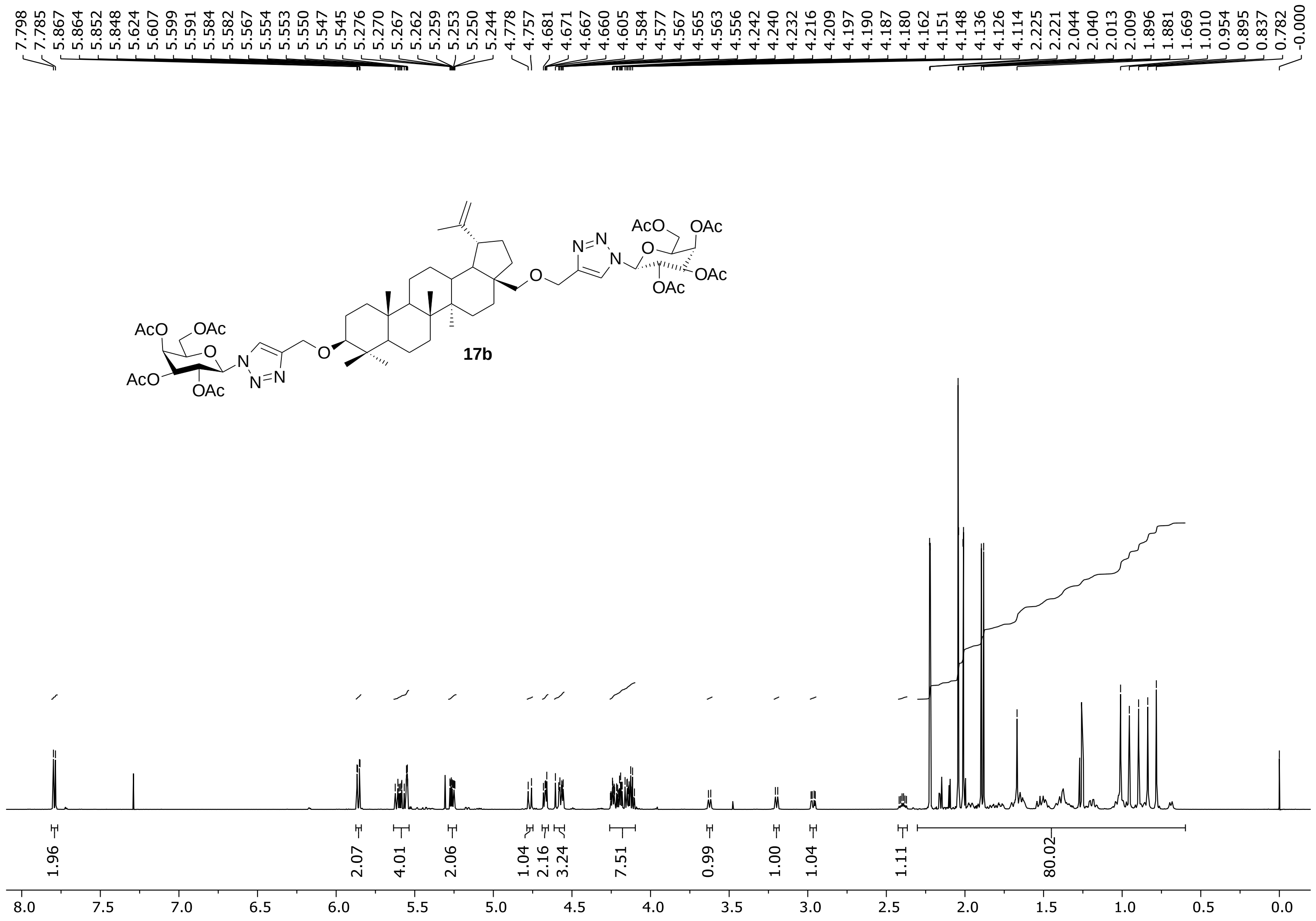

^{1}H NMR spectrum of glycoconjugates type **III** (**17b**); 600 MHz/$CDCl_3$/TMS; δ (ppm).

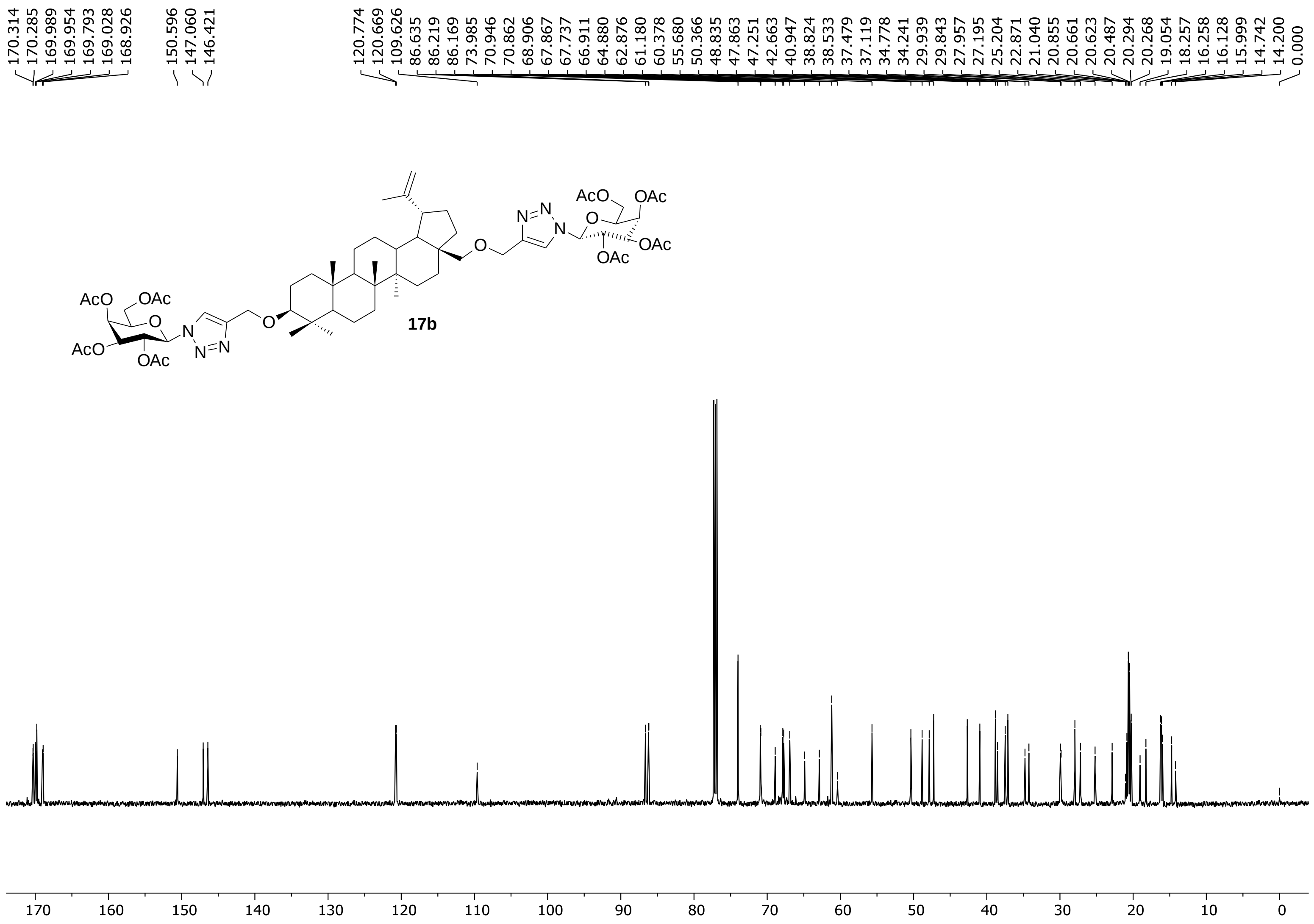

^{13}C NMR spectrum of glycoconjugates type **III** (**17b**); 150 MHz/CDCl$_3$/TMS; δ (ppm).

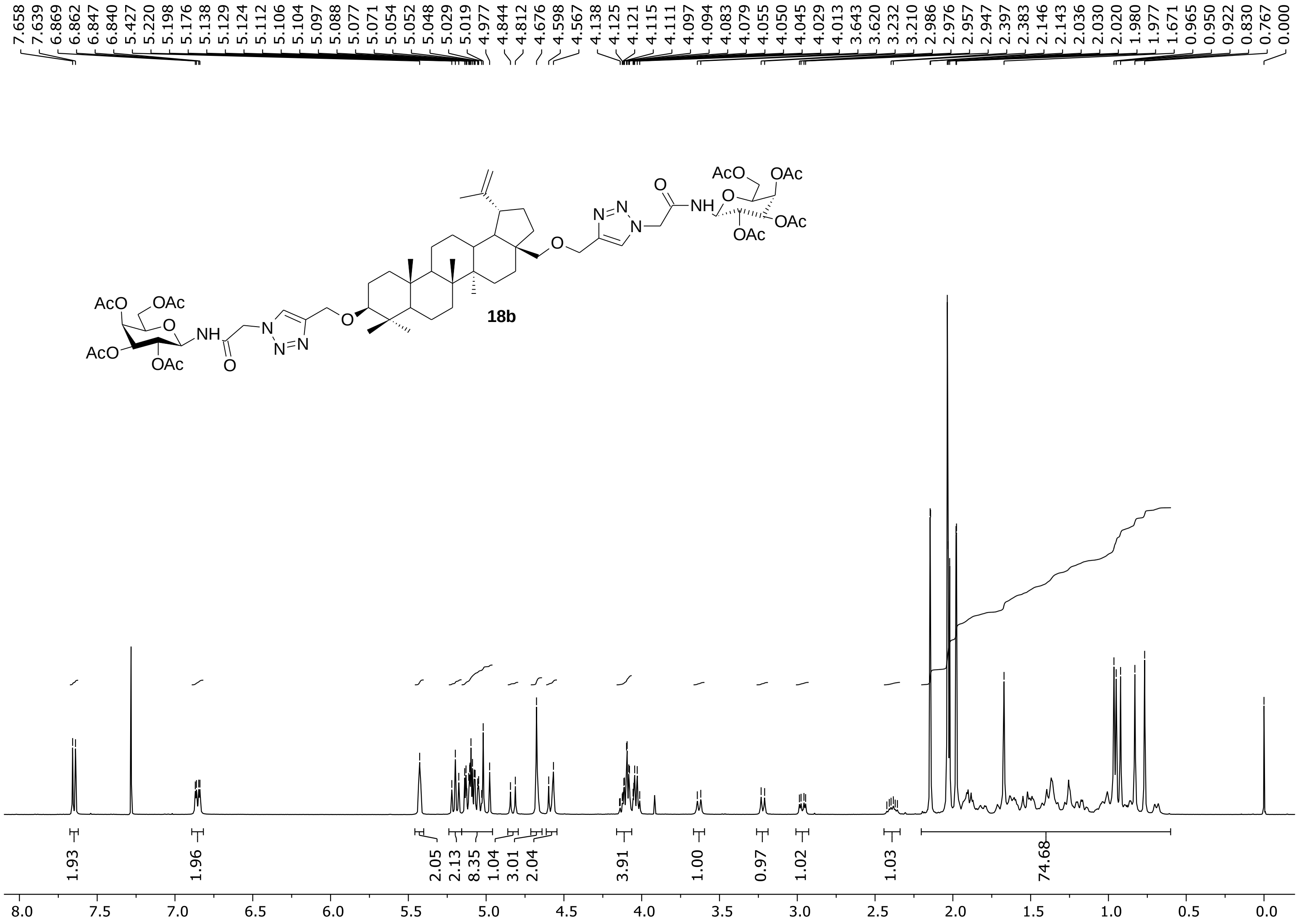

^{1}H NMR spectrum of glycoconjugates type **IV** (**18b**); 600 MHz/$CDCl_3$/TMS; δ (ppm).

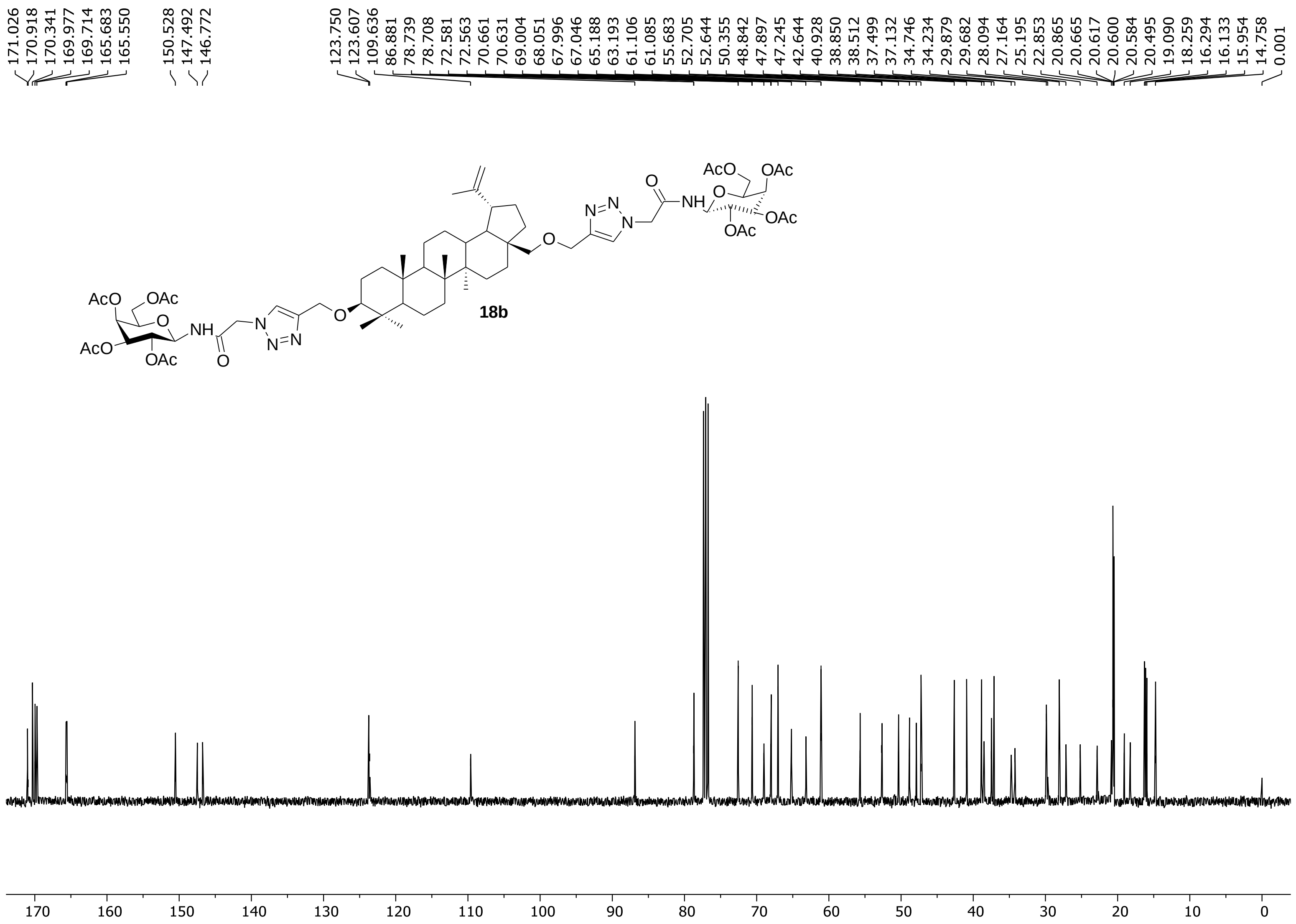

^{13}C NMR spectrum of glycoconjugates type **IV** (**18b**); 150 MHz/$CDCl_3$/TMS; δ (ppm).

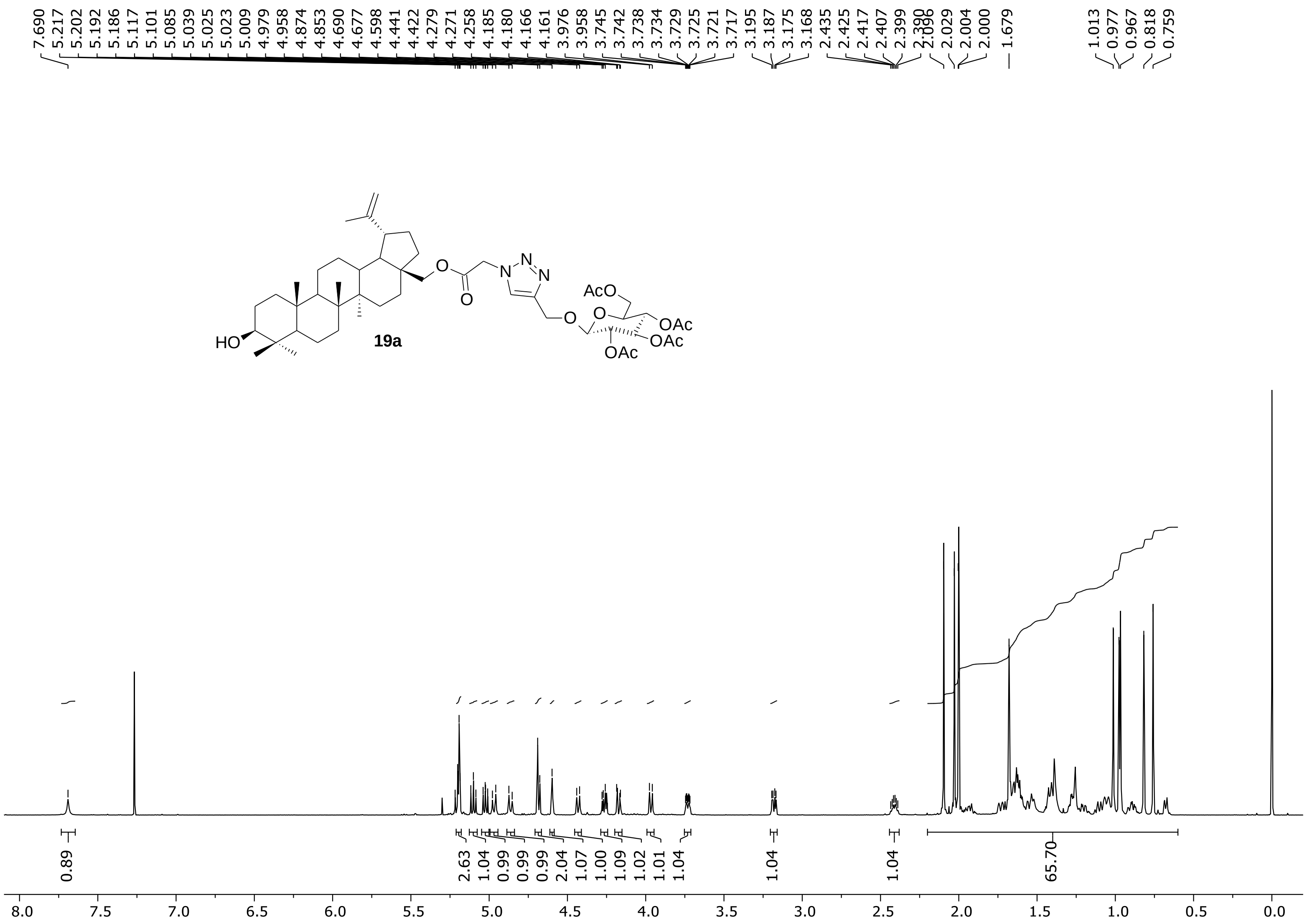

^{1}H NMR spectrum of glycoconjugates type **V** (**19a**); 600 MHz/$CDCl_3$/TMS; δ (ppm).

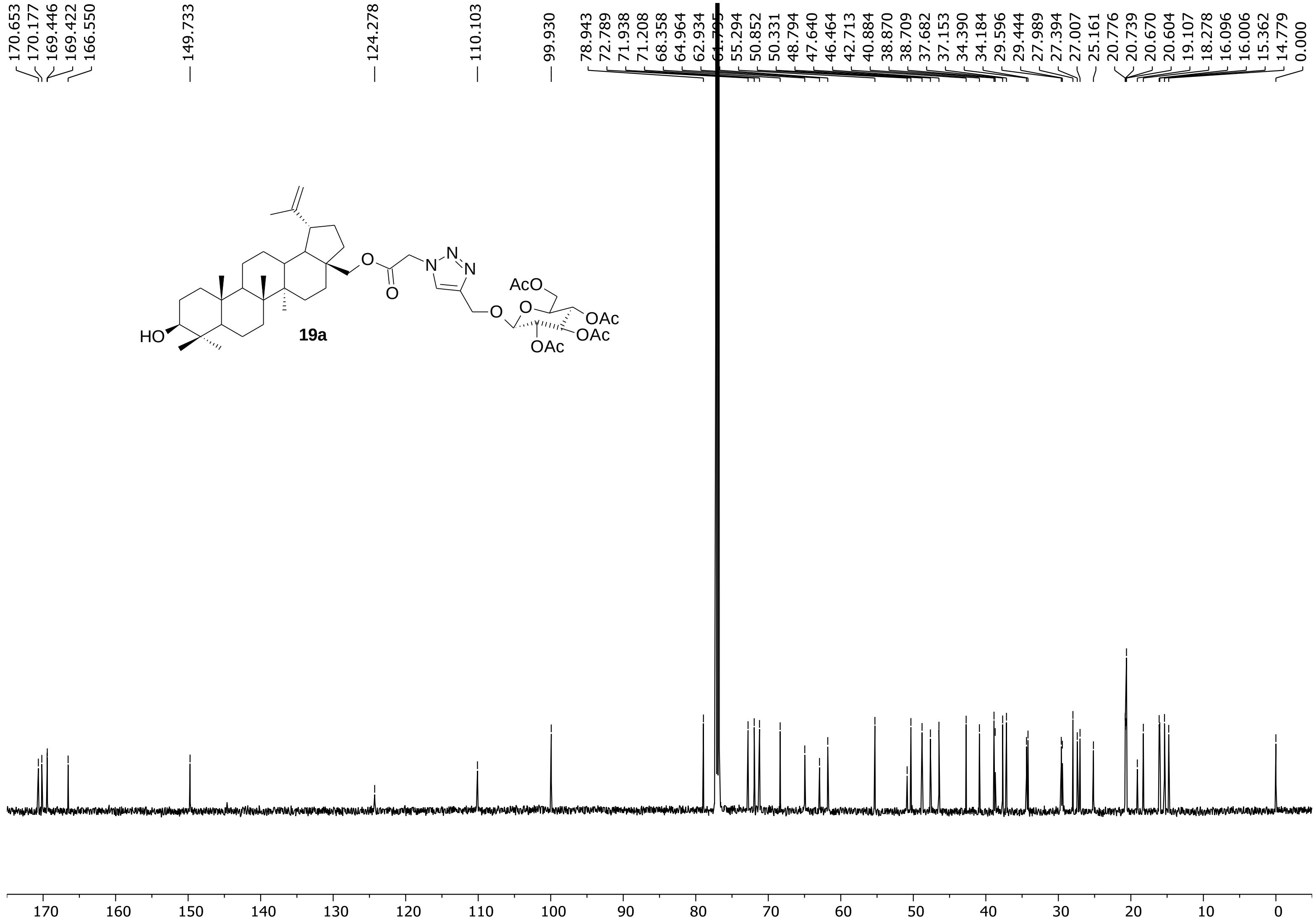

^{13}C NMR spectrum of glycoconjugates type **V** (**19a**); 150 MHz/$CDCl_3$/TMS; δ (ppm).

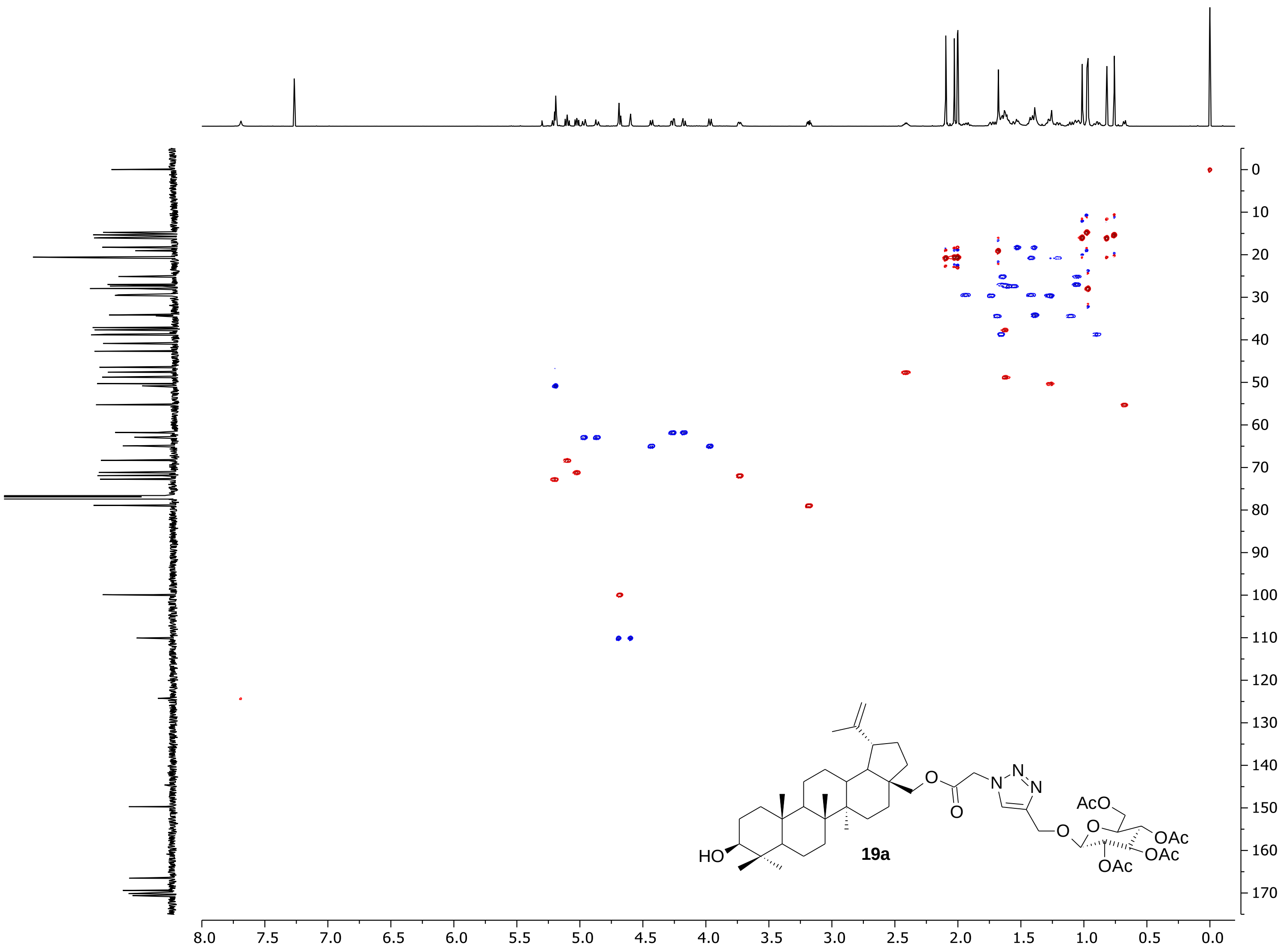

gHSQC spectrum of glycoconjugates type **V** (**19a**).

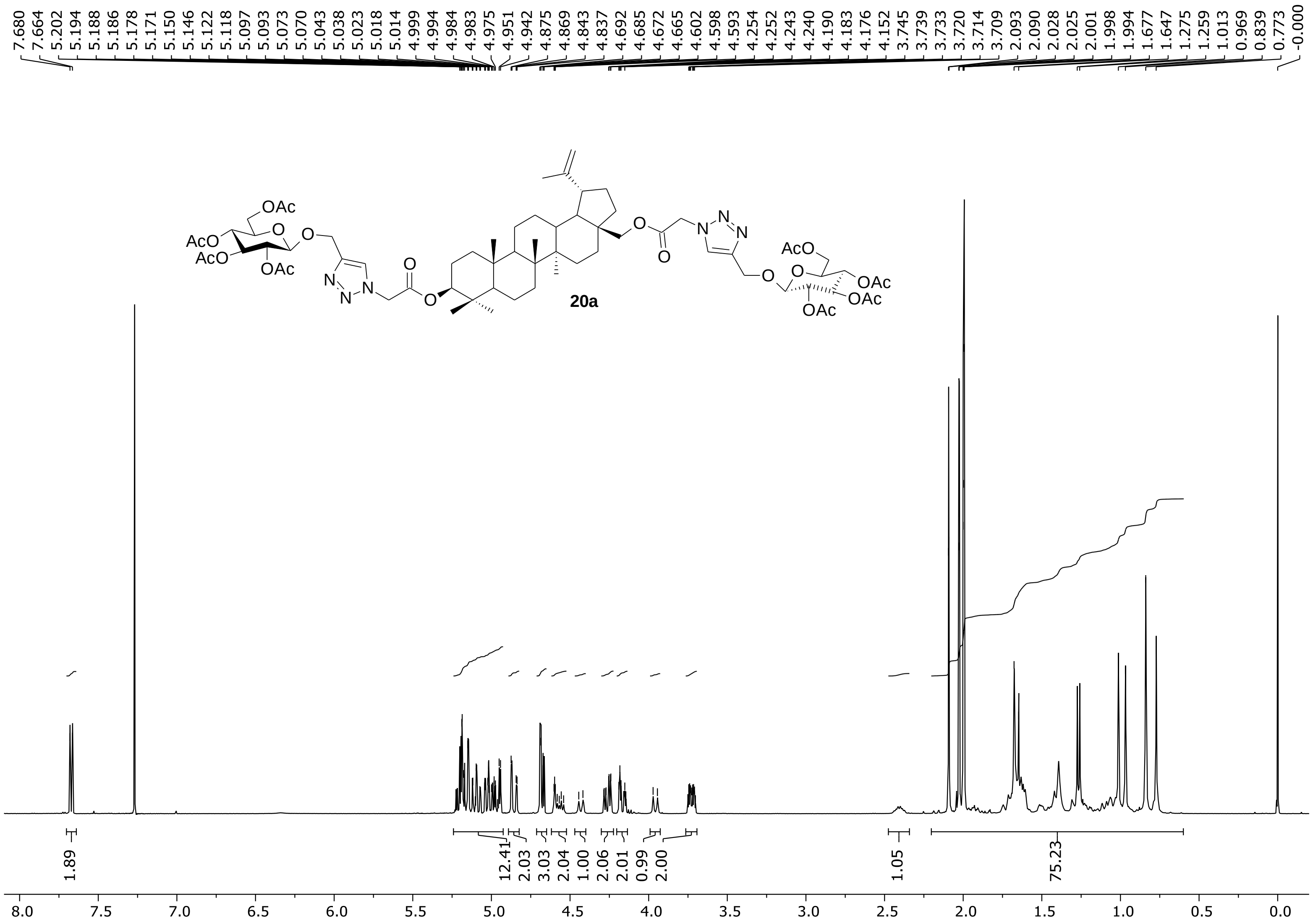

^{1}H NMR spectrum of glycoconjugates type **VI** (**20a**); 600 MHz/$CDCl_3$/TMS; δ (ppm).

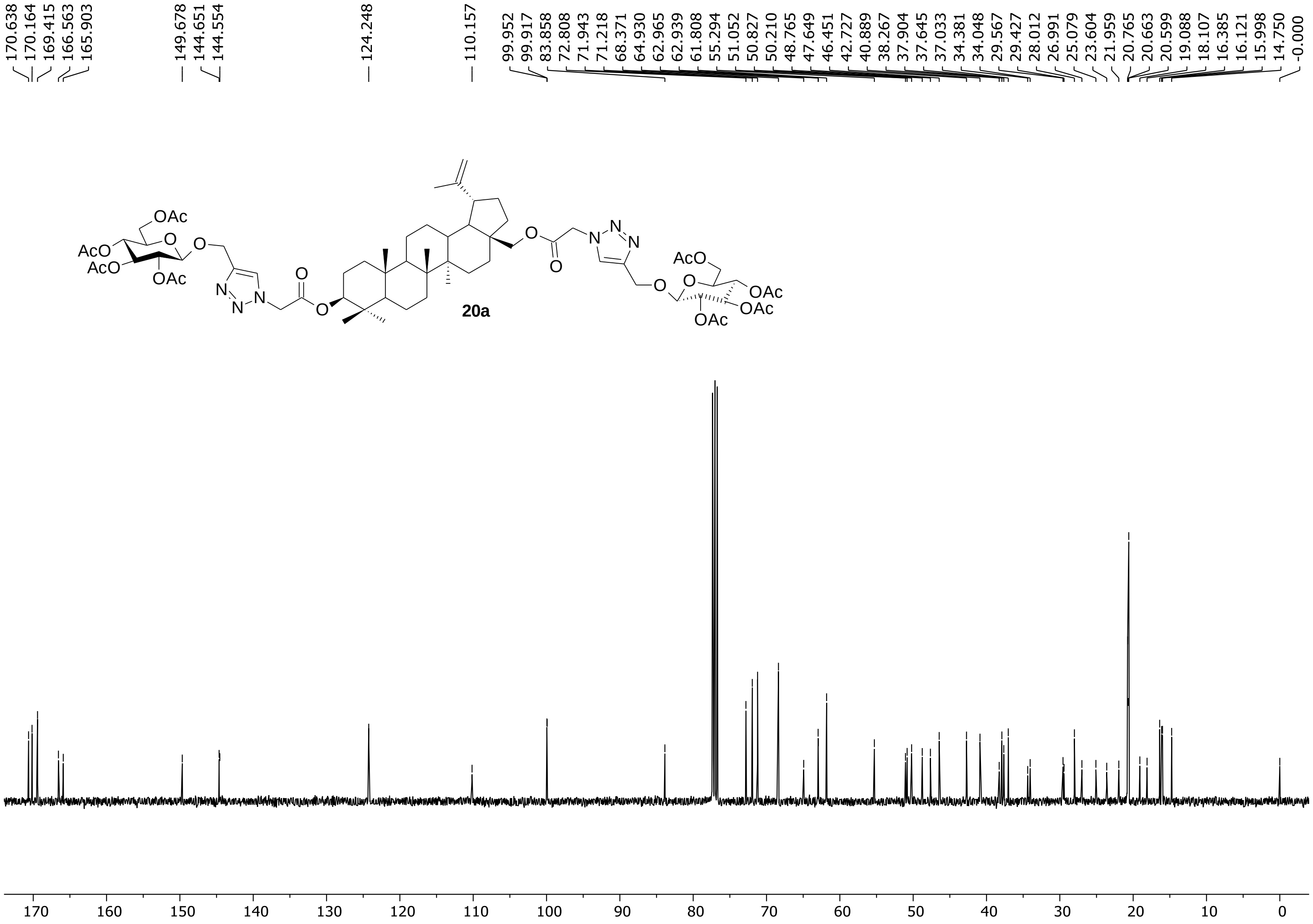

^{13}C NMR spectrum of glycoconjugates type **VI** (**20a**); 150 MHz/$CDCl_3$/TMS; δ (ppm).

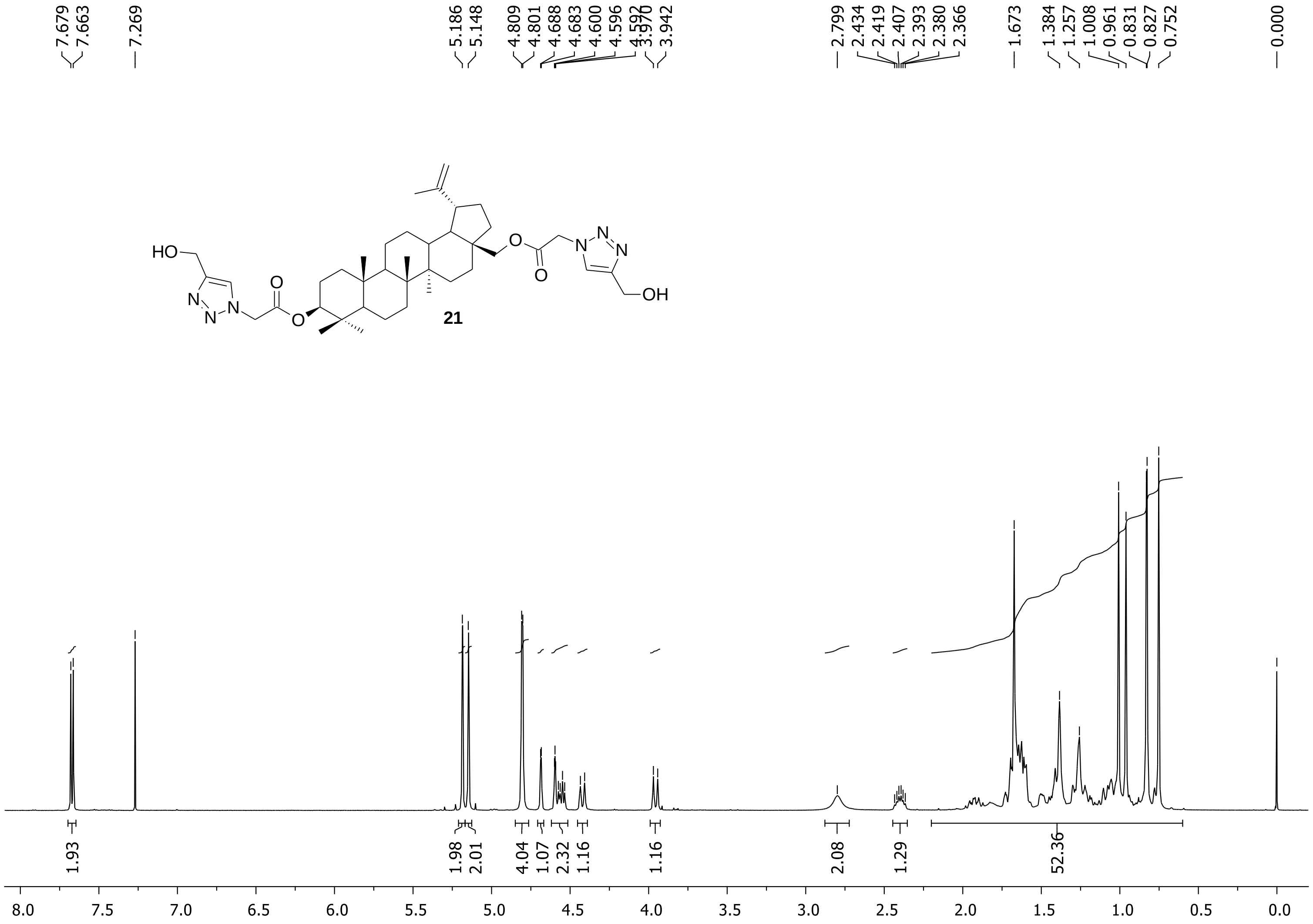

^{1}H NMR spectrum of compound (**21**); 600 MHz/$CDCl_3$/TMS; δ (ppm).

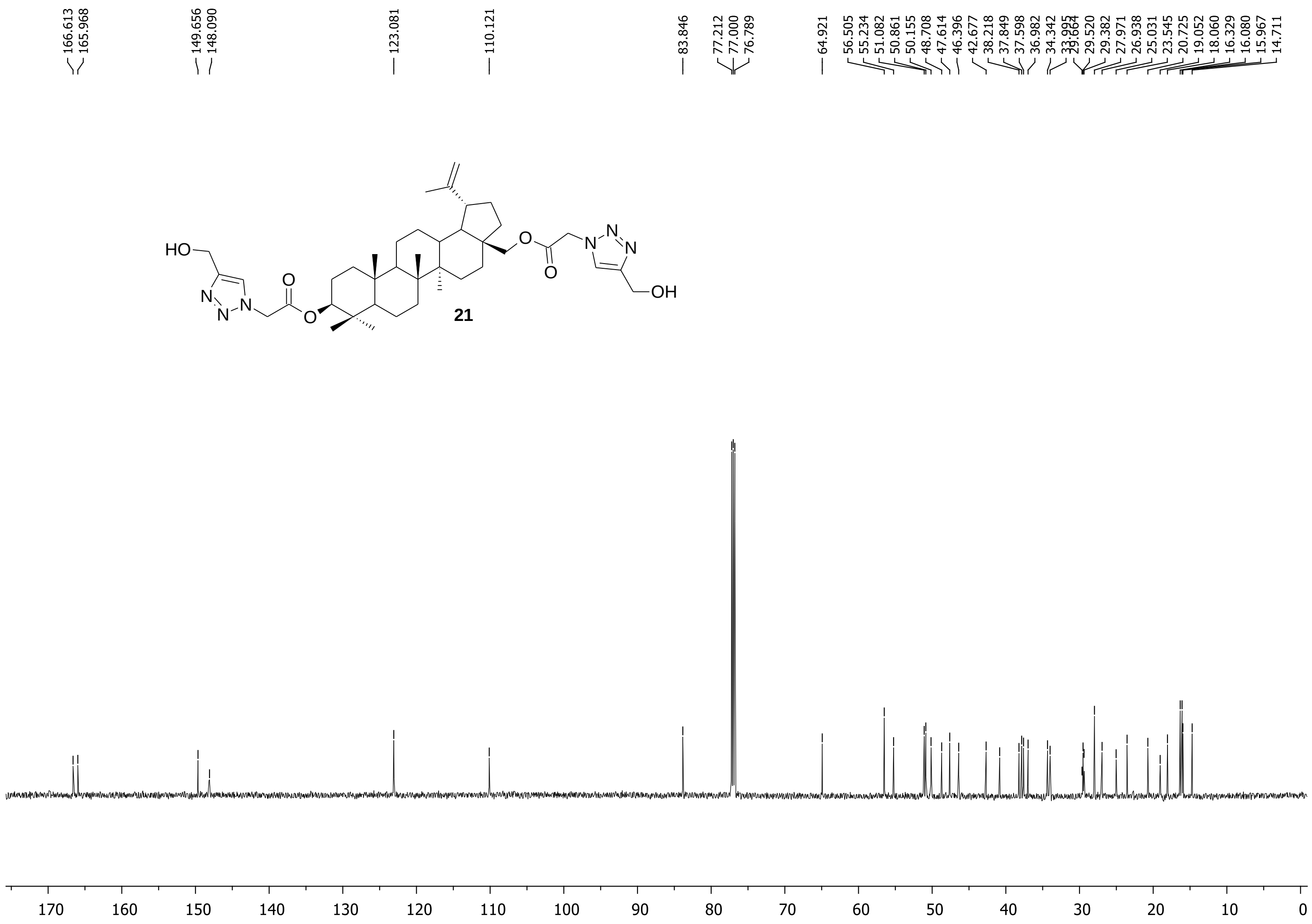

^{13}C NMR spectrum of of compound (**21**); 150 MHz/$CDCl_3$/TMS; δ (ppm)

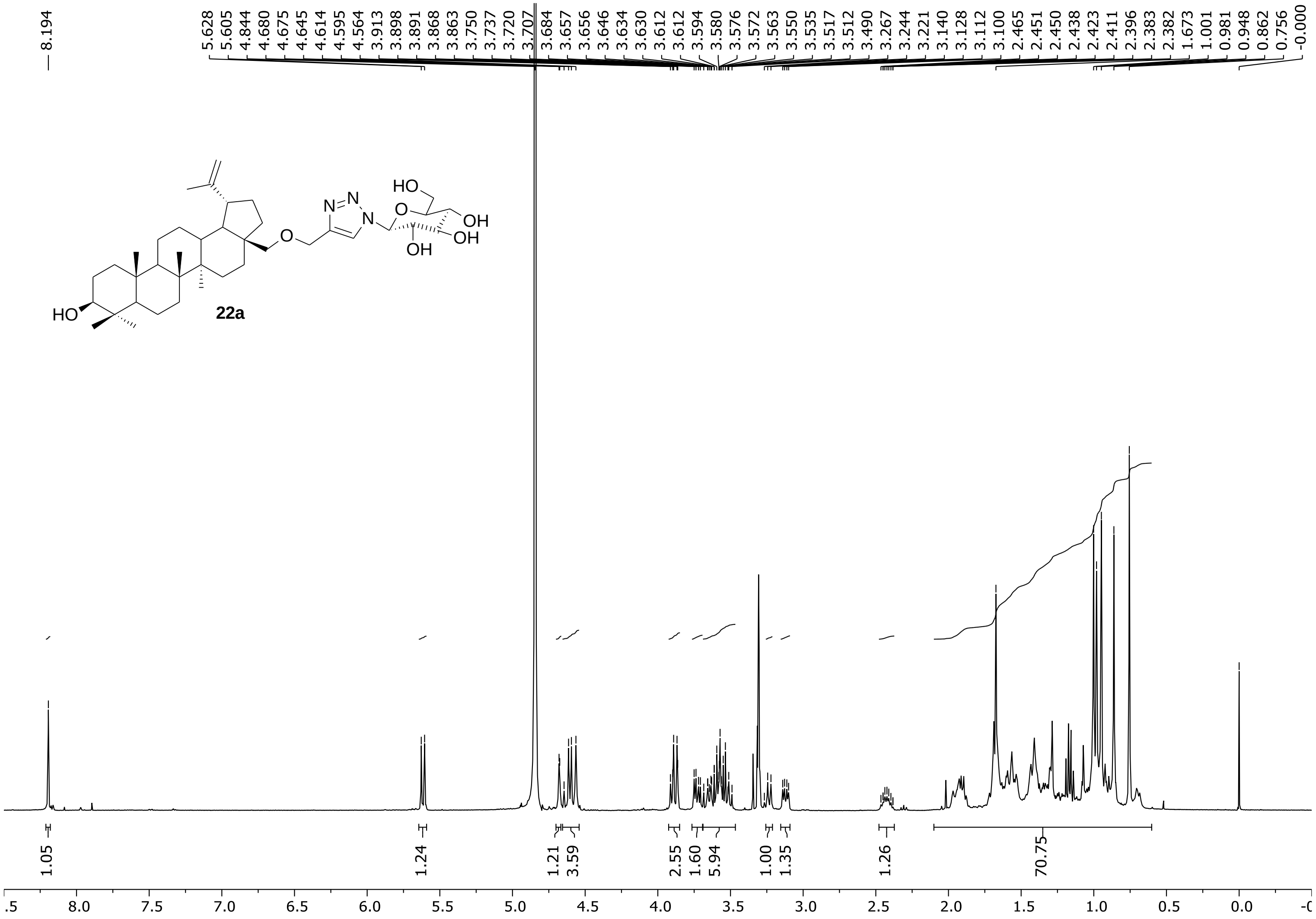

^{1}H NMR spectrum of glycoconjugates type **I** (**22a**); 400 MHz/MeOD/TMS; δ (ppm).

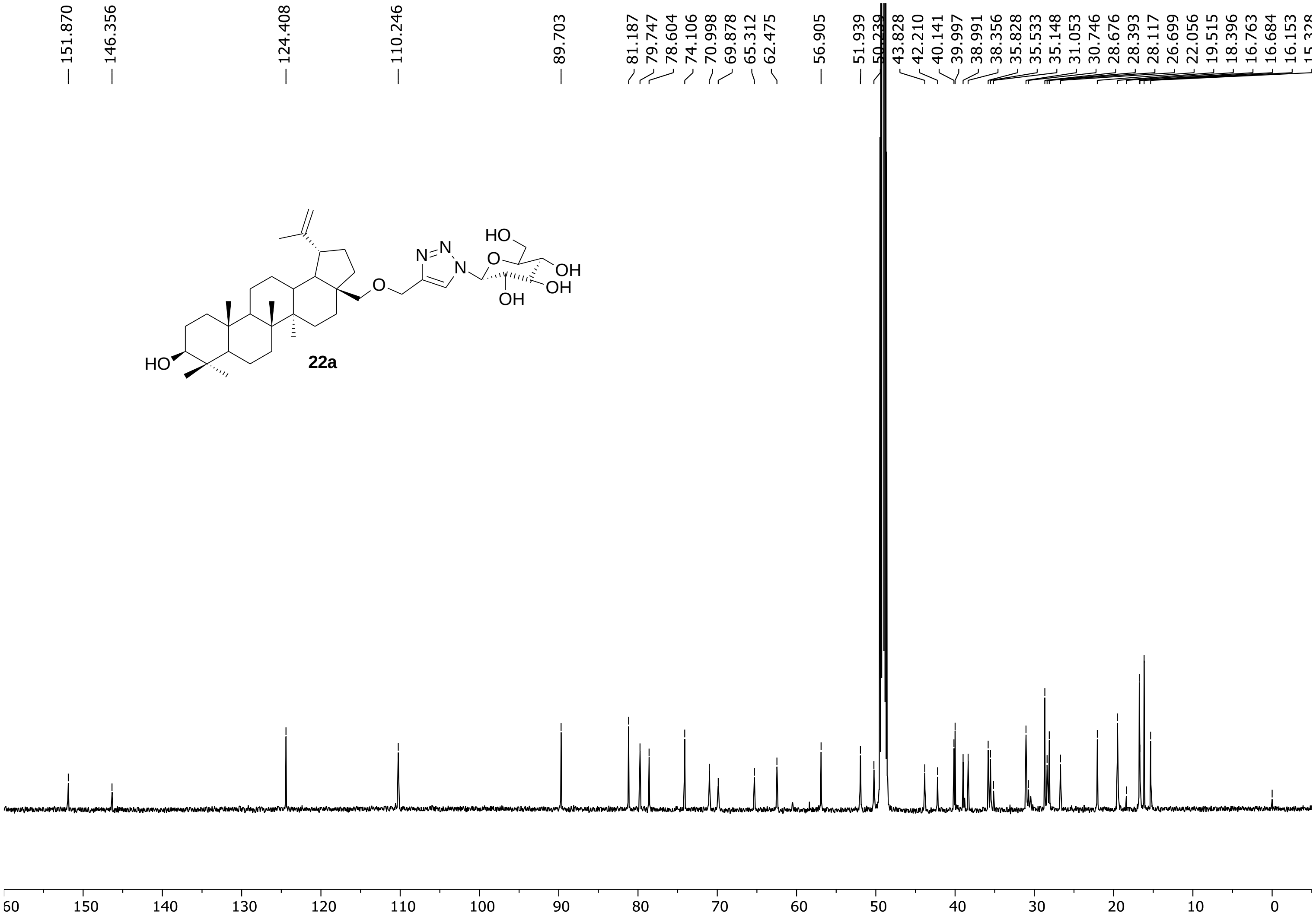

^{13}C NMR spectrum of glycoconjugates type **I** (**22a**); 150 MHz/ MeOD/TMS; δ (ppm).

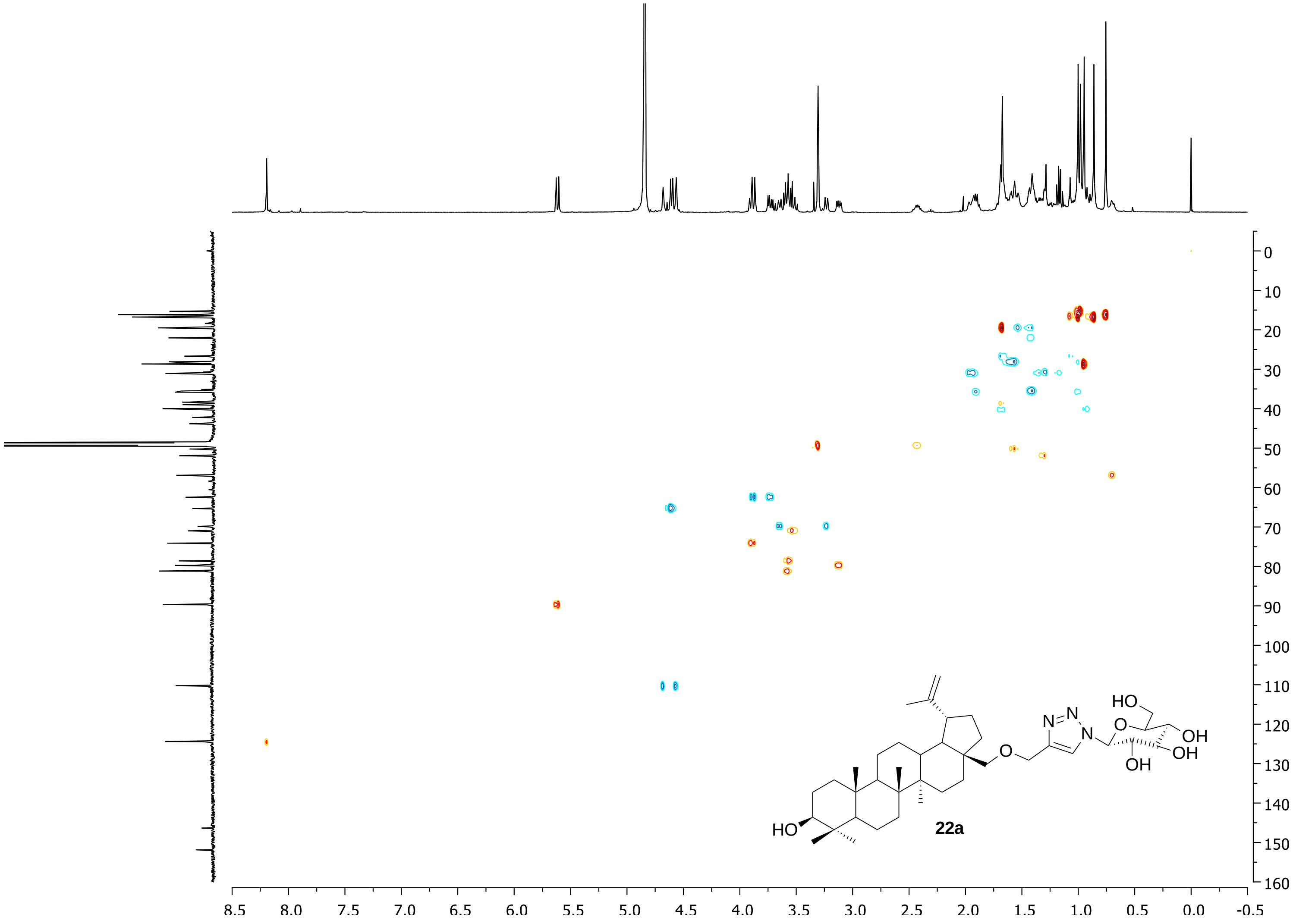

gHSQC spectrum of glycoconjugates type **I** (**22a**);

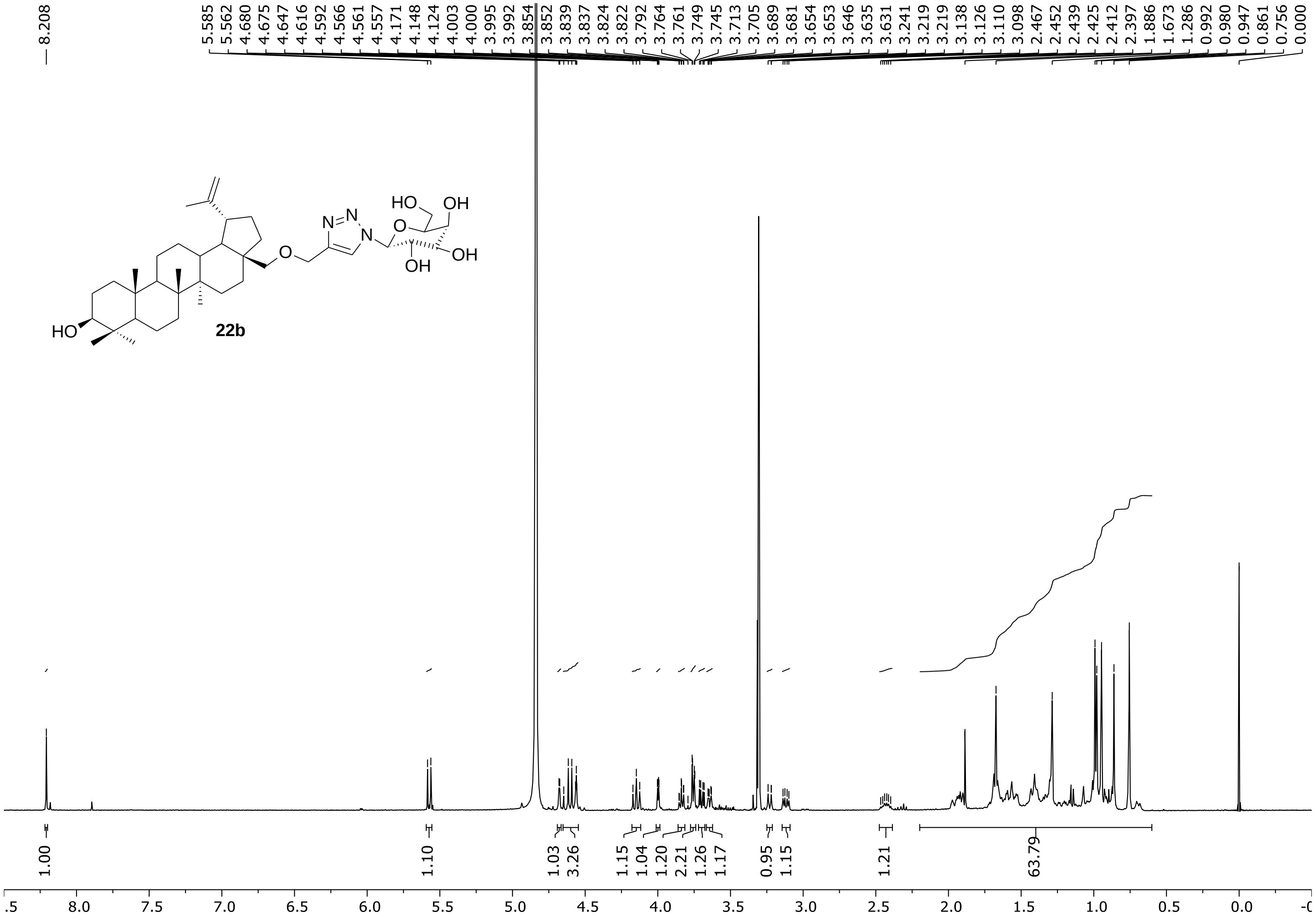

^{1}H NMR spectrum of glycoconjugates type **I** (**22b**); 400 MHz/ MeOD/TMS; δ (ppm).

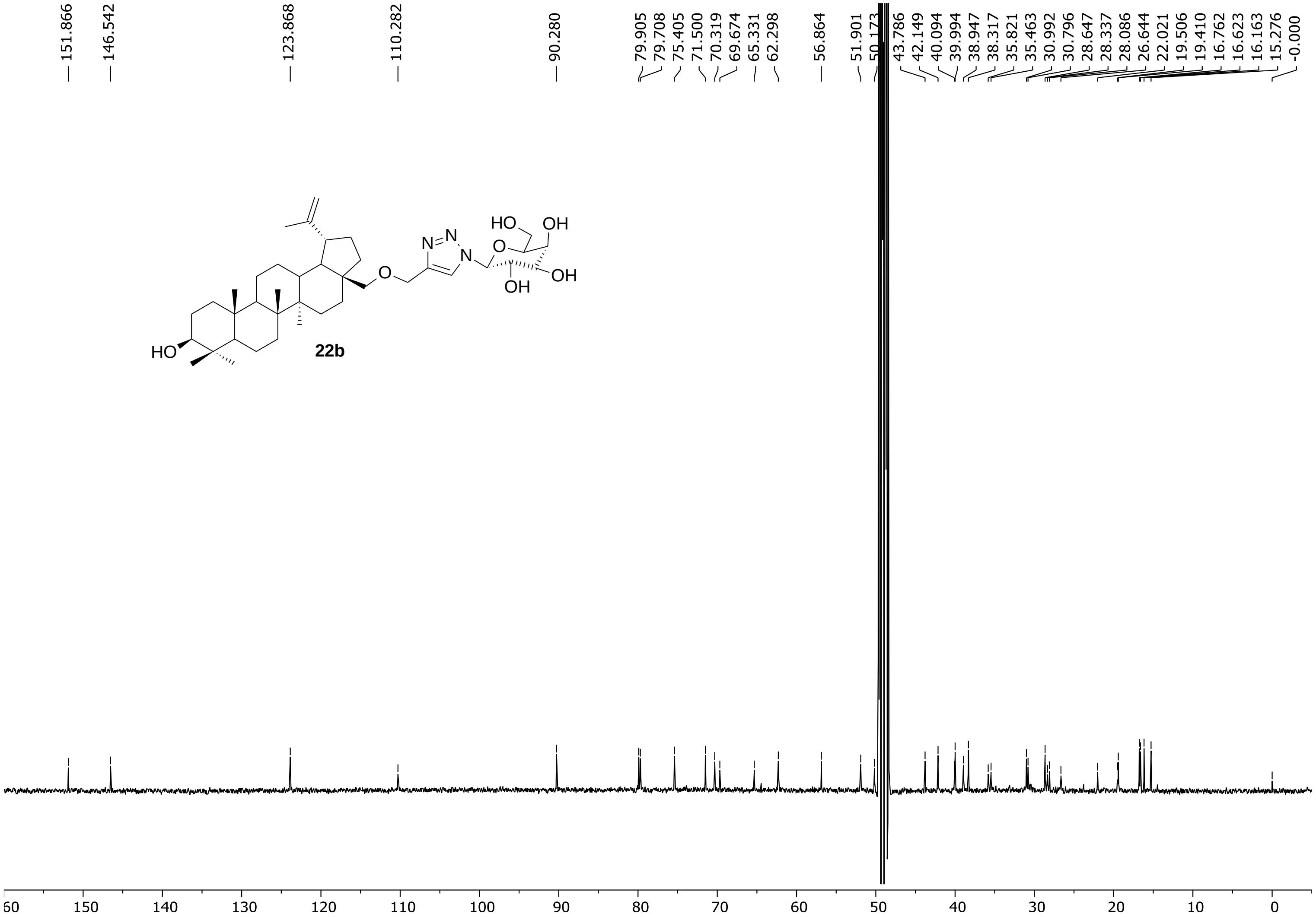

^{13}C NMR spectrum of glycoconjugates type **I** (**22b**); 150 MHz/ MeOD /TMS; δ (ppm).

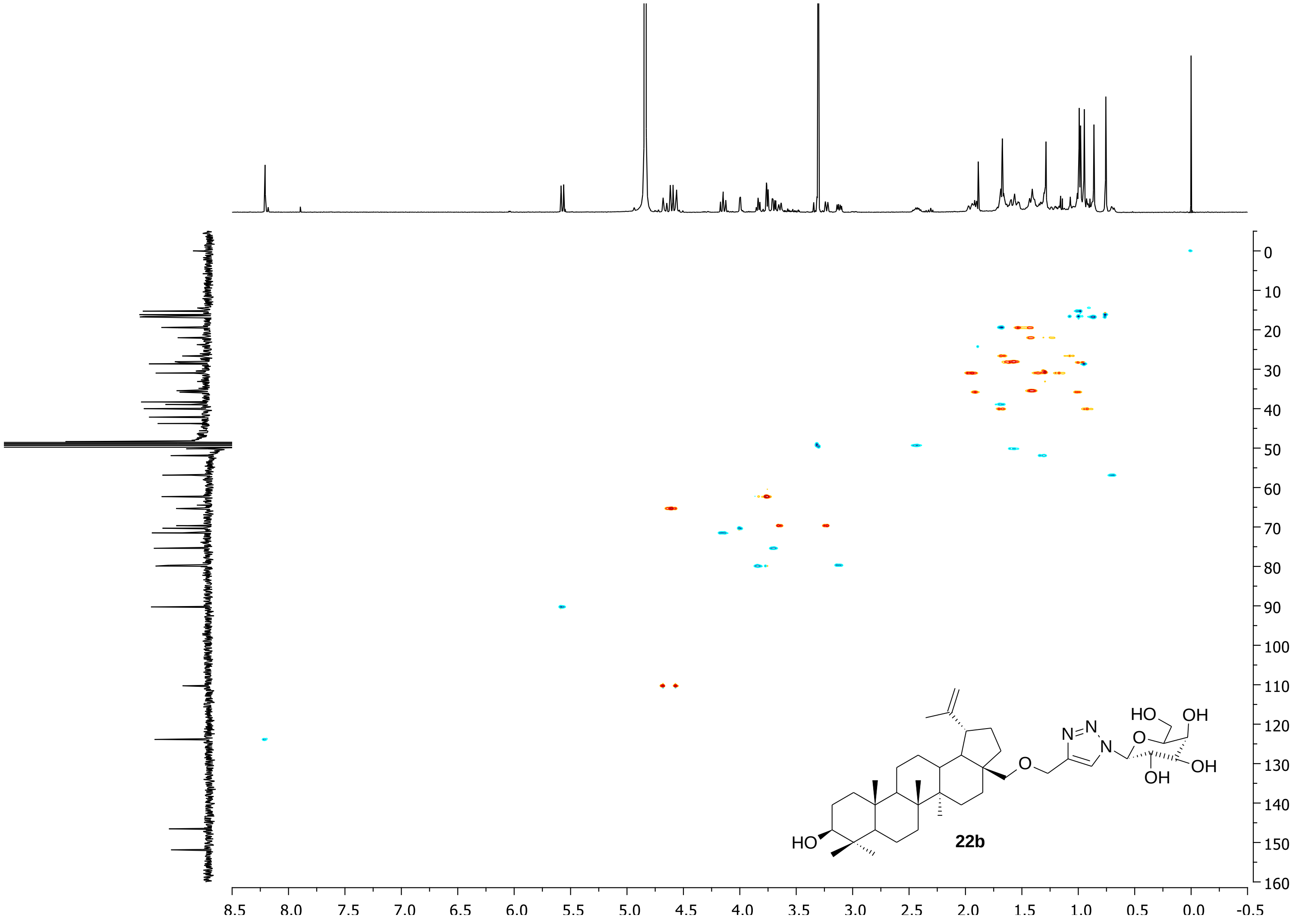

gHSQC spectrum of glycoconjugates type **I** (**22b**);

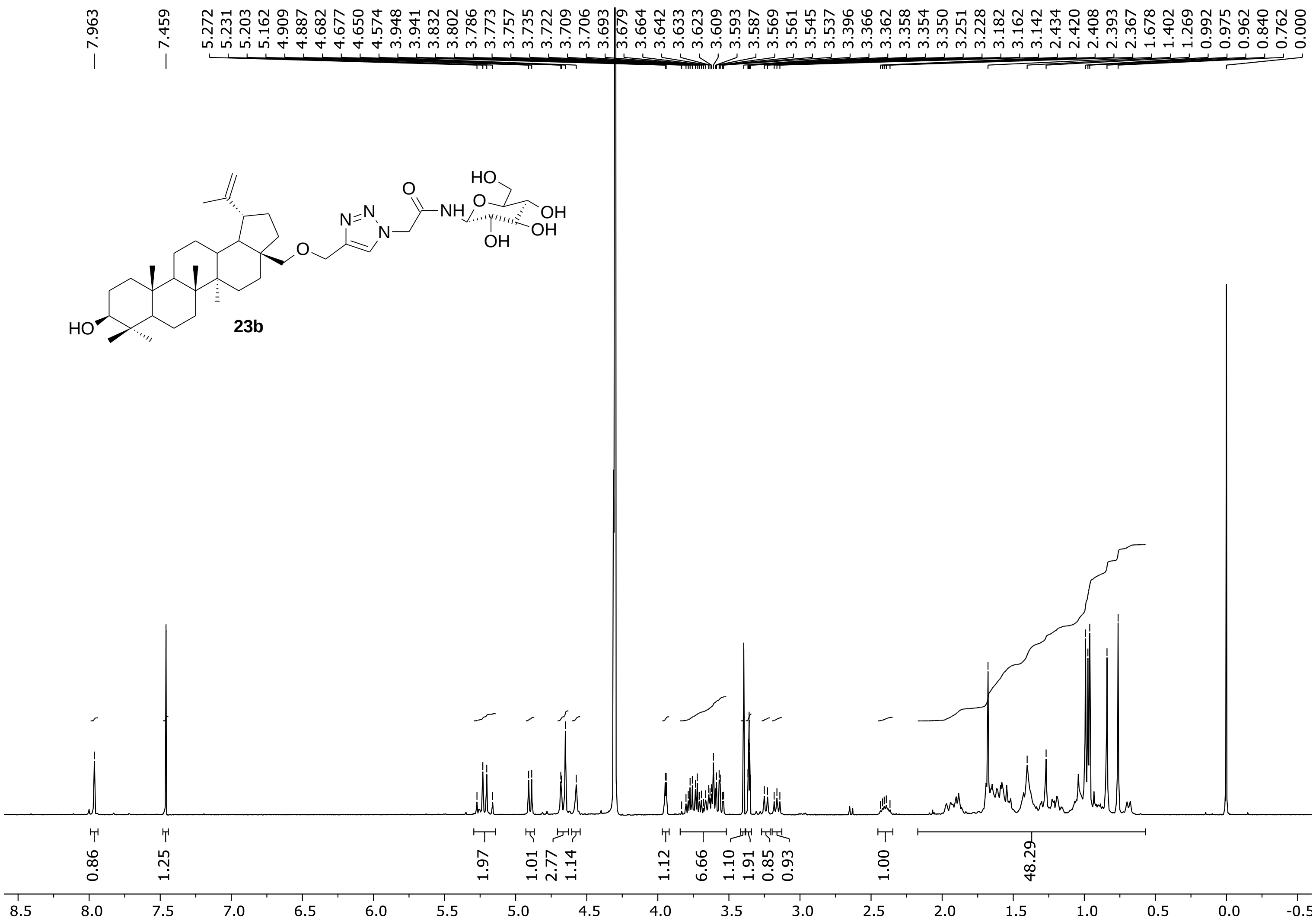

^{1}H NMR spectrum of glycoconjugates type **II** (**23b**); 400 MHz/ MeOD/TMS; δ (ppm).

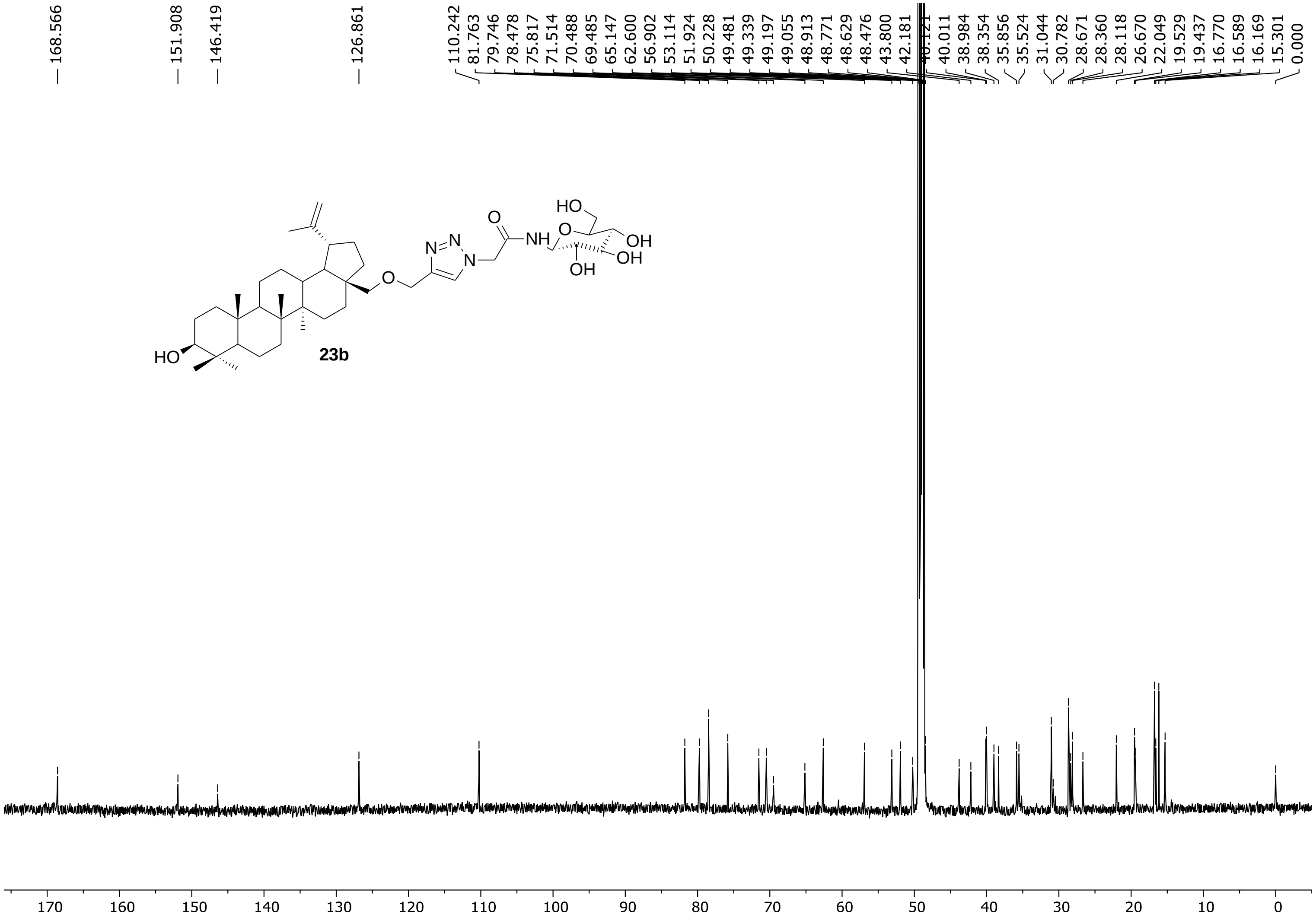

^{13}C NMR spectrum of glycoconjugates type **II** (**23b**); 150 MHz/ MeOD/TMS; δ (ppm).

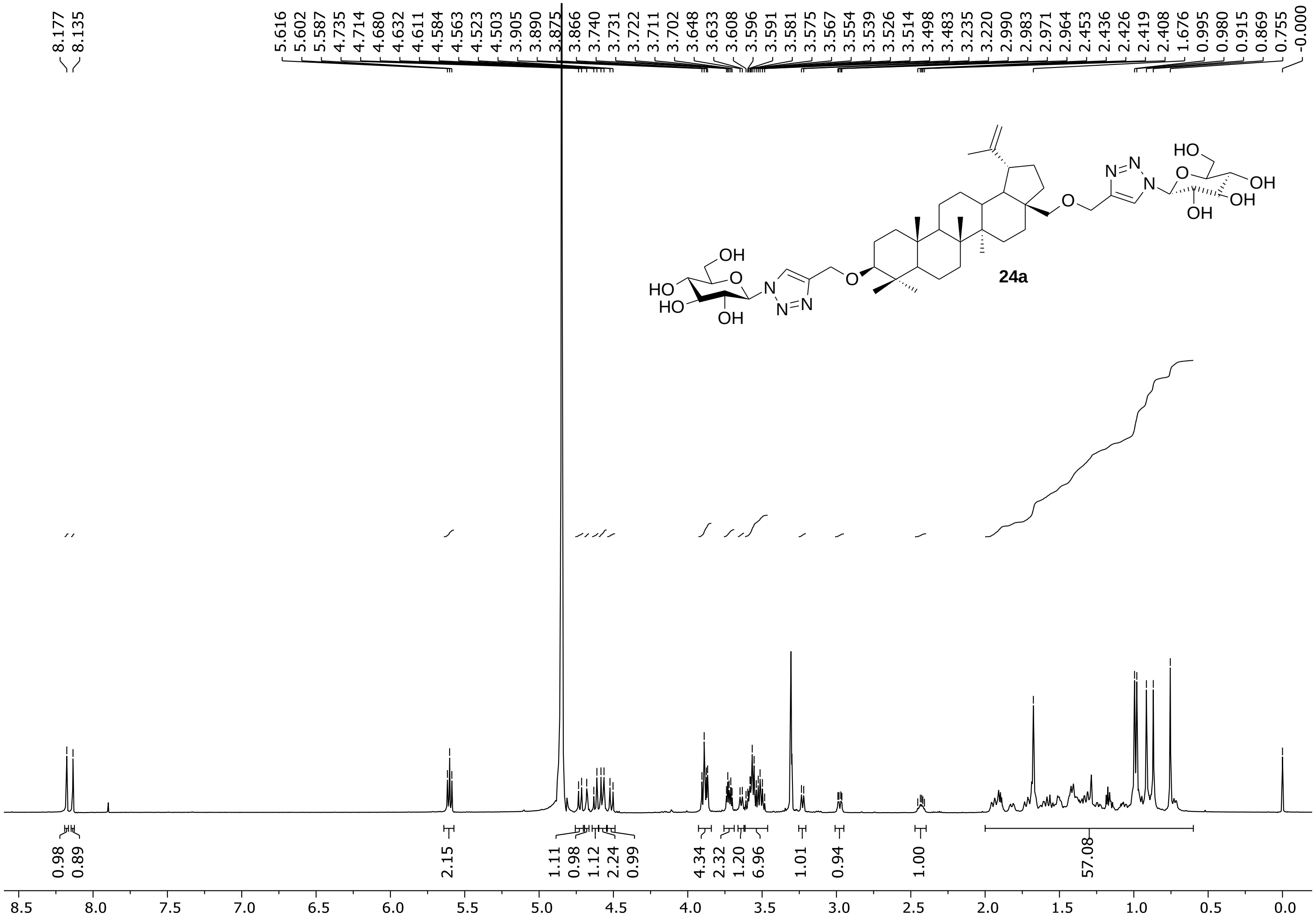

^{1}H NMR spectrum of glycoconjugates type **III** (**24a**); 600 MHz/ MeOD/TMS; δ (ppm).

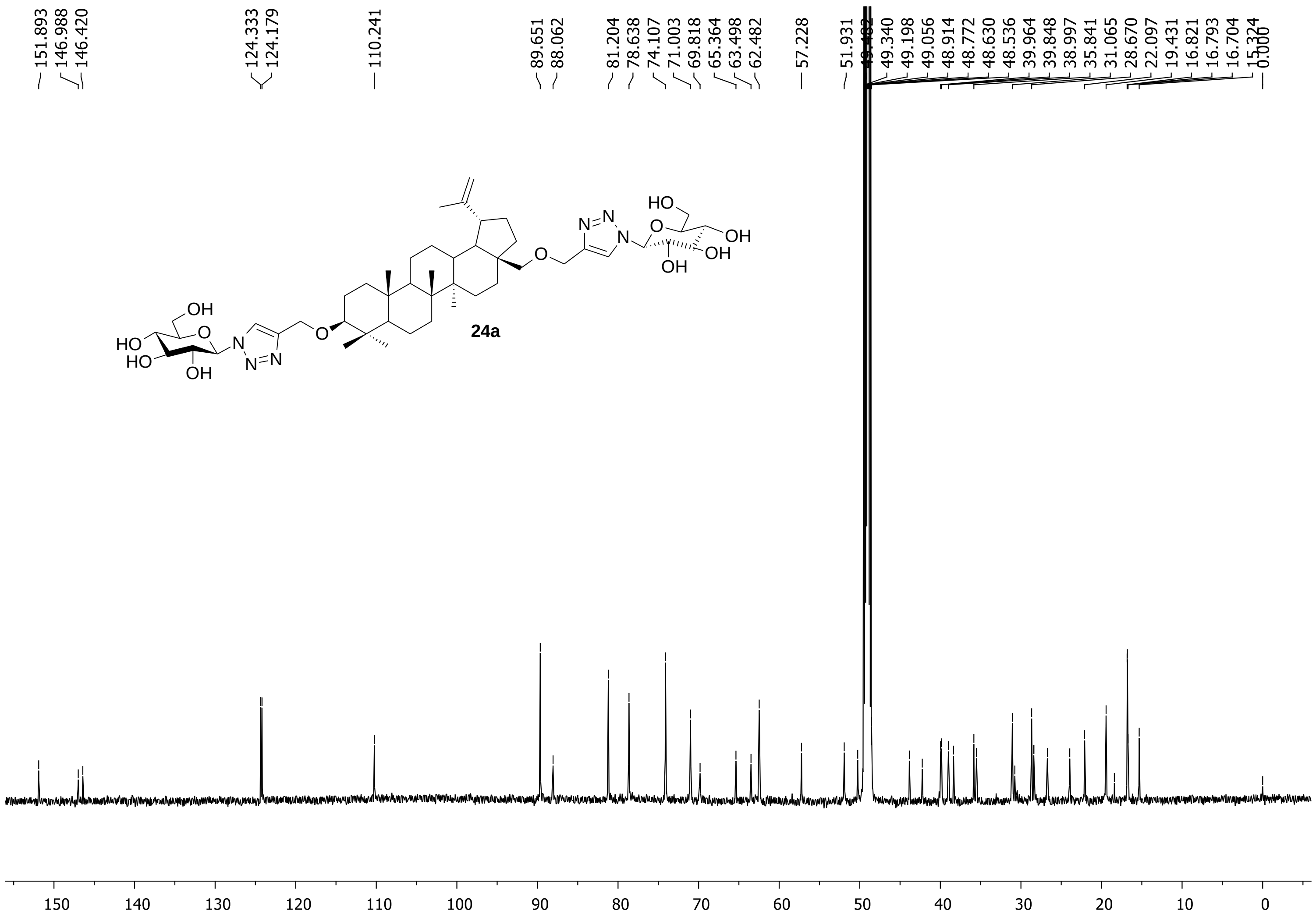

^{13}C NMR spectrum of glycoconjugates type **III** (**24a**); 150 MHz/ MeOD/TMS; δ (ppm).

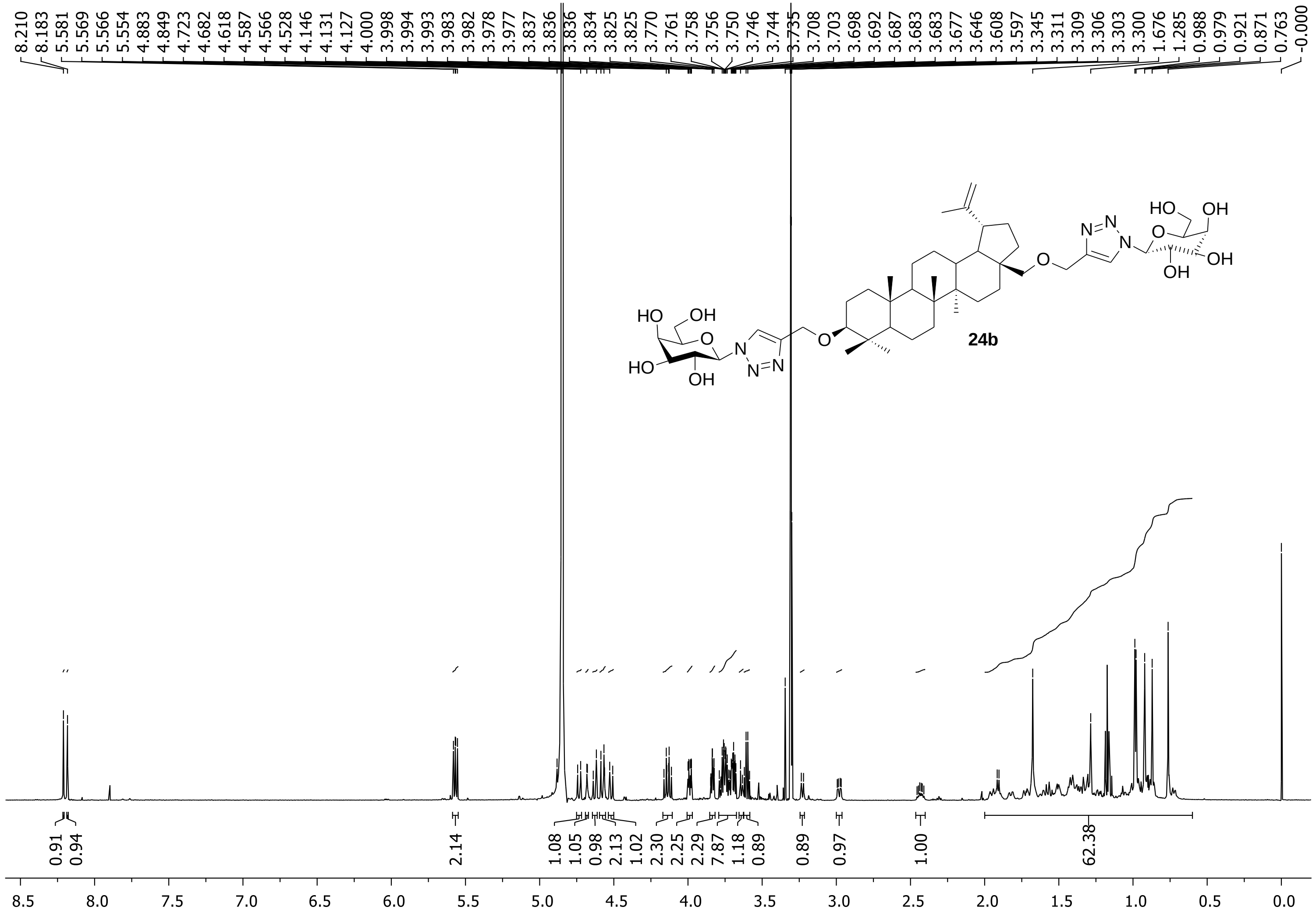

^{1}H NMR spectrum of glycoconjugates type **III** (**24b**); 600 MHz/ MeOD/TMS; δ (ppm).

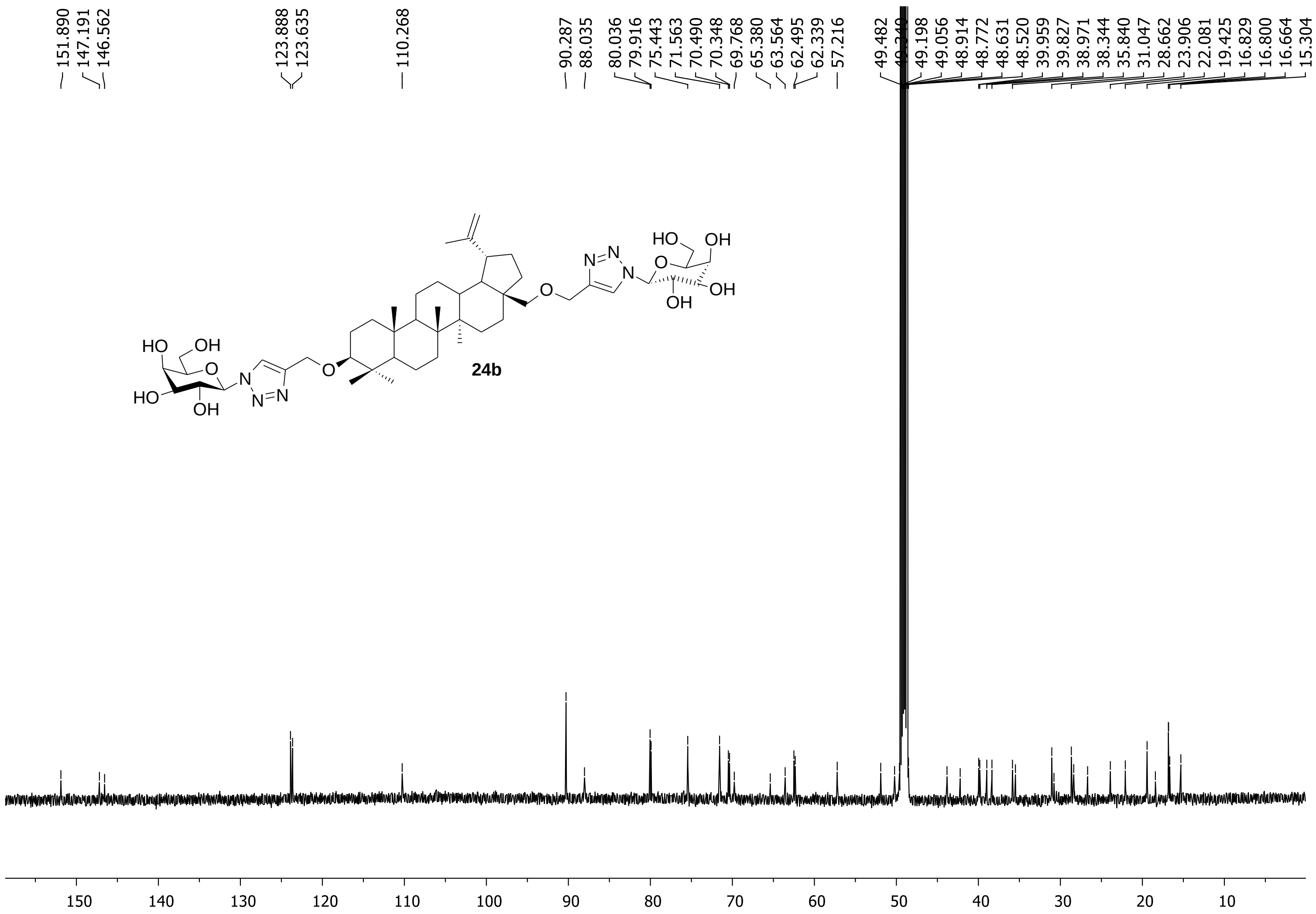

^{13}C NMR spectrum of glycoconjugates type **III** (**24b**); 150 MHz/ MeOD/TMS; δ (ppm).

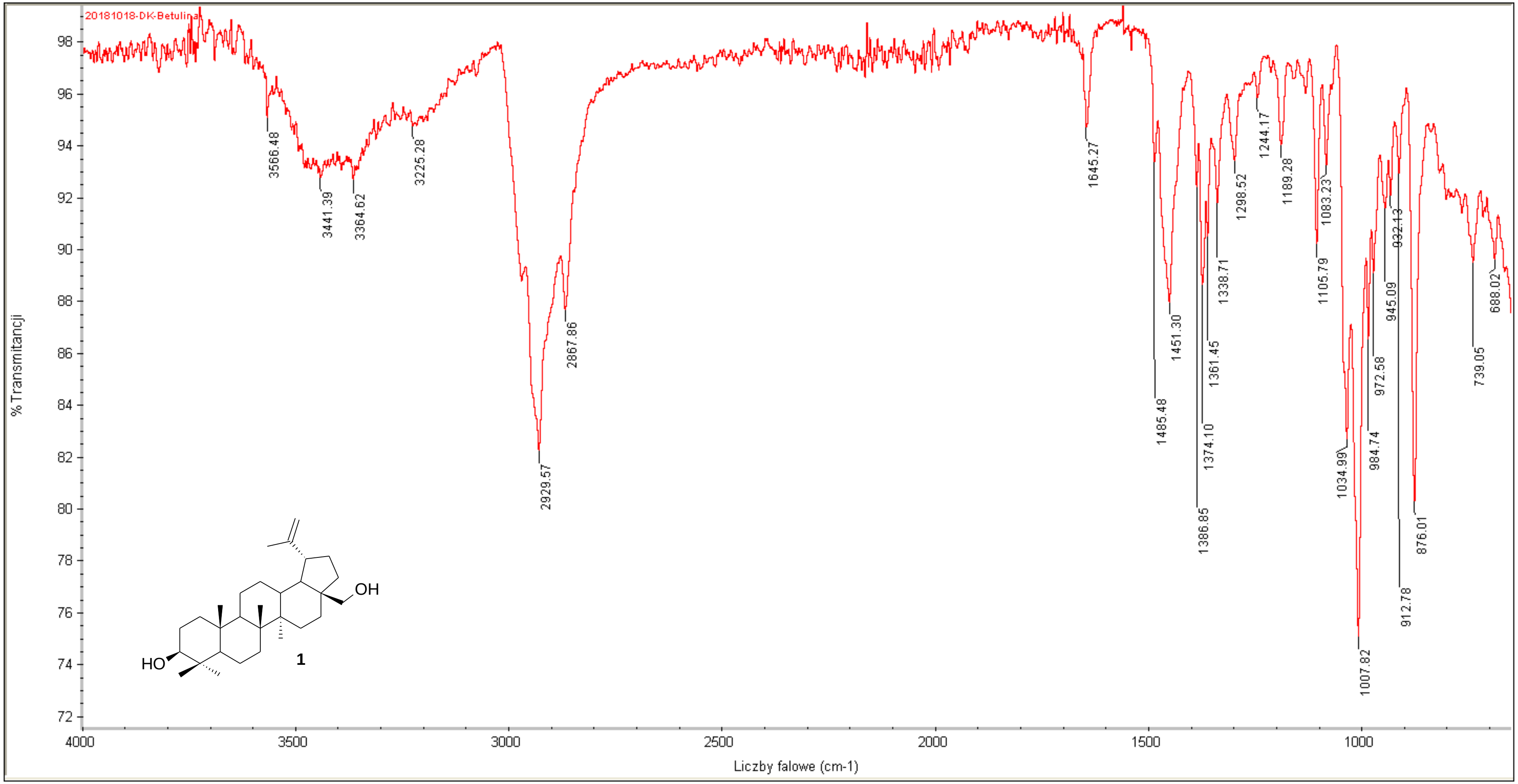

IR spectrum of betulin (**1**); ATR (cm^{-1}).

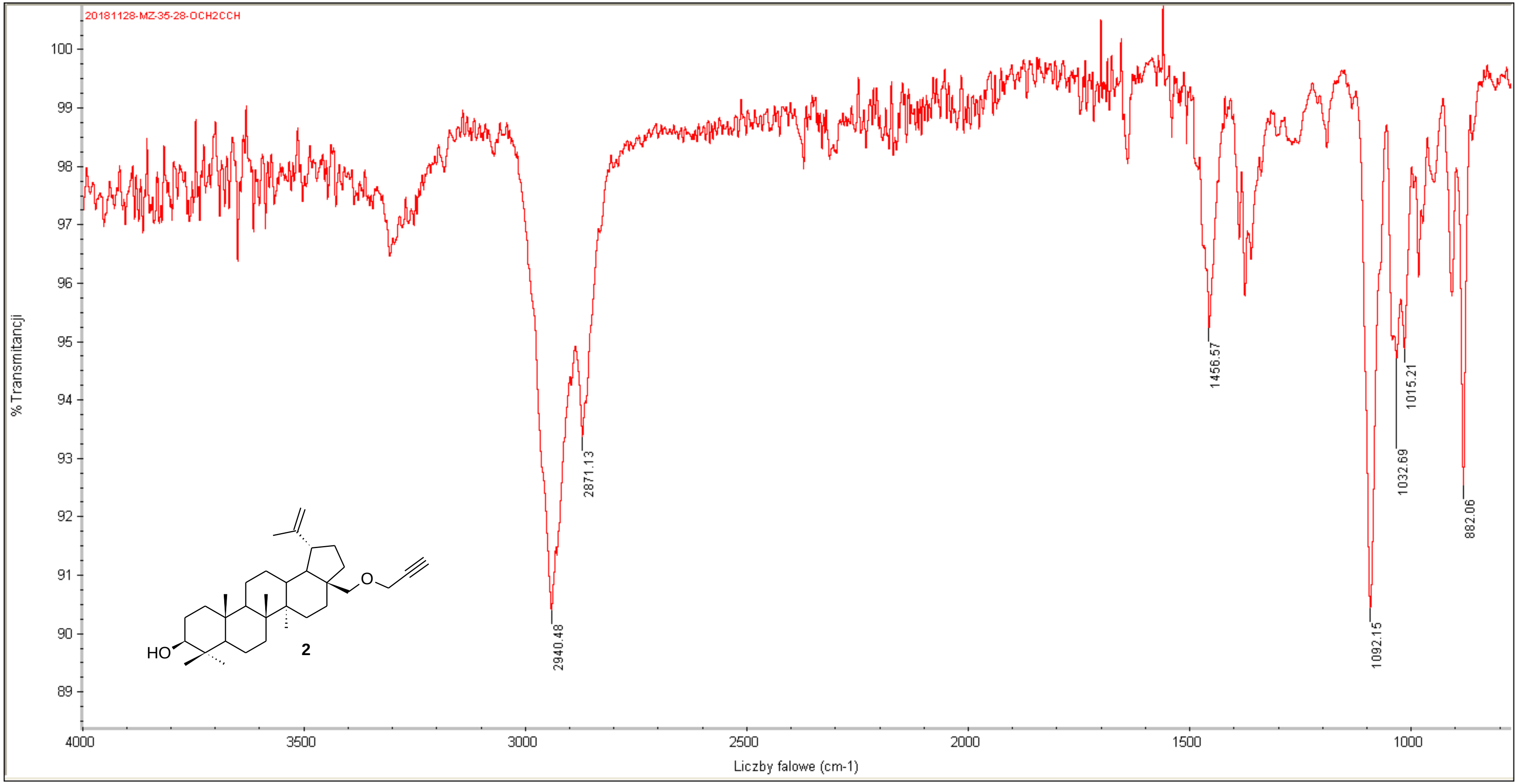

IR spectrum of 28-*O*-propargylbetulin (**2**); ATR (cm^{-1}).

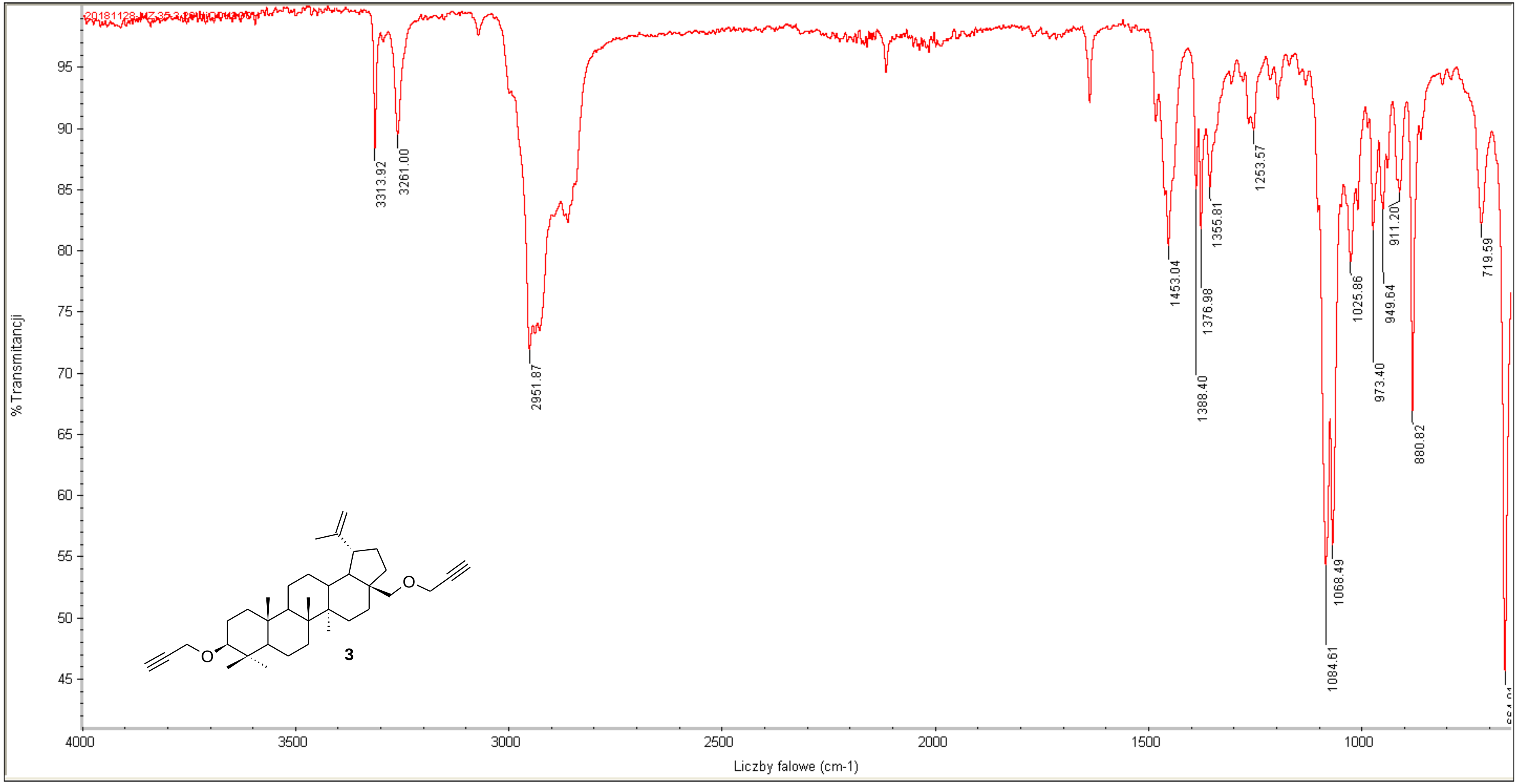

IR spectrum of 3,28-*O,O′*-dipropargylbetulin (**3**); ATR (cm^{-1}).

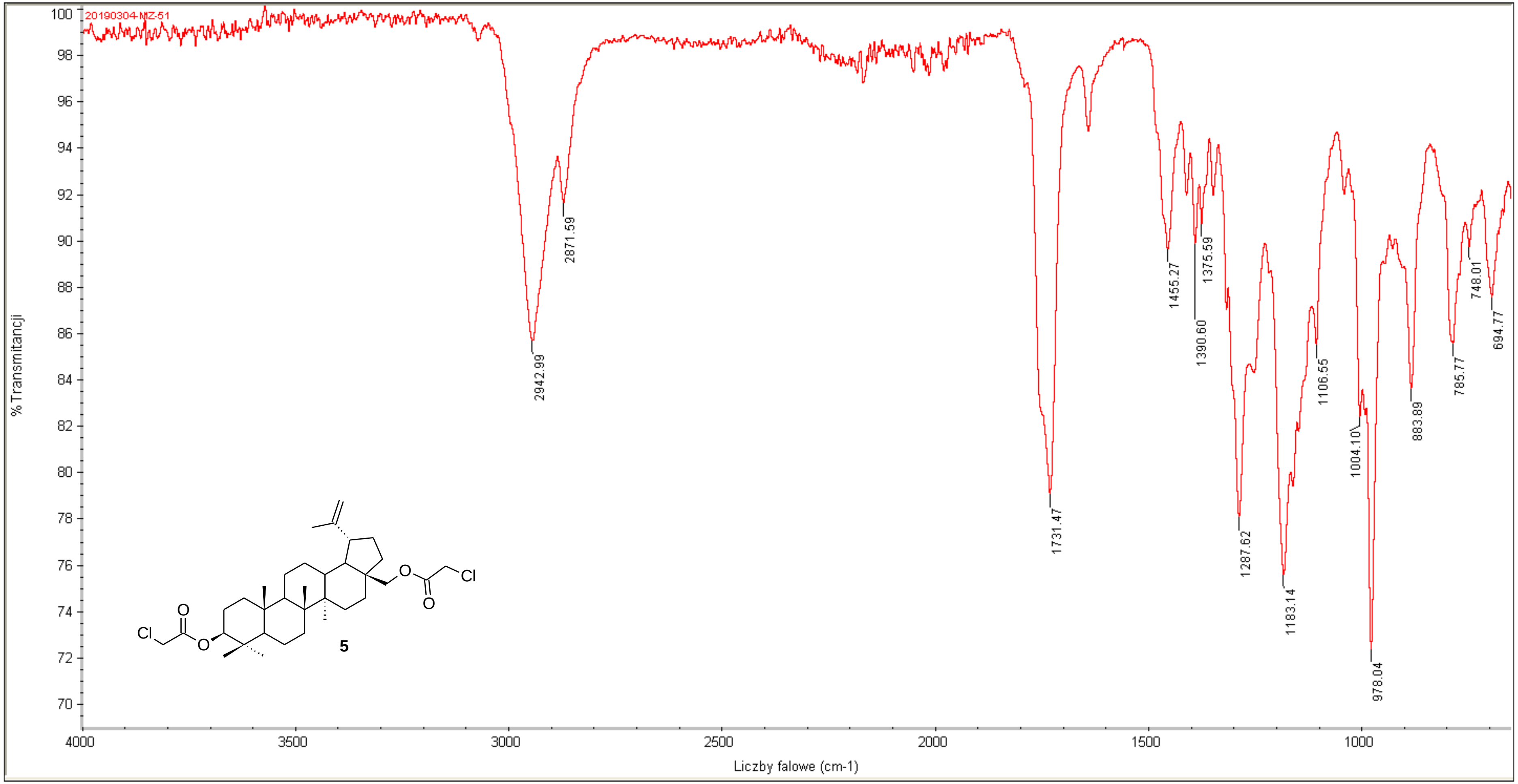

IR spectrum of 3,28-*O,O′*-di(2-chloroacetyl)betulin (**5**); ATR (cm^{-1}).

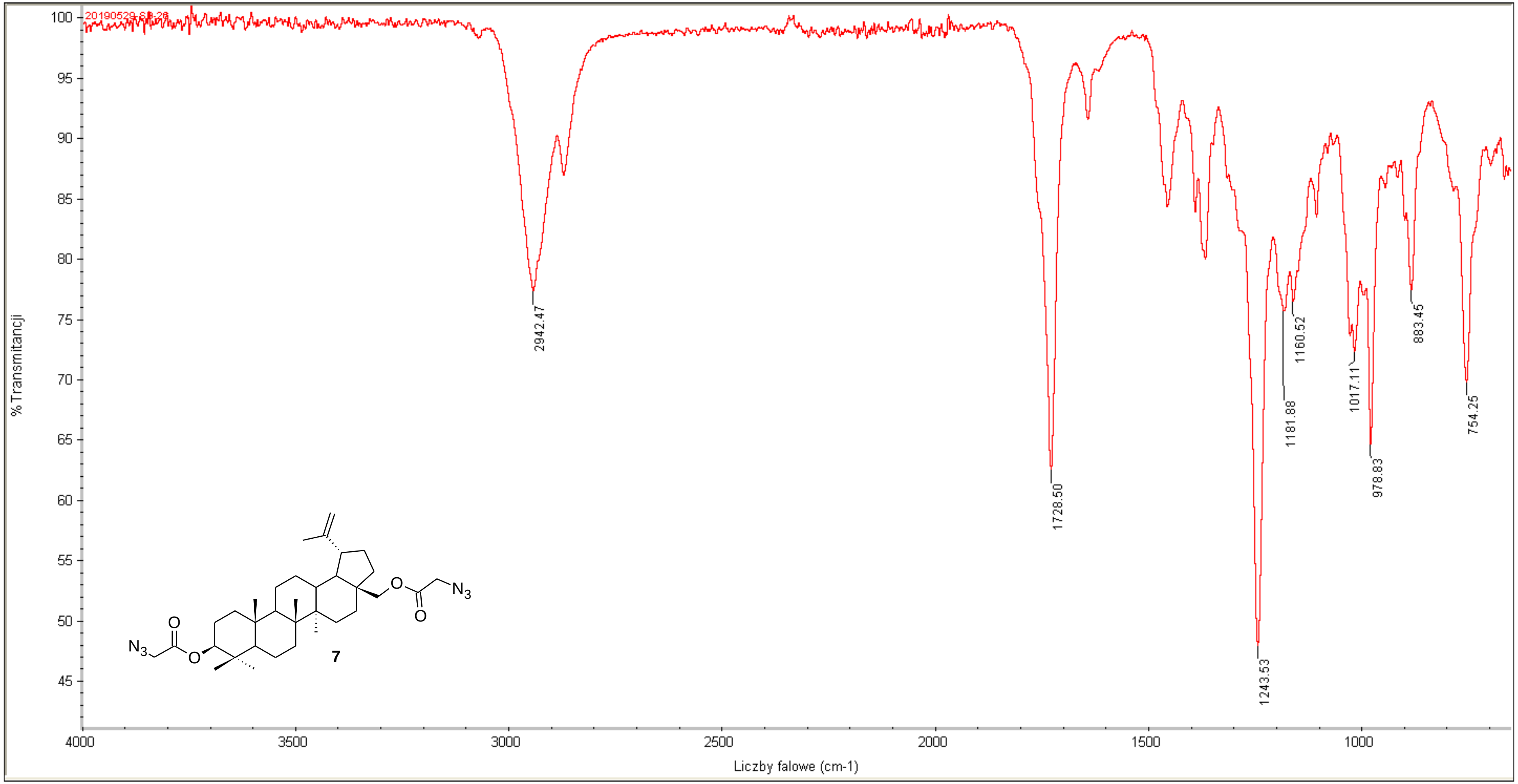

IR spectrum of 3,28-*O*,*O*′-di(2-azidoacetyl)betulin (**7**); ATR (cm^{-1}).

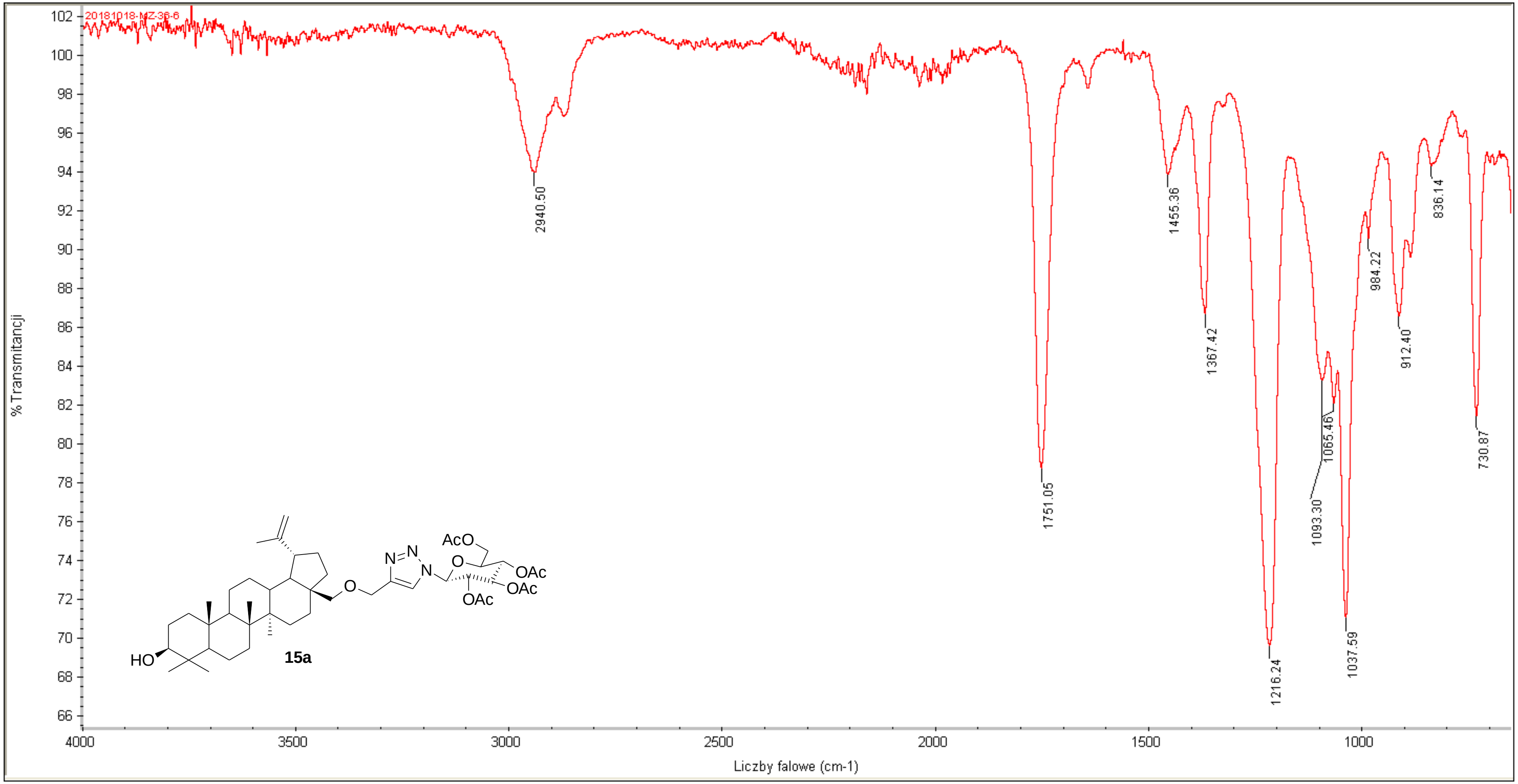

IR spectrum of glycoconjugates type **I** (**15a**); ATR (cm^{-1}).

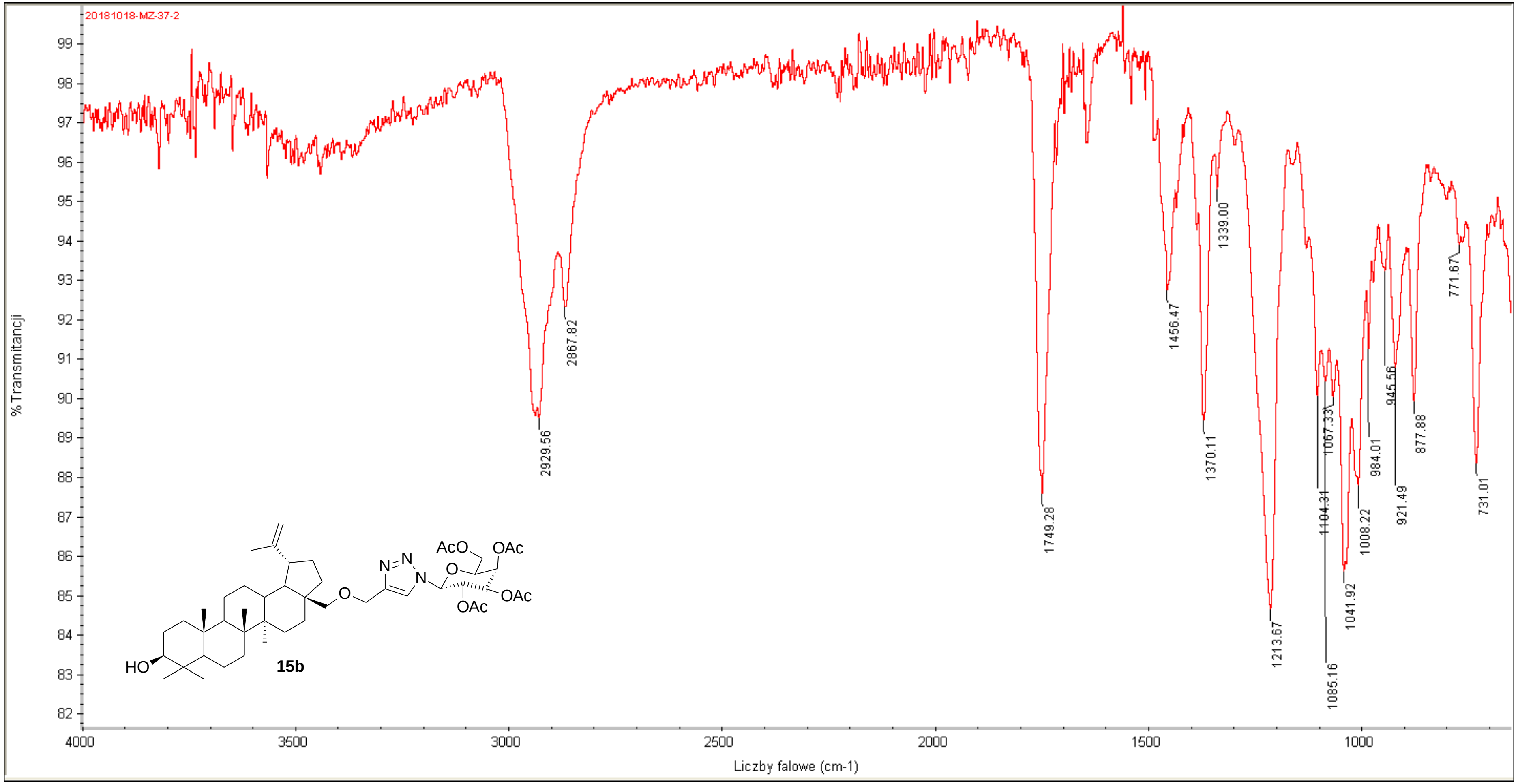

IR spectrum of glycoconjugates type **I** (**15b**); ATR (cm^{-1}).

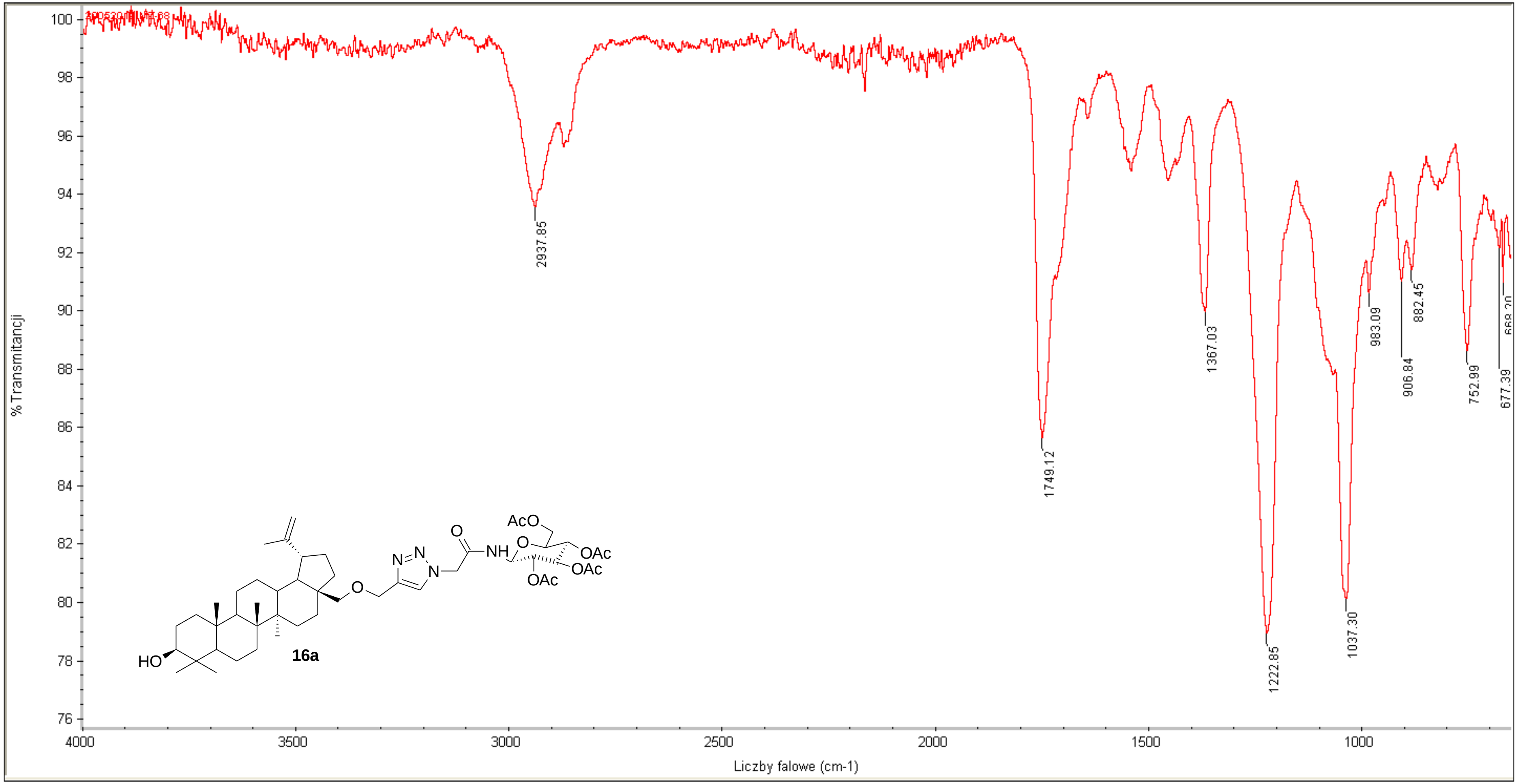

IR spectrum of glycoconjugates type **II** (**16a**); ATR (cm-1).

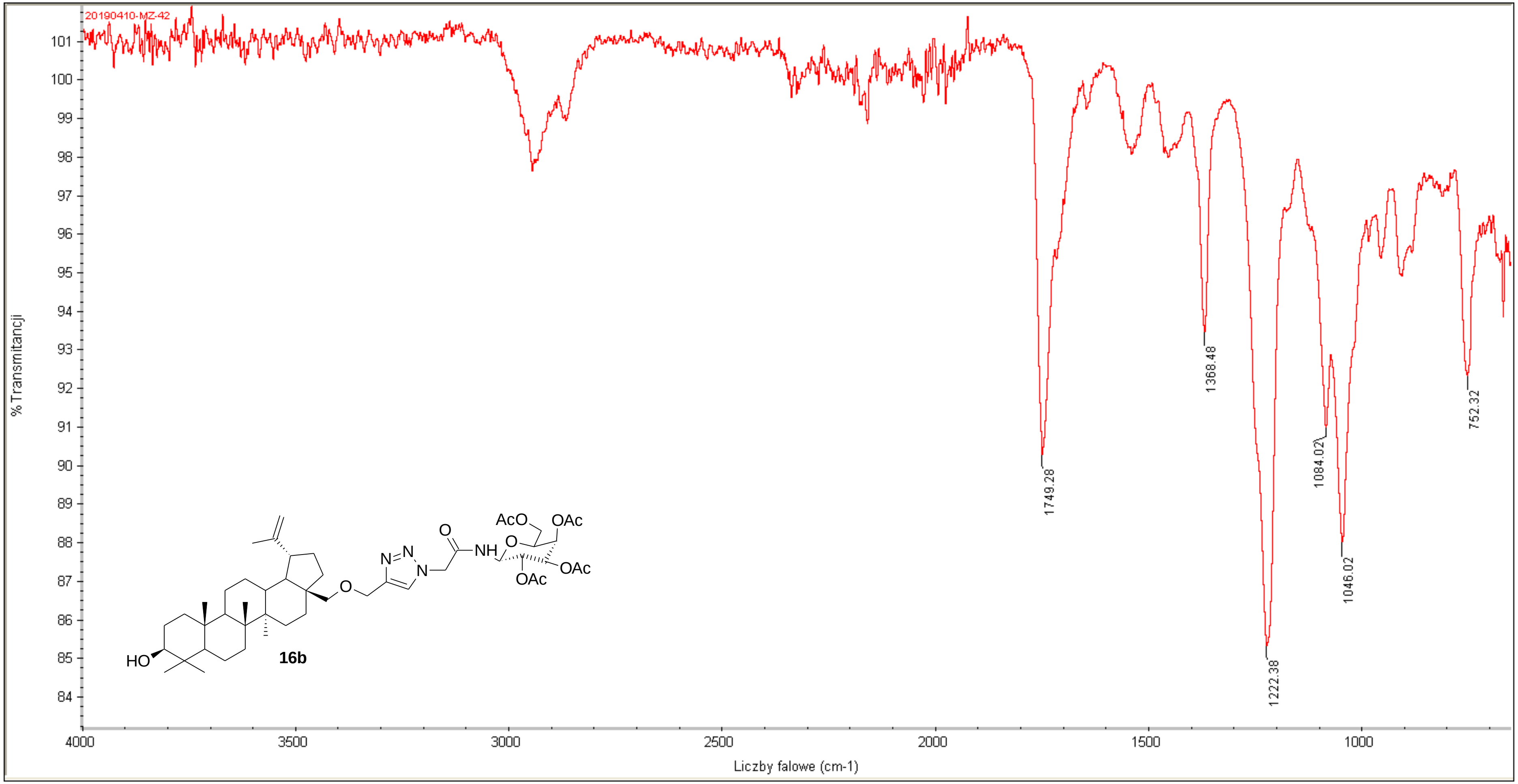

IR spectrum of glycoconjugates type **II** (**16b**); ATR (cm^{-1}).

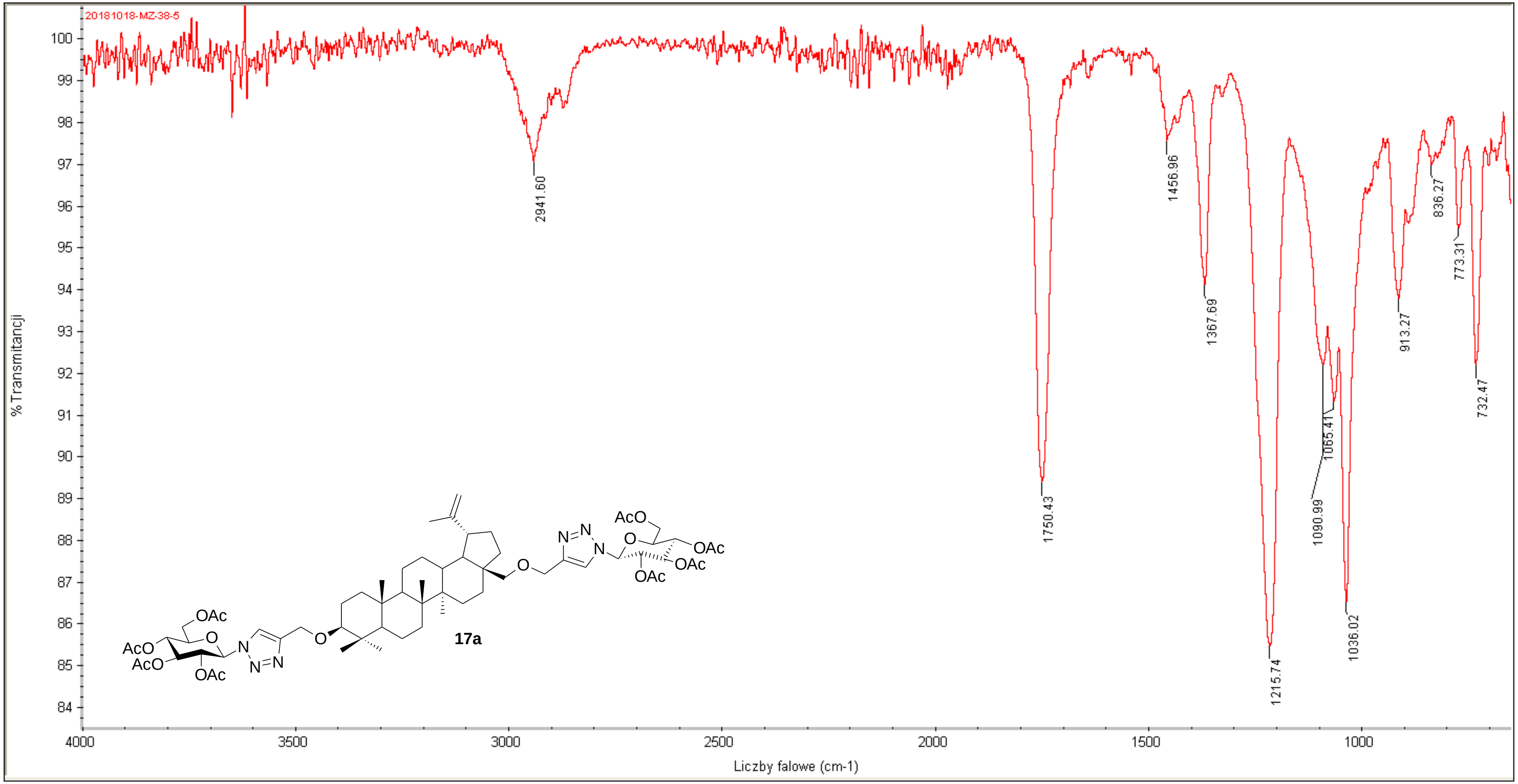

IR spectrum of glycoconjugates type **III** (**17a**); ATR (cm-1).

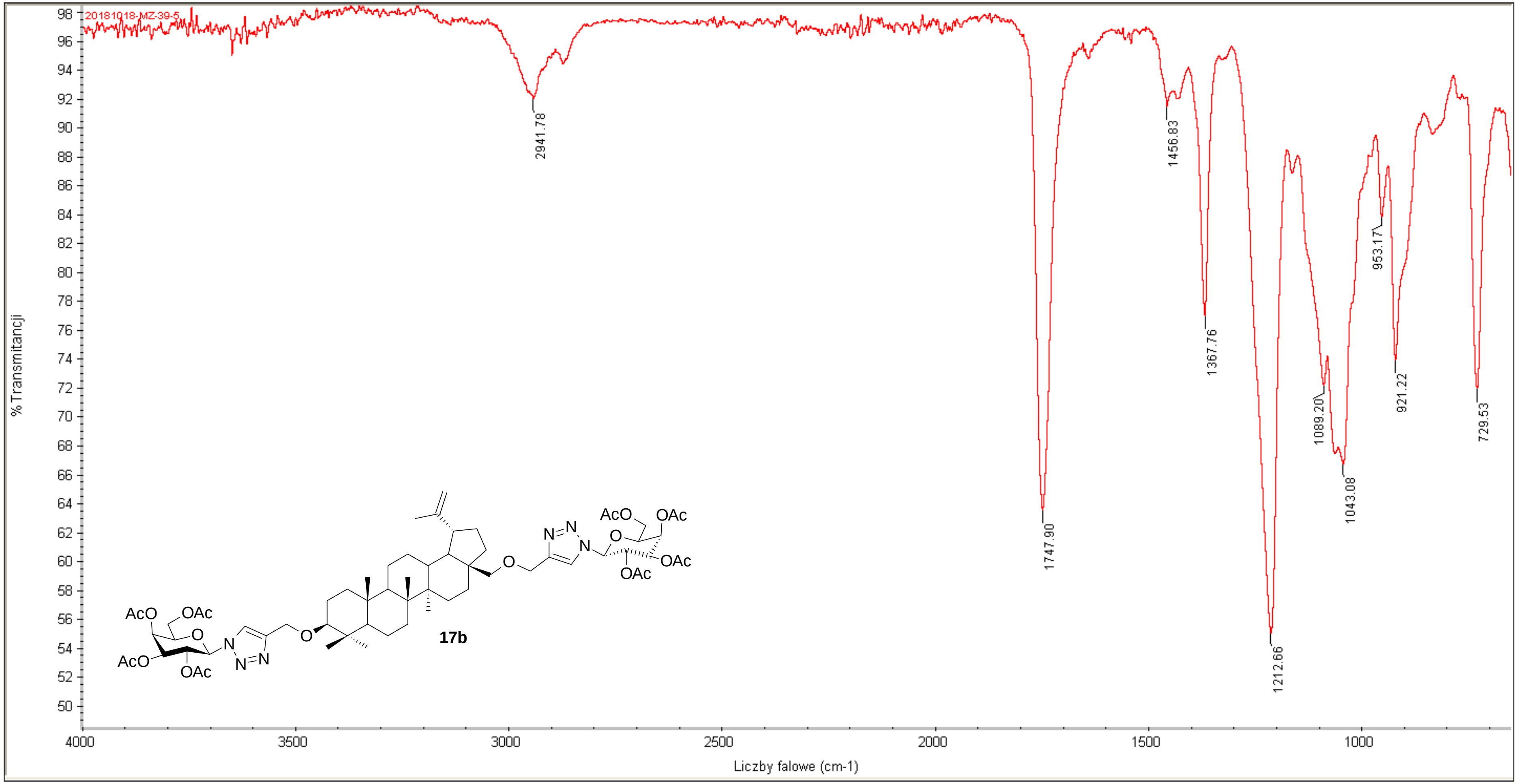

IR spectrum of glycoconjugates type **III** (**17b**); ATR (cm^{-1}).

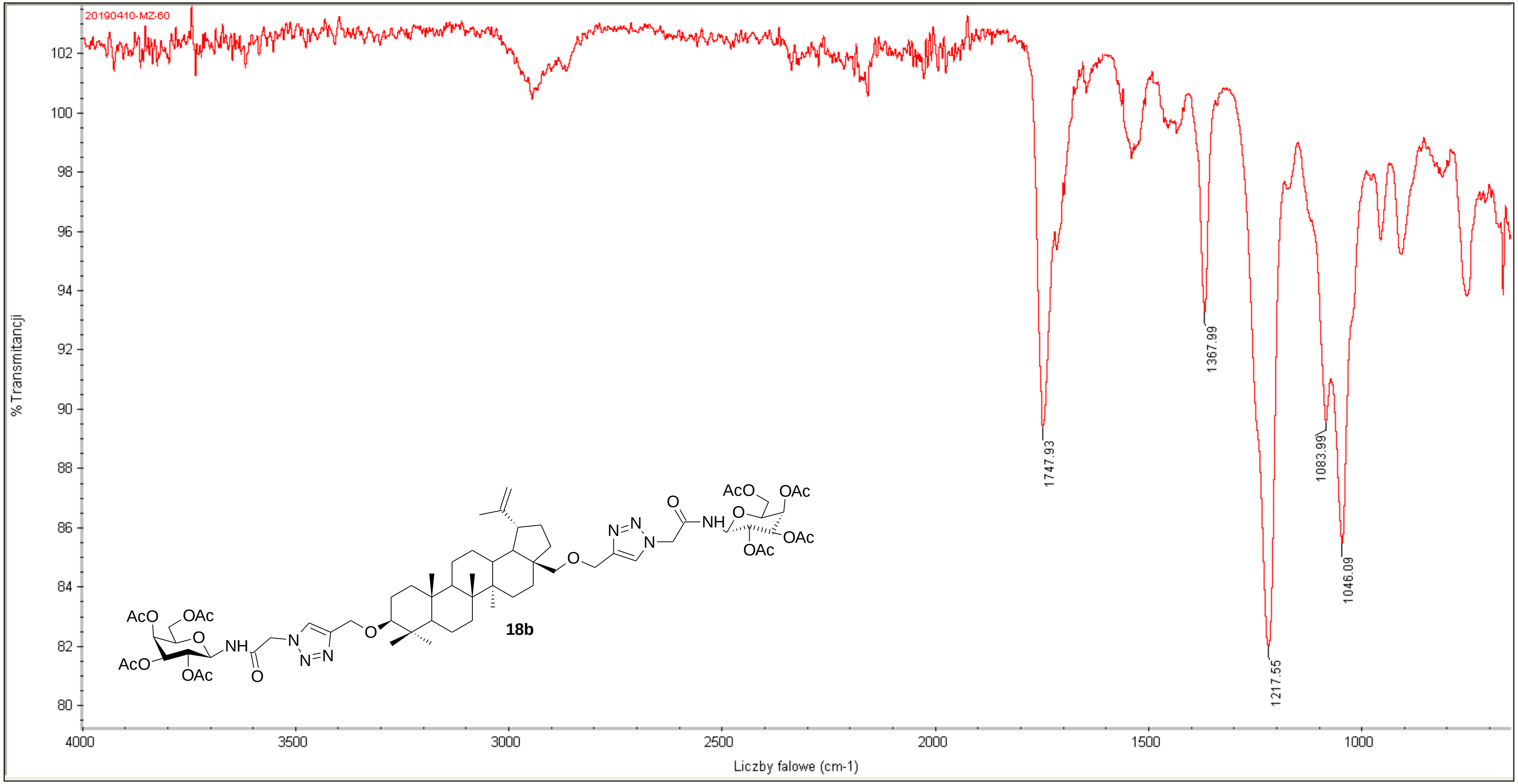

IR spectrum of glycoconjugates type **IV** (**18b**); ATR (cm-1).

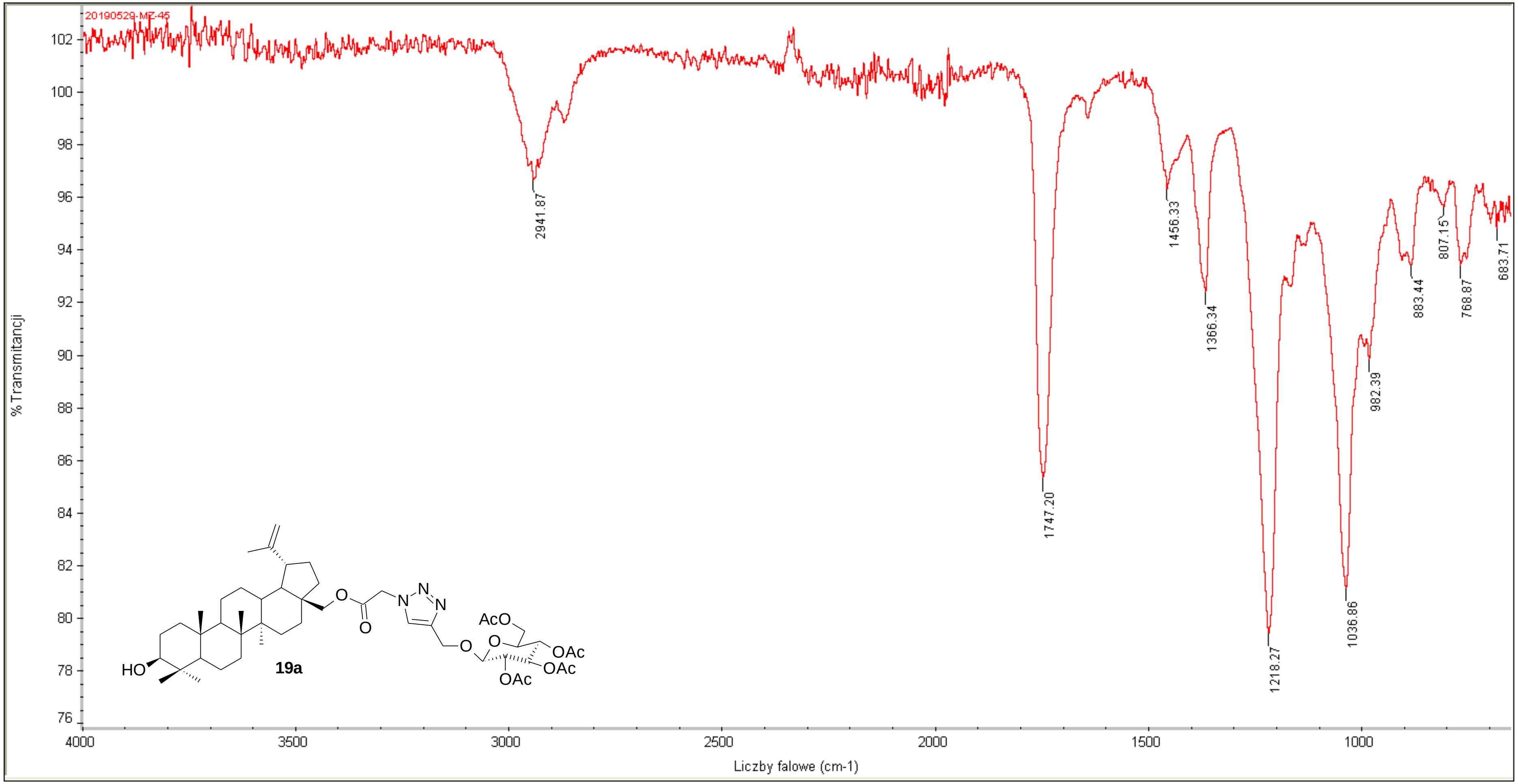

IR spectrum of glycoconjugates type **V** (**19a**); ATR (cm^{-1}).

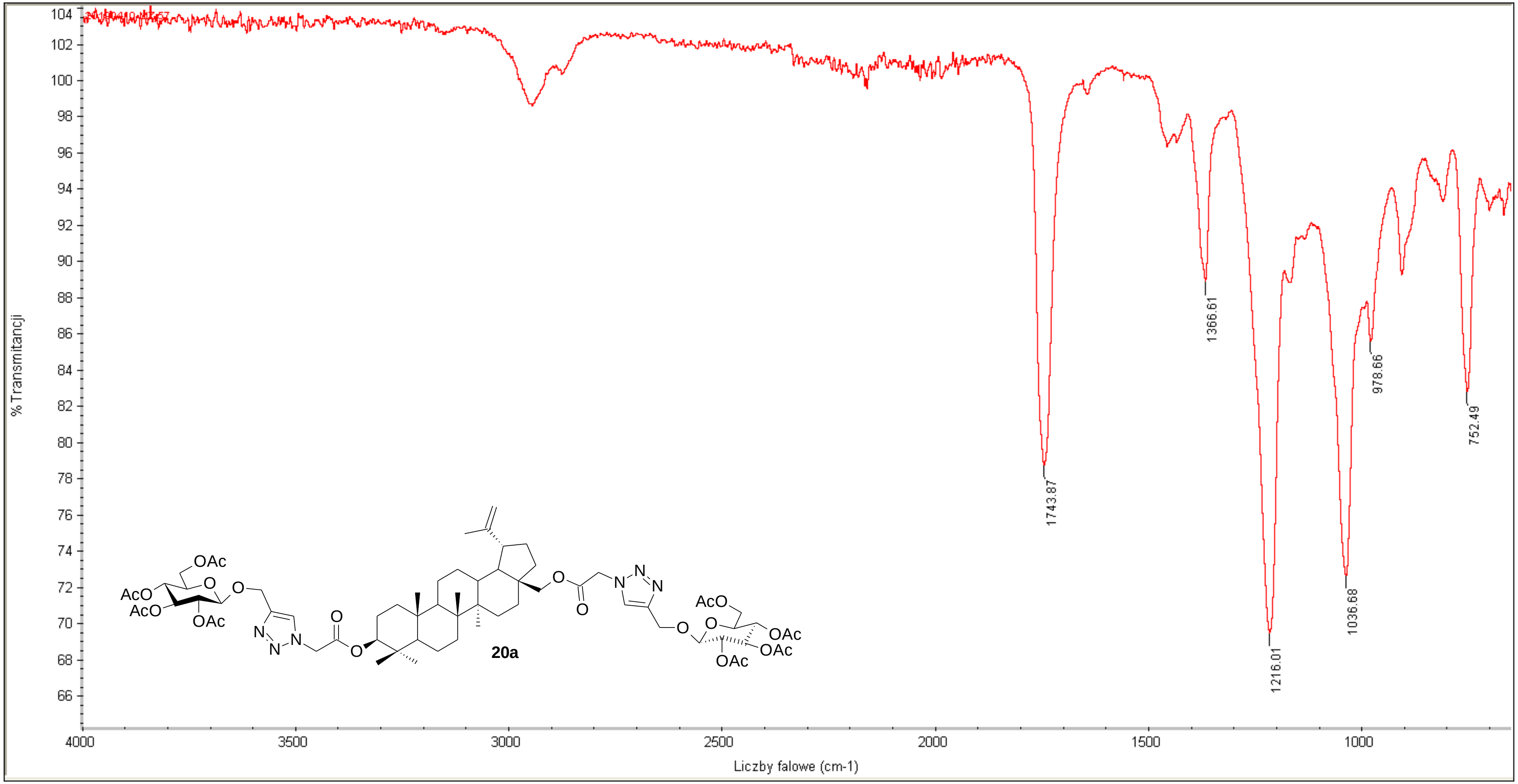

IR spectrum of glycoconjugates type **VI** (**20a**); ATR (cm^{-1}).

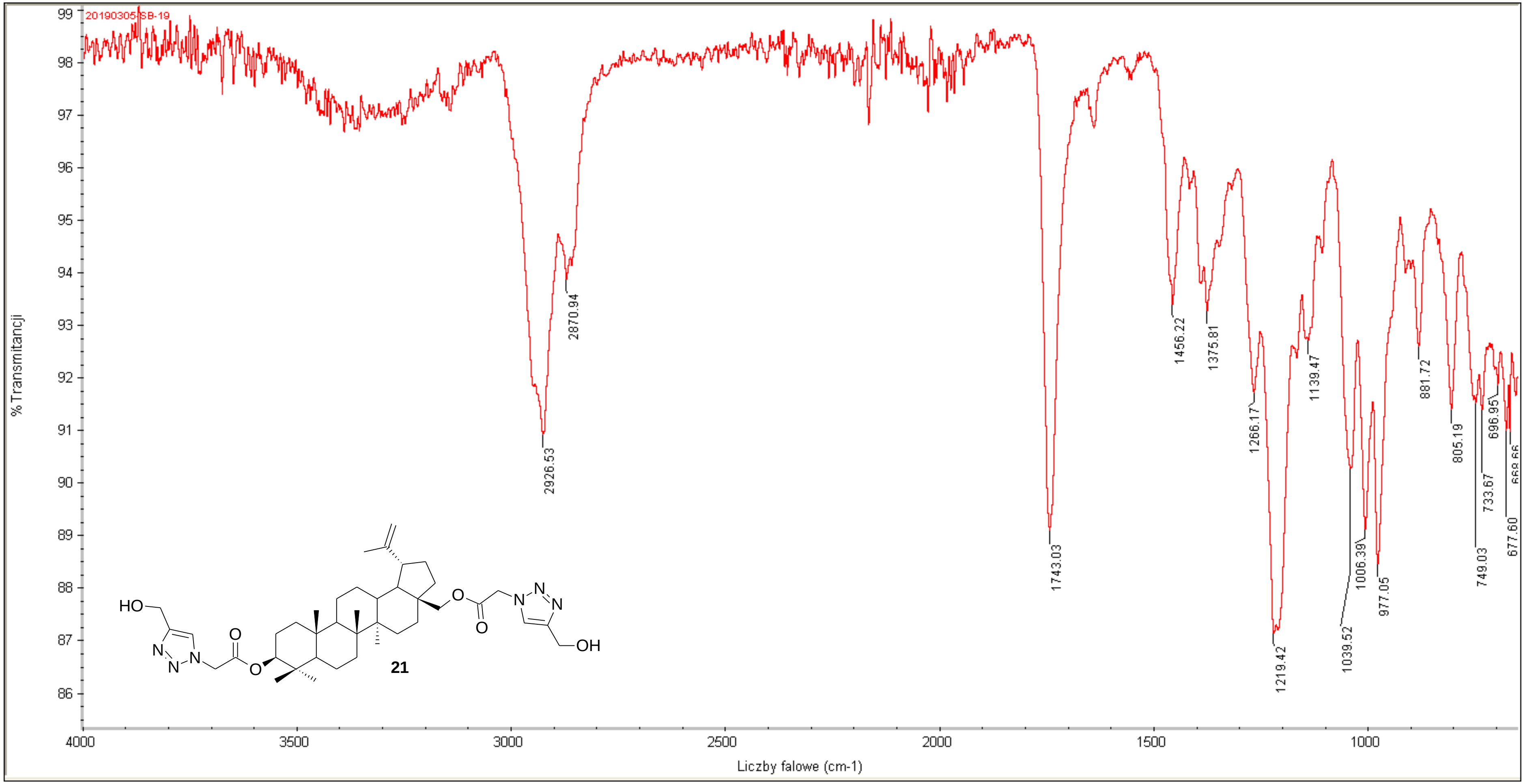

IR spectrum of compound (**21**); ATR (cm-1).

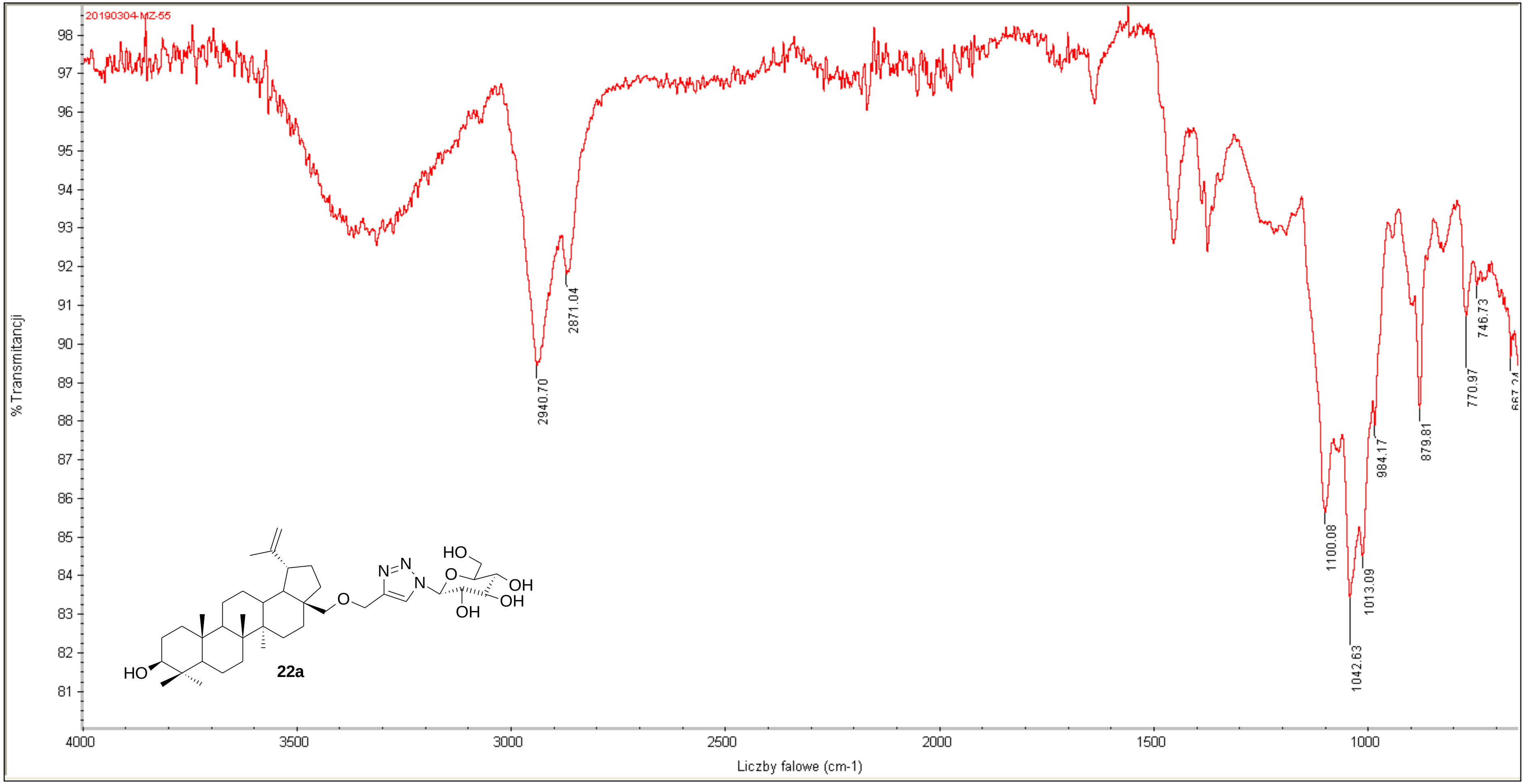

IR spectrum of glycoconjugates type **I** (**22a**); ATR (cm^{-1}).

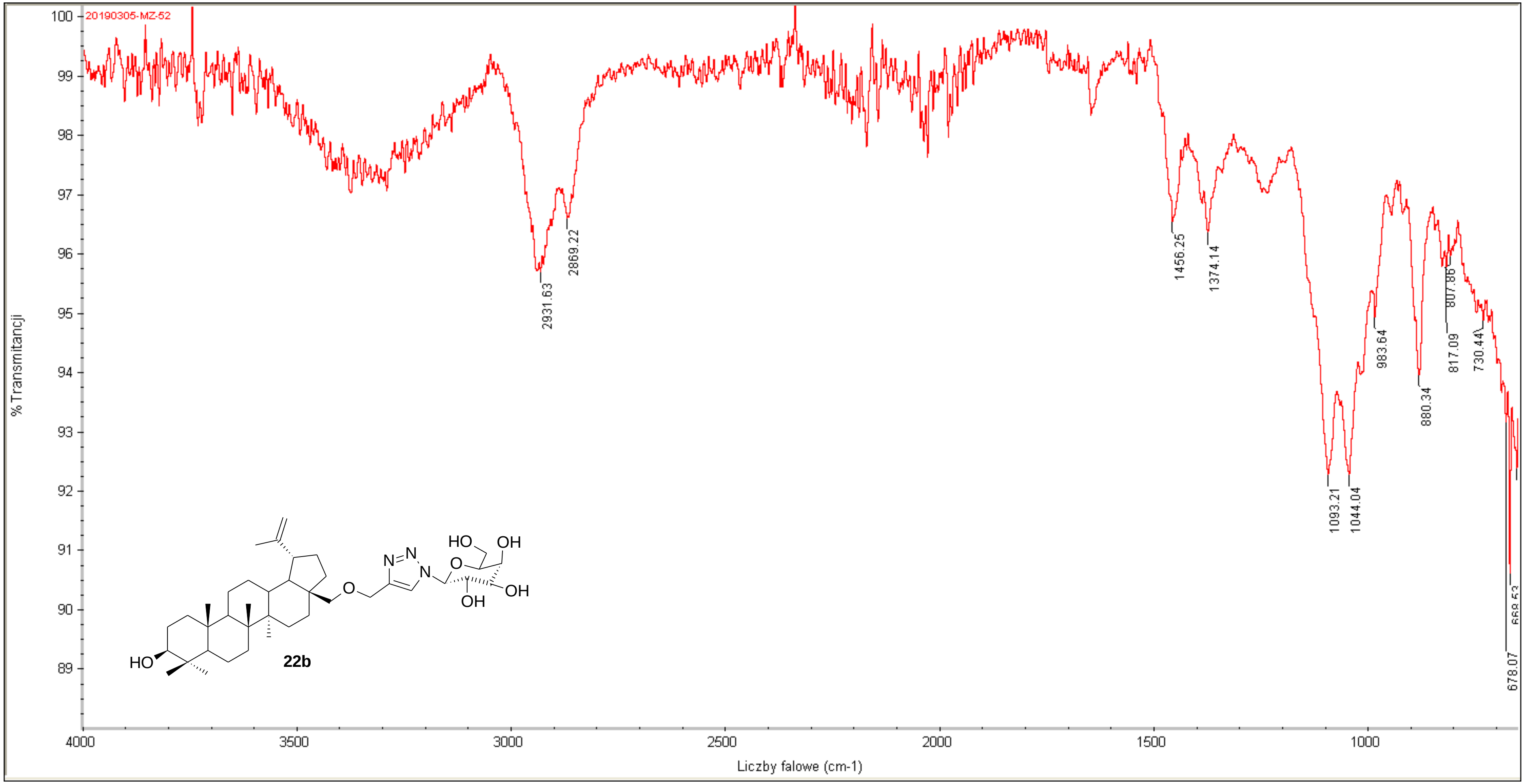

IR spectrum of glycoconjugates type **I** (**22b**); ATR (cm^{-1}).

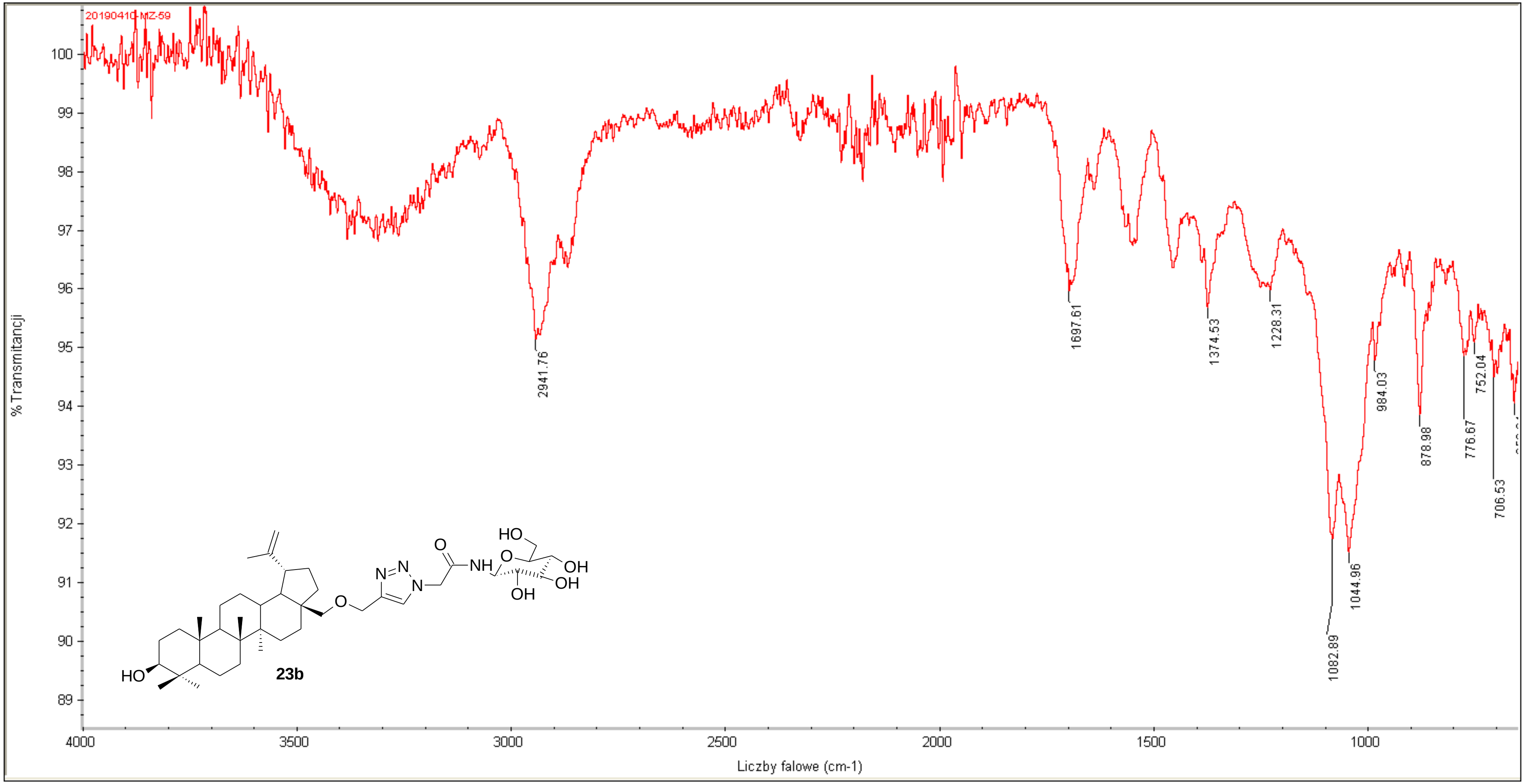

IR spectrum of glycoconjugates type **II** (**23b**); ATR (cm^{-1}).

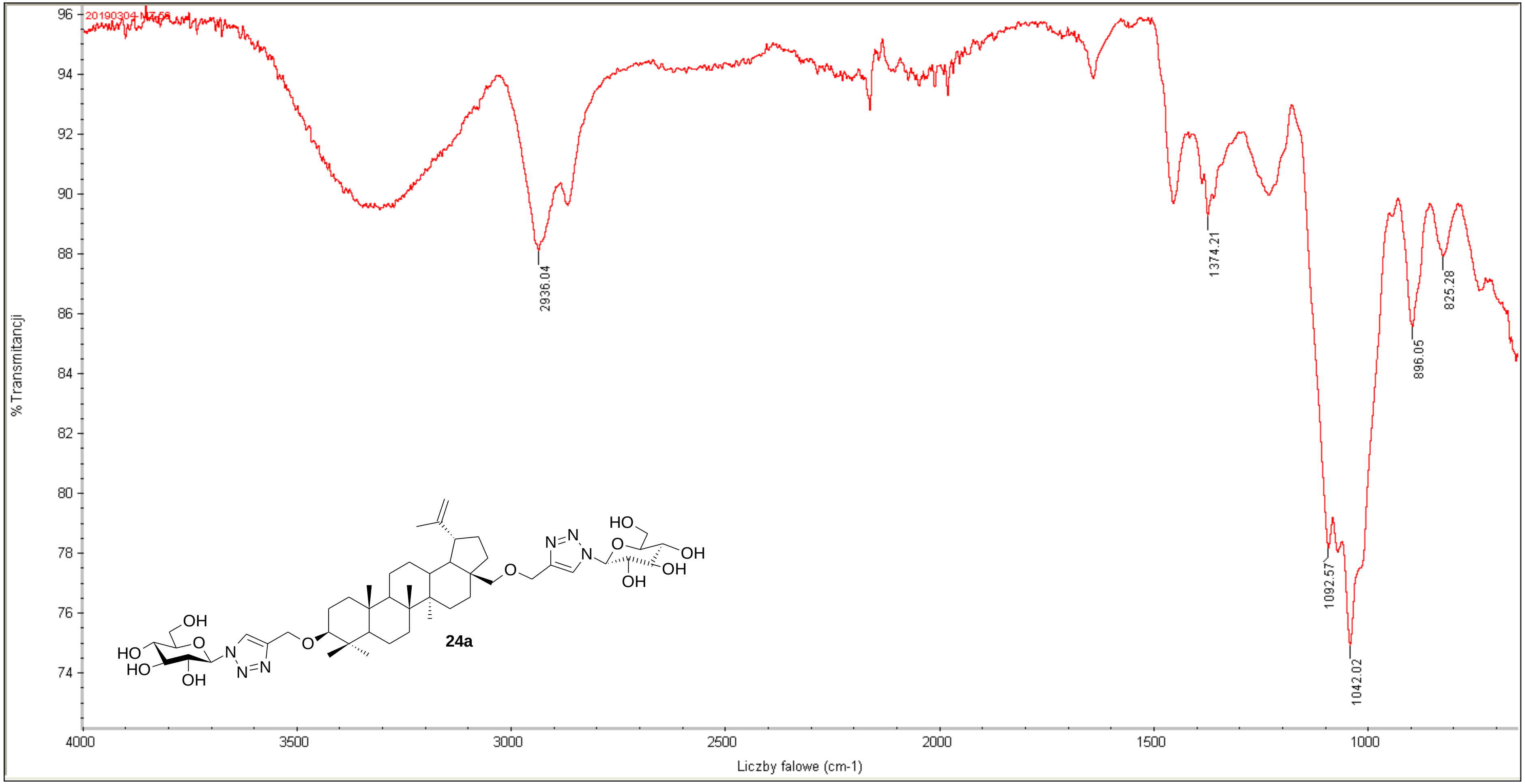

IR spectrum of glycoconjugates type **III** (**24a**); ATR (cm^{-1}).

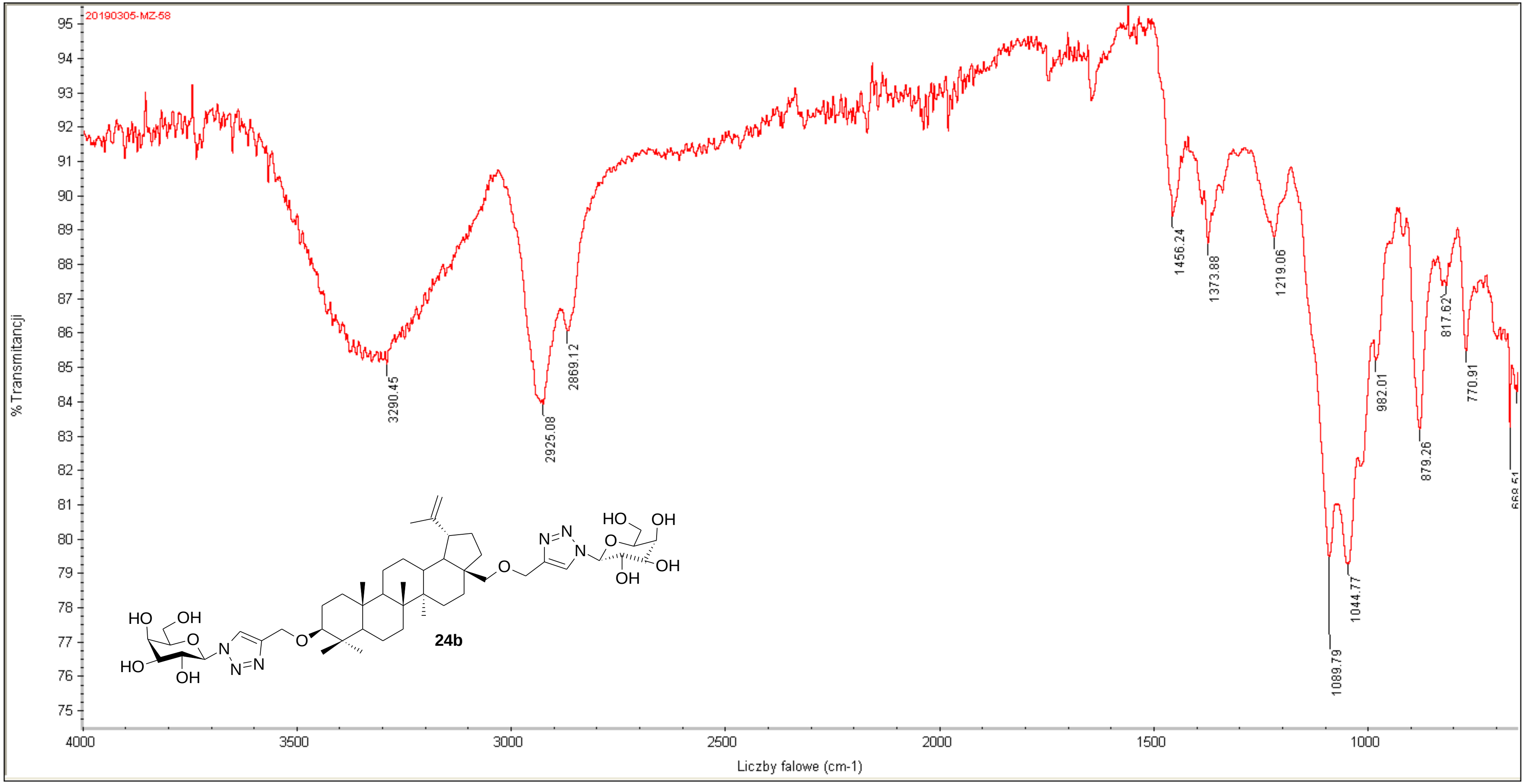

IR spectrum of glycoconjugates type **III** (**24b**); ATR (cm^{-1}).
